# Target product profiles for neonatal care devices: systematic development and outcomes with NEST360 and UNICEF

**DOI:** 10.1186/s12887-023-04342-1

**Published:** 2023-11-15

**Authors:** Rebecca P. Kirby, Elizabeth M. Molyneux, Queen Dube, Cindy McWhorter, Beverly D. Bradley, Martha Gartley, Z. Maria Oden, Rebecca Richards-Kortum, Jennifer Werdenberg-Hall, Danica Kumara, Sara Liaghati-Mobarhan, Megan Heenan, Meaghan Bond, Chinyere Ezeaka, Nahya Salim, Grace Irimu, Kara M. Palamountain, the TPP Survey, Consensus Meeting Participants Collaborative Authorship Group, Albert Manasyan, Anna Worm, Antke Zuechner, Audrey Chepkemoi, Bentry Tembo, Casey Trubo, Chishamiso Mudenyanga, Daniel Wald, David Goldfarb, Edith Gicheha, Elizabeth Asma, Emily Ciccone, Emmie Mbale, Florin Gheorghe, Guy Dumont, Helga Naburi, Jeffrey Pernica, John Appiah, Jonathan Strysko, Josephine Langton, Joy Lawn, Kate Klein, Kondwani Kawaza, Kristoffer Gandrup-Marino, Lizel Lloyd, Maggie Woo Kinshella, Mamiki Chise, Marc Myszkowski, Martha Franklin Mkony, Mary Waiyego, Matthew Khoory, Melissa Medvedev, Msandeni Chiume, Naomi Spotswood, Noah Mataruse, Norman Lufesi, Ornella Lincetto, Pascal Lavoie, Rachel Mbuthia, Rhoda Chifisi, Rita Owino, Robert Moshiro, Ronald Mbwasi, Sam Akech, Sona Shah, Steffen Reschwamm, Steve Adudans, Thabiso Mogotsi, Walter Karlen, Zelalem Demeke

**Affiliations:** 1 Kellogg School of Management, Northwestern University, Illinois, USA; 2 Department of Paediatrics, Kamuzu University of Health Sciences (Formerly College of Medicine, University of Malawi), Blantyre, Malawi; 3 Department of Paediatrics, College of Medicine, University of Malawi, Blantyre, Malawi and Department of Paediatrics, Queen Elizabeth Central Hospital, Blantyre, Malawi; 4 UNICEF Supply Division, Copenhagen, Denmark; 5 Clinton Health Access Initiative, Toronto, Canada; 6 Rice360 Institute for Global Health Technologies, Rice University, Texas, USA; 7 3rd Stone Design Inc, San Rafael, CA USA; 8 Department of Paediatrics, College of Medicine, University of Lagos, Lagos, Nigeria; 9 Department of Paediatrics and Child Health, Muhimbili University of Health and Allied Sciences, Dar Es Salaam, Tanzania; 10 Department of Paediatrics and Child Health, University of Nairobi, Nairobi, Kenya

**Keywords:** Newborn, Low- and middle-income countries, Target product profile, Technology, Medical devices

## Abstract

**Background:**

Medical devices are critical to providing high-quality, hospital-based newborn care, yet many of these devices are unavailable in low- and middle-income countries (LMIC) and are not designed to be suitable for these settings. Target Product Profiles (TPPs) are often utilised at an early stage in the medical device development process to enable user-defined performance characteristics for a given setting. TPPs can also be applied to assess the profile and match of existing devices for a given context.

**Methods:**

We developed initial TPPs for 15 newborn product categories for LMIC settings. A Delphi-like process was used to develop the TPPs. Respondents completed an online survey where they scored their level of agreement with each of the proposed performance characteristics for each of the 15 devices. Characteristics with < 75% agreement between respondents were discussed and voted on using *Mentimeter™* at an in-person consensus meeting.

**Findings:**

The TPP online survey was sent to 180 people, of which 103 responded (57%). The majority of respondents were implementers/clinicians (51%, 53/103), with 50% (52/103) from LMIC. Across the 15 TPPs, 403 (60%) of the 668 performance characteristics did not achieve > 75% agreement. Areas of disagreement were voted on by 69 participants at an in-person consensus meeting, with consensus achieved for 648 (97%) performance characteristics. Only 20 (3%) performance characteristics did not achieve consensus, most (15/20) relating to quality management systems. UNICEF published the 15 TPPs in April 2020, accompanied by a report detailing the online survey results and consensus meeting discussion, which has been viewed 7,039 times (as of January 2023).

**Conclusions:**

These 15 TPPs can inform developers and enable implementers to select neonatal care products for LMIC. Over 2,400 medical devices and diagnostics meeting these TPPs have been installed in 65 hospitals in Nigeria, Tanzania, Kenya, and Malawi through the NEST360 Alliance. Twenty-three medical devices identified and qualified by NEST360 meet nearly all performance characteristics across 11 of the 15 TPPs. Eight of the 23 qualified medical devices are available in the UNICEF Supply Catalogue. Some developers have adjusted their technologies to meet these TPPs. There is potential to adapt the TPP process beyond newborn care.

**Supplementary Information:**

The online version contains supplementary material available at 10.1186/s12887-023-04342-1.

## Key findings

**Table:** 

**WHAT WAS KNOWN?**
• Many lifesaving newborn technologies that exist in high-income countries are not currently available in low- and middle-income countries because they are either ineffective, unaffordable, or not easy to use • The gap in effective, affordable, and easy-to-use newborn technologies could be solved by new product development specifically designed for resource-limited settings. Target Product Profiles (TPPs) are developed early in the medical device and diagnostic development process to help product manufacturers understand the unmet market needs
**WHAT WAS DONE THAT IS NEW?**
• We used a Delphi-like process to develop TPPs for neonatal care devices, applying four steps: (i) Reviewed WHO guidelines to identify medical devices and diagnostics, or product categories, needed to provide WHO level-2 care for small and sick newborns (including continuous positive airway pressure) resulting in 15 product categories (ii) drafted minimal and optimal performance characteristics for each of the 15 product categories (iii) conducted a survey of performance characteristics including respondents with clinical, technical, and industry expertise (iv) held a meeting for voting and discussions for items where the survey did not achieve consensus • This approach was novel in scope – applying the TPP process to newborn technologies, breadth – the large number of TPPs developed at once, and reach – the number of countries and types of stakeholders included throughout the process. Establishing a unique methodology to apply the TPP process to medical technologies for newborn care demonstrates the potential to replicate this approach for other global public health burdens • This manuscript differs from the 232-page report published on the UNICEF website outlining all of the final TPPs as it showcases the value of the process and the methodology followed in an abbreviated fashion
**WHAT WAS FOUND?**
• In total, over 180 organisations/individuals were asked to participate in the survey, of whom 103 responded (response rate, 57%). Sixty-nine stakeholders attended the in-person consensus meeting • Fifteen TPPs were drafted with a total of 668 performance characteristics, of which 403 were discussed at the consensus meeting as they did not achieve consensus through the survey. Following the consensus meeting, 97% (648) achieved consensus. Only 3% (20/668) of performance characteristics did not ultimately achieve consensus
**WHAT NEXT?**
• UNICEF published the 15 TPPs in April 2020, accompanied by a report detailing the online survey results and consensus meeting discussion, which has been viewed 7,039 times (as of January 2023) • While the development of TPPs generally has proven helpful in the diagnostics and pharmaceutical development space, it was novel to use the process for medical devices for newborn care. In our exercise, 15 newborn care TPPs were developed, and the process helped facilitate dialogue between the supply (manufacturer and product developer) and demand (healthcare user) sides. Given our learning, there is potential to adapt and apply the TPP development process to other fields beyond newborn care

## Background

Every year, 2.3 million newborns die, and 30 million small and sick newborns require hospital care worldwide [1]. Hospital-based care for small and sick newborns has the potential to avert approximately 750,000 neonatal deaths each year [2–4]. Despite the Sustainable Development Goals (SDGs) target for each country to reduce neonatal mortality to < 12 deaths per 1,000 live births by 2030, 63 countries remain off track [5]. To accelerate progress, the *Every Newborn Action Plan* (ENAP) calls for countries to establish at least one unit providing level-2 care plus respiratory support with continuous positive airway pressure (CPAP) (simply referred to as level-2 care in this paper) in 80% of subnational units (e.g., districts) by 2025 [2]. Standard care at this level includes thermal care, kangaroo mother care (KMC) for all stable neonates weighing < 2000g, assisted feeding and intravenous fluids, safe administration of oxygen, neonatal sepsis management with injected antibiotics, management of neonatal jaundice with phototherapy, management of neonatal encephalopathy, detection of congenital abnormalities and referral or management of congenital disabilities. In addition, care during the transition from level-2 includes management of respiratory distress with CPAP [1]. Achieving coverage and ensuring care provided is of high-quality requires the right space, the right people, and the right devices and diagnostics [6, 7].

While medical devices are a crucial component of high-quality, hospital-based care of small and sick newborns, many lifesaving newborn technologies that have existed in high-income countries for decades are not widely available in low- and middle-income countries (LMIC) [8]. Furthermore, technologies designed for use in high-income settings may not meet the operating environment found in LMIC, failing to address context-specific conditions affecting device performance (e.g., dust, extreme temperatures, humidity, frequent power outages, etc.) that must be considered when deciding which devices to procure for LMIC [9, 10]. To address these disparities in the availability of technology, the development of effective, affordable, rugged devices is a priority for reducing deaths and disabilities in preterm babies [8, 11, 12]. To reduce the three leading causes of neonatal death (prematurity, intrapartum-related complications, and infection), a bundle of tools for diagnosis and treatment is required across six main functions or pathways of care: (i) provide hydration and nutrition, (ii) prevent and treat infections, (iii) provide temperature stability, (iv) provide breathing support, (v) monitor and treat jaundice, and (vi) monitor and treat hypoglycaemia [11].

The World Health Organization (WHO) defines appropriate healthcare technologies as those that are scientifically valid, adapted to local needs, accepted by users and recipients, and maintainable with local resources [13]. Toward the WHO’s recommendation for appropriate healthcare technologies, NEST360, an international alliance of 17 organisations, and four governments (Kenya, Malawi, Nigeria, Tanzania) united to reduce preventable newborn deaths in African hospitals. Working in support of SDG 3.2 (Newborn and child mortality: By 2030, end preventable deaths of newborns and children under 5 years of age, with all countries aiming to reduce neonatal mortality and under‑5 mortality), we began a process of defining medical device and diagnostic characteristics for hospital-based newborn care in LMIC. This work, which is part of a partnership with African governments to implement a package of care that includes affordable, suitably designed, high-quality technologies to improve quality of care, developed into the Target Product Profiles (TPPs) discussed in this manuscript.

The objective of these TPPs was to create consensus among users, buyers, and implementers (the demand side) on the ideal performance characteristics for LMIC and communicate these to product developers to support innovation (the supply side). A TPP is a strategic document that summarises the key features of an innovation needed to address an unmet need. A TPP outlines the desired characteristics of a target product by defining the intended use, target population(s) and other desired attributes, including safety and efficacy-related characteristics. TPPs are often used early in the medical device and diagnostic development process to help product manufacturers and industry understand the unmet market needs. They can also be used by regulatory agencies. For example, the Food and Drug Administration (FDA) has used TPPs to facilitate communication between the pharmaceutical industry, the agency, and other stakeholders outside of industry to aid new drug development [14]. TPPs can also be used as guiding documents in the public health sector to define an unmet need in the hopes of stimulating innovation, an important goal of the newborn TPPs outlined in this paper [15]. For example, the TPP process has been applied to other disciplines, such as diagnostics for tuberculosis, hepatitis C, and others [16–19].

This paper aims to outline the systematic process used to develop TPPs for newborn categories. These TPPs were developed to address the unmet need for providing WHO level-2 quality care for small and sick newborns, including the provision of respiratory support with CPAP. Within six pathways of care, 15 product categories were identified as necessary to manage the care of small and sick newborns, and TPPs were created for each of these product categories (Table 1). A product category is a broader term that consists of both medical devices (e.g., syringe/infusion pump, phototherapy, CPAP, flow splitter, oxygen concentrator, pulse oximeter, respiratory rate/apnea monitor, suction pump, radiant warmer, temperature monitor, conductive warmer) or a diagnostic (e.g., bilirubinometer, glucometer, haemoglobinometer, pH monitor). Therefore, of the 15 product categories, 11 were medical devices and 4 were diagnostics. The 15 product categories and a brief description of their clinical role include: Syringe Pump (delivering medication and small quantities of fluids continuously through an intravenous line), Bilirubinometer (point-of-care (POC) tool to guide treatment of infants receiving phototherapy), Phototherapy (treatment with blue light to prevent morbidity and mortality for severe cases of neonatal jaundice), Glucometer (POC tool to evaluate glucose levels), Haemoglobinometer (POC tool to evaluate haemoglobin concentration), pH Monitor (POC tool to evaluate pH), CPAP (provides treatment for neonatal respiratory distress by delivering a blended mix of air and oxygen to the neonate), Flow Splitter (allows the output of a concentrator or other oxygen source to be split between multiple patients while independently monitoring and adjusting each flow rate), Oxygen Concentrator (device that can concentrate oxygen from the air for use with a multitude of devices), Pulse Oximeter (non-invasive sensor to measure pulse rate (PR) and blood oxygenation levels (SpO2)), Respiratory Rate/Apnea Monitor (technology to measure respiratory rate and detects periods of non-breathing, Suction Pump (technology to clear a neonate’s airway through the use of a suction pump), Radiant Warmer (technology to administer and control heat to stabilise neonate’s temperature), Temperature Monitor (tool to monitor neonate’s temperature), and Conductive Warmer (technology providing conductive heating while also allowing healthcare workers with visibility and access to the baby).

**Table 1:** Product categories for newborn target product profiles

Product Categories for Newborn Target Product Profiles:	
**Hydration, Nutrition, and Drug Delivery** Small and sick babies have special fluid and nutritional requirements	
1. Syringe Pump	Syringe pumps deliver medication and small quantities of fluids continuously through an intravenous line
**Jaundice Management** Most neonates, term and preterm, will have elevated levels of unconjugated bilirubin and some amount of jaundice during the first one to two weeks of life	
2. Phototherapy	Treatment with blue light phototherapy is necessary to prevent morbidity and mortality for severe cases of neonatal jaundice
3. Bilirubinometer	All infants should have a laboratory evaluation of serum bilirubin both to diagnose jaundice and to guide treatment of infants receiving phototherapy
**Point-of-Care Diagnostics** Access to diagnostic laboratories remains a key challenge in low-resource settings	
4. Glucometer	Monitoring glucose levels to detect hypoglycemia, a common metabolic problem in newborns that can result in neurologic complications if left untreated
5. Haemoglobinometer	Haemoglobin concentration refers to the amount of the oxygen-carrying protein in the blood and is a diagnostic for anaemia (low haemoglobin) or polycythemia (high haemoglobin)
6. pH Monitor	pH is an important blood gas measurement that assesses the acid–base status of the blood
**Respiratory Support** Upon birth, a baby's lung must transition from fetal to neonatal life in three key ways: 1) fluid in the lungs must be absorbed and replaced with air, 2) lungs must expand fully and regular breathing must be established, and 3) pulmonary blood flow is increased	
7. Continuous positive airway pressure (CPAP)	bCPAP provides treatment for neonatal respiratory distress by delivering a blended mix of air and oxygen to the neonate
8. Flow Splitter	A flow splitter allows the output of a concentrator or other oxygen source to be split between multiple patients while independently monitoring and adjusting each flow rate
9. Oxygen Concentrator	An oxygen concentrator is a device able to concentrate oxygen from the air for use with a multitude of devices
10. Pulse Oximeter	Pulse oximeters use a non-invasive sensor to measure pulse rate (PR) and blood oxygenation levels (SpO2) (i.e., percentage of oxygenated haemoglobin in arterial blood)
11. Respiratory Rate/Apnea Monitor	Technology to measure respiratory rate and detect periods of non-breathing
12. Suction Pump	Clinicians periodically need to clear a neonate’s airway using a suction pump
**Thermal Management** A newborn's ability to stay warm can be easily compromised by the temperature of its surroundings since newborn infants regulate body temperature much less efficiently than adults and lose heat more easily	
13. Radiant Warmer	Hypothermia can be prevented using radiant warmers that carefully control heat based on manual settings or the neonate’s temperature
14. Temperature Monitor	Given that temperatures less than 36.5 °C have been shown to be an independent risk factor for death in neonates, early recognition and treatment of hypothermia is critical
15. Conductive Warmer	Conductive warmers provide conductive heating either below or around the patient while also allowing healthcare workers with visibility and access to the baby

## Methods

We adopted a systematic approach to develop TPPs for neonatal care devices [16] applying four steps: (i) Reviewed WHO guidelines to identify medical devices, or product categories, needed to provide WHO level-2 care for small and sick newborns [2] resulting in 15 product categories (ii) Drafted minimal and optimal performance characteristics for each of the 15 product categories (iii) Surveyed global stakeholders with clinical, technical, and industry expertise using a Delphi-like survey method soliciting input on performance characteristics (iv) Held an in-person meeting for discussion of items where the survey did not achieve consensus and voted using *Mentimeter™* (Mentimeter Software, Stockholm, Sweden).

### Step 1: Reviewed WHO guidelines to identify medical devices or product categories needed to provide level-2 care for small and sick newborns

The three main causes of neonatal death (prematurity, intrapartum-related complications, and infection) require a bundle of tools for diagnosis and treatment which Maynard et al. classified across six main functions or pathways of care [11]. Within six pathways of care, 15 product categories were identified as necessary to manage the care of small and sick newborns. The standards for improving the quality of care for small and sick newborns in health facilities [20, 21] and national guidelines for the care of small and sick newborns in Malawi, Kenya, Nigeria, [22] and Tanzania were reviewed to identify medical devices commonly recommended for inpatient level-2 CPAP care for small and sick newborns.

### Step 2: In conjunction with experts, drafted minimal and optimal performance characteristics for each of the 15 product categories

The performance characteristics were determined following a review of other TPPs for medical devices and diagnostics [18, 23–25]. The key characteristics of TPPs for which individual specifications were defined fell under five domains, i.e. (i) scope of test / device and safety standards, (ii) purchasing considerations, (iii) training and maintenance, (iv) utility requirements, and (v) technical characteristics (Table 2). The technical characteristics were consistent across diagnostic product categories (Bilirubinometer, Glucometer, Haemoglobinometer, Ph Monitor) but varied for other product categories. The data sources informing the individual product category specifications in the TPPs included expert consultation, literature review, field observations, available guidelines, and standards and package inserts [26–32]. Minimal characteristics refer to the lowest acceptable requirements met by all devices. Optimal performance characteristics refer to the ideal targets that products should aim to achieve. The optimal and minimal characteristics are uniquely defined on each individual TPP per attribute. The product development timeline envisioned was four years (i.e., the bounds of characteristics were set with the assumption of a maximum 4-year development timeline). The four-year time horizon for development was chosen in consideration of the 2030 ENAP Target (80% of districts having at least one functional WHO level-2 inpatient unit to care for small and sick newborns by 2030). To achieve the goal and ensure that devices would be available well in advance, an ambitious timeline of four years to develop and six years to scale in roughly 10 years was estimated.

**Table 2:** Performance characteristics defined for each TPP

**Product Categories**	**Performance Characteristics**^a^
All 15 Product Categories	**Scope of Test / Device and Safety Standards** Intended Use; Target Operator; Target Population; Target Setting; Quality Management; Regulation **Purchasing Considerations** Instrument Pricing; Consumable Pricing **Training and Maintenance** User Instructions; Warranty; Decontamination **Utility Requirements** Power Source; Battery; Voltage
**Product Categories**	**Technical Characteristics**
1. Syringe Pump	Benchtop Measurement Accuracy (for Flow Rate); Flow Rate Requirements; Occlusion Detection; Syringe Requirements; Drug Library; Alarm Characteristics; Size; Weight
2. Phototherapy	Irradiance; Effective Treatment Area; Peak Wavelength; Light Source; Bulb Lifetime; Ease of Replacing Bulbs; Irradiance Meter
3. Bilirubinometer	Linear Range; Accuracy; Results; Format; Result Units; Precision; Sample; Calibration; Kit Stability & Storage; Equipment Required
4. Glucometer	
5. Haemoglobinometer	
6. pH Monitor	
7. CPAP	Flow Driver; Oxygen Flow Capacity; Pressure; Total (blended) Flow; Humidification; Alarms; Accessories; Consumables
8. Flow Splitter	Air Flow per Patient; Flow Control; Number of Outputs; Indication
9. Oxygen Concentrator	Flow Meter; Minimal Flow Rate; Flow Rate; Time to Reach 95% of Specified Performance; Oxygen Purity; Alarms; Indicators; Mobility; Oxygen Monitor; Oxygen Outlet; Noise Level; Weight; Durability and Robustness; Usage Meter
10. Pulse Oximeter	Pulse rate; Pulse rate accuracy; Pulse rate resolution; Sp02 Accuracy; Sp02 Range; Alarms; Alarm Limits – PR; Alarm Limits—Sp02; Continuous Measurement; Patient Interface; Size; Weight
11. Respiratory Rate/Apnea Monitor	Apnea Detection; Respiratory Rate Accuracy; Respiratory Rate Range; Alarm; Patient Interface; Respiratory Rate Alarm Limits; Apnea Intervention
12. Suction Pump	Pressure; Bottle Capacity; Noise Level; Cleaning; Maintenance; Operation Mode
13. Radiant Warmer	Benchtop Measurement Accuracy; Clinical Measurement Accuracy; Stability; Includes Timer; Includes Scale Mobility; Time to Indicate Accurate Temperature; Uniformity; Alarm Characteristics; Alarm Limits; Operating Temperature; Patient Interface; Patient Accessibility and Visibility; Temperature Control
14. Temperature Monitor	Benchtop Measurement Accuracy; Clinical Measurement Accuracy; Time to Indicate Accurate Temperature; Alarm Characteristics; Alarm Limits; Patient Interface; Size; Weight
15. Conductive Warmer	Form Factor; Benchtop Measurement Accuracy; Conductive Surface Temperature of Baby (required if servo-controlled); Clinical Measurement Accuracy (Compare to another gold standard); Maximum CO2 Concentration (If Enclosed Device); Maximum Temperature (of the conductive surface); Humidification (If Enclosed device); Surface Temperature overshoot when the temperature control is set to its maximum setting; Time to Indicate Accurate Temperature of baby; Uniformity (If Enclosed, then uniformity of air)(If Not Enclosed, then uniformity of mattress); Alarm Characteristics; Patient Interface; Patient Accessibility and Visibility; Temperature Control; Operating Conditions

^a^Performance characteristics include some specifications

### Step 3: Surveyed global stakeholders with clinical, technical, and industry expertise using a Delphi-like survey method soliciting input on performance characteristics

An open online survey, coded in *Qualtrics*™ (Qualtrics, Seattle, Washington, and Provo, Utah, USA) and containing the 15 draft TPPs, was distributed to stakeholders. Respondents were asked to provide a statement on their level of agreement for each of the proposed characteristics. Agreement was scored on a Likert scale ranging from 1 to 5 (1 = disagree, 2 = mostly disagree, 3 = neither agree nor disagree, 4 = mostly agree, 5 = fully agree) with an option to opt-out with the selection of “Other – Do not have the expertise to comment” [33]. If participants did not agree with the characteristic (i.e., selected 3 or below), they were asked to provide an explanation with comments. Participants who agreed with the statements could also provide comments but were not explicitly asked to do so. This survey did not engage the public and/or patients at any stage of TPP development. The survey was pre-tested for usability and functionality and to gather a time estimate to complete the survey. The survey included a ‘Save and Continue’ button as well as a ‘Go Back’ button.

The process for developing these TPPs, including identifying survey respondents and consensus meeting participants, was developed in collaboration with United Nations Children's Fund (UNICEF), a UN partner that aims to help countries, particularly developing countries, build their capacity to form appropriate policies and deliver services for children and their families. A Delphi-like process was used to facilitate stakeholder consensus-building and obtain expert advice. A list of 180 potential stakeholders was identified to constitute an expert panel for the Delphi survey. The stakeholders were chosen to provide representation from (i) Implementers and Clinicians (including from Non-governmental Organisations NGOs) working to deliver neonatal care in low-resource settings; (ii) Technical agencies and researchers; (iii) Advocacy organisations/Civil society; and (iv) industry (innovators and manufacturers). These stakeholders were selected based on their work in newborn care in low- and middle-income countries with representation from the following work sectors: clinical care for neonates, maintenance of medical devices, design and manufacture of devices, leaders of neonatology and paediatric associations within LMIC, members of Newborn Technical Working Groups within Ministries of Health, and United Nations and governmental partners engaged in purchasing/distributing medical devices and diagnostics. Invitations to participate were distributed via email by NEST360 and their partner institution, UNICEF, as well as connections facilitated by other global health organisations. The invitation provided instructions, including a link to the survey and an estimated amount of time to complete the survey (20–30 min per product category). A classic Delphi process requires at least two rounds of survey ahead of an in-person meeting [34].

### Step 4: Held an in-person meeting for discussion of items where the survey did not achieve consensus and voted using Mentimeter™

An in-person consensus meeting was held in South Africa in November 2019 to build further consensus within the 15 TPPs. The main purpose of this meeting was to discuss issues on which fewer than 75% of the respondents agreed or on which a distinct subgroup disagreed. If any voting was necessary throughout the consensus-gathering meeting, > 75% was considered a majority [35]. Consensus meeting moderators presented the results and comments for the characteristics with < 75% agreement from the survey, then solicited additional feedback on each characteristic. Then, a proposed change to the TPP characteristic was discussed amongst the consensus meeting participants. If consensus was not achieved after two votes on proposed changes, meeting participants agreed to move forward, and the disagreement was noted in a publicly available meeting report.

## Results

Over 180 organisations/individuals were invited to participate in the survey, which was first disseminated in April 2019, of whom 103 responded (response rate, 57%) (Additional file 1). Respondents represented 22 countries across various geographies: Africa (*n* = 50, 49%), North America (*n* = 39, 38%), Europe (*n* = 7, 7%), Oceania (*n* = 6, 6%), and Asia (*n* = 1, 1%). The most common countries represented included: USA (*n* = 21), Malawi (*n* = 15), and Kenya (*n* = 11). Survey respondents self-disclosed their affiliation, which included implementers/clinicians (*n* = 53, 51%), technical agencies/researchers (*n* = 15, 15%), industry (*n* = 5, 5%), Ministry of Health Representation (*n* = 5, 5%), international bodies (*n* = 2, 2%), advocacy agencies (*n* = 2, 2%), and “Other” which included distributors, academics, non-profits / NGOs, international bodies and consultants (*n* = 21, 20%) (Table 3). A total of 69 stakeholders, many of whom participated in the survey, participated at the in-person consensus meeting in November 2019 in South Africa.

**Table 3:** Summary of organisational affiliation for Delphi-like survey respondents

Respondent Categorization and Region	Number of Respondents	Number of Respondents
**Advocacy Organization**	**2**	**2%**
Africa	2	100%
**Implementer / Clinician**	**53**	**51%**
Africa	32	60%
Asia	1	2%
Europe	2	4%
North America	15	28%
Oceania	3	6%
**Industry**	**5**	**5%**
Africa	1	20%
Europe	3	60%
North America	1	20%
**International Body**	**2**	**2%**
Africa	1	50%
Europe	1	50%
**Ministry of Health**	**5**	**5%**
Africa	4	80%
North America	1	20%
**Other**	**21**	**20%**
Africa	8	38%
Europe	4	19%
North America	8	38%
Oceania	1	5%
**Technical Agency / Researcher**	**15**	**15%**
Africa	4	27%
Europe	2	13%
North America	7	47%
Oceania	2	13%
**Funders**	**0**	**0%**

Initially, two rounds of the survey were planned; however, since a 50% consensus for most characteristics was reached after the first-round survey, a second-round survey was not initiated.

**Fig. 1:**
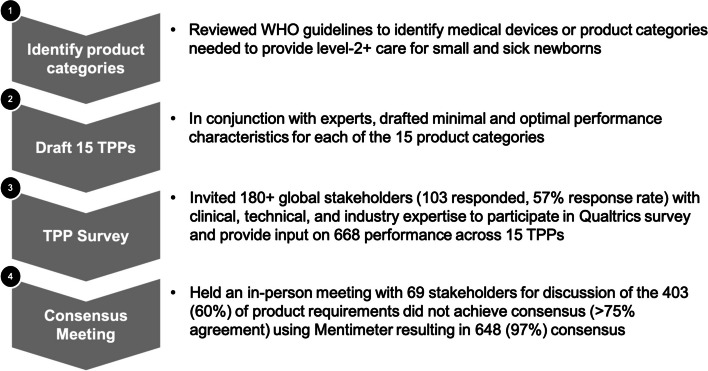
Flow chart summarising TPP process and level of consensus achieved for performance characteristics for final TPPs

Across the 15 TPPs, there were a total of 334 characteristics comprised of 668 optimal and minimal performance characteristics, of which 403 (60%) did not achieve > 75% agreement in the initial survey and were discussed and voted on at the consensus meeting (Fig. 1). Of the 403 characteristics that did not achieve consensus in the TPP survey, 195 characteristics were comprised of overarching characteristics that appeared across all of the TPPs, including Target Operator, Target Population, Target Setting, Quality Management, Regulation, User Manual / Instructions and Warranty. Consensus meeting moderators reviewed over 1,780 comments received in the survey, presented the results and summarised comments from these 403 performance characteristics, solicited additional feedback on each characteristic, and then proposed a change to the TPP characteristic resulting in 97% agreement across the 668 performance characteristics.

Note that the data are the opinions of the participants, which were frequently diverse, despite reaching the 75% threshold determined for consensus. Throughout the consensus meeting, various research questions were identified (Additional file 3 pg. 17–18). For example, the most accessible point-of-care glucometers are designed to be accurate at high glucose ranges for management of adult diabetes; few are intended for use or accurate in the low glucose concentrations seen in hypoglycemic newborns. The group discussed the need to compare and measure the performance of adult glucometers at neonatal-relevant levels vs designing neonate-specific glucometers.

Survey participants were not required to respond to each of the individual product category surveys for the 15 TPPs. Response rates across different product categories ranged from 6 to 47 participants. (Table 4). Of the 15 product categories, 11 had fewer than 25 respondents on the *Qualtrics*™ survey. Point-of-Care Diagnostics, Infection Prevention and Control, and Hydration and Nutrition Pathways of Care were least represented.

**Table 4:** Summary of respondents to TPP survey by product category^1^

Product Category	Number of Respondents
**Respiratory Support**	
Pulse Oximeter (Continuous)	47
CPAP	44
Oxygen Concentrator	30
Flow Splitter	17
Respiratory Rate/Apnea Monitor	15
Suction Pump	12
**Jaundice Management**	
Phototherapy	25
Bilirubinometer	13
**Point-of-Care Diagnostic**	
Glucometer	13
Haemoglobinometer	8
pH Monitor	6
**Thermal Management**	
Temperature Monitor (Continuous)	12
Conductive Warmer	12
Radiant Warmer	6
**Hydration, Nutrition, and Drug Delivery**	
Syringe Pump	10

^a^Overall percentage is not included because survey participants were not required to respond to each of the individual product category surveys for the 15 TPPs

A final set of 15 TPPs, accompanied by a report highlighting the online survey results and consensus meeting discussion, was prepared and published on the UNICEF website [36]. Since its publication in April 2020, this report has been viewed 7,039 times (as of 22 January 2023). Roughly 3,457 users representing 91 countries have viewed the report.

## Discussion

A systematic, four-step process was implemented for the development of 15 TPPs for newborn devices. A consensus-driven approach was utilised to define the product characteristics, and 97% consensus was ultimately achieved across 668 performance characteristics (only 20 performance characteristics in quality management systems, CPAP devices, and pulse oximeter devices did not achieve consensus). Consensus (greater than 75% agreement) was not achieved after two votes on proposed changes due to varying opinions, as briefly described in the results section and in more detail in the full TPP report available in Additional file 3. This disagreement highlights areas where more study may be needed. For example, within the CPAP product category, one of the three areas that did not achieve consensus was related to whether heated humidification was required as a minimal characteristic of a CPAP device. Clinicians noted that heated and humidified air is most important for the smallest newborns weighing less than 1–1.25 kg. Other clinicians responded that the mortality impact has not been explicitly studied in a low-resource setting and that adding this characteristic to the device would increase the cost of equipment, consumables, and maintenance. Consensus was not achieved in this case because further study is needed to explore outcomes and effects with and without heated humidification.

Since publication, the TPP report has been viewed over 7,000 times. The target audience for these 15 TPPs includes both the supply side (innovators, product developers, and manufacturers who develop the technology) and the demand side (healthcare workers, implementers, government workers, and procurement agencies). The development of these TPPs was influential as product developers have adjusted technology development efforts to meet the performance characteristics defined by the TPPs.

On the supply side, product developers reference TPPs at an early stage in the medical device development process. TPPs help inform the ideal characteristics of a medical device and align with the needs of end users. For example, one Swedish manufacturer who works to develop specialised point-of-care tests for newborns, Calmark Sweden AB, referenced the bilirubinometer TPP and adjusted their product to meet the performance characteristics defined by the TPPs. Another company, Neopenda, referenced multiple TPPs, including Pulse Oximeter, Respiratory Rate Monitor, and Temperature Monitor, to develop their 4-in-1 vital signs monitor.

On the demand side, implementers have utilised the TPPs to assist in defining procurement specifications. For example, Palladium International, on behalf of the USAID Integrated Health Program, incorporated components of the TPPs into a tender for the purchase of small/sick newborn training materials [37].

A total of 23 medical devices have been identified by NEST360 as meeting nearly all the performance characteristics across 11 (syringe pump, bilirubinometer, phototherapy, glucometer, haemoglobinometer, CPAP, flow splitter, oxygen concentrator, continuous pulse oximeter, suction pump, radiant warmer) of the 15 TPPs. Over 2,400 of these medical devices have been installed in 65 hospitals in Nigeria, Tanzania, Kenya, and Malawi as part of the NEST360 Alliance [38]. Currently, eight of the 23 NEST360-qualified products are available in the UNICEF Supply Catalogue. While several technologies have been designed to meet the TPPs or are currently under development, unmet technology needs remain. Specifically, there are limited technologies available in diagnostic, monitoring, and thermoregulation product categories (e.g., pH monitor, respiratory rate and apnea monitor, conductive warmer, and continuous temperature monitoring). The need for additional newborn TPPs was identified by participants at the meeting (Additional file 2), as well as for more aspirational TPPs (e.g., resilient oxygen concentrator) to push research and development forward.

In a systematic review conducted by Cocco et al., a common decision-making framework was identified, which consisted of three distinct phases for TPP development: scoping, drafting, and consensus-building [15]. While the methodology outlined above follows this general framework, the approach was novel in scope – applying the TPP process to newborn technologies, breadth – the large number of TPPs developed at once, and reach – the number of countries and types of stakeholders included throughout the process. Establishing a unique methodology to apply the TPP process to medical technologies for newborn care demonstrates the potential to replicate this approach for other widespread and burdensome concerns. For example, this process, which outlined an efficient way of gathering collective experts to drive a whole package of support, could be applied to developing a suite of TPPs for other bundles of care, whether by location at a facility (e.g., another ward at the hospital or the laboratory) or by patient type (e.g., maternal care, or people living with HIV).

The strengths of this work include: (i) it followed a formalised step-wise process established for the development of consensus-based TPPs; (ii) it included a broad group of stakeholders and representative points of view; (iii) it allowed for an open dialogue between end-users and product developers, which captured trade-offs and a consensus building approach. Specifically, this process incorporated a breadth of expertise represented both in the survey and at the consensus meeting. Representation from Ministries of Health, NGOs, and international agencies brought credibility to the process. Furthermore, having representation from the medical device industry and early-stage innovators proved invaluable in facilitating a healthy and productive dialogue. For example, a valuable dialogue ensued for the Linear Range requirement on the Bilirubinometer TPP whereby clinicians noted that the upper end of the range was more important, and product developers noted that from a technical perspective, going above 25 mg/dL was relatively easy up to 30 mg/dL. Similar to other TPP processes, our approach was to include industry in the survey and discussion. But anticipating any potential conflict of interest concerns, the voting percentages were calculated including and excluding industry representation. This allowed us to incorporate a very important perspective from industry in the consensus meeting and resulting TPPs.

However, we acknowledge several limitations. With respect to our methodology, we acknowledge that the TPPs reflect the opinion of stakeholders represented in the online survey and at the consensus meeting and therefore have limited generalisability. Based on a systematic review conducted by Cocco et al., the number of participants invited to the consensus-building meetings varied (< 20 participants: *n* = 5; between 20 and 50 participants: *n* = 7) [15]. While we attempted to have a representative group of stakeholders and had 69 stakeholders in attendance at the consensus meeting, we had varying response rates across different product categories in the initial TPP survey. Also, we had varying response rates across stakeholders’ organisational affiliations and geographies. As noted in Table 3, over 51% of our survey respondents identified themselves as implementers / clinicians. This may be because survey participants had to select one organisational affiliation; thus, the survey may not have captured involvement in other organisations including professional societies and ministries of health. Many of the survey respondents and consensus meeting participants not only provide clinical care to neonates in hospitals in LMIC but also serve / have served in other roles, including but not limited to leaders of paediatric and neonatology associations within LMIC and members of Ministry of Health Newborn Technical Working Groups within LMIC. We also note that implementer / clinician is a broad term, and we did not ask survey respondents or consensus meeting participants to further define their clinical cadre, speciality, and sub-specialty, such as neonatal nurse or neonatologist, or any additional clarifying details about their role. For example, one of the participating neonatologists was the only neonatologist in his country, but details such as this were not part of the survey. Also, a number of survey respondents were recorded as being from North America and not LMIC. This may be because survey participants had to select one geography; thus, the survey may not have captured the geographic work sector of participants originally from North America who also practice in LMIC. The survey and consensus meeting were conducted in English which may exclude manufacturers / product developers from China and participants from French-speaking countries. Second, the TPP process balances being prescriptive with defined characteristics (which might restrict innovation) with being open-ended (which might limit value to innovators seeking guidance on a useful starting point in defining medical device performance). Furthermore, the TPPs only address 15 product categories within the newborn space so certain important types of care (e.g., kangaroo mother care KMC) were not included. During the Consensus Meeting, we asked participants to suggest additional TPPs of interest included in Additional file 3 (pg. 18).

TPP documents as guiding tools also have limitations. A limitation of TPPs more broadly is that they are published at a point in time and therefore, become outdated as new information becomes available. For example, the Oxygen Concentrator TPP was published following our 2019 meeting as of a point-in-time in early 2020. However, as the COVID-19 pandemic impacted the demand for Oxygen Concentrators, new implications arose that impacted the necessary product requirements for low- and middle-income countries. Therefore, the prior TPP was outdated and a new resilient oxygen concentrator TPP was developed by UNICEF to address the concerns identified [39]. A potential solution is to make TPPs “living” documents akin to WHO clinical guidelines, where panels review and solicit feedback from a broader pool of stakeholders bi-annually [40]. Nonetheless, partners have been able to adapt to the ever-changing environment and address new needs as they arise. Finally, TPPs do not measure all qualitative human factors that may hold greater significance than technical specifications in determining device performance. Many of the human factors identified by the systematic review conducted by Cocco et al. were included in the 15 TPPs (e.g., size and portability, training and education, etc.). However, many other qualitative human factors are more subjective in nature and difficult to define in a TPP. Yet, the ultimate commercial success of these technologies in LMIC may depend on factors including human resource constraints, access to consumables, and other qualitative characteristics. To mitigate this, NEST360 devised a qualification process to evaluate commercially available medical devices that meet the TPPs. This evidence-based process includes product landscaping, rigorous laboratory and environmental testing, usability assessment by target users, and device performance monitoring in newborn wards across Kenya, Malawi, Nigeria, and Tanzania [38]. The NEST360 Qualification Process can help designers recognise and adapt to common challenges faced in resource-limited settings, ultimately leading to superior clinical outcomes.

Evaluation and validation of technologies in the target settings of varying geographies is a key area for research. Additional research questions were identified at the consensus meeting that may inform future product development and guidelines. These research questions include optimising the storage and stacking of equipment to better account for infrastructure constraints and reduce the challenges associated with the bulk weight and footprint of certain medical instruments, along with exploring the impact of reusable consumables and the potential to develop guidelines on decontamination and reprocessing of single-use devices. Additionally, the team also identified the need to develop methods to safely provide adequate levels of heated humidification for CPAP recipients in low-resource settings (specifically for babies less than 1.25 kg). A standardised methodology for measuring the time required to indicate the accurate temperature of a baby and verifying consistency in temperature across the surface area for warmers is also required.

Additionally, testing whether adult glucometers are accurate at the low glucose concentrations seen in hypoglycaemic newborns could increase access without requiring new product development. Another area of interest is to define international standards for respiratory rate accuracy and the ethical challenges that exist in standardising experimental conditions to define a gold standard (i.e., using humans as a 'reasonable reference standard'). Moreover, the team also identified the need to review existing literature on power cuts to determine how long the power supply should last.

## Conclusion

The need for effective, affordable, and user-friendly technologies in LMIC is critical to achieving the ENAP coverage targets of 80% of districts having at least one functional WHO level-2 inpatient unit to care for small and sick newborns [10]. While the development of TPPs generally has proven helpful in the diagnostics and pharmaceutical development space, this approach was novel in three ways: (i) scope – applying the TPP process to newborn technologies, (ii) breadth – the large number of TPPs developed at once, and (iii) reach – the number of countries and types of stakeholders included throughout the process. Establishing a unique methodology to apply the TPP process to medical technologies for newborn care demonstrates the potential to replicate this approach for other widespread and burdensome concerns. In our exercise, 15 newborn care TPPs were developed (which have been viewed on the UNICEF website over 7,000 times since publication), and the process helped facilitate dialogue between the supply (manufacturer and product developer) and demand (healthcare user) sides. Given our learning, there is potential to adapt and apply the TPP development process to other fields beyond newborn care.

## Supplementary Information

**Additional file 1.** Summary of response rate by country for all Delphi-like survey responses. Includes a breakdown of the number of participants and the overall percentage by geography and country for the TPP survey.

**Additional file 2.** Additional newborn TPPs proposed by consensus meeting participants. Includes a list of additional proposed TPPs in the newborn space for future consideration and development.

**Additional file 3.** Target Product Profiles for newborn care and consensus meeting report. Full 233-page report included on the UNICEF website, which features all 15 of the final TPPs and a summary of the consensus meeting discussion.

**Additional file 4.** Target Product Profile Qualtrics survey. Qualtrics survey for all of the TPPs.

## Data Availability

The TPP results are available open access on the UNICEF website: https://www.unicef.org/supply/documents/target-product-profile-newborn-care.

## Abbreviations

CPAP: Continuous Positive Airway Pressure
ENAP: Every Newborn Action Plan
FDA: Food and Drug Administration
kg: Kilograms
KMC: Kangaroo mother care
LMIC: Low- and middle-income countries
NEST360: Newborn Essential Solutions and Technologies
NGOs: Non-governmental Organisations
QMS: Quality Management Systems
TPP: Target Product Profile
UNICEF: United Nations Children’s Fund
WHO: World Health Organization
