# Supplementary material for: Target product profiles for neonatal care devices: systematic development and outcomes with NEST360 and UNICEF
Source: BMC Pediatr. 2023 Nov 15;23(Suppl 2):564. doi: 10.1186/s12887-023-04342-1 (PMC10647088; doi:10.1186/s12887-023-04342-1)
Supplement: Supplementary file 1 — Additional file 1. Summary of response rate by country for all Delphi-like survey responses. Includes a breakdown of the number of participants and the overall percentage by geography and country for the TPP survey. [file 12887_2023_4342_MOESM1_ESM.pdf]

ADDITIONAL FILES

Additional file 1: Summary of response rate by country for all Delphi-like survey responses

**Country (n = Number of Respondents)**	**Percentage**
**Africa (50)**	**49%**
Malawi (14)	14%
Kenya (10)	10%
Nigeria (7)	7%
Tanzania (6)	6%
Ethiopia (4)	4%
Botswana (2)	2%
Rwanda (2)	2%
Ghana (1)	1%
Mozambique (1)	1%
Senegal (1)	1%
South Africa (1)	1%
Uganda (1)	1%
**North America (39)**	**38%**
USA (22)	21%
Canada (10)	10%
UK (6)	6%
Mexico (17	1%
**Europe (7)**	**7%**
Denmark (2)	1%
France (2)	2%
Switzerland (2)	2%
Italy (1)	1%
**Oceania (6)**	**6%**
Australia (6)	6%
**Asia (1)**	**1%**
India (1)	1%
