# Supplementary material for: Target product profiles for neonatal care devices: systematic development and outcomes with NEST360 and UNICEF
Source: BMC Pediatr. 2023 Nov 15;23(Suppl 2):564. doi: 10.1186/s12887-023-04342-1 (PMC10647088; doi:10.1186/s12887-023-04342-1)
Supplement: Supplementary file 2 — Additional file 2. Additional newborn TPPs proposed by consensus meeting participants. Includes a list of additional proposed TPPs in the newborn space for future consideration and development. [file 12887_2023_4342_MOESM2_ESM.pdf]

ADDITIONAL FILES

Additional file 2: Additional newborn TPPs proposed by consensus meeting participants

**Hydration, Nutrition and Drug Delivery**
o Breast milk pump
o Lactation support tools (e.g., storage bags)
o Total parenteral nutrition (TPN)
o Milk banking
**Jaundice Management**
o ROP screening and treatment
o Retinopathy camera (e.g., RetCam)
**Point-of-Care Diagnostics**
o C-reactive protein (CRP) point-of-care test
**Respiratory Support**
o Mechanical ventilator
o Oxygen blender
o Bedside pulmonary function testing
o Newborn resuscitation device
o Electrocardiogram (ECG)
**Thermal Management**
o Incubator
o Cooling mattresses for therapeutic hypothermia
o Infrared / Spot Check Thermometer (Temperature test)
o Non-electric infant warmers (e.g., Phase Change Material)
**Other**
o Multi-parameter monitoring
o Advanced hemodynamic monitoring
o Transport
§ Oxygen delivery during transport
§ Transporter
§ Transport incubator
§ Infusion pump
§ Portable ultrasound
o Cranial ultrasound
o Backup power package
o Maintenance package
