# Supplementary material for: Target product profiles for neonatal care devices: systematic development and outcomes with NEST360 and UNICEF
Source: BMC Pediatr. 2023 Nov 15;23(Suppl 2):564. doi: 10.1186/s12887-023-04342-1 (PMC10647088; doi:10.1186/s12887-023-04342-1)
Supplement: Supplementary file 3 — Additional file 3. Target Product Profiles for newborn care and consensus meeting report. Full 233-page report included on the UNICEF website, which features all 15 of the final TPPs and a summary of the consensus meeting discussion. [file 12887_2023_4342_MOESM3_ESM.pdf]

unicef for every child

NEST360°

Target Product Profiles

FOR NEWBORN CARE IN LOW-RESOURCE SETTINGS

CONSENSUS MEETING REPORT, MARCH 2020

v1.2

Target Product Profiles for Newborn Care in Low-Resource Settings (v1.2)

Consensus Meeting Report (March 2020)

Acknowledgements
This report was prepared by Rebecca Kirby and Kara Palamountain from Northwestern University with input from UNICEF and other stakeholders. The document summarizes consensus achieved at a meeting on target product profiles for newborn care in low-resource settings, convened by NEST360°. This document was finalized following consideration of all comments and suggestions made by meeting participants at the Consensus Meeting.

NEST360° is made possible by generous commitments from the John D. and Catherine T. MacArthur Foundation, the Bill & Melinda Gates Foundation, The ELMA Foundation, the Children's Investment Fund Foundation, The Lemelson Foundation, the Ting Tsung and Wei Fong Chao Foundation and individual donors to Rice 360°.

Note to the reader
Because of the richness of the discussion, and in an attempt to keep this report simple and readable, this report aims to convey the themes addressed in each session, rather than attempting to provide a chronological summary of the dialogue.

Disclaimer: The TPPs do not replace or supersede any existing UNICEF TPPs. The TPPs do not constitute tender specifications, nor is UNICEF bound to tender or procure products that arise as a result of these TPPs. UNICEF may require regulatory approval and proof of compliance to quality management and product-specific international standards for tendering purposes.

Table of Contents

TABLE OF CONTENTS **3**

INTRODUCTION **6**
BACKGROUND 6
DEVELOPING TARGET PRODUCT PROFILES 7
DELPHI-LIKE SURVEY PROCESS 8
CONSENSUS MEETING 10
OVERARCHING CHARACTERISTICS 11
BROAD THEMES AND CONSIDERATIONS 15
RESEARCH QUESTIONS 17
FUTURE NEONATAL TPPS 18

FINAL TARGET PRODUCT PROFILES **19**

HYDRATION, NUTRITION, AND DRUG DELIVERY **20**
SYRINGE PUMP 20
Introduction: Syringe Pump *20*
Final TPP: Syringe Pump *21*
Consensus Meeting Summary: Syringe Pump *22*
Delphi-like Survey: Syringe Pump *25*

JAUNDICE MANAGEMENT **35**
BILIRUBINOMETER 36
Introduction: Bilirubinometer *36*
Final TPP: Bilirubinometer *36*
Consensus Meeting Summary: Bilirubinometer *38*
Delphi-like Survey: Bilirubinometer *39*
PHOTOTHERAPY LIGHT 47
Introduction – Phototherapy Light *47*
Final TPP - Phototherapy Light *47*

Consensus Meeting Summary: Phototherapy Light *49*
Delphi-like Survey: Phototherapy Light *50*

POINT-OF-CARE DIAGNOSTICS **57**
GLUCOMETER 58
Introduction - Glucometer *58*
Final TPP - Glucometer *58*
Consensus Meeting Summary: Glucometer *60*
Delphi-like Survey: Glucometer *61*
HEMOGLOBINOMETER 68
Introduction: Hemoglobinometer *68*
Final TPP: Hemoglobinometer *68*
Consensus Meeting Summary: Hemoglobinometer *70*
Delphi-like Survey: Hemoglobinometer *70*
PH MONITOR 76
Introduction: pH Monitor *76*
Final TPP: pH Monitor *76*
Consensus Meeting Summary: PH Monitor *78*
Delphi-like Survey: pH Monitor *78*

INFECTION PREVENTION AND CONTROL **83**
SEPSIS DIAGNOSTIC 84
Introduction: Sepsis Diagnostic *84*
Consensus Meeting Summary: Sepsis Diagnostic *84*
Use Case Survey: Sepsis Diagnostic *86*

RESPIRATORY SUPPORT **93**
CPAP 94
Introduction: CPAP *94*
Final TPP: CPAP *95*
Consensus Meeting Summary: CPAP *96*
Delphi-like Survey: CPAP *98*
FLOW SPLITTER 107
Introduction: Flow Splitter *107*
Final TPP: Flow Splitter *107*
Consensus Meeting Summary: Flow Splitter *108*
Delphi-like Survey: Flow Splitter *109*
OXYGEN CONCENTRATOR 113
Introduction: Oxygen Concentrator *113*

Final TPP: Oxygen Concentrator *113*
Consensus Meeting Summary: Oxygen Concentrator *116*
Delphi-like Survey: Oxygen Concentrator *122*
PULSE OXIMETER (CONTINUOUS) 138
Introduction: Pulse Oximeter (Continuous) *138*
Final TPP: Pulse Oximeter (Continuous) *138*
Consensus Meeting Summary: Pulse Oximeter (Continuous) *140*
Delphi-like Survey: Pulse Oximeter *145*
RESPIRATORY RATE / APNEA MONITOR 161
Introduction: Respiratory Rate / Apnea Monitor *161*
Final TPP: Respiratory Rate / Apnea Monitor *161*
Consensus Meeting Summary: Respiratory Rate / Apnea Monitor *163*
Delphi-like Survey: Respiratory Rate / Apnea Monitor *166*
SUCTION PUMP 176
Introduction: Suction Pump *176*
Final TPP: Suction Pump *176*
Consensus Meeting Summary: Suction Pump *177*
Delphi-like Survey: Suction Pump *178*

THERMAL MANAGEMENT 184
RADIANT WARMERS 186
Introduction: Radiant Warmer *186*
Final TPP: Radiant Warmer *186*
Consensus Meeting Summary: Radiant Warmer *188*
Delphi-like Survey: Radiant Warmer *190*
TEMPERATURE MONITOR (CONTINUOUS) 199
Introduction: Temperature Monitor (Continuous) *199*
Final TPP: Temperature Monitor (Continuous) *199*
Consensus Meeting Summary: Temperature Monitor (Continuous) *201*
Delphi-like Survey: Temperature Monitor *202*
CONDUCTIVE WARMER 210
Introduction: Conductive Warmer *210*
Final TPP: Conductive Warmer *211*
Consensus Meeting Summary: Conductive Warmer *213*
Delphi-like Survey: Warming Crib *214*

APPENDIX A: DELPHI-LIKE SURVEY RESPONDENT ORGANIZATIONAL DESIGNATION 223

APPENDIX B: CONSENSUS MEETING PARTICIPATION 224
APPENDIX C: ABBREVIATIONS 225
REFERENCES 226

INTRODUCTION

BACKGROUND

Globally, 2.5 million children die in the first month of life and more than half of these deaths are due to conditions that could be prevented or treated with access to simple, affordable interventions [1].

The first 28 days of life – the neonatal period – represent the most vulnerable time for a child's survival. Globally, more children than ever before are being born in facilities and there are well-described, low-cost, evidence-based practices to address neonatal mortality. However, three quarters of neonatal deaths (nearly 2 million) happen in the first week of life when a child is still at or near a health facility [2]. Health interventions are needed that can provide comprehensive neonatal care at facilities to address the major causes of neonatal deaths. Many of these health interventions are known and can be cost-effective. These interventions though may be different from other interventions needed to address broader under-5 deaths [3].

For the first time ever in 2015 the world pledged to end preventable newborn deaths by 2030 (Sustainable Development Goal 3.2) [4]. On current trends, more than 60 countries will miss the Sustainable Development Goal (SDG) target of reducing neonatal mortality to at or below 12 deaths per 1,000 live births by 2030. About half will still not reach the target by 2050. These countries carry about 80 per cent of the burden of neonatal deaths in 2016 [3]. Focused efforts to strengthen the ability of health systems to deliver neonatal care are still needed in sub-Saharan Africa and South East Asia so as to prevent 80 per cent of these deaths [1].

To address neonatal mortality, the World Health Organization (WHO) is working with Ministries of Health and partners to expand quality services for sick and small newborns in the first week of life [5]. Critical to the sustainable implementation of quality facility-based services will be equipping not only people, but facilities with neonatal equipment that is high quality, affordable, robust, and appropriate for comprehensive care delivery in low-resource settings.

Globally, the largest contributors to neonatal mortality are preterm birth, intrapartum complications, and infection. Many deaths attributable to these causes are preventable through six categories of care:

1. HYDRATION, NUTRITION, AND DRUG DELIVERY
2. JAUNDICE MANAGEMENT
3. POINT-OF-CARE DIAGNOSTICS
4. INFECTION PREVENTION AND CONTROL
5. RESPIRATORY SUPPORT
6. THERMAL MANAGEMENT

Most neonatal healthcare technologies that support these pathways of care are designed for high-resource settings and are either unavailable or unsuitable for use in low-resource settings. As a result, providers in low-resource settings lack the tools needed to deliver quality, comprehensive, newborn care.

There is an urgent need for neonatal healthcare technologies that are affordable, rugged, effective, simple to use and maintain, and able to operate from various power supplies.

DEVELOPING TARGET PRODUCT PROFILES

Manufacturers need Target Product Profiles (TPPs) at an early stage in the medical device and diagnostic development process. These TPPs help inform the ideal targets and specifications and align with the needs of end users. TPPs outline the most important performance and operational characteristics as well as pricing. In the TPPs to follow, the term "Minimal" is used to refer to the lowest acceptable output for a characteristic and "Optimal" is used to refer to the ideal target for a characteristic. The Optimal and Minimal characteristics define a range. Products should meet at least all of the Minimal characteristics and preferably as many of the Optimal characteristics as possible. TPPs should also specify the goal to be met (e.g. to initiate treatment), the target population, the level of implementation in the healthcare system and the intended end users.

An initial set of TPPs were developed listing a proposed set of performance and operational characteristics for 16 product categories. The development timeline envisioned in the TPPs was four years, although some commercially available technologies may fit some of the criteria already. For several of the characteristics, only limited evidence was available and further expert advice was sought from additional stakeholders.

DELPHI-LIKE SURVEY PROCESS

To obtain this expert advice and to further develop the TPPs, a Delphi-like process was used to facilitate consensus building among stakeholders. The initial TPPs were sent to a more comprehensive set of stakeholders including clinicians, implementers, representatives from Ministry of Health, advocacy organizations, international agencies, academic and technical researchers and members of industry. In total, 103 stakeholders from 22 countries participated in the TPP development process via survey.

The number of Delphi-like survey respondents is included next to each product category.

- Pulse Oximeter – 47 respondents
- CPAP (formerly titled Bubble CPAP)* – 44 respondents
- Sepsis Diagnostic (formerly titled Sepsis Test)* – 33 respondents *Note: For this product category, a Use Case survey (vs. a TPP) was utilized*
- Oxygen Concentrator – 30 respondents
- Phototherapy Light – 25 respondents
- Flow Splitter – 17 respondents
- Radiant Warmer – 17 respondents
- Respiratory Rate / Apnea Monitor (formerly titled Respiratory Rate Monitor)* – 15 respondents
- Glucometer (formerly titled Glucose Test)* – 13 respondents
- Bilirubinometer (formerly titled Serum Bilirubin Test)* – 13 respondents
- Suction Pump – 12 respondents
- Temperature Monitor – 12 respondents
- Conductive Warmer (formerly titled Warming Crib)* – 12 respondents
- Syringe Pump – 10 respondents
- Hemoglobinometer (formerly titled Hemoglobin Test)* – 8 respondents
- pH Monitor (formerly titled pH Test)* – 6 respondents

NOTE: Based upon discussion and review throughout the development of these TPPs, the names of the product categories designated above with a * were modified from the time that the original survey was sent. The title of the product category originally included in the Delphi-like survey is included in parenthesis for reference.

Survey respondents were requested to provide a statement on their level of agreement with each of the proposed characteristics for each TPP. Agreement was scored on a Likert scale ranging from 1 to 5 (1=disagree, 2=mostly disagree, 3= neither agree nor disagree, 4=mostly agree, 5=fully agree) with an option to opt out with the selection of "Other - Do not have the expertise to comment". If participants did not agree with the characteristic (i.e., selected 3 or below) they were asked to provide an explanation with comments. Participants who agreed with the statements could also provide comments however were not explicitly asked. In total, over 1,780 comments were reviewed and summarized in this report.

For each characteristic in each product category, a percentage agreement was calculated for both the Minimal and Optimal requirements. The percentage agreement was calculated as the ratio of the sum of number of respondents who selected 4 and 5, to the sum of numbers of respondents who gave any score (from 1 to 5 where 5=fully agree, 4=mostly agree, 3=neither agree nor disagree, 2=mostly disagree and 1=disagree). Consensus for the survey characteristics was pre-specified at greater than 50% of respondents providing a score of at least 4 on the Likert scale.

A classic Delphi process requires at least two rounds of survey ahead of an in-person meeting. Initially, two rounds of the survey were planned, but since 50% consensus for most characteristics was reached after the first round survey, a second round survey was not initiated. Survey results are detailed by characteristic in the individual product category sections.

In total, over 180 organizations/individuals were asked to participate in this Delphi-like survey process, of whom 103 (see Appendix A) responded (response rate, 56%). Survey respondents were asked to self-disclose their affiliation. In summary, about half of responders were implementers/clinicians, 15% were from technical agencies/researchers, 5% industry, 5% Ministry of Health Representation, 2% international bodies, 2% advocacy agencies, and the remaining 22% were "Other" which includes distributors, academics, non-profits / NGOs, international bodies and consultants (see summary in Figure 1 below). A breakdown of participation by product category is included in the individual product category sections.

Figure 1: Summary of organizational affiliation of all Delphi-like survey responses

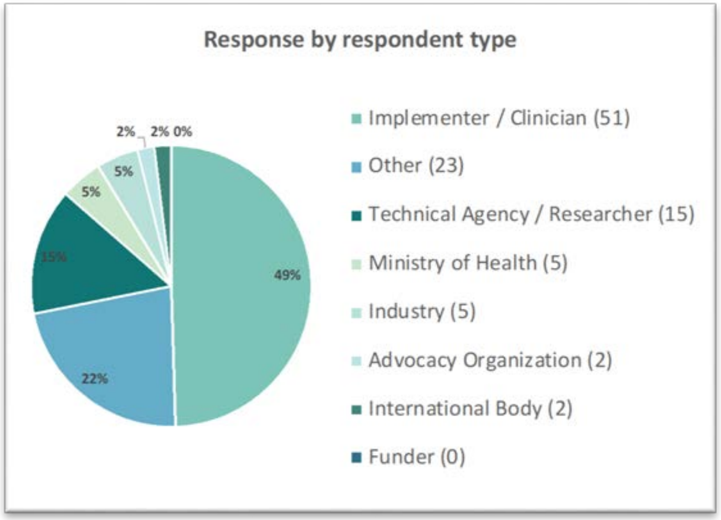

Respondent type	Percentage
Implementer / Clinician (51)	49%
Other (23)	22%
Technical Agency / Researcher (15)	15%
Ministry of Health (5)	5%
Industry (5)	5%
Advocacy Organization (2)	2%
International Body (2)	2%
Funder (0)	0%

Figure 2: Summary of response rate by country for all Delphi-like survey responses

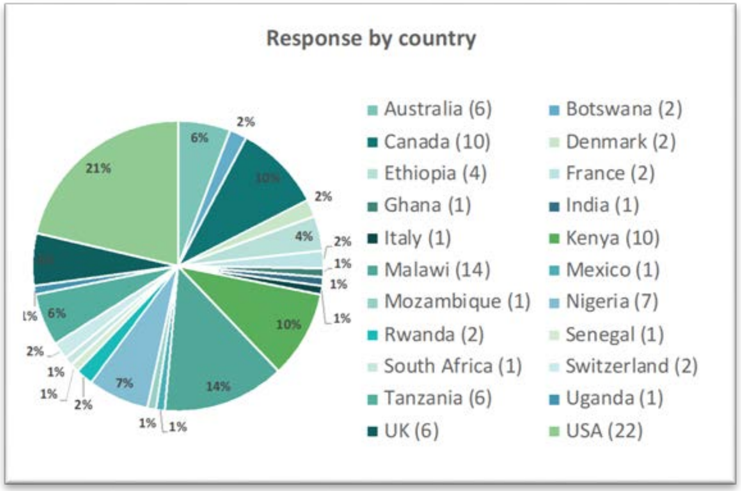

Country	Percentage
USA (22)	21%
Malawi (14)	14%
Canada (10)	10%
Kenya (10)	10%
Nigeria (7)	7%
Australia (6)	6%
Tanzania (6)	6%
UK (6)	6%
Ethiopia (4)	4%
Botswana (2)	2%
Denmark (2)	2%
France (2)	2%
Rwanda (2)	2%
Switzerland (2)	2%
Ghana (1)	1%
India (1)	1%
Italy (1)	1%
Mexico (1)	1%
Mozambique (1)	1%
Senegal (1)	1%
South Africa (1)	1%
Uganda (1)	1%

CONSENSUS MEETING

On November 20 - 22, 2019 over 69 stakeholders gathered in Stellenbosch, South Africa to focus on building further consensus on areas of discrepancy in opinion within the 16 TPPs. More specifically, characteristics on which fewer than 75% of the respondents agreed, or on which a distinct subgroup disagreed, were discussed. Consensus Meeting moderators presented the results and comments from characteristics with <75% agreement from the Delphi-like survey, the moderators then solicited additional feedback on each characteristic with <75% agreement from the Consensus Meeting participants, and then a proposed change to the TPP characteristic was discussed amongst Consensus Meeting participants. In some cases, Consensus Meeting participants nearly universally agreed on proposed changes. In other cases, Consensus Meeting participants failed to reach 75% consensus on proposed changes. If consensus was not achieved after two votes on proposed changes, meeting participants agreed to move forward and the disagreement is noted in this report.

Methodology for Mentimeter Voting Results: Certain proposed changes to TPP characteristics, for which a distinct subgroup disagreed, were anonymously voted on using Mentimeter.com to determine the overall level of agreement and disagreement amongst the Consensus Meeting participants. The Mentimeter Voting Results are presented throughout this report in three distinct categories:

I. Overall vote – Includes all Consensus Meeting participants who voted on Mentimeter.com. To eliminate the possibility of duplicate votes, all respondents were asked to enter their name (to be viewed only by the report authors) and blank (potentially duplicate votes) were eliminated from the overall vote.
II. Clinicians – Includes all Consensus Meeting participants who voted on Mentimeter.com and who designated themselves as a Clinician on Mentimeter.com.
III. Excluding involvement with product development - Includes all Consensus Meeting participants who voted on Mentimeter.com minus those who indicated on a Declaration of Interest form that they are 'currently or have been involved in the development of a candidate technology or product' specific to the Product Category being voted on.

Of the 133 stakeholders that were invited to the meeting, 69 participants were able to attend. Participants comprised country representatives, stakeholders from technical and funding agencies, researchers, implementers and civil society organizations, and representatives from companies working on newborn care technologies (see Appendix B for the Consensus Meeting Participant List). An overview of the discussion and final consensus achieved is incorporated throughout the sections to follow.

Most characteristics discussed are presented in this report within the individual product categories, however, a few overarching characteristics that applied to all product categories were discussed in unison and are presented together.

OVERARCHING CHARACTERISTICS

The following summarizes the discussion at the Consensus Meeting for the overarching characteristics that appeared in all TPPs.

Target Operator

There was agreement in the room that the Target Operator characteristic for all product categories would be for use in low- and middle-income countries by a wide variety of clinicians, including nurses, clinical officers, and pediatricians.

Optimal: For use in low- and middle-income countries by a wide variety of clinicians, including nurses, clinical officers, and pediatricians
Minimal: Same as Optimal

Original Optimal and Minimal: For use in low- and middle-income countries by a wide variety of clinicians, including nurses, clinical officers, and pediatricians.

Target Population

During the discussion, some participants proposed expanding the Target Population characteristic to a wider population beyond neonates. Others, including product developers and clinicians, felt that it was important to specify the patient population by body weight, rather than age (i.e., up to 5 kg) since the gestational age was often difficult to measure. Consensus was achieved in the room for the Optimal characteristic that the product must be useful and effective and validated for neonatal period.

Optimal: Neonates (born at any gestational age and require ongoing care)
Minimal: Same as Optimal

Original Optimal and Minimal: Neonates (<28 days)

Target Setting
Some participants felt strongly that the Target Setting characteristic should be broadened from "hospitals in low-resource settings" to optimally include "primary care health facilities". Some participants challenged this and noted that personnel in some primary facilities may not have the proper training or resources available. One participant noted that the small and sick babies in most need would likely be transferred to a higher-level referral hospital rather than being treated in the primary health facility. On November 20, a vote was conducted with the results below:

Optimal: Health facilities in low-resource settings
- *Overall Vote: 93% Agree (n = 44)*
- *Clinicians: 94% Agree (n = 32)*
- *Excluding involvement with product development: 94% Agree (n = 32)*

Minimal: Hospitals in low-resource settings
- *Overall Vote: 93% Agree (n = 46)*
- *Clinicians: 94% Agree (n = 32)*
- *Excluding involvement with product development: 94% Agree (n = 34)*

The discussion reconvened on Friday, November 22 as some participants expressed concern about the proposed expansion to expand the optimal Target Setting to encompass all health facilities. The main concern expressed was that certain product categories could be used incorrectly in health facilities without proper staff, training, or infrastructure. Additionally, some participants questioned how verification of technologies would be conducted in health facilities given the wide range staff and infrastructure conditions. Participants also noted that the recently published WHO-UNICEF Technical Specifications and Guidance for Oxygen Therapy Devices [6, p. 10-12], provides a general guide for how Oxygen Therapy devices (e.g., oxygen concentrators, flow splitters, CPAP, pulse oximeters, etc.) may be integrated within different levels of the health system. Consensus was achieved that for Glucometer, Flow Splitter, Oxygen Concentrator, Pulse Oximeter, Respiratory Rate Monitor, Suction Pump, and Temperature Monitor the Optimal *Target Setting* characteristic could include health facilities, however, for the remaining products, both the Optimal and the Minimal characteristic would be for use in hospitals in low-resource settings.

For product categories: Syringe Pump, Phototherapy Light & Meter, Bilirubinometer, Hemoglobinometer, pH Monitor, CPAP, Radiant Warmer, and Conductive Warmer
Optimal: Hospitals in low-resource settings
Minimal: Hospitals in low-resource settings

For product categories: Glucometer, Flow Splitter, Oxygen Concentrator, Pulse Oximeter, Respiratory Rate Monitor, Suction Pump, and Temperature Monitor
Optimal: Hospitals in low-resource settings, but may be used in health facilities based on country guidelines
Minimal: Hospitals in low-resource settings

Original Optimal and Minimal: Hospitals in low-resource settings

Quality Management (previously titled 'International standard')
Participants voiced universal support that technologies should be manufactured in a quality system even if a specific ISO standard for the device does not yet exist. Given the wide discrepancy in quality management systems, in-country clinicians and procurement agencies and suppliers felt strongly that adherence to international standards, was important. Furthermore, participants noted that compliance with ISO certification is difficult to measure and therefore diminishes the weight of its importance. Product developers explained that requiring the minimum to meet ISO certification could impact the price of the product and potentially limits the approach towards innovation. Some participants emphasized how this characteristic was closely related to the Regulation characteristic requirements. One participant noted that ISO 13485 certification is compulsory for obtaining a CE mark.

On November 20, a vote was conducted with the results below:

Minimal: Same as Optimal - ISO 13485:2016 Medical devices – Quality management systems - Requirements for regulatory purposes [80]

- *Overall Vote: 62% Agree (n = 29)*
- *Clinicians: 56% Agree (n = 18)*
- *Excluding involvement with product development: 55% Agree (n = 20)*

For Quality Management, it was agreed to proceed with the following requirements for both the Optimal and Minimal characteristic even though 75% consensus was not achieved: ISO 13485:2016 Medical devices – Quality management systems - Requirements for regulatory purposes [80].

On November 22, the group reconvened the discussion on the characteristic Regulation (see below).

Original Optimal and Minimal (characteristic previously titled 'International Standard'): ISO 13485:2016 Medical devices – Quality management systems – Requirements for regulatory purposes [80].

Regulation
There was an extensive discussion on regulatory requirements on November 20 which continued November 22. Some participants emphasized how this characteristic was closely related to the Quality Management characteristic requirements.

Product developers emphasized that gaining a CE mark is not indicative of "ability to sell your product" and is often costly and time-consuming which can be restrictive to early stage developers and potentially stifle future innovation. Product developers face many challenges in securing regulatory approval such as the lengthy time cycles, ability to access capital, and access to regulatory experts / consultants. Some participants noted that regulatory approval or CE marking does not necessarily translate to good performance. Participants responded that it is still important for manufacturers to "do the right thing with regard to performance testing" and encouraged more transparency. Additionally, there is a great disconnect in post-market surveillance assistance making it difficult for developers to continue collecting data on follow-up user studies to ensure technologies are delivering as promised. The group determined that there is a great need to further support innovators in these settings.

Another theme emerged highlighting the complexities of regulatory certification and the multitude of options available which can, at times, lead to confusion. The group agreed that there is an opportunity to better harmonize the medical device certification process and acknowledged that the CE mark certification process continues to evolve. In alignment with the theme expressed that regulatory approval does not necessarily translate to good performance, international NGOs explained that they are using their "buying power" to push for greater transparency, especially with public good documents that inform the buyer. However, international agencies and NGOs emphasized the importance of quality control for medical devices and that "we need to hold products in as high-standing as possible". Discussion also ensued on the strength of local regulatory bodies and the importance of local clinical efficacy trials in addition to CE mark to ensure local buy-in.

Some in-country clinicians and international agencies felt that "CE mark only" was enough, while others voiced support for "clearance from at least one stringent regulatory body". In-country clinicians and distributors emphasized that country ministries will often look to "Big 5" for guidance when making purchasing decisions. International NGOs emphasized that manufacturers expanding in international markets will generally secure regulatory approval with broad application. Additionally, international NGOs noted "you will be hard-pressed finding a donor who will procure without approval from a Stringent Regulatory Authority (SRA)".

Optimal: At least one of: CE marking, approved by US FDA or another stringent regulatory body of a founding member of IMDRF (e.g., Japan or Australia or Canada)
Minimal: Same as Optimal.

Original Optimal and Minimal: CE marking or US FDA Clearance

User Manual / Instructions
The group agreed that the primary intent was that a user could read the manual, but there were various opinions on whether the Optimal should include translation into all the relevant UN official languages and at least one national language for the country of intended use. Participants noted that if too many translations were included, this could cause unintended consequences that the manual might become too bulky and burdensome and ultimately not be used.

Optimal: User manual and additional training materials (checklists, videos, guides) in at least one national official language for the country of intended use. Attached to device with labels and markings where possible.
Minimal: User manual provided in at least one national official language.

Original Optimal: User manual and additional training materials (checklists, videos, guides) in English and local language. Attached to device with labels and markings where possible.
Original Minimal: User manual provided.

Warranty
While most agreed that a 5 year Warranty Period was Optimal, some felt this was not a realistic target. Participants noted that for many medical devices, the WHO-UNICEF Technical Specifications and Guidance for Oxygen Therapy Devices [6, p. 86-145] often require at least 1 year warranty, with 2 to 5 years being the recommendation, depending on the device. One suggestion was to include uptime hours in the warranty language, rather than a period of time. Consensus was achieved on the Optimal characteristic (5 year warranty) via agreement in the room and the Minimal characteristic (1 year warranty) via a vote.

On November 20, a vote was conducted with the results below:

Optimal: 5 years
Minimal: 1 year
- *Overall Vote: 91% agreement (n = 35)*
- *Clinicians: 87% Agree (n = 23)*
- *Excluding involvement with product development: 89% Agree (n = 27)*

Original Optimal: 5 years
Original Minimal: 1 year

BROAD THEMES AND CONSIDERATIONS

The following summarizes additional themes that emerged from the Consensus Meeting.

Instrument Pricing
In order to provide a consistent measure of pricing, the ex-works price is included in the TPPs. Participants highlighted that ex-works pricing is not a true measure of landed cost and is often vastly understated to what a procurement agent will pay. One participant from an international NGO noted that there is a "minimum 30% mark-up on the ex-works price." The rationale for using the ex-works price is that it is a reliable measure that can be used for consistent comparison across geographies since distributor markups vary by country and geography.

Utility Requirements

A significant portion of the discussion was devoted to deliberating on how equipment can be designed to work in health facilities with limited electrical infrastructure. Designing the equipment for low-resource conditions often requires back-up batteries which adds to the expense of the technology, as well as the size of the equipment which can pose a challenge in crowded newborn wards. Some participants noted that rather than designing equipment for these facilities with limited electrical infrastructure, to consider whether a broader investment in electrical infrastructure would be a better use of funds. This inherent tradeoff was discussed multiple times when electrical characteristics were discussed.

Additionally, there were a variety of characteristics in the initial survey that related to Utility Requirements (i.e., electricity and power) that varied slightly in title across the TPPs. During the TPP Consensus Meeting, participants agreed that all characteristics relating to Utility Requirements (includes Back-up Battery; Battery Power; Batteries; Voltage; Power Requirement; Maximum Power Consumption; Response During Power Outage; Surge Protection, Electrical Plug) be reviewed and harmonized following the TPP meeting across the product categories. These characteristics have since been reviewed and harmonized into four distinct characteristics (Power Source, Battery, Voltage, and Power Consumption) in the final TPPs.

- **Power Source** - This defines the desired power source for the device and can be broken down into the following categories:
    - *Mains power* - device must be plugged into a mains power source for use
    - *Mains with battery backup* - device must be plugged into a mains power source for use, however, in the case of a power failure, the device has a battery backup that can last a specified period of time
    - *Mains with rechargeable battery* - device has a rechargeable battery that operates both when the device is charged by a mains power source, or, when the device is plugged in (e.g., a mobile phone)
    - *Battery is disposable and replaceable*
    - *No power required (i.e., disposable device)*
- **Battery** - This includes the length of time the rechargeable or disposable battery should function
- **Voltage** - This specifies the preferred voltage conversion if the Power Source utilizes Mains Power. Note that for certain technologies (i.e., Bilirubinometer, Glucometer, Hemoglobinometer, pH monitor, and Pulse Oximeter), the Voltage characteristic is included in reference to the rechargeable battery charger requirements. For example, while the Optimal Voltage characteristic is "None" (i.e., no charging is necessary), the Minimal Voltage characteristic should conform to “the voltage and frequency of the purchasing country’s local power grid (e.g., 110-120 VAC at 60 Hz or 220-240 VAC at 50 Hz)" to ensure that the charger for the battery is compliant.
- **Power Consumption** - This specifies the maximum Watts of electricity that the device should consume

Ideally, all devices should be developed to withstand power surges and voltage spikes.

Note that comments received in the Pre-Meeting survey report highlighted the importance of the correct frequency in electrical plugs. It was noted that a universal adaptor would not safely support the conversion of 60Hz equipment to 50Hz and

that a machine relying on this method could fail in a short period of time (applicable to Oxygen Concentrator, Warming Crib, Radiant Warmer).

RESEARCH QUESTIONS

Throughout the Consensus Meeting discussions, the following research questions were identified:

- **Syringe Pump**: During this discussion, clinicians explained that bulk weight of pumps and footprint of instruments is a challenge and emphasized the importance of stackability and interlocking devices. A research question for product developers was created to further explore how to optimize the stacking of equipment together and the ability to address concerns with the weight of heavy pumps.
- **Bilirubinometer**: One research question that emerged during the pricing discussion was to evaluate the long-term cost effectiveness of a point-of-care Bilirubinometer vs. clinical diagnosis or current standard of care by measuring and evaluating the number of false positives and false negatives based on clinical diagnosis data versus a point-of-care tool. The proposal was that the outcome of this comparison could be used to justify the value of the point-of-care tool.
- **Glucometer**: The most accessible point-of-care glucometers are designed to be accurate at high glucose ranges for management of adult diabetes; few are intended for use or accurate in the low glucose concentrations seen in hypoglycemic newborns. The group discussed the need to compare and measure the performance of adult glucometers at neonatal-relevant measures vs. neonate specific glucometers.
- **CPAP**: A research question was created to further explore outcomes and effects with and without heated humidification. Some clinicians commented that humidification helps with the avoidance of hypothermia which is becoming increasingly important. These clinicians claimed that it is likely that heated and humidified air is most important for the smallest newborns less than 1-1.25kg. Other clinicians responded that the mortality impact has never been explicitly studied.
- **CPAP**: A research question was created to further explore the impact of reusable accessories. An existing JHPIEGO paper "Infection Prevention and Control - Module 6. Processing Surgical Instruments and Medical Devices" was referenced in providing recommendations on how to develop guidelines on the reprocessing of single-use device [7, p. 77-81].
- **Respiratory Rate Monitor:** International standards for respiratory rate accuracy do not currently exist. There is therefore a need to define gold standard for respiratory rate accuracy and standardize experimental conditions. Ethical considerations are important in evaluating and validating these standards at upper and lower ranges on neonates. One participant recommended that both SpO2 and respiratory rate accuracy thresholds be based on real clinical data (typical variability). In the Pre-Meeting report survey, one individual commented that given there was not a 'gold standard' measurement for respiratory rate, they specified a reasonable reference standard with human experts and video recordings and specifying an acceptable degree of agreement with that standard, using the 95% Limits of Agreement and the Bland-Altman plot. However, an international NGO responded that using humans as a 'reasonable reference standard' can be troublesome since they can often be inconsistent or incorrect. Furthermore, they noted that "regulators will likely not see [human experts] as a means to validate".

- **Respiratory Rate Monitor**: A research question was established to review existing literature on power cuts to determine how long power supply should last. One meeting participant subsequently sent the following recommendations providing data on power cuts to share with the broader group in this report: 1) Limited electricity access in health facilities of sub-Saharan Africa: a systematic review of data on electricity access, sources, and reliability [66] 2) Oxygen insecurity and mortality in resource-constrained healthcare facilities in rural Kenya [67] and 3) Assessment of Power Availability and Development of a Low-Cost Battery-Powered Medical Oxygen Delivery System: For Use in Low-Resource Health Facilities in Developing Countries [68].
- **Radiant Warmer:** A research question to further explore the time required to indicate the accurate temperature of the baby and to measure the time in a standardized way was created.
- **Applicable to All**: Given the wide range of staff and infrastructure at health facilities, how do you validate technologies for Target Setting?

FUTURE NEONATAL TPPS

Due to time limitations, we were unable to create additional Target Product Profiles at the Consensus Meeting, however, a survey identified the need for future development of TPPs in the following areas:

- Hydration, Nutrition and Drug Delivery
  - Breast milk pump
  - Lactation support tools (e.g., storage bags)
  - Total parenteral nutrition (TPN)
  - Milk banking
- Jaundice Management
  - ROP screening and treatment
  - Retinopathy camera (e.g., RetCam)
- Point-of-Care Diagnostics
  - C-reactive protein (CRP) point-of-care test
- Respiratory Support
  - Mechanical ventilator
  - Oxygen blender
  - Bedside pulmonary function testing
  - Newborn resuscitation device Note: There is an existing document WHO Technical specifications of Neonatal Resuscitation Devices could be a helpful starting point for the development of this TPP noting changes may need to be considered for affordability, ease of use, etc [69].
  - Electrocardiogram (ECG)
- Thermal Management
  - Incubator

    - Cooling mattresses for therapeutic hypothermia
    - Infrared / Spot Check Thermometer (Temperature test)
    - Non-electric infant warmers (e.g., Phase Change Material)
- Other
    - Multi-parameter monitoring
    - Advanced hemodynamic monitoring
    - Transport
        - Oxygen delivery during transport
        - Transporter
        - Transport incubator
        - Infusion pump
        - Portable ultrasound
    - Cranial ultrasound
    - Backup power package
    - Maintenance package

No immediate next steps, beyond surveying participants about the TPPs that need to be developed were identified, but the report authors acknowledge the need for coordination and can coordinate interested parties moving forward. If you are interested in adapting this Delphi-like process for future development of these or other TPPs for newborns, please contact Becca Kirby (Becca.kirby@kellogg.northwestern.edu) and Kara Palamountain (k-palamountain@kellogg.northwestern.edu).

FINAL TARGET PRODUCT PROFILES

Please refer to each product category section below for the final target product profile.

HYDRATION, NUTRITION, AND DRUG DELIVERY

Small and sick babies have special fluid and nutritional requirements [8,9]. Intravenous (IV) infusions of water, electrolytes and glucose are given to neonates during the first weeks of life to maintain fluid and electrolyte balances and to provide energy for basic metabolic processes [10]. Fluid therapy requires delivery at precise volumes and flow rates, and fluid overload can be life-threatening [10,11].

SYRINGE PUMP

INTRODUCTION: SYRINGE PUMP

Syringe pumps deliver medication and small quantities of fluids continuously through an intravenous line and are a “priority medical device” as described by the World Health Organization. In high-resource hospitals, syringe pumps are used to provide rehydration fluids, breastmilk, dextrose to hypoglycemic infants, and antibiotics to infants with infection. In hospitals where syringe pumps do not exist or are unable to be maintained or operated, these fluids are delivered via a gravity-fed IV drip, slow push by nurses, or using burettes. These are all much less accurate methods of delivery and put infants at significant risk of over/under dosing, medical error, line complications, fluid overload, or hypovolemia. Additionally, since premature babies are likely to need slow introduction to breastmilk over the first week of life, syringe pumps are critical to maintaining normal glucose and hydration until preterm infants can tolerate adequate volumes of breastmilk orally or by nasogastric tube. For these reasons, syringe pumps were listed as a pressing technology for improving newborn care in The Global Action Report on Preterm Birth [12].

The FDA has reported that syringe pumps currently on the market are difficult to use [13]. Moreover, existing syringe pumps are expensive, and require costly, brand-specific consumables, making them unsuitable for use outside of high-resource settings. To be effective in reducing infant mortality on a global scale, pumps must be designed with a simple user interface to avoid setup errors and function accurately with the variety of syringe brand and sizes. In addition to withstanding hot and humid environments, the pump must be easily calibrated and maintained by local technicians. Syringe pumps are often unavailable for infants in need of life-saving IV treatment.

Table 1: Final TPP for Syringe Pump

Final target product profile for Syringe Pump		
**Characteristic**	**Optimal**	**Minimal**
SCOPE		
**Intended Use**	Treatment of conditions requiring precise administration of drugs and fluids; including but not limited to dextrose solution for hypoglycemia and antibiotics for infection	
**Target Operator**	For use in low- and middle-income countries by a wide variety of clinicians, including nurses, clinical officers, and pediatricians	
**Target Population**	Neonates (born at any gestational age and require ongoing care)	
**Target Setting**	Hospitals in low-resource settings	
SAFETY AND STANDARDS		
**Quality Management** [1]	ISO 13485:2016 Medical devices – Quality management systems -- Requirements for regulatory purposes	
**Regulation**	At least one of: CE marking, approved by US FDA or another stringent regulatory body of a founding member of IMDRF (e.g., Japan or Australia or Canada)	
TECHNICAL CHARACTERISTICS		
**Benchtop Measurement Accuracy (for Flow Rate)**	±1.0%	±3.0%
**Flow Rate Requirements**	0.1 - 60 mL/hr	
**Occlusion Detection**	Continuous adjustment (fully adjustable)	Adjustable based on pre-set (5, 10, 25 psi)
**Syringe Requirements**	Syringe 5-60mL, works with multiple syringe types	
**Drug Library**	Yes	No
**Alarm Characteristics**	Visual and Auditory	

**Size**	Small footprint; portable	
**Weight**	<1.5 kg (without batteries)	<5 kg (without batteries)
PURCHASING CONSIDERATIONS		
**Instrument Pricing**	<$300 ex-works	<$1,000 ex-works
UTILITY REQUIREMENTS		
**Power Source**	Mains with rechargeable battery	Mains with rechargeable battery
**Battery**	Rechargeable battery, >12hr on single charge	Rechargeable battery, >4hr on single charge
**Voltage**	Model must match the voltage and frequency of the purchasing country's local power grid (e.g., 110-120 VAC at 60 Hz or 220-240 VAC at 50 Hz)	
TRAINING AND MAINTENANCE		
**User Instructions**	User manual and additional training materials (checklists, videos, guides) in at least one national official language for the country of intended use. Attached to device with labels and markings where possible	User manual provided in at least one national official language
**Warranty**	5 years	1 year
**Decontamination**	Easy to clean with common disinfecting agents	

[1] There was not 75% voting agreement on the Minimal characteristic. Please refer to the TPP Report discussion for additional detail.

CONSENSUS MEETING SUMMARY: SYRINGE PUMP

To arrive at the final TPP for Syringe Pump (Table 1), we conducted a pre-meeting survey to prioritize the items for discussion at the Consensus Meeting for characteristics that achieved below 75% agreement in the survey results (Table 2). An overview of the discussion at the Consensus Meeting of these characteristics is included below.

- **Benchtop Measurement Accuracy (for Flow Rate)**
  - Clarification was added to the Benchtop Measurement Accuracy characteristic to confirm the reference to flow rate. Consensus was achieved in the room (without a Mentimeter vote) for the Optimal and Minimal characteristic. Participants noted that current commercial standards specify

±2-3% and that accuracy is self-declared and listed on the insert, but that a standard does not currently exist. Volume accuracy is dependent on whether the device is being used for administering fluids or drugs.
  - *Optimal: ± 1.0%*
  - *Minimal: ± 3.0%*
- **Clinical Measurement Accuracy**
  - This characteristic was deleted from the TPP as it is not reported or tested.
- **Flow Rate Requirements**
  - Consensus was achieved in the room (without a Mentimeter vote) for the Optimal and Minimal characteristic. There was a lengthy discussion on the tradeoffs of broadening the Minimal and Optimal characteristics to 0.1-60 mL/hr. It was discussed that if cost is not significantly impacted, then clinicians wanted to broaden the range. Product developers noted that from a technical perspective, it was not a challenge to have a broad range, but rather it was dependent on the syringe size and brand since the brand impacts the performance. Product developers were not certain whether a lower limit of 0.1 ml/hr would impact the price. Clinicians clarified that Syringe Pumps designed for administering fluids, may require less stringent accuracy than drug administration. Healthcare workers noted that Syringe Pumps are often used in neonatal units for fluid delivery and that district hospitals do not typically use Syringe Pumps for drug delivery. A question arose on the difference between a Syringe Pump and an Infusion Pump and how the two pieces of equipment differ. Some clinicians noted that increased accuracy would be beneficial from a procurement standpoint since one device could be procured to meet both purposes.
  - *Optimal: .1 to 60 ml/hr*
  - *Minimal: Same as Optimal*
- **Occlusion Detection**
  - Consensus was achieved in the room for the Optimal (without a Mentimeter vote) and Minimal characteristic. Participants commented that the normal pressure used to detect an occlusion is usually 0.1 to 15 or 17 psi for an adjustable range. Clinicians confirmed that for the most part, they do not generally change the pressure. Participants in the room confirmed that for neonates, it is important to set specific graduations but the specific numbers do not need to be defined in the TPP. One participant shared an article on "The Safe Use of Infusion Devices" which provided specific pressures for neonates: "In neonates, pressures of 50 mm Hg are typical because of lower flow rates and shorter cannula... Neonatal default settings are much lower (100 mm Hg)," [14].
  - *Optimal: Completely adjustable*
  - *Minimal: Yes (ability to adjust to pre-set pressure)*
    - Overall Vote - 92% Agree (n = 13)
    - Clinicians - 92% Agree (n = 12)
    - Excluding involvement with product development - 91% Agree (n =11)
- **Ability to calculate flow rates based on patient size**
  - This characteristic was not discussed as it was determined that it should be removed from the TPP.
- **Syringe Requirements**
  - Consensus was achieved in the room for the Optimal (without a Mentimeter vote) and Minimal characteristic. Participants removed the “failsafe mode to reject syringes that don’t match machine setting” from the Optimal characteristic.

- Optimal: *Syringe 5-60mL, works with multiple syringe types*
- *Minimal: Syringe 5-60mL and works with more than 1 syringe type*
  - Overall Vote - 100% Agree (n = 11)
  - Clinicians - 100% Agree (n = 9)
  - Excluding involvement with product development - 100% Agree (n =10)

- **Alarm Characteristics**
  - Consensus was achieved in the room (without a Mentimeter vote) to change the Minimal characteristic to both Visual and Auditory alarms (same as Optimal). Product developers noted that a trade-off exists between the number of alarms and the size of the device.
  - *Optimal: Visual and Auditory*
  - *Minimal: Same as Optimal*
- **Maximum Power Consumption**
  - This characteristic was not discussed as it was determined to remove from the TPP. Note that a new characteristic, Power Source, was added to the TPP.
- **Voltage**
  - There was agreement in the room that all characteristics relating to Utility Requirements (e.g., Back-up Battery; Battery Power; Batteries; Voltage; Power Requirement; Maximum Power Consumption; Response During Power Outage; Surge Protection, Electrical Plug) be reviewed and harmonized following the TPP meeting.
  - *Optimal and Minimal: Model must match the voltage and frequency of the purchasing country's local power grid (e.g., 110-120 VAC at 60 Hz or 220-240 VAC at 50 Hz)*
- **Battery**
  - There was agreement in the room that all characteristics relating to Utility Requirements (e.g., Back-up Battery; Battery Power; Batteries; Voltage; Power Requirement; Maximum Power Consumption; Response During Power Outage; Surge Protection, Electrical Plug) be reviewed and harmonized following the TPP meeting.
  - *Optimal: Rechargeable battery, >12hr on single charge*
  - *Minimal: Rechargeable battery, >4hr on single charge*
- **Weight**
  - Consensus was achieved in the room (without a Mentimeter vote) for the Optimal and Minimal characteristic. Clinicians noted that current machines are less than 2kg. Product developers noted that from a technical perspective, battery packs requiring 12 hours on a single charge could make the machine heavier. Clinicians explained that bulk weight accumulates quickly and emphasized the importance of stackability and interlocking devices. A research question for product developers was created to further explore how to optimize the stacking of equipment together and the ability to address concerns with the weight of heavy pumps.
  - *Optimal: <1.5kg (without batteries)*
  - *Minimal: <5kg (without batteries)*
- **Instrument Pricing**
  - Consensus was achieved in the room (without a Mentimeter vote) for the Optimal and Minimal characteristic.

- *Optimal: <$300 ex-works*
- *Minimal: <$1,000 ex-works*

- **Consumable Pricing**
  - Consensus was achieved to remove this characteristic since consumables are purchased separately for Syringe Pumps.

DELPHI-LIKE SURVEY: SYRINGE PUMP

Table 2: Delphi-like survey results for Syringe Pump TPP prior to Consensus Meeting (data as of Oct 25, 2019)

	Optimal		Minimal		
**Characteristic**	**Optimal requirement**	**% agreement (n size)**	**Minimal requirement**	**% agreement (n size)**	**Collated comments from Delphi-like survey**
**Intended Use**	Optimal: Treatment of conditions requiring precise administration of drugs or fluids; including but not limited to dextrose solution for hypoglycemia and antibiotics for infection.	100%<br>n = 9	Minimal: Same as Optimal.	88%<br>n = 8	3 comments as summarized below<br>• Theme: Alternative Intended Use provided<br>o Optimal: Treatment of conditions requiring precise administration drugs or fluids; including but not limited to dextrose solution of hypoglycemia, antibiotics for infection and feed advancement in small infants or infants at risk for HIE. I'd have to check how many of the essential newborn medicines actually need the meds to go over syringe pump? Mostly we found syringe pumps to be key for administering precise fluids to small infants<br>o Optimal: I would like if it could take every syringe size, would stop when the baby has been filled. It needs to be human proof<br>o Optimal: Treatment of conditions requiring precise administration of drugs or fluids
**Target Operator**	Optimal: For use in low- and middle-income countries by a wide variety of clinicians, including	100%<br>n = 10	Minimal: Same as Optimal	100%<br>n = 9	0 comments

	Optimal		Minimal		
	nurses, clinical officers, and pediatricians.				
**Target Population**	Optimal: Neonates (<28 days)	80%<br>n = 10	Minimal: Same as Optimal.	78%<br>n = 9	4 comments as summarized below<br>• Theme: Broaden to other Target Populations<br>o Ideally, when thinking about syringe pumps, it would be great if they also are able to be used for diverse needs in a hospital (oxytocin, pediatrics) rather than only for neonates. This can ease burden on hospitals if they have one pump type that can be used across many services<br>• Optimal: Proportionately, you would be using it more on neonates. I would want one or two for my pediatric ward for my hypertensives, convulsing, diabetic children<br>• Neonates, pediatrics and adults
**Target Setting**	Optimal: Hospitals in low-resource settings	100%<br>n = 9	Minimal: Same as Optimal.	100%<br>n = 8	0 comments
**International Standard**	Optimal: ISO 13485:2016 Medical devices – Quality management systems -- Requirements for regulatory purposes.	83%<br>n = 6	Minimal: Same as Optimal.	80%<br>n = 5	0 comments
**Regulation**	Optimal: CE marking or US FDA Clearance	71%<br>n = 7	Minimal: Same as Optimal.	67%<br>n = 6	3 comments as summarized below<br>• CE mark is more than adequate<br>• Performance is more important

	Optimal		Minimal		
**Benchtop Measurement Accuracy**	Optimal: ±1.0%	**57%**<br>**n = 7**	Minimal: ±3.0%	**33%**<br>**n = 6**	6 comments as summarized below<br>• What's the difference between Clinical and Benchtop accuracy? The correct term is flow rate accuracy. Any CE marked product is bound to meet this spec<br>• Theme: Too Stringent<br>o If you're loading the maximum amount of something that can be given into the syringe, then it doesn't seem like it matters too much if it's a little fast or a little slow? Max fluids to a neonate in a day is between 150-180ml/kg/day and 1% of that would only be 1.5-1.8ml/kg/day inaccuracy (max 5ml if you guess newborns on average are 2.5kg) per day? I think you could go 5% maybe even 10% and it wouldn't really matter?? Or at least off the top of my head I can't think why it would<br>o These errors are very low. Unlikely to make a clinical difference<br>o Way too small! Suggest updating to: Benchtop Measurement Flow Rate and Volume Accuracy<br>▪ Optimal: ±5%<br>▪ Minimal: ±10%<br>• Theme: Not Stringent Enough<br>o Minimal: Ideally I would want benchtop accuracy to be tighter<br>o Minimal: I would want 2% the smaller volume (e.g., insulin) and neonates I would like it to be more accurate

	Optimal		Minimal		
**Clinical Measurement Accuracy**	Optimal: ±1.0%	**63%**<br>**n = 8**	Minimal: ±3.0%	**57%**<br>**n = 7**	3 comments as summarized below<br>• Too Stringent<br>o Way too small! Suggest updating to: Clinical Measurement Flow Rate and Volume Accuracy<br>▪ Optimal: ±10%<br>▪ Minimal: ±15%<br>• Not Stringent Enough<br>o Minimal: I would want 2% the smaller volume (e.g., insulin) and neonates I would like it to be more accurate
**Flow Rate Requirements**	Optimal: 0.5-60mL/hr	**78%**<br>**n = 9**	Minimal: 3-30mL/hr	**63%**<br>**n = 8**	4 comments as summarized below<br>• One respondent said, range of ml/hr for most infants (those less than 3.5kg) will range only 1-12 mL/hr. Up to 20 mL/hr would accommodate a 5kg infant, up to 40 mL/hr for a 10kg infant.<br>• Theme: Additional ranges were suggested<br>o Optimal: I would want to go as low as .25 mL/hr; with antibiotic you may need slower rate and also adrenaline you would want to go sometimes as low as .1 (ICU care)<br>o This needs to be lower if indications is for neonates<br>o The Optimal flow range is misleading because it can be across so many different syringe sizes. Suggest adding clarity to the Optimal and increasing the Minimal to larger<br>• Optimal: 0.5-60 mL/hr (different syringe sizes allowed)<br>• Minimal: 3-60 mL/hr

	Optimal		Minimal		
**Occlusion Detection**	Optimal: Adjustable	**67%**<br>**n = 9**	Minimal: 5, 10, 25 psi	**71%**<br>**n = 7**	3 comments as summarized below<br>• Maybe I need to educate myself on this, not sure what you would need to adjust the sensitivity for occlusion? Like you get false positives if the IV gauge is smaller or something?<br>• Optimal: I would want the syringe pump to be set at a reasonable amount. I am not clear what reasonable is<br>• Occlusion detection is required but the exact alarm setting is not important
**Syringe Requirements**	Optimal: Syringe 5-60mL, works with multiple syringe types. Failsafe mode to reject syringes that don't match machine settings.	**80%**<br>**n = 10**	Minimal: 5-60mL, proprietary syringes	**56%**<br>**n = 9**	6 comments as summarized below<br>• I'd have to review which essential neonatal medications would benefit from a syringe pump to make a comment on whether or not you needed to accommodate syringe sizes this small and if introducing this complexity seems necessary (We never used syringe pumps for meds in our wards)? If mainly used for fluids then needs to accommodate syringes 50-100ml<br>• Theme: Considerations related to proprietary syringes<br>o Working with multiple syringe types should be the minimum - proprietary syringes are really hard for procurement.<br>o If I have to have a proprietary, I would like it without the falange.<br>o This requirement is confusing as proprietary vs non-proprietary is not quantifiable. The minimal should be that the pump works with multiple syringe brands and list the brands most commonly used.<br>o Should be able to work with multiple syringe types to avoid downtimes when

	Optimal		Minimal		
					proprietary syringes are not available for any reason.
**Ability to calculate flow rates based upon patient's size**	Optimal: Yes	**100%**<br>**n = 8**	Minimal: No	**71%**<br>**n = 7**	3 comments as summarized below<br>• As long as programming rates can be done within the needed range, this is not essential<br>• Optimal: Size might be unrelated to what I am trying to give them. this would only work if it was calibrated for maintenance fluids; you couldn't just have a standard. As long as you can put in the patient size and then select the drug / fluid<br>• I would like to see this as mandatory
**Drug Library**	Optimal: Yes	**88%**<br>**n = 8**	Minimal: No	**86%**<br>**n = 7**	4 comments as summarized below<br>• Not sure what this is referring to<br>• This is a great feature - not essential as the pump can fully meet needs without it but it can ease programming<br>• Optimal: Not needed<br>• Optimal: If it says give so many mgs, that would be ok. If it says give so many mL, then that would be problematic. It increases the chance of user misinterpretation depending on the concentration of drug being added
**Alarm Characteristics**	Optimal: Visual and Auditory	**100%**<br>**n = 9**	Minimal: Visual	**38%**<br>**n = 8**	5 comments as summarized below<br>• Theme: Auditory preferred over visual<br>o Why visual as minimal instead of auditory?<br>o I feel that the minimum alarm requirements should include auditory alarms. In my experience, syringe pumps may be left without close monitoring for several hours. If clinicians are in another room or not in visual sight of the alarm, an auditory alarm would be more beneficial than a visual alarm

	Optimal		Minimal		
					o Visual and auditory alarms are required as minimal specifications<br>o Minimal: If there was one alarm function, I would prefer it to be auditory over visual
**Decontamination**	Optimal: Easy to clean with common disinfecting agents	**100%**<br>**n = 9**	Minimal: Same as Optimal.	**100%**<br>**n = 8**	1 comment<br>• Optimally would be able to re-use large (50-100ml) syringes
**Maximum Power Consumption**	Optimal: <1 Watt	**57%**<br>**n = 7**	Minimal: <5 Watts	**50%**<br>**n = 6**	3 comments as summarized below<br>• Lower power consumption is helpful, but not essential given all the other priority features<br>• What is the rationale for picking these power specs and need for this requirement? Given battery powered is a requirement below, then there is no need to include AC power consumption. Also Max power consumption is not indicative of what the device will consume on average over X number of hours of operation (Power draw might be an additional spec needed for all devices)<br>• Specifications not relevant
**Voltage**	Optimal: 110-240V 50-60hz	**86%**<br>**n = 7**	Minimal: 220-240V 50-60hz	**67%**<br>**n = 6**	1 comment<br>• Depend of the destination country (or 110 V or 220 V)
**Battery Power**	Optimal: >4hr on single charge	**100%**<br>**n = 9**	Minimal: None.	**38%**<br>**n = 8**	6 comments as summarized below<br>• Theme: Minimal should include battery back-up<br>o I think these have to have a battery option, they're really essential<br>o As a minimum, some power battery backup should be included<br>o Minimal: Battery power should be able to handle up to 2 hours of power outages

	Optimal		Minimal		
					o Minimal: Our power is so unreliable that it would cause me enormous anxiety not to know what is going on so I don't know what has been given when the power goes back on<br>o Must have a battery<br>o Battery backup is necessary to maintain drug administration which could be life saving, especially in areas with epileptic power supply
**Size**	Optimal: Small footprint; portable	**100%**<br>**n = 9**	Minimal: Same as Optimal.	**100%**<br>**n = 8**	1 comment<br>• Small footprint is difficult to define
**Weight**	Optimal: <7 kg	**75%**<br>**n = 8**	Minimal: <10 kg	**71%**<br>**n = 7**	2 comments as summarized below<br>• Theme: Specification not needed<br>o 7-10 kg are both quite heavy for a syringe pump, and weight plays into shipping and distribution costs so lighter is helpful but from a clinical use standpoint the weight is not really important.<br>o Specifications not relevant
**User Manual**	Optimal: User manual and additional training materials (checklists, videos, guides) in English and local language. Attached to device with labels and markings where possible.	**89%**<br>**n = 9**	Minimal: User manual provided.	**88%**<br>**n = 8**	2 comments as summarized below<br>• User manual at a minimum should be provided hard copy and soft copy, with easy online access<br>• Optimal: should include trouble shooting and how to clean
**Warranty**	Optimal: 5 years	**78%**<br>**n = 9**	Minimal: 1 year	**88%**<br>**n = 8**	3 comments as summarized below<br>• Theme: 5 years too long<br>o Five year warranty would be really great, but not expected as that is longer than most equipment so not essential.

	Optimal		Minimal		
					o Minimal: most business give 1 year warranty, but adding years shows confidence in the product.<br>o No supplier will agree with a 5 year warranty
**Instrument Pricing**	Optimal: <$1,000 ex-works	**78%**<br>**n = 9**	Minimal: <$2,000 ex-works	**38%**<br>**n = 8**	5 comments as summarized below<br>• Theme: Lower Optimal and Minimal<br>o To be competitive with models on the market - and in government procurement processes - the minimal costs should be <$1000 and Optimal even a bit lower.<br>o Optimal: $500 would be more acceptable<br>o Minimal: Only if it was a gift<br>o Needs to be lower<br>o These prices are too high. Consider changing to OPT: $250 and MIN: $1500 This is based on actual quotes
**Consumable Pricing**	Optimal: <$3 per patient ex-works	**100%**<br>**n = 6**	Minimal: <$10 per patient ex-works	**60%**<br>**n = 5**	1 comment<br>• Optimal: my comment would be that impacted by length of stay of the patient or per episode of illness

Figure 3: Summary of organizational affiliation for Syringe Pump TPP

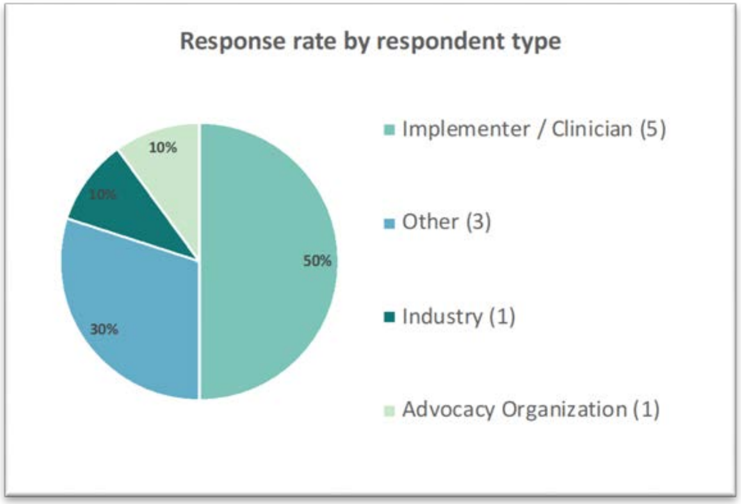

Respondent type	Percentage
Implementer / Clinician (5)	50%
Other (3)	30%
Industry (1)	10%
Advocacy Organization (1)	10%

Figure 4: Summary of response rate by country for Syringe Pump TPP

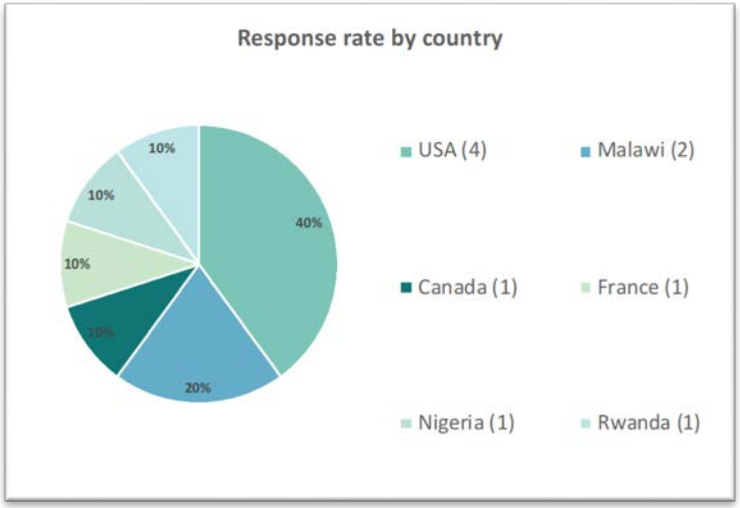

Country	Percentage
USA (4)	40%
Malawi (2)	20%
Canada (1)	10%
France (1)	10%
Nigeria (1)	10%
Rwanda (1)	10%

JAUNDICE MANAGEMENT

Most neonates, term and preterm, will have elevated levels of unconjugated bilirubin and some amount of jaundice during the first one to two weeks of life due to increased levels of unconjugated bilirubin with transient impaired excretion, which is normal in this age group. This condition is particularly prevalent in preterm babies and, if the levels of unconjugated bilirubin are very high and left untreated, may lead to irreversible neurologic damage known as kernicterus.

Phototherapy treats unconjugated hyperbilirubinemia that exceeds safe levels. These levels are based on day of life and risk factors and typically occur within the first one to two weeks of life.

Treatment with blue light phototherapy is necessary to prevent morbidity and mortality from dangerous levels of neonatal jaundice. The blue light is absorbed by bilirubin, which is then broken down in the blood, allowing the infant to excrete the excess bilirubin before it can accumulate and cause permanent brain damage (kernicterus) or death. Jaundice is preventable and treatable; however, kernicterus is permanent and irreversible, resulting in life-long disability.

INTRODUCTION: BILIRUBINOMETER

Severe jaundice may not be readily evident to the naked eye until already at dangerously high levels. Additionally, jaundice may not present until several days after birth when an infant has already left the hospital. Thus, early monitoring of bilirubin in at-risk infants is critical in order to prevent severe jaundice, which may result in permanent neurological damage, particularly in premature babies who are at greater risk of death and disability due to jaundice.

All infants should have a laboratory evaluation of serum bilirubin (with result turn around within six hours) both to diagnose jaundice and to guide treatment of infants receiving phototherapy. In low-resource settings though, many facilities do not have the ability to run a blood test, and those that do face many challenges both to run the test and obtain results within a meaningful timeframe.

The ideal solution in a low-resource setting would be a reliable point-of-care test which can test serum bilirubin both before and during phototherapy treatment.

FINAL TPP: BILIRUBINOMETER

Table 3: Final TPP for Bilirubinometer

Final target product profile for Bilirubinometer		
**Characteristic**	**Optimal**	**Minimal**
SCOPE		
**Intended Use**	Quantification of serum bilirubin in neonates for the diagnosis and management of jaundice at the patient's bedside	
**Target Operator**	For use in low- and middle-income countries by a wide variety of clinicians, including nurses, clinical officers, and pediatricians	
**Target Population**	Neonates (born at any gestational age and require ongoing care)	
**Target Setting**	Hospitals in low-resource settings	
SAFETY AND STANDARDS		

**Quality Management** [1]	ISO 13485:2016 Medical devices – Quality management systems -- Requirements for regulatory purposes	
**Regulation**	At least one of: CE marking, approved by US FDA or another stringent regulatory body of a founding member of IMDRF (e.g., Japan or Australia or Canada)	
TECHNICAL CHARACTERISTICS		
**Linear Range**	0-40 mg/dL (0-684 µmol/L)	5-30 mg/dL (85.5 - 513 µmol/L)
**Accuracy**	± 10% from 5-30mg/dL (85.5 - 513 µmol/L)	± 20% from 5-30mg/dL (85.5 - 513 µmol/L) [2]
**Results Format**	Quantitative across whole linear range	
**Result Units**	Must display mg/dL or µmol/L (shall have ability to select or switch between either)	
**Precision**	4% CV	15% CV
**Sample**	Whole blood heel-stick sample <50 µL; does not require user to separate serum/plasma using a centrifuge	
**Calibration**	No calibration	Minimal user calibration required
**Kit Stability & Storage**	Stable for >12 months with harsh ambient conditions (temperature 5-45 °C, humidity 15% to 95%, dusty air, elevation >=2000 meters) and transport stress (48h with fluctuations up to 50°C and down to 0°C)	Stable for 12 months with harsh ambient conditions (temperature 10-40 °C, humidity 15%-95% elevation up to 2000 meters) and transport stress (48h with fluctuations up to 50°C and down to 0°C)
**Equipment Required**	Small, portable or hand-held device; device-free/disposable preferred; does not require centrifuge	Small, table-top device; portable device optional; does not require centrifuge
PURCHASING CONSIDERATIONS		
**Instrument Pricing**	<$200 ex-works	<$800 ex-works
**Consumable Pricing**	<$0.50 per test ex-works	$1.50 per test ex-works
UTILITY REQUIREMENTS		
**Power Source**	No power required	Mains with rechargeable battery

**Battery**	None (i.e. a disposable test that requires no electricity)	Rechargeable battery, >100 tests on a single charge.
**Voltage**	None.	Model must match the voltage and frequency of the purchasing country's local power grid (e.g., 110-120 VAC at 60 Hz or 220-240 VAC at 50 Hz)

[1] *There was not 75% voting agreement on the Minimal characteristic. Please refer to the TPP Report discussion for additional detail.*
[2] *Source: https://www.westgard.com/2019-clia-changes.htm CLIA proposed changes define Accuracy as ±20%. These changes are proposed as of Feb 2019.*

CONSENSUS MEETING SUMMARY: BILIRUBINOMETER

To arrive at the final TPP for Bilirubinometer (Table 3), we conducted a pre-meeting survey to prioritize the items for discussion at the Consensus Meeting for characteristics that achieved below 75% agreement in the survey results (Table 4). An overview of the discussion at the Consensus Meeting of these characteristics is included below.

- **Linear Range**
  - Consensus was achieved in the room (without a Mentimeter vote) for the Minimal characteristic. Clinicians noted that the upper end of the range was more important (since above roughly 25mg/dL will not change behavior) and that 5mg/dL for the lower end of the range was acceptable. From a technical perspective, product developers noted that going above 25mg/dL was relatively easy up to 30mg/dL, especially compared to extending the lower end of the range. Product developers explained that reducing the lower end was more expensive, but 3-4mg/dL detection was reasonable from a manufacturing perspective.
  - *Minimal: 5 - 30 mg/dL* (85.5 - 513 μmol/L)
- **Accuracy**
  - Consensus was achieved in the room (without a Mentimeter vote) for both the Optimal and Minimal characteristics. Participants noted new proposed CLIA laboratory standards [15]. Clinicians mentioned that central laboratory results take more time in low-resource settings (often a minimum of 24 hours). Since clinicians may rely on quick turnaround point-of-care tests in low-resource settings, clinicians requested better accuracy at higher ends of the range, hence the decision to be more stringent than the proposed CLIA standards for the Optimal characteristic. Clinicians noted that at the high and low ranges though, their behavior for treatment would likely not change. Product developers noted that it is a "big ask" to improve beyond 10% accuracy as a centrifuge and other lab equipment for blood sample testing would be required.
  - *Optimal: ±10% from 5-30mg/dL (85.5 - 513 μmol/L)*
  - *Minimal: ±20% from 5-30mg/dL (85.5 - 513 μmol/L)*
- **Results Format**
  - Consensus was achieved in the room (without a Mentimeter vote) for the Minimal characteristic to equal the previously agreed upon Optimal characteristic.

- *Optimal: Quantitative across whole linear range.*
- *Minimal: Same as Optimal.*

- **Results Unit**
  - Consensus was achieved in the room (without a Mentimeter vote) for both the Optimal and Minimal characteristics.
  - *Optimal: Must display mg/dL or µmol/L (shall have ability to select or switch between either)*
  - *Minimal: Same as Optimal.*
- **Instrument Pricing**
  - Consensus was achieved in the room (without a Mentimeter vote) to keep the Minimal characteristic under $800 ex-works and emphasize the disagreement in the room on setting a reasonable price. Participants highlighted that the cheaper the price the better, however, noted the clear tradeoff between instrument and consumable pricing (i.e., if consumables were cheap at $0.05 per test, then $800 could be acceptable). Since there are not many benchmarks on the market, the price point for what this would cost is not clear. One research question for the future would be to evaluate the number of false positives and false negatives based on clinical diagnosis data versus a point-of-care tool. The outcome of this comparison, may be used to justify the purchase of the point-of-care tool.
  - *Minimal: <$800 ex-works*
- **Number of steps**
  - Consensus was achieved in the room (without a Mentimeter vote) that the fewer steps the better and therefore, it was suggested that this characteristic be removed from the TPP since there was variation in measurement of the number of steps.

DELPHI-LIKE SURVEY: BILIRUBINOMETER

Table 4: Delphi-like survey results for Bilirubinometer TPP prior to Consensus Meeting (data as of Oct 25, 2019)

	Optimal		Minimal		
**Characteristic**	**Optimal requirement**	**% agreement (n size)**	**Minimal requirement**	**% agreement (n size)**	**Collated comments from Delphi-like survey**
**Intended Use**	Optimal: Quantification of total serum bilirubin in neonates for the diagnosis and management of jaundice at the patient's bedside	**92%**<br>**n = 13**	Minimal: Same as Optimal.	**100%**<br>**n = 12**	1 comment<br>• Ideally, would pair together the ability to simultaneously test for Coombs positivity and bilirubin on the same POC machine<br>• I would also say Optimally this would report direct and indirect separately (would diversify its utility to other parts of the hospital outside of neonates)"

	Optimal		Minimal		
**Target Operator**	Optimal: For use in low- and middle-income countries by a wide variety of clinicians, including nurses, clinical officers, and pediatricians.	**100%**<br>**n = 13**	Minimal: Same as Optimal	**100%**<br>**n = 12**	0 comments
**Target Population**	Optimal: Neonates (<28 days)	**85%**<br>**n = 13**	Minimal: Same as Optimal.	**100%**<br>**n = 11**	4 comments as summarized below<br>• Theme: Broaden age range<br>o Sometimes you have babies that are > 28 days. e.g., 40 days would be ideal<br>o Optimally this could be used in older people as well, not sure if fetal hemoglobin is affecting how this test works or not
**Target Setting**	Optimal: Hospitals in low-resource settings	**83%**<br>**n = 12**	Minimal: Same as Optimal.	**91%**<br>**n = 11**	2 comments as summarized below<br>• Theme: Broaden Target Setting<br>o Potentially higher income countries<br>o The jaundiced babies will be referred from lower level facilities; health centers and this test should be available from those lower level facilities up to hospitals so as to benefit all the at-risk babies<br>o Minimal: hospital in resource-limited settings, Optimal: health centers (primary)
**International Standard**	Optimal: ISO 13485:2016 Medical devices – Quality management systems -- Requirements for regulatory purposes.	**100%**<br>**n = 7**	Minimal: Same as Optimal.	**100%**<br>**n = 6**	0 comments
**Regulation**	Optimal: CE marking or US FDA Clearance	**89%**<br>**n = 9**	Minimal: Same as Optimal.	**88%**<br>**n = 8**	1 comment as summarized below<br>• Consider additional ‘or’ options:

	Optimal		Minimal		
					o Other Stringent Regulatory Authorities – Japan or Australia or Canada<br>o Consider regulatory bodies of Low- and Middle-Income Countries
**Linear Range**	Optimal: 0-40 mg/dL	**91%**<br>**n = 11**	Minimal: 0-30 mg/dL	**64%**<br>**n = 11**	6 comments as summarized below<br>• Theme: A variety of ranges were suggested<br>o Minimal would be 5-25 (these are the clinically meaningful numbers for intervention in terms of both phototherapy and exchange transfusion. Having accuracy outside of this window may interest people for research reasons? But won't change clinical management that I'm aware of<br>o Minimal is still too high – should be more like 20<br>o As long as minimal has high reading for >30
**Accuracy**	Optimal: Within 20% or 0.4 mg/dL, whichever is greater	**69%**<br>**n = 13**	Minimal: Same as Optimal.	**55%**<br>**n = 11**	7 comments as summarized below<br>• Theme: Accuracy definition needs clarity. A range of perspectives were provided.<br>o "Needs to be whichever is lower, 20% or .4 mg/dL"<br>o "Given that the range in which most clinically meaningful bilirubin decisions would be made (5-25mg/dL) +/- 20% seems too generous? +/- 1mg/dL serum bili seems more reasonable to me"<br>o "Need more accuracy<br>o Recommend changing minimal to within 25% or 2mg/dL"<br>o "0.4mg/dL is reasonable. However 20% would not be acceptable in higher values. For example of the bilirubin level is 20

	Optimal		Minimal		
					mg/dL and the accuracy ranges from 18-22mg/dL, that could alter management decisions if it were 18 or 22. 20% or 0.4mg/dL whichever is lower would be more appropriate."
**Results Format**	Optimal: Quantitative across whole linear range	100%<br>n = 13	Minimal: Quantitative; semi quantitative below 2 or above 20 mg/dL	67%<br>n = 12	3 comments as summarized below<br>• Theme: Minimal should require quantitative across the whole linear range
**Result Units**	Optimal: mg/dL and mmol/L	92%<br>n = 12	Minimal: Same as Optimal.	64%<br>n = 11	5 comments as summarized below<br>• Theme: Variation in unit defined in guidelines across countries<br>o "We are used to mmol/L but international guidelines use mg/dL"<br>o "mmol/L because our guidelines are written mmol/L"<br>o "Most tables are labeled with both so I think reporting in one or the other is also fine"<br>o "Easy to change"<br>o "Minimal might be. 'mg/dL or mmol/L' set at factory pre-shipment'"
**Precision**	Optimal: 4% CV	88%<br>n = 8	Minimal: 15% CV	86%<br>n = 7	3 comments as summarized below<br>• Theme: Precision / CV is not an understood term / unit
**Sample**	Optimal: whole blood heel-stick sample <50 μL; does not require user to separate	100%<br>n = 11	Minimal: Same as Optimal.	100%<br>n = 10	4 comments as summarized below<br>• Theme: Questions about Sample type<br>o Venipuncture blood<br>o No blood stick

	Optimal		Minimal		
	serum/plasma using a centrifuge				
**Number of Steps**	Optimal: No more than 1-4 steps (requiring operator intervention)	92%<br>n = 12	Minimal: No more than 4-6 steps (requiring operator intervention)	64%<br>n = 11	6 comments as summarized below<br>• Theme: Variation in responses to 4-6 steps<br>o Short, precise instruction required<br>o Again, not sure on standards here but seems reasonable<br>o 6 steps not feasible<br>o Fewer is better, but 4-6 is okay for minimal<br>o 4-6 steps is too much<br>o How do we quantify this?
**Calibration**	Optimal: No calibration	85%<br>n = 13	Minimal: Minimal user calibration required	85%<br>n = 13	3 comments as summarized below<br>• Theme: Challenges with requiring calibration in certain settings<br>o People won't calibrate<br>o How do we quantify minimal?<br>o If it does not need user calibration, that would be better especially in smaller hospitals where systems may not be robust<br>o Optimal: Calibration will always be needed unless there is external QA
**Kit Stability & Storage**	Optimal: Stable for >12 months with harsh ambient conditions (temperature 5-45 °C, humidity 15% to 95%, dusty air, elevation >=2000 meters) and transport stress (48h with fluctuations up to 50°C and down to 0°C)	100%<br>n = 12	Minimal: Stable for 12 months with harsh ambient conditions (temperature 10-40 °C, humidity 15%-95% elevation up to 2000	92%<br>n = 12	3 comments as summarized below<br>• Theme: Is this technically feasible?

	Optimal		Minimal		
			meters) and transport stress (48h with fluctuations up to 50°C and down to 0°C)		
**Equipment Required**	Optimal: Small, portable or hand-held device; device-free/disposable preferred; does not require centrifuge	85%<br>n = 13	Minimal: Small, table-top device; portable device optional; does not require centrifuge	92%<br>n = 12	3 comments as summarized below<br>• Concerns with theft of hand-held devices<br>• The minimal and Optimal might be the same. Both should be small, but hand-held vs. table top does not give a clear advantage either way
**Power Requirement**	Optimal: None (i.e. a disposable test that requires no electricity)	83%<br>n = 12	Minimal: 110-220V AC current; DC power with rechargeable battery lasting up to 8 hours of testing	91%<br>n = 11	3 comments as summarized below<br>• Does this mean it requires batteries? If so, I would rather have the rechargeable option<br>• Does none mean batteries required? If so, I don't agree<br>• "This category is not consistent with other similar battery backed up devices (pulse-ox, temp monitor). The product configuration requires some type of electricity. May need to separate and reformat category here."
**Instrument Pricing**	Optimal: <$200 ex-works	91%<br>n = 11	Minimal: <$800 ex-works	60%<br>n = 10	3 comments as summarized below<br>• Theme: $800 is considered high
**Consumable Pricing**	Optimal: <$0.50 per test ex-works	100%<br>n = 10	Minimal: $1.50 per test ex-works	78%<br>n = 9	3 comments as summarized below

	Optimal		Minimal		
					• No test (on the market or in development) will be able to meet the minimal currently. $2.00 is more feasible<br>• Expensive. Around $1 may be okay

Figure 5: Summary of organizational affiliation for Serum Bilirubin Test TPP from Delphi-like Survey prior to Consensus Meeting (data as of Oct 25, 2019)

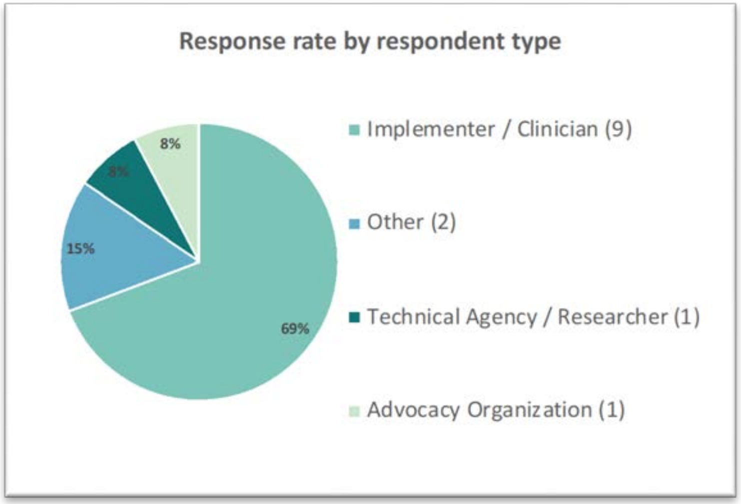

Respondent type	Percentage
Implementer / Clinician (9)	69%
Other (2)	15%
Technical Agency / Researcher (1)	8%
Advocacy Organization (1)	8%

Figure 6: Summary of response rate by country for Serum Bilirubin Test TPP from Delphi-like Survey prior to Consensus Meeting (data as of Oct 25, 2019)

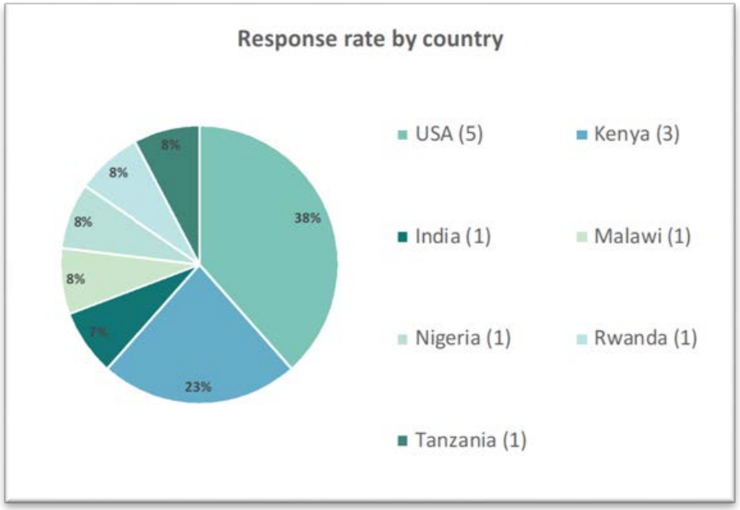

Country	Percentage
USA (5)	38%
Kenya (3)	23%
India (1)	8%
Malawi (1)	8%
Nigeria (1)	8%
Rwanda (1)	8%
Tanzania (1)	8%

INTRODUCTION – PHOTOTHERAPY LIGHT

Treatment with blue light phototherapy is necessary to prevent morbidity and mortality for severe cases of neonatal jaundice. The blue light breaks down bilirubin in the blood, allowing the infant to excrete the excess bilirubin before it can accumulate and cause permanent brain damage (kernicterus) or death.

There is a dose dependent response of neonatal hyperbilirubinemia to phototherapy which depends on: (1) Duration of phototherapy; (2) Degree of irradiance given which is dependent on wavelength and type of light used; (3) the amount of body surface area irradiated; and (4) the distance of light from patient (this will vary and is based on manufacturers recommendation but is typically 10-30cm).

Phototherapy lights can also be paired with an irradiance meter so that clinicians can determine if the infant is receiving a therapeutic dose of light. Typically, optimal spectral irradiance is 25 -30microW/cm2/nm, although higher spectral irradiance of 30-35 microW/cm2/nm may be used in more severe cases. If the dose is too low, clinicians may adjust the placement of the infant, the height or output power of the light, or replace burnt out light elements.

There are many types of phototherapy lights and modalities including LED, spotlights, fluorescent blue lights, halogen lights, and phototherapy blankets. LED lights have been shown to be the safest and most efficacious for administering phototherapy, as they give off the least heat and are associated with the lowest risk of hyperthermia and dehydration; although, this sometimes comes at an increased cost [16-18].

FINAL TPP - PHOTOTHERAPY LIGHT

Table 5: Final TPP for Phototherapy Light

Final target product profile for Phototherapy Light		
**Characteristic**	**Optimal**	**Minimal**
SCOPE		
**Intended Use**	Treatment of hyperbilirubinemia in neonates	

**Target Operator**	For use in low- and middle-income countries by a wide variety of clinicians, including nurses, clinical officers, and pediatricians	
**Target Population**	Neonates (born at any gestational age and require ongoing care)	
**Target Setting**	Hospitals in low-resource settings	
SAFETY AND STANDARDS		
**Quality Management** [1]	ISO 13485:2016 Medical devices – Quality management systems -- Requirements for regulatory purposes	
**Regulation**	At least one of: CE marking, approved by US FDA or another stringent regulatory body of a founding member of IMDRF (e.g., Japan or Australia or Canada)	
TECHNICAL CHARACTERISTICS		
**Irradiance**	Standard Phototherapy: 8-10 uW/cm2/nm AND Intensive Phototherapy: >30 uW/cm2/nm	
**Effective Treatment Area**	>2000 cm2	>1300 cm2
**Peak Wavelength**	430-490 nm	
**Light Source**	LED	
**Bulb Lifetime**	60,000 hours	44,000 hours
**Ease of Replacing Bulbs**	Capable of being replaced by a technician with minimal training and basic tools (screwdrivers)	
**Irradiance Meter**	Included	Available
PURCHASING CONSIDERATIONS		
**Instrument Pricing**	<$400 ex-works	<$1,000 ex-works
UTILITY REQUIREMENTS		
**Power Source**	Mains with battery backup	Mains Power
**Battery**	Provides battery backup	None
**Voltage**	Model must match the voltage and frequency of the purchasing country's local power grid (e.g., 110-120 VAC at 60 Hz or 220-240 VAC at 50 Hz)	
TRAINING AND MAINTENANCE		

**User Instructions**	User manual and additional training materials (checklists, videos, guides) in at least one national official language for the country of intended use. Attached to device with labels and markings where possible	User manual provided in at least one national official language
**Warranty**	5 years	1 year

[1] *There was not 75% voting agreement on the Minimal characteristic. Please refer to the TPP Report discussion for additional detail.*

CONSENSUS MEETING SUMMARY: PHOTOTHERAPY LIGHT

To arrive at the final TPP for Phototherapy Light (Table 5), we conducted a pre-meeting survey to prioritize the items for discussion at the Consensus Meeting for characteristics that achieved below 75% agreement in the survey results (Table 6). An overview of the discussion at the Consensus Meeting of these characteristics is included below.

- **Effective Treatment Area**
  - Consensus was achieved in the room (without a Mentimeter vote) for the Optimal characteristic that the Effective Treatment Area would be expanded to measure >2000 cm^2 and the Minimal would be adjusted to >1300 cm^2. Clinicians emphasized the importance of expanding the Optimal Effective Treatment Area to be equal to the base size of a basinet or incubator at 2000 cm^2 even though some guidelines (e.g., AAP) specify that effective surface area is1800 cm^2 (60 x 30 cm) [70] [71]. Product developers warned against increasing the size for the purpose of using one machine for multiple babies while clinicians acknowledged that in low-resource settings, this often occurred despite knowing that this wasn't the proper use of the device. Clinicians also noted that increasing the Optimal Effective Treatment Area was necessary to accommodate larger babies ("chubby chaps") and movement ("squiggly wigglies").
  - *Optimal: >2000 cm^2*
  - *Minimal: >1300 cm^2*
- **Irradiance Meter**
  - Consensus was achieved in the room (without a Mentimeter vote) for the Minimal characteristic to be adjusted and specify that an irradiance meter is available for use but that it is not required to be bundled with every phototherapy light purchase. The concern expressed was that this would add an additional cost to the price of the phototherapy light. Clinicians and product developers agreed that an irradiance meter could be purchased separately (estimated cost between $100 - $300) or one could be shared across the unit. There was a discussion that broader guidelines/toolkits for procurement officers on the minimal infrastructure requirements should be developed so that hospitals who purchase a Phototherapy Light also ensure that an Irradiance Meter is available.
  - *Minimal: Available.*
- **Voltage**

- There was agreement in the room that all characteristics relating to Utility Requirements (e.g., Back-up Battery; Battery Power; Batteries; Voltage; Power Requirement; Maximum Power Consumption; Response During Power Outage; Surge Protection, Electrical Plug) be reviewed and harmonized following the TPP meeting.
- *Optimal and Minimal: Model must match the voltage and frequency of the purchasing country's local power grid (e.g., 110-120 VAC at 60 Hz or 220-240 VAC at 50 Hz)*

The following characteristics were not discussed at the TPP Consensus Meeting explicitly, however, additional comments were received and incorporated into the discussion:

- **Battery**
  - Participants commented that ideally, a battery back-up should be available internal to the device. Additionally, ideally the device should not be damaged by cycling of power/voltage spikes in the case of a power surge. The Optimal characteristic for Battery includes "Provides battery backup" in response to this point.

DELPHI-LIKE SURVEY: PHOTOTHERAPY LIGHT

Table 6: Delphi-like survey results for Phototherapy Light TPP prior to Consensus Meeting (data as of Oct 25, 2019)

	Optimal		Minimal		
**Characteristic**	**Optimal requirement**	**% agreement (n size)**	**Minimal requirement**	**% agreement (n size)**	**Collated comments from Delphi-like survey**
**Intended Use**	Optimal: Treatment of hyperbilirubinemia in neonates.	**100%** **n = 24**	Minimal: Same as Optimal.	**100%** **n = 22**	0 comments
**Target Operator**	Optimal: For use in low- and middle-income countries by a wide variety of clinicians, including nurses, clinical officers, and pediatricians.	**96%** **n = 23**	Minimal: Same as Optimal	**95%** **n = 22**	1 comment<br>• Technology is widely used regardless of country income
**Target Population**	Optimal: Neonates (<28 days)	**100%** **n = 23**	Minimal: Same as Optimal.	**100%** **n = 21**	1 comment<br>• Would potentially be useful up to 40 days

	Optimal		Minimal		
**Target Setting**	Optimal: Hospitals in low-resource settings	**88%**<br>**n = 24**	Minimal: Same as Optimal.	**91%**<br>**n = 22**	2 comments<br>• Technology is required regardless of country income<br>• Not necessarily low-income countries
**International Standard**	Optimal: ISO 13485:2016 Medical devices – Quality management systems -- Requirements for regulatory purposes.	**100%**<br>**n = 15**	Minimal: Same as Optimal.	**93%**<br>**n = 14**	1 comment<br>• "ISO standardizes across the board - yes, it should. If it doesn't meet the ISO standards, does that mean it is not effective? If it meets the regional standards, that would be okay, but it's preferred that it meets ISO standards."
**Regulation**	Optimal: CE marking or US FDA Clearance	**83%**<br>**n = 18**	Minimal: Same as Optimal	**88%**<br>**n = 17**	6 comments as summarized below<br>• Theme: Reduce regulatory options or add more flexibility<br>• CE Mark alone is sufficient<br>• Consider additional 'or' options:<br>o Other Stringent Regulatory Authorities – Japan or Australia or Canada<br>o Consider regulatory bodies of Low- and Middle-Income Countries
**Irradiance**	Optimal: Standard Phototherapy: 8-10 uW/cm2/nm AND Intensive Phototherapy: >30 uW/cm2/nm	**94%**<br>**n = 18**	Minimal: Same as Optimal	**94%**<br>**n = 16**	2 comments as summarized below<br>• Need clinical reference<br>• The luminous flux depend of the distance of measurement.
**Effective Treatment Area**	Optimal: >1300 cm2	**73%**<br>**n = 15**	Minimal: Same as Optimal.	**79%**<br>**n = 14**	5 comments as summarized below<br>• Needs to be bigger:<br>o 1920 cm2<br>o 1250 cm2<br>o Should cover the whole baby and baby should be naked, without pampers

	Optimal		Minimal		
					o Need clinical reference
**Peak Wavelength**<br>*(corrected from 'Pressure')*	Optimal: 430-490 nm	**100%**<br>**n = 19**	Minimal: Same as Optimal.	**100%**<br>**n = 17**	1 comment<br>• Can also be 425-475 nm
**Light Source**	Optimal: LED	**100%**<br>**n = 23**	Minimal: Same as Optimal.	**100%**<br>**n = 21**	1 comment<br>• Recommended and safer. New technology and longer life span
**Bulb Lifetime**	Optimal: 60,000 hours	**95%**<br>**n = 21**	Minimal: 44,000 hours	**89%**<br>**n = 18**	5 comments as summarized below<br>• Most manufactures have shelf life of 50,000 hours. That is a reasonable number<br>• 1,000 hours is something accepted by industry standards as minimal requirement<br>• Agree, but need clarity on if this is as reported by manufacturer or actually tested. It is assumed that irradiance levels reduce as hours increase. In my mind this spec means that at "44,000 hours" the irradiance level still meets the initial spec of >30 irradiance<br>• There is no more bulbs in the equipment we are talking about LED
**Ease of Replacing Bulbs**	Optimal: Capable of being replaced by a technician with minimal training and basic tools (screwdrivers)	**90%**<br>**n = 21**	Minimal: Same as Optimal.	**85%**<br>**n = 20**	5 comments as summarized below<br>• Theme: Remove this characteristic or change to adapt to LED<br>o Given the long duration of LED, it is expected that machines will be trashed before light expires. Hence, changing the

	Optimal		Minimal		
					light is not that essential in LED Phototherapy<br>o Recommend changing Optimal to: bulbs last lifetime of device<br>o Recommend changing minimal to: Capable of being replaced by a technician with minimal training and basic tools<br>o "LEDs bulbs should not be replaced by a technician. Bulbs will burn out at the end of life of the unit and should be returned to manufacturer. this is the replacement cycle of the devices."<br>• Very important - can't keep replacing everyday bulbs. Spare bulbs need to be available. Don't want to have to request them on a one-off basis from the US
**Irradiance Meter**	Optimal: Included	**75%**<br>**n = 20**	Minimal: Same as Optimal.	**72%**<br>**n = 18**	8 comments as summarized below<br>• Theme: Can be made available separately<br>• Theme: I can be used across whole hospital to reduce cost<br>• Change 'Included' to 'Available'
**Voltage**	Optimal: 110-240 50-60hz	**74%**<br>**n = 19**	Minimal: 220-240 50-60hz	**72%**<br>**n = 18**	8 comments as summarized below<br>• Suppliers should offer either a single unit capable of running of 110-240 volt, or have two different versions, one of 110 volt and another for 220 -240 volt.
**Response During Power Outage**	Optimal: Provides battery backup internal to device	**91%**<br>**n = 23**	Minimal: Is not damaged by cycling of power/voltage spikes	**81%**<br>**n = 21**	6 comments as summarized below<br>• Theme: Minimal is not in correct category as it does not directly relate to the Optimal. Possibly a separate spec?<br>• Recommendation to make to Characteristics

	Optimal		Minimal		
					1. Battery backup: Optimal - provides battery backup internal to device; minimal – none<br>2. Protection from power surge: Optimal - is not damaged by cycling of power/voltage spikes; minimal: none
**User Instructions**	Optimal: User manual and additional training materials (checklists, videos, guides) in English and local language. Attached to device with labels and markings where possible.	**100%**<br>**n = 23**	Minimal: User manual provided.	**95%**<br>**n = 22**	4 comments as summarized below<br>• Theme: Training materials will likely need to be developed separate from the manufacturer
**Warranty**	Optimal: >5 years	**78%**<br>**n = 23**	Minimal: ≥1 year	**82%**<br>**n = 22**	10 comments as summarized below<br>• Theme: 5 years too long<br>• Suggested Ranges: 2 years<br>To honor a 5 year warranty, you will have to have strong in-country representation. All an extended warranty is a degree of assurance of the above, and this will come at a cost. Manufactures of concentrators willing to extend a warranty from 2-5 do so at a cost. What might be more useful is that during any procurement, consideration be given to establishing a SLA with an in-country rep. In this case, you can take care of any major PPM requirements, as well as "swap out" in the event of a break-down, and there is no discussion of warranties and no need for spares and an in-country source for consumables.
**Instrument Pricing**	Optimal: <$400 ex-works	**95%**<br>**n = 20**	Minimal: <$1,000 ex-works	**75%**<br>**n = 20**	9 comments as summarized below<br>• Theme: Geography is extremely price sensitive and even $400 was viewed as the maximum<br>• Optimal is too low and may impact quality of device provided<br>• Current brands are $2,000 (may not be ex-works)<br>• $400-500 maximum

	Optimal		Minimal		
					• Too expensive<br>• Pricing ought to be reasonable for LMIC budgets and not prohibitive

Figure 7: Summary of organizational affiliation for Phototherapy Light TPP from Delphi-like Survey prior to Consensus Meeting (data as of Oct 25, 2019)

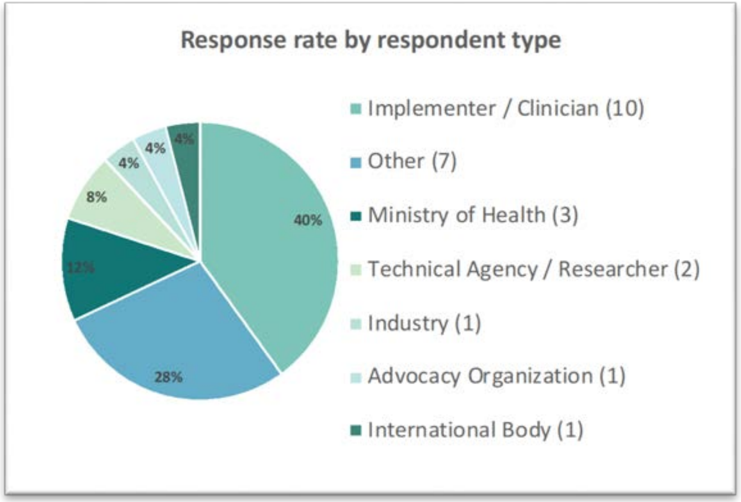

Respondent type	Percentage
Implementer / Clinician (10)	40%
Other (7)	28%
Ministry of Health (3)	12%
Technical Agency / Researcher (2)	8%
Industry (1)	4%
Advocacy Organization (1)	4%
International Body (1)	4%

Figure 8: Summary of response rate by country for Phototherapy Light TPP from Delphi-like Survey prior to Consensus Meeting (data as of Oct 25, 2019)

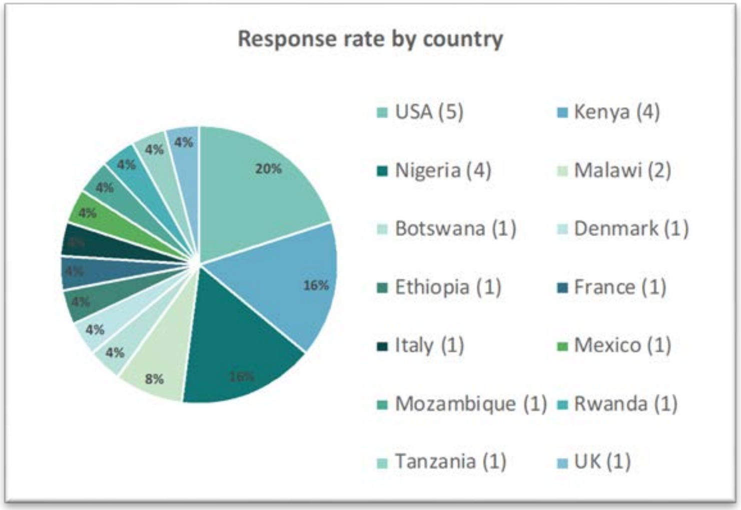

Country	Percentage
USA (5)	20%
Kenya (4)	16%
Nigeria (4)	16%
Malawi (2)	8%
Botswana (1)	4%
Denmark (1)	4%
Ethiopia (1)	4%
France (1)	4%
Italy (1)	4%
Mexico (1)	4%
Mozambique (1)	4%
Rwanda (1)	4%
Tanzania (1)	4%
UK (1)	4%

POINT-OF-CARE DIAGNOSTICS

Access to diagnostic laboratories remains a key challenge in low-resource settings [19]. Point-of-care diagnostic tests can therefore enable health-care workers to provide more rapid and effective care [20]. Simple, rapid, and affordable point-of-care tests which require minimal or no electricity, a laboratory, or highly trained staff, are now available and widely used for several common conditions in low- and middle-income countries (LMICs) [21]. These point-of-care tests offer an unprecedented opportunity to reduce inequalities in health, and to help LMICs achieve the health-related Sustainable Development Goals (SDGs) [4,11].

INTRODUCTION - GLUCOMETER

Hypoglycemia is a common metabolic problem in newborns and can result in neurologic complications if left untreated. Small and premature newborns are at increased risk for hypoglycemia. Monitoring blood glucose concentration allows clinicians to intervene with supplemental glucose for at-risk infants. Most common point-of-care glucometers are designed to be accurate at high glucose ranges for management of adult diabetes; few are intended for use or accurate in the low glucose concentrations seen in hypoglycemic newborns.

FINAL TPP - GLUCOMETER

Table 7: Final TPP for Glucometer

Final target product profile for Glucometer		
**Characteristic**	**Optimal**	**Minimal**
SCOPE		
**Intended Use**	Quantitative measurement of blood glucose for diagnosis and management of neonatal	
**Target Operator**	For use in low- and middle-income countries by a wide variety of clinicians, including nurses, clinical officers, and pediatricians	
**Target Population**	Neonates (born at any gestational age and require ongoing care)	
**Target Setting**	Hospitals in low-resource settings, but, may be used in health facilities based on country guidelines	Hospitals in low-resource settings
SAFETY AND STANDARDS		
**Quality Management** [1]	ISO 13485:2016 Medical devices – Quality management systems -- Requirements for regulatory purposes	
**Regulation**	At least one of: CE marking, approved by US FDA or another stringent regulatory body of a founding member of IMDRF (e.g., Japan or Australia or Canada)	
TECHNICAL CHARACTERISTICS		
**Linear Range**	0-50 mmol/L (0-900 mg/dL)	0-20 mmol/L (0-360 mg/dL)

**Accuracy**	± 6% across the whole range ± 0.2 mmol/L at 2.5 mmol/L (± 3.6 mg/dL at 45 mg/dL)	± 8% [2]<br>± 0.2 mmol/L at 3 mmol/L (± 3.6 mg/dL at 54 mg/dL)
**Results Format**	Quantitative across whole linear range (should be able to switch between mg and mmol)	
**Result Units**	mg/dL OR mmol/L	
**Precision**	±2% or 2.5 mg/dL, whichever is greater	
**Sample**	Whole blood heel-stick sample <5 µL	Whole blood heel-stick sample <50 µL
**Calibration**	No calibration	Minimal user calibration required
**Kit Stability & Storage**	Stable for >12 months with harsh ambient conditions (temperature 5-45 °C, humidity 15% to 95%, dusty air, elevation >=2000 meters) and transport stress (48h with fluctuations up to 50°C and down to 0°C)	Stable for 12 months with harsh ambient conditions (temperature 10-40 °C, humidity 15%-95% elevation up to 2000 meters) and transport stress (48h with fluctuations up to 50°C and down to 0°C)
**Equipment Required**	Small, portable or hand-held device; device-free/disposable preferred	Small, table-top device; portable device optional
PURCHASING CONSIDERATIONS		
**Instrument Pricing**	<$30 ex-works	
**Consumable Pricing**	$0.05 per test ex-works, ideally with generic strips	$.20 per test ex-works
UTILITY REQUIREMENTS		
**Power Source**	No power required	Mains with rechargeable battery
**Battery**	None (i.e. a disposable test that requires no electricity)	Rechargeable battery, >100 tests on a single charge.
**Voltage**	None.	Model must match the voltage and frequency of the purchasing country's local power grid (e.g., 110-120 VAC at 60 Hz or 220-240 VAC at 50 Hz)

[1] *There was not 75% voting agreement on the Minimal characteristic. Please refer to the TPP Report discussion for additional detail.*

[2] Source: https://www.westgard.com/2019-clia-changes.htm *CLIA proposed changes define Accuracy as ±8%. Current CLIA standard is* ± 6 mg/dL or ± 10% (greater). *These changes are proposed as of Feb 2019.*

CONSENSUS MEETING SUMMARY: GLUCOMETER

To arrive at the final TPP for Glucometer (Table 7), we conducted a pre-meeting survey to prioritize the items for discussion at the Consensus Meeting for characteristics that achieved below 75% agreement in the survey results (Table 8). An overview of the discussion at the Consensus Meeting of these characteristics is included below.

- **Results Format**
  - Consensus was achieved in the room (without a Mentimeter vote) for the Minimal characteristic to be the same as Optimal and to add the ability to change between mmol/L and mg/dL in both settings.
  - *Optimal: Quantitative across whole linear range (should be able to switch between mg and mmol)*
  - *Minimal: Same as Optimal*
- **Precision**
  - Consensus was achieved in the room (without a Mentimeter vote) for the Minimal characteristic to be the same as Optimal. Participants noted that the range of commercially accepted equipment is <5% CV for neonates.
  - *Optimal: ±2% or 2.5 mg/dL, whichever is greater*
  - *Minimal: Same as Optimal*
- **Instrument Pricing**
  - Consensus was achieved in the room (without a Mentimeter vote) for the Minimal and Optimal characteristic to be <$30 ex-works. Participants noted that devices exist for $20 that are approved for at-home use only, while devices approved and tested for use in sick neonates can cost $500-$900 ex-works. Given the current market gap, a research question was developed to consider pressure testing the market for off-label use of adult glucometers in neonates.
  - *Optimal: <$30 ex-works*
  - *Minimal: Same as Optimal*
- **Consumable Pricing**
  - Consensus was achieved in the room (without a Mentimeter vote) for the Minimal and Optimal characteristic. Participants emphasized the need for a generic test strip. For commercially available products labeled for neonatal use, the current ex-works price per test is roughly $1-$2.
  - *Optimal: $0.05 per test ex-works, ideally with generic strips*
  - *Minimal: $.20 per test ex-works*
- **Battery**
  - There was agreement in the room that all characteristics relating to Utility Requirements (e.g., Back-up Battery; Battery Power; Batteries; Voltage; Power Requirement; Maximum Power Consumption; Response During Power Outage; Surge Protection,

Electrical Plug) be reviewed and harmonized following the TPP meeting. In this specific case, the language used in the Optimal and Minimal characteristics were adjusted during this harmonization review following the vote.
  - *Optimal: None (i.e. a disposable test that requires no electricity)*
  - *Minimal: Rechargeable, >100 tests on a single charge*

- **Voltage**
  - There was agreement in the room that all characteristics relating to Utility Requirements (e.g., Back-up Battery; Battery Power; Batteries; Voltage; Power Requirement; Maximum Power Consumption; Response During Power Outage; Surge Protection, Electrical Plug) be reviewed and harmonized following the TPP meeting. In this specific case, the language used in the Optimal and Minimal characteristics were adjusted during this harmonization review following the vote.
  - *Optimal: None*
  - *Minimal: Model must match the voltage and frequency of the purchasing country's local power grid (e.g., 110-120 VAC at 60 Hz or 220-240 VAC at 50 Hz)*

DELPHI-LIKE SURVEY: GLUCOMETER

Table 8: Delphi-like survey results for Glucometer TPP prior to Consensus Meeting (data as of Oct 25, 2019)

	**Optimal**		**Minimal**		
**Characteristic**	**Optimal requirement**	**% agreement (n size)**	**Minimal requirement**	**% agreement (n size)**	**Collated comments from Delphi-like survey**
**Intended Use**	Optimal: Quantitative measurement of blood glucose for diagnosis and management of neonatal hypoglycemia	85%<br>n = 13	Minimal: Same as Optimal.	82%<br>n = 11	4 comments as summarized below<br>• We need this also for neonatal ***hyper***glycemia<br>• Optimal use would not be restricted to neonates<br>• Minimal use can be restricted to neonates/infants<br>• At a minimum, the device could be semi-quantitative and indicate normal - low - severely low
**Target Operator**	Optimal: For use in low- and middle-income countries by a wide variety of clinicians, including nurses, clinical officers, and pediatricians.	92%<br>n = 13	Minimal: Same as Optimal	91%<br>n = 11	3 comments as summarized below<br>• Theme: Broaden to include additional Target Operators<br>o Include nurse aides and community healthcare workers<br>o Ideally usable by patients and community health workers

	Optimal		Minimal		
**Target Population**	Optimal: Neonates (<28 days)	**77%**<br>**n = 13**	Minimal: Same as Optimal.	**73%**<br>**n = 11**	6 comments as summarized below<br>• Theme: Broaden to include additional age ranges<br>o This should be available to use in any baby - consider the KMC baby who was born at 1.2kg and is now 5 weeks old<br>o Would consider need for this over first 3 months of life, particularly for preterm/LBW babies<br>o Need for children up to 13 years<br>o Yes, but can be used in other ages too<br>o Adaptable to all levels of population
**Target Setting**	Optimal: Hospitals in low-resource settings	**77%**<br>**n = 13**	Minimal: Same as Optimal.	**73%**<br>**n = 11**	5 comments as summarized below<br>• Theme: Broaden to include additional settings<br>o This should be available both within healthcare facilities and hospitals of all levels<br>o Ideal target settings should include health posts, clinics, traditional birth attendants<br>o Sometimes the community healthcare workers need this too
**International Standard**	Optimal: ISO 13485:2016 Medical devices – Quality management systems -- Requirements for regulatory purposes.	**100%**<br>**n = 6**	Minimal: Same as Optimal.	**100%**<br>**n = 5**	0 comments
**Regulation**	Optimal: CE marking or US FDA Clearance	**100%**<br>**n = 6**	Minimal: Same as Optimal.	**100%**<br>**n = 5**	0 comments
**Linear Range**	Optimal: 0-50 mmol/L (0-900 mg/dL)	**85%**<br>**n = 13**	Minimal: 0-20 mmol/L (0-360 mg/dL)	**75%**<br>**n = 12**	2 comments<br>• Minimal: 20 mmol/L would be too low for hyperglycemia; 40 mmol/L would be better

	Optimal		Minimal		
					• Optimal range (if you're trying to pick a device that could be used outside neo unit) I understand 0-900 mg/dL though seems like anything over 500 mg/dL in peds will generally have the same management (don't know about adults)<br>• Minimal range of 0-300 mg/dL for neonates<br>• Do any actually read to 50 mmol/L?<br>• 0-600 mg/dL may be needed
**Accuracy**	Optimal: ± 0.2 mmol/L at 2.5 mmol/L (± 3.6 mg/dL at 45 mg/dL)	**77%**<br>**n = 13**	Minimal: ± 0.2 mmol/L at 3 mmol/L (± 3.6 mg/dL at 54 mg/dL)	**75%**<br>**n = 12**	4 comments as summarized below<br>• +/- 0.1 may be better<br>• +- 0.2 at entire linear range<br>• So in neonates this range of accuracy for minimal requirement seems too large? Hypoglycemia is 25-30mg/dL in a JUST BORN baby. Later on its <60 mg/dL so having a range of accuracy of 20mg/dL seems too broad? I'm also not familiar w/what the standards are for current POC vs serum glucose measurements
**Results Format**	Optimal: Quantitative across whole linear range	**100%**<br>**n = 12**	Minimal: Quantitative; semi quantitative at <2 mmol/L	**55%**<br>**n = 11**	7 comments as summarized below<br>• Minimal: In hospital, you need quantitative so you can follow up and give treatment. For home use and community it should be color coded and the actual figures<br>• Semi quantitative OK <25mg/dL<br>• Quantitative better<br>• Is sufficient to have low set at 2 mmol/L<br>• better to have quantitative across the whole range. May be < 1mmol could be semi quantitative<br>• If semi quantitative at <2 mmol/L could only be useful in the first 48-72 hours of life. Thereafter, cut-off needs to be higher

	Optimal		Minimal		
**Result Units**	Optimal: mg/dL OR mmol/L	85%<br>n = 13	Minimal: Same as Optimal.	82%<br>n = 11	2 comments summarized below<br>• mmol/L only
**Precision**	Optimal: +-2% or 2.5 mg/dL, whichever is greater	83%<br>n = 12	Minimal: 5% CV	67%<br>n = 9	5 comments as summarized below<br>• up to 2.5mg/dL seems ok, 2% seems too permissive even for Optimal?<br>• Not sure I fully understand but a precision error of 2% seems large when measuring hypoglycemia where small variants can make a significant difference<br>• convert our units
**Sample**	Optimal: whole blood heel-stick sample <5 μL	100%<br>n = 12	Minimal: whole blood heel-stick sample <50 μL	80%<br>n = 10	3 comments as summarized below<br>• Needs as small amount of blood as possible<br>• Existing glucometers require very little blood
**Calibration**	Optimal: No calibration	92%<br>n = 12	Minimal: Minimal user calibration required	91%<br>n = 11	2 comments as summarized below<br>• Need calibration<br>• Better without calibration
**Kit Stability & Storage**	Optimal: Stable for >12 months with harsh ambient conditions (temperature 5-45 °C, humidity 15% to 95%, dusty air, elevation >=2000 meters) and transport stress (48h with fluctuations up to 50°C and down to 0°C)	91%<br>n = 11	Minimal: Stable for 12 months with harsh ambient conditions (temperature 10-40 °C, humidity 15%-95% elevation up to 2000 meters) and transport stress (48h	90%<br>n = 10	2 comments as summarized below<br>• Should work in any setting / environment

	Optimal		Minimal		
			with fluctuations up to 50°C and down to 0°C)		
**Equipment Required**	Optimal: Small, portable or hand-held device; device-free/disposable preferred	**100%**<br>**n = 13**	Minimal: Small, table-top device; portable device optional	**92%**<br>**n = 12**	2 comments as summarized below<br>• Table-top too big for glucose monitoring
**Voltage**	Optimal: 110-240 50-60hz	**78%**<br>**n = 9**	Minimal: 220-240 50-60hz	**57%**<br>**n = 7**	3 comments as summarized below<br>• Should be battery operated
**Power Requirement**	Optimal: >4hr on single charge	**85%**<br>**n = 13**	Minimal: None	**75%**<br>**n = 12**	5 comments as summarized below<br>• Batteries should be rechargeable with electricity<br>• Minimal: does seem like you would need battery power option?<br>• Simple battery device which does not require electricity will be ideal<br>• Was minimal and Optimal reversed here?
**Instrument Pricing**	Optimal: <$30 ex-works	**82%**<br>**n = 11**	Minimal: <$100 ex-works	**30%**<br>**n = 10**	6 comments as summarized below<br>• Minimal: $100 seems very high<br>• A device that will cost less than what is available in the market will be ideal, the market price of the current price is around $20.<br>• This seems very high for a glucometer<br>• Good glucometers are available for $30<br>• Minimal needs to be cheaper than 100$. There are good glucometers for $10-20 on the market

	Optimal		Minimal		
**Consumable Pricing**	Optimal: $0.05 per test ex-works	**90%**<br>**n = 10**	Minimal: $1.50 per test ex-works	**33%**<br>**n = 9**	6 comments as summarized below<br>• Current state-of-the art blood glucose strips (e.g. Freestyle Lite or Bayer Contour) are around $1.00, so $1.50 seems too much<br>• $0.2 may be reasonable<br>• Minimal: current tests cost $1 or 100 KES<br>• The strip cost is more than the machine cost within six months<br>• $1.50 seems high per test<br>• The price of the glucometer itself is not so important as the cost of the strips, which can be prohibitive. Also major barrier to use is the incompatibility of many glucometer strips between different brand machines. Would be hugely beneficial to have generic strips to use on different glucometer machines

Figure 9: Summary of organizational affiliation for Glucose Test TPP from Delphi-like Survey prior to Consensus Meeting (data as of Oct 25, 2019)

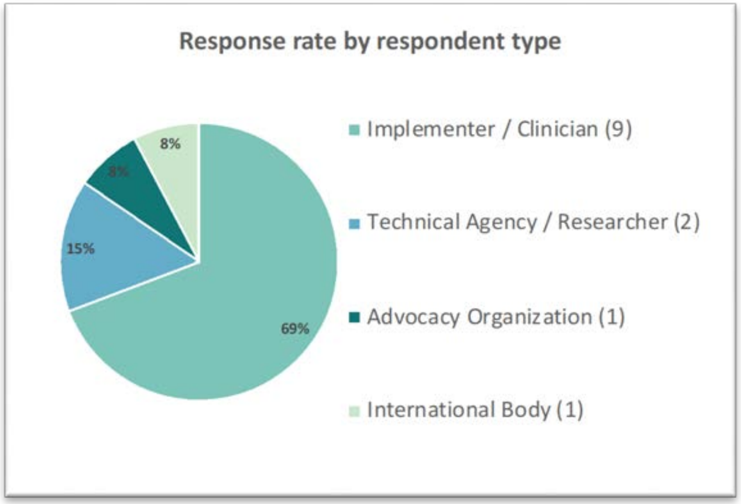

Respondent type	Percentage
Implementer / Clinician (9)	69%
Technical Agency / Researcher (2)	15%
Advocacy Organization (1)	8%
International Body (1)	8%

Figure 10: Summary of response rate by country for Glucose Test TPP from Delphi-like Survey prior to Consensus Meeting (data as of Oct 25, 2019)

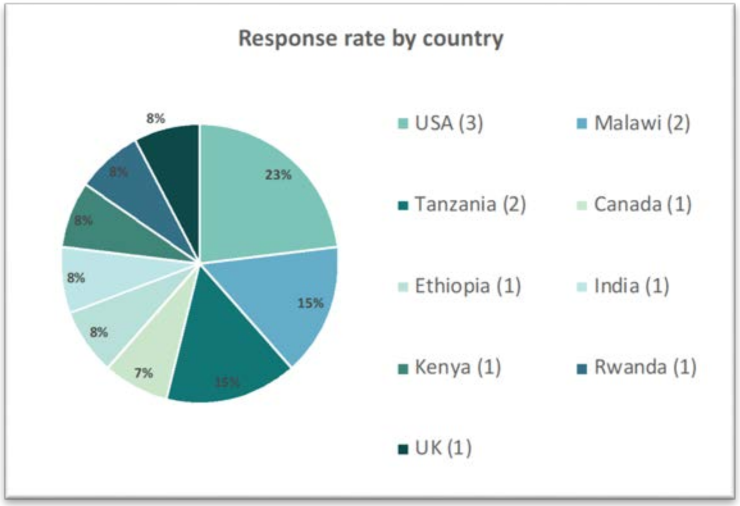

Country	Percentage
USA (3)	23%
Malawi (2)	15%
Tanzania (2)	15%
Canada (1)	8%
Ethiopia (1)	8%
India (1)	8%
Kenya (1)	8%
Rwanda (1)	8%
UK (1)	8%

INTRODUCTION: HEMOGLOBINOMETER

Hemoglobin concentration refers to the amount of the oxygen-carrying protein in the blood, and is a diagnostic for anemia (low hemoglobin) or polycythemia (high hemoglobin).

FINAL TPP: HEMOGLOBINOMETER

Table 9: Final TPP for Hemoglobinometer

Final target product profile for Hemoglobinometer		
**Characteristic**	**Optimal**	**Minimal**
SCOPE		
**Intended Use**	Quantitative determination of hemoglobin in capillary, venous, or arterial whole blood	
**Target Operator**	For use in low- and middle-income countries by a wide variety of clinicians, including nurses, clinical officers, and pediatricians	
**Target Population**	Neonates (born at any gestational age and require ongoing care)	
**Target Setting**	Hospitals in low-resource settings	
SAFETY AND STANDARDS		
**Quality Management** [1]	ISO 13485:2016 Medical devices – Quality management systems -- Requirements for regulatory purposes	
**Regulation**	At least one of: CE marking, approved by US FDA or another stringent regulatory body of a founding member of IMDRF (e.g., Japan or Australia or Canada)	
TECHNICAL CHARACTERISTICS		
**Linear Range**	0-25 g/dL	4.5-25 g/dL.
**Accuracy**	± 1 g/dL [2]	± 1.75 g/dL [2]
**Results Format**	Quantitative across whole linear range	Quantitative; semi quantitative below 5 or above 25 g/dL

**Result Units**	g/dL OR g/L	
**Precision**	1.5% CV	2% CV
**Sample**	Whole blood heel-stick sample <10 μL	Whole blood heel-stick sample <25 μL
**Number of Steps**	No more than 1-3 steps (requiring operator intervention)	No more than 4-6 steps (requiring operator intervention)
**Calibration**	No calibration	Minimal user calibration required
**Kit Stability & Storage**	Stable for >12 months with harsh ambient conditions (temperature 5-45 °C, humidity 15% to 95%, dusty air, elevation >=2000 meters) and transport stress (48h with fluctuations up to 50°C and down to 0°C)	Stable for 12 months with harsh ambient conditions (temperature 10-40 °C, humidity 15%-95% elevation up to 2000 meters) and transport stress (48h with fluctuations up to 50°C and down to 0°C)
**Equipment Required**	Small, portable or hand-held device; device-free/disposable preferred	Small, table-top device; portable device optional
PURCHASING CONSIDERATIONS		
**Instrument Pricing**	<$200 ex-works	<$300 ex-works
**Consumable Pricing**	$0.05 per test ex-works	$0.50 per test ex-works
UTILITY REQUIREMENTS		
**Power Source**	No power required	Mains with rechargeable battery
**Battery**	None (i.e. a disposable test that requires no electricity)	Rechargeable battery, >100 tests on a single charge.
**Voltage**	None	Model must match the voltage and frequency of the purchasing country's local power grid (e.g., 110-120 VAC at 60 Hz or 220-240 VAC at 50 Hz)

[1] There was not 75% voting agreement on the Minimal characteristic. Please refer to the TPP Report discussion for additional detail.

[2] Source: https://www.westgard.com/2019-clia-changes.htm; CLIA proposed changes define Accuracy as ±4%. Current CLIA standard is ± 7%. These changes are proposed as of Feb 2019.

CONSENSUS MEETING SUMMARY: HEMOGLOBINOMETER

To arrive at the final TPP for Hemoglobinometer (Table 9), we conducted a pre-meeting survey to prioritize the items for discussion at the Consensus Meeting for characteristics that achieved below 75% agreement in the survey results (Table 10). An overview of the discussion at the Consensus Meeting of these characteristics is included below.

- **Linear Range**
  - Consensus was achieved in the room (without a Mentimeter vote) for the Optimal and Minimal characteristic. Clinicians noted that 25 g/dL was appropriate for the upper range. For the lower range, clinicians were comfortable with a reading that accurately goes down to 4.5. One participant commented that the lowest reported levels of hemoglobin concentrations measured in blood was 0.6 g/dL [22]. Product developers noted that from a technical perspective, the incremental price to adjust the measurement range is dependent on the type of test. For example, it can be more challenging to get a wider range with a non-invasive test. Furthermore, participants commented that much less expensive tests can go down to 4-5 g/dL while more expensive tests are 0-25 g/dL.
  - *Optimal: 0-25 g/dL*
  - *Minimal: 4.5-25 g/dL.*
- **Instrument Pricing**
  - Consensus was achieved in the room on the Minimal Instrument Pricing. Participants commented that less expensive tests currently exist for $100-$200, however, there is a wide range with more expensive ones at $800-$900 in price. Participants expressed concern over signaling the market with too high of a price and a vote was conducted in the room where the Minimal was agreed at $300.
  - *Minimal: Ex-works Instrument Price of $300 vs. $400*
    - Overall Vote - 73% voted "$300" (n = 11)
    - Clinicians - 80% voted "$300" (n = 10)
    - Excluding involvement with product development - 73% voted "$300" (n = 11)

DELPHI-LIKE SURVEY: HEMOGLOBINOMETER

Table 10: Delphi-like survey results for Hemoglobinometer TPP prior to Consensus Meeting (data as of Oct 25, 2019)

	Optimal		Minimal		
**Characteristic**	**Optimal requirement**	**% agreement (n size)**	**Minimal requirement**	**% agreement (n size)**	**Collated comments from Delphi-like survey**

	Optimal		Minimal		
**Intended Use**	Optimal: Quantitative determination of hemoglobin in capillary, venous, or arterial whole blood.	**86%**<br>**n = 7**	Minimal: Same as Optimal.	**83%**<br>**n = 6**	2 comments as summarized below<br>• Optimally this would also give WBC and a neutrophil % to risk stratify for sepsis<br>• Is this only measuring Hb? Not that it's bourne out to be great (but it's certainly better than nothing) it seems like we would also want this instrument to get WBC and maybe a neutrophil count? Unless you're on envisioning the utility of this as a rapid diagnostic for anemia NOT generalizable to use in sepsis (where rapid Hb assessment to determine need for transfusion is also important)<br>• Minimal would be capillary whole blood
**Target Operator**	Optimal: For use in low- and middle-income countries by a wide variety of clinicians, including nurses, clinical officers, and pediatricians.	**86%**<br>**n = 7**	Minimal: Same as Optimal	**100%**<br>**n = 6**	2 comments as summarized below<br>• With non-invasive it is no longer necessary for trained phlebotomists to take measurements<br>• Optimal would be usable by community health workers as well
**Target Population**	Optimal: Neonates (<28 days)	**57%**<br>**n = 7**	Minimal: Same as Optimal.	**80%**<br>**n = 5**	4 comments as summarized below<br>• Theme: Broaden the Target Population<br>o Ideally this could be used for infants, children and adults as well (not sure if it has to be specific to neonates because of HbF)<br>o Required for other infants as well<br>o Can be used across all ages<br>o Optimal would be for neonates AND older infants and children
**Target Setting**	Optimal: Hospitals in low-resource settings	**86%**<br>**n = 7**	Minimal: Same as Optimal.	**100%**<br>**n = 6**	2 comments as summarized below<br>• Target setting should include health posts and clinics in LMIC as many patients won't have access to a hospital

	Optimal		Minimal		
**International Standard**	Optimal: ISO 13485:2016 Medical devices – Quality management systems -- Requirements for regulatory purposes.	**100%** **n = 3**	Minimal: Same as Optimal.	**100%** **n = 2**	0 comments
**Regulation**	Optimal: CE marking or US FDA Clearance	**100%** **n = 3**	Minimal: Same as Optimal.	**100%** **n = 2**	0 comments
**Linear Range**	Optimal: 0-25 g/dL	**86%** **n = 7**	Minimal: Same as Optimal.	**67%** **n = 6**	2 comments as summarized below<br>• Why is the range so high? 0-20 or even 2-20 seems more meaningful?<br>• I question anything above 17 as necessary
**Accuracy**	Optimal: +-1 g/dL	**83%** **n = 6**	Minimal: +-1.75 g/dL	**80%** **n = 5**	2 comments as summarized below<br>• Range of 3.5g/dL seems high to me?<br>• Way too strict, propose to update Optimal 7% and Minimal to 15%<br>• CLIA standards are 7%
**Results Format**	Optimal: Quantitative across whole linear range	**100%** **n = 6**	Minimal: Quantitative; semi quantitative below 5 or above 25 g/dL	**80%** **n = 5**	2 comments as summarized below<br>• I'd change minimal to "above 20"<br>• For neonates the transfusion threshold would be higher than 5 g/dL so that threshold seems too low in that age group (would be closer to 7.5-8.5, or even higher in the first week of life). Even for older children a higher value around 7 might be more appropriate
**Result Units**	Optimal: g/dL OR g/L	**100%** **n = 7**	Minimal: Same as Optimal.	**83%** **n = 6**	1 comment<br>• At a minimum, one of the units could be displayed, together with a conversion chart that comes with the machine

	Optimal		Minimal		
**Precision**	Optimal: 1.5% CV	**83%**<br>**n = 6**	Minimal: 2% CV	**80%**<br>**n = 5**	2 comments<br>• Theme: do not understand Characteristic
**Sample**	Optimal: whole blood heel-stick sample <10 µL	**100%**<br>**n = 6**	Minimal: whole blood heel-stick sample <25 µL	**100%**<br>**n = 5**	1 comment<br>• Noninvasive may be a first line measure prior to taking a blood draw
**Number of Steps**	Optimal: No more than 1-3 steps (requiring operator intervention)	**100%**<br>**n = 6**	Minimal: No more than 4-6 steps (requiring operator intervention)	**80%**<br>**n = 5**	2 comments<br>• Above 4 steps gets complicated<br>• Too many steps
**Calibration**	Optimal: No calibration	**100%**<br>**n = 7**	Minimal: Minimal user calibration required	**83%**<br>**n = 6**	2 comments<br>• There is significant drift in devices if they are not calibrated. Anything requiring a blood sample should be calibrated prior to the measurement. There are huge questions about the validity of global hemoglobin data from DHS for this (and other) reasons. Noninvasive devices require minimum to no calibration<br>• Preferably without calibration
**Kit Stability & Storage**	Optimal: Stable for >12 months with harsh ambient conditions (temperature 5-45 °C, humidity 15% to 95%, dusty air, elevation >=2000 meters) and transport stress (48h with fluctuations up to 50°C and down to 0°C)	**100%**<br>**n = 6**	Minimal: Stable for 12 months with harsh ambient conditions (temperature 10-40 °C, humidity 15%-95% elevation	**100%**<br>**n = 6**	0 comments

	Optimal		Minimal		
			up to 2000 meters) and transport stress (48h with fluctuations up to 50°C and down to 0°C)		
**Equipment Required**	Optimal: Small, portable or hand-held device; device-free/disposable preferred	100%<br>n = 7	Minimal: Small, table-top device; portable device optional	100%<br>n = 6	0 comments
**Power Requirement**	Optimal: None (i.e. a disposable test that requires no electricity)	100%<br>n = 6	Minimal: 110-220V AC current; DC power with rechargeable battery lasting up to 8 hours of testing	86%<br>n = 7	2 comments<br>• I question whether disposable tests have the accuracy required<br>• Solar power would be best if an energy source is needed and might be better than a disposable test to avoid bio-hazardous trash
**Instrument Pricing**	Optimal: <$200 ex-works	83%<br>n = 6	Minimal: <$800 ex-works	60%<br>n = 5	2 comments<br>• Still expensive for most LIC where the test may be highly required<br>• < $100 would be better
**Consumable Pricing**	Optimal: $0.05 per test ex-works	100%<br>n = 5	Minimal: $0.50 per test ex-works	80%<br>n = 5	2 comments<br>• Varies depending on what equipment is being used<br>• Too expensive for hemoglobin

Figure 11: Summary of organizational affiliation for Hemoglobin Test TPP from Delphi-like Survey prior to Consensus Meeting (data as of Oct 25, 2019)

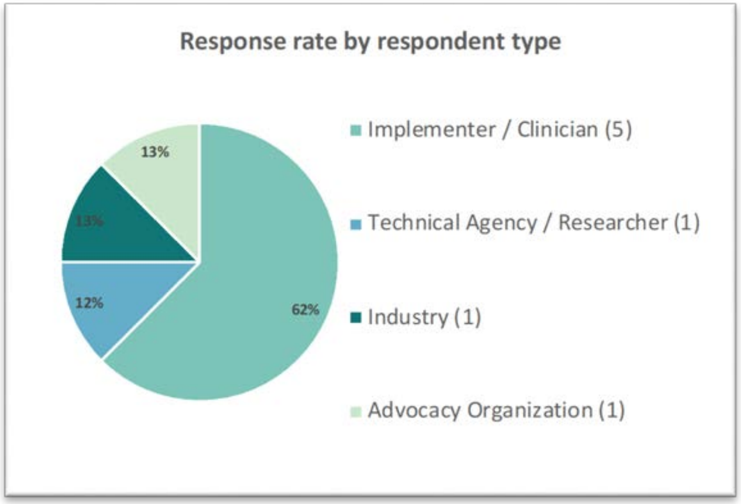

Respondent type	Percentage
Implementer / Clinician (5)	63%
Technical Agency / Researcher (1)	13%
Industry (1)	13%
Advocacy Organization (1)	13%

Figure 12: Summary of response rate by country for Hemoglobin Test TPP from Delphi-like Survey prior to Consensus Meeting (data as of Oct 25, 2019)

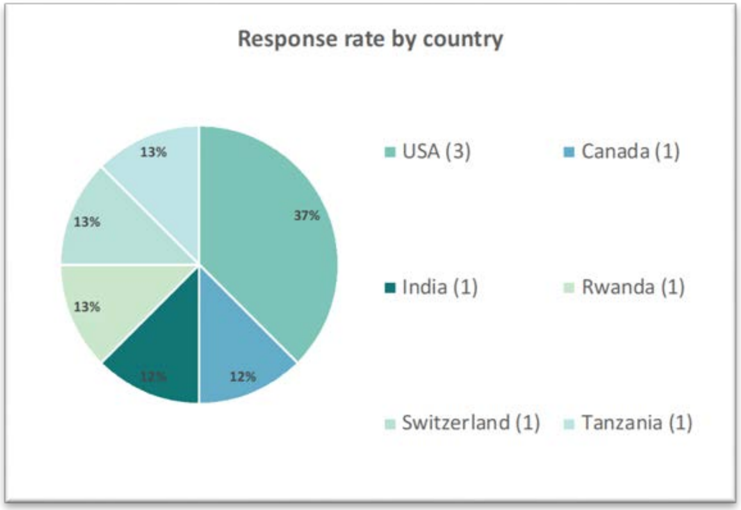

Country	Percentage
USA (3)	38%
Canada (1)	13%
India (1)	13%
Rwanda (1)	13%
Switzerland (1)	13%
Tanzania (1)	13%

INTRODUCTION: PH MONITOR

pH is an important blood gas measurement that assesses the acid-base status of the blood. pH can be assessed on arterial cord blood as well as peripheral arterial, venous, and capillary blood and, when interpreted with other tests and clinical conditions, provide information on the status of the neonate. Although clinically relevant pH values vary by condition, postnatal age (in minutes/hours), and type of blood sample (i.e., venous, arterial, etc.), pH values below 7.4 can indicate acidosis, which can be either metabolic, respiratory, or mixed. In the newborn setting, blood gas analysis is typically employed in an intensive care setting and can be utilized to augment management of invasive and non-invasive positive pressure respiratory support, sepsis, and perinatal asphyxia. To differentiate between the different types of acidosis, it is necessary to measure not only pH but also pCO_2, pO_2, and base excess.

FINAL TPP: PH MONITOR

Table 11: Final TPP for pH Monitor (not discussed at Consensus Meeting)

Final target product profile for pH Monitor		
**Characteristic**	**Optimal**	**Minimal**
SCOPE		
**Intended Use**	Quantitative measurement of pH for diagnosis and management of metabolic acidosis	
**Target Operator**	For use in low- and middle-income countries by a wide variety of clinicians, including nurses, clinical officers, and pediatricians	
**Target Population**	Neonates (born at any gestational age and require ongoing care)	
**Target Setting**	Hospitals in low-resource settings	
SAFETY AND STANDARDS		
**Quality Management** [1]	ISO 13485:2016 Medical devices – Quality management systems -- Requirements for regulatory purposes	
**Regulation**	At least one of: CE marking, approved by US FDA or another stringent regulatory body of a founding member of IMDRF (e.g., Japan or Australia or Canada)	

TECHNICAL CHARACTERISTICS		
**Linear Range**	6.5-8.2	6.9-7.45
**Accuracy**	± 0.04 [2]	
**Precision**	± 0.01	
**Sample**	Whole blood heel-stick sample <5 µL	Whole blood heel-stick sample <50 µL
**Results Format**	Quantitative	
**Calibration**	No calibration	Minimal user calibration required
**Kit Stability & Storage**	Stable for >12 months with harsh ambient conditions (temperature 5-45 °C, humidity 15% to 95%, dusty air, elevation >=2000 meters) and transport stress (48h with fluctuations up to 50°C and down to 0°C)	Stable for 12 months with harsh ambient conditions (temperature 10-40 °C, humidity 15%-95% elevation up to 2000 meters) and transport stress (48h with fluctuations up to 50°C and down to 0°C)
**Equipment Required**	Small, portable or hand-held device; device-free/disposable preferred	Small, table-top device; portable device optional
**Time to Result**	<3 seconds	<2 minutes
PURCHASING CONSIDERATIONS		
**Instrument Pricing**	<$30 ex-works	<$100 ex-works
**Consumable Pricing**	$0.05 per test ex-works	$1.50 per test ex-works
UTILITY REQUIREMENTS		
**Power Source**	No power required	Mains with rechargeable battery
**Battery**	None (i.e. a disposable test that requires no electricity)	Rechargeable battery, >100 tests on a single charge.
**Voltage**	None.	Model must match the voltage and frequency of the purchasing country's local power grid (e.g., 110-120 VAC at 60 Hz or 220-240 VAC at 50 Hz)

[1] *There was not 75% voting agreement on the Minimal characteristic. Please refer to the TPP Report discussion for additional detail.*

[2] Source: *https://www.westgard.com/2019-clia-changes.htm CLIA proposed changes define Accuracy as* ± 0.04 which is the same as the current standard for *Blood gas pH. These changes are proposed as of Feb 2019.*

CONSENSUS MEETING SUMMARY: PH MONITOR

To arrive at the final TPP for pH Monitor (Table 11), we conducted a pre-meeting survey to prioritize the items for discussion at the Consensus Meeting for characteristics that achieved below 75% agreement in the survey results (Table 12). Given restrictions on timing, we were not able to discuss any of the characteristics for pH Monitor at the Consensus Meeting. Please note that the number of participants in the pre-meeting survey is low.

DELPHI-LIKE SURVEY: PH MONITOR

Table 12: Delphi-like survey results for pH Monitor TPP prior to Consensus Meeting (data as of Oct 25, 2019)

	**Optimal**		**Minimal**		
**Characteristic**	**Optimal requirement**	**% agreement (n size)**	**Minimal requirement**	**% agreement (n size)**	**Collated comments from Delphi-like survey**
**Intended Use**	Optimal: Quantitative measurement of pH for diagnosis and management of metabolic acidosis and/or respiratory acidosis.	**67%**<br>**n = 6**	Minimal: Same as Optimal.	**60%**<br>**n = 5**	2 comments as summarized below<br>• Minimal / Optimal: pH on its own is not very useful; it won't help me identify respiratory vs. metabolic acidosis; would help you identify that the baby is acidotic but I need to know more<br>• Measurement of just the pH may not be as useful as having additional pO2, pCO2 and HCO3 also being made available along with pH. Interpretation of pH requires these other parameters as well
**Target Operator**	Optimal: For use in low- and middle-income countries by a wide variety of clinicians, including nurses, clinical officers, and pediatricians.	**83%**<br>**n = 6**	Minimal: Same as Optimal	**80%**<br>**n = 5**	0 comments

	Optimal		Minimal		
**Target Population**	Optimal: Neonates (<28 days)	**83%**<br>**n = 6**	Minimal: Same as Optimal.	**80%**<br>**n = 5**	1 comment<br>• Can be used in older ages as well
**Target Setting**	Optimal: Hospitals in low-resource settings	**83%**<br>**n = 6**	Minimal: Same as Optimal.	**80%**<br>**n = 5**	0 comments
**International Standard**	Optimal: ISO 13485:2016 Medical devices – Quality management systems -- Requirements for regulatory purposes.	**80%**<br>**n = 5**	Minimal: Same as Optimal.	**75%**<br>**n = 4**	0 comments
**Regulation**	Optimal: CE marking or US FDA Clearance	**80%**<br>**n = 5**	Minimal: Same as Optimal.	**75%**<br>**n = 4**	0 comments
**Linear Range**	Optimal: 6.5-8.2	**83%**<br>**n = 6**	Minimal: 6.9-7.45	**60%**<br>**n = 5**	2 comments as summarized below<br>• These ranges would/could change if intended use changes<br>• Insufficient range
**Accuracy**	Optimal: ± 0.04	**100%**<br>**n = 6**	Minimal: Same as Optimal.	**100%**<br>**n = 5**	0 comments
**Precision**	Optimal: +-0.01	**100%**<br>**n = 5**	Minimal: Same as Optimal.	**100%**<br>**n = 6**	0 comments
**Sample**	Optimal: whole blood heel-stick sample <5 µL	**83%**<br>**n = 6**	Minimal: whole blood	**40%**<br>**n = 5**	1 comment<br>• Suggest updating to include umbilical cord blood sample

	Optimal		Minimal		
			heel-stick sample <50 µL		o Optimal: whole blood heel-stick sample or umbilical cord whole blood sample<br>o Minimal: whole blood heel-stick
**Results Format**	Optimal: Quantitative	**100%**<br>**n = 6**	Minimal: Same as Optimal.	**100%**<br>**n = 5**	0 comments
**Calibration**	Optimal: No calibration	**100%**<br>**n = 6**	Minimal: Minimal user calibration required	**60%**<br>**n = 5**	1 comment<br>• Better without calibration
**Kit Stability & Storage**	Optimal: Stable for >12 months with harsh ambient conditions (temperature 5-45 °C, humidity 15% to 95%, dusty air, elevation >=2000 meters) and transport stress (48h with fluctuations up to 50°C and down to 0°C)	**100%**<br>**n = 5**	Minimal: Stable for 12 months with harsh ambient conditions (temperature 10-40 °C, humidity 15%-95% elevation up to 2000 meters) and transport stress (48h with fluctuations up to 50°C and down to 0°C)	**100%**<br>**n = 4**	0 comments
**Equipment Required**	Optimal: Small, portable or hand-held device; device-	**100%**<br>**n = 6**	Minimal: Small, table-top device; portable	**100%**<br>**n = 5**	0 comments

	Optimal		Minimal		
	free/disposable preferred		device optional		
**Voltage**	Optimal: 110-240 50-60hz	**100%** **n = 5**	Minimal: 220-240 50-60hz	**100%** **n = 4**	0 comments
**Power Requirement**	Optimal: >4hr on single charge	**100%** **n = 6**	Minimal: None	**80%** **n = 5**	1 comment<br>• Needs battery back up
**Time to Result**	Optimal: <3 seconds	**100%** **n = 6**	Minimal: <2 minutes	**100%** **n = 5**	0 comments
**Instrument Pricing**	Optimal: <$30 ex-works	**83%** **n = 6**	Minimal: <$100 ex-works	**60%** **n = 5**	2 comments<br>• Minimal: just pH on its own is not useful.<br>• Just knowing the pH is of limited value. A combination with pCO2/pO2 and HCO3 at least would be needed.
**Consumable Pricing**	Optimal: $0.05 per test ex-works	**100%** **n = 6**	Minimal: $1.50 per test ex-works	**80%** **n = 5**	0 comments

Figure 11: Summary of organizational affiliation for pH Test TPP from Delphi-like Survey prior to Consensus Meeting (data as of Oct 25, 2019)

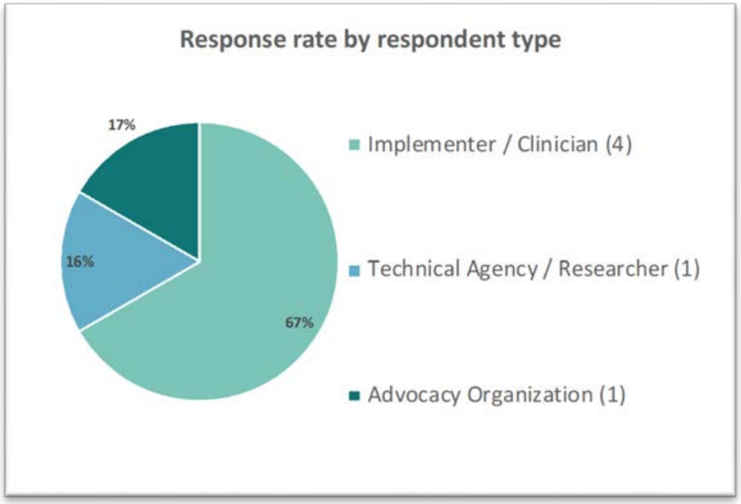

Respondent type	Percentage
Implementer / Clinician (4)	67%
Technical Agency / Researcher (1)	17%
Advocacy Organization (1)	17%

Figure 12: Summary of response rate by country for pH Test TPP from Delphi-like Survey prior to Consensus Meeting (data as of Oct 25, 2019)

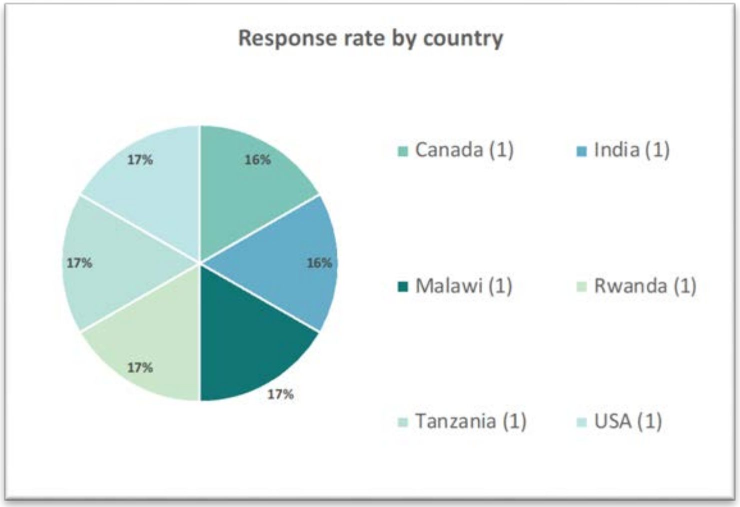

Country	Percentage
Canada (1)	17%
India (1)	17%
Malawi (1)	17%
Rwanda (1)	17%
Tanzania (1)	17%
USA (1)	17%

INFECTION PREVENTION AND CONTROL

Neonatal infections are more common where there is limited access to basic health services and where good hygiene practices are lacking [23]. The most important protective interventions for nosocomial infections are frequent hand-washing, exclusive breastfeeding and facility cleanliness [24,25], but widespread implementation of these interventions is challenging in low-resource settings. Infants (and their mothers) who are malnourished or have a chronic illness are at risk of infection because of immunosuppression and a susceptibility to preterm birth [26]. Premature infants have an increased risk of infection, regardless of the mother's antibody status [11,12].

INTRODUCTION: SEPSIS DIAGNOSTIC

Neonatal sepsis is a major cause of newborn mortality and must be identified and treated quickly to ensure survival and minimize morbidity. However, it is not easy to diagnose. Due to the immaturity of a neonatal immune systems, natural history of late deterioration, and high morbidity in the presence of a serious bacterial infection, the standard of care in neonates is to treat while simultaneously screening for sepsis with blood, urine, and spinal fluid cultures and microscopy until studies suggest that infection is unlikely to be present. There are some useful guidelines that help to identify neonates and young infants at risk of sepsis and guide clinical management. However, even when these guidelines are used, many more babies receive antibiotics than those who truly have serious bacterial infections and need antibiotics [27].

Serious bacterial infections can be identified by clinical assessment, biochemically (with biomarkers), or microbiologically. However, limited availability of microbiological diagnostic testing in low and middle income countries (LMIC) is a major barrier to safe antibiotic use and shortening courses of treatment. The currently available diagnostic tests have significant barriers in their use and interpretation [28]. Additionally, there is currently no accepted biomarker for use in low- and middle-income countries [29]. The availability, cost, rapidity of results, sensitivity, specificity, predictive value, and the interpretation of results pose challenges for the widespread use of biomarkers. Small studies have described hundreds of biomarkers associated with severe neonatal infections and biomarkers, alone or in combination, that have been used to identify newborn infections: procalcitonin (PCT), C-reactive protein (CRP), tumor necrosis factor-a (TNF-a), interferon-g (IFN-g), interleukin-6 (IL-6), interleukin (IL-8) [30]. The majority of these studies have evaluated biomarkers in combination with C-reactive protein (CRP), already in widespread clinical use for the diagnosis of infection. As an acute-phase reactant, CRP alone is less useful in the earliest phases of severe neonatal infection because it does not peak until 12 to 24 hours after infection and can also be triggered by a non-infectious insult, such as trauma.

CONSENSUS MEETING SUMMARY: SEPSIS DIAGNOSTIC

It was clear in the time available for group discussion at the Consensus meeting that further analysis and consultation will be required to formulate a TPP, particularly to delineate the practicality and clinical impact of each use case. Give this, a data-based analysis of potential use cases and further survey process are planned.

The process of developing a TPP for a point-of-care test (POCT) for neonatal possible serious bacterial presented distinct challenges. This is largely because point-of-care testing for neonatal infections is not a currently used diagnostic strategy in clinical practice, in both low-, middle-, and high-income settings. There is thus no similar technology routinely used from which the basics of development considerations and implementation measures can be used as learning points for target product profile development specific for wider use across other settings.

To begin the process of developing a TPP, we worked in partnership with Dr. Naomi Spotswood at the Burnet Institute and Dr. David Goldfarb from the University of British Columbia to develop Use Cases for potential Sepsis Diagnostics. In the first stage, six potential use cases were developed to describe the more likely clinical scenarios where a POCT for neonatal possible serious infections might be used. The first four of these were for scenarios to assist healthcare workers to decide if antimicrobials should start, the fifth to decide if antimicrobials should stop, and the sixth to identify infections with antimicrobial resistant pathogens. Initial discussion in the consensus meeting focussed on whether the first four use cases (starting antimicrobials) could be condensed into one use case. While collapsing use cases one to four into a single use case may be simpler conceptually, it was noted that each use case would have different microbiology, immunology and epidemiology, each of which will affect the pre-test probability of infection in the target population. Further, clinical thresholds for starting antimicrobials for the same use case may differ between settings. Overall it was agreed that reducing unnecessary antimicrobial use would be a key attribute of a neonatal sepsis POCT.

Moving forward, relevant questions are below. Each would ideally be estimated for the setting of interest. 1(c) and 2(c) require pre-defined target sensitivity and specificity:

1. For the first four use cases:
    a. How frequently are neonates evaluated for possible serious bacterial infections?
    b. What is the frequency of confirmed serious bacterial infection?
    c. Based on 1(a) and 1(b), how many antimicrobial courses could be avoided with use of a POCT?
2. For use case five:
    a. How frequently do hospitalised neonates receive antimicrobials?
    b. Amongst these neonates, what is the frequency of confirmed serious bacterial infection?
    c. Based on 2(a) and 2(b), how much excess antimicrobial exposure could be avoided with use of a POCT?
3. For each of use cases one to five, what is the frequency of confirmed infection with a pathogen resistant to first line antimicrobials?

The next steps for the Sepsis Diagnostic TPP are to:

- Conduct an analysis of currently available data to provide estimates for the above questions. This will allow clearer evaluation of the potential clinical impact of a POCT for each use case.
- Formulate and distribute an extended survey to finalise the TPP for a neonatal sepsis POCT. This is planned to reach beyond the original group: the WHO possible Serious Bacterial Infections Community of Practice group and Medicins Sans Frontiers have been identified as examples of groups to contact given their practical knowledge and experience relevant to this process.
- Given the wide relevance of a TPP for a neonatal sepsis point-of-care test, the group will consider publication of the TPP development process and final results in a peer reviewed journal.

USE CASE SURVEY: SEPSIS DIAGNOSTIC

Sepsis (serious infection) in neonates and young infants is devastating for many babies and their families around the world. It is also not easy to diagnose. There are some useful guidelines that help to identify neonates and young infants at risk of sepsis, and guide clinical management. However, even when these guidelines are used, many more babies receive antibiotics than those who truly have serious bacterial infections which need antibiotics [31].

Researchers around the world are trying to develop a point-of-care test for sepsis. This is a test that can be done by any healthcare worker with a quick result. However, a point-of-care test for sepsis could be used in a number of ways, and it is important that researchers know which way (a 'Use Case') will be most helpful to healthcare workers. The following six 'Use Cases' were presented in a survey. The purpose of the survey was to evaluate which of these 'Use Cases' would be of most practical benefit to clinicians who manage neonates with possible serious bacterial infections. The aim is that a test like this would be used in combination with existing guidelines provided by the World Health Organization [27].

Use Case 1. Start Antibiotics - Community Referral: A test that can be used when a baby first comes to a health facility from the community for assessment, and has one or more signs of possible serious bacterial infection. Examples of these include respiratory rate >60 breaths per minute, being unable to breastfeed, or deep jaundice. The test is to help the healthcare worker decide if they should start antibiotics. If the test is positive, this means that the baby is likely to have a serious bacterial infection. The baby needs antibiotics and supportive care. If a blood culture can be sent, this should be collected before the antibiotics are started. If the test is negative, this means the baby is highly unlikely to have a serious bacterial infection. Instead they need careful observation, and the healthcare worker should consider other reasons for their illness.

Use Case 2. Start Antibiotics - Well Baby with Risk Factors at Birth: A test that can be used when an otherwise well baby has been born with risk factors for sepsis. Examples of these risk factors are fever in the mother during labour, prolonged

rupture of the membranes (>18 hours), or foul-smelling amniotic fluid. Other non-maternal risk factors might include preterm labour. The test is to help the healthcare worker decide if they should start antibiotics. If the test is positive, this means that the baby is likely to have a serious bacterial infection. The baby needs antibiotics and supportive care. If a blood culture can be sent, this should be collected before the antibiotics are started. If the test is negative, this means the baby is highly unlikely to have a serious bacterial infection. The baby would stay with mother and receive normal newborn care.

Use Case 3. Start Antibiotics - Unwell at Birth: A test that can be used when a baby has been born with signs of sepsis with or without maternal risk factors. Signs of sepsis include tachypnea, temperature instability, or tachycardia. The test is to help the healthcare worker decide if they should start antibiotics.

If the test is positive, this means the baby is likely to have a serious bacterial infection. The baby needs antibiotics and supportive care. If a blood culture can be sent, the sample should be collected before the antibiotics are started.

If the test is negative, this means the baby is highly unlikely to have a serious bacterial infection. If the baby remains unwell, they need careful observation, and the healthcare worker should consider other reasons for their illness.

Use Case 4. Start Antibiotics - Small or Premature Baby who becomes Unwell: A test that can be used for a baby who is already admitted to a health facility because they are small or premature who becomes unwell and has one or more signs of a possible serious bacterial infection. The test is to help the healthcare worker decide if they should start antibiotics. If the test is positive, this means that the baby is likely to have a serious bacterial infection. The baby needs antibiotics and supportive care. If a blood culture can be sent, this should be collected before the antibiotics are started. If the test is negative, this means the baby is highly unlikely to have a serious bacterial infection. Instead they need careful observation, and the healthcare worker should consider other reasons for their illness.

Use Case 5. Stop Antibiotics: A test that can be used for a baby who is already admitted to a health facility and who has already received at least one day of antibiotics for a possible serious bacterial infection. The test is to help the healthcare worker decide if the antibiotics can stop. If the test is positive this means that the baby is likely to have a serious bacterial infection. The baby needs to continue their antibiotics. If there are positive blood or cerebrospinal fluid culture results, the antibiotics may need to change to make sure they are the best antibiotic to treat the infection that has been identified. If the test is negative, this means that the baby is highly unlikely to have a serious bacterial infection. The antibiotics can stop. If the baby is still unwell, the healthcare worker should consider other reasons for their illness.

Use Case 6. Resistance: A test that can be used for a baby who is already admitted to a district health facility, has already commenced antibiotics, and remains unwell. The test is to tell the healthcare worker if the baby has an infection resistant to first line (the usual) antibiotics. If the test is positive this means the baby is highly likely to have a serious bacterial infection which is resistant to the first line antibiotics which are usually started. The baby needs a different antibiotic. The test may provide some information which guides the choice of this antibiotic. If the test is negative, this means that either the baby does

not have a serious bacterial infection, or that the infection is being appropriately treated by the first line antibiotics which are usually started.

A survey with the six use cases was completed by 33 respondents (see Figures 13 and 14). Respondents were asked questions to prioritize and rank the use cases (see Figure 15). Based on the results presented below, use case 1 and 5 received the highest score despite a wide range (see Table 13 and 14).

Table 13: Initial Use Case Survey

	Prioritization Score [1]		Rank Score [2]	
	Average	Range	Average	Range
Use Case 1. Start Antibiotics - Community Referral	77.97	(10 - 100)	2.59	(1 - 6)
Use Case 2. Start Antibiotics - Well Baby with Risk Factors at Birth	69.35	(4 - 100)	3.48	(1 - 6)
Use Case 3. Start Antibiotics - Unwell at Birth	60.69	(10 - 100)	4.00	(1 - 6)
Use Case 4. Start Antibiotics - Small or Premature Baby who becomes Unwell	72.72	(15 - 100)	3.55	(1 - 6)
Use Case 5. Stop Antibiotics	77.26	(22 - 100)	3.55	(1 - 6)
Use Case 6: Resistance	75.66	(19 - 100)	3.83	(1 - 6)

[1] Prioritization takes the average weight assigned to each use case based on the sliding scale. Note that the respondent could assign every use case at the maximum 100 (i.e., no force rank or sum total).
[2] Rank takes the average of each assigned rank by use case per submission.

Table 14: Initial Use Case Survey – Detailed Results

Prioritization Score [1]						Rank Score [2]					
**Use Case 1**	**Use Case 2**	**Use Case 3**	**Use Case 4**	**Use Case 5**	**Use Case 6**	**Use Case 1**	**Use Case 2**	**Use Case 3**	**Use Case 4**	**Use Case 5**	**Use Case 6**
82	68	45	51	99	19	2	3	5	4	1	6
39	76	30	84	32	74	4	2	6	1	5	3
80	95	50	50	70	60	2	1	4	5	3	6
10	60	10	27	79	61	6	2	4	5	1	3
100	100	100	100	100	100	3	5	2	1	4	6
55	10	50	20	80	70	3	6	4	5	1	2
80	16	40	40	64	73	1	2	3	4	5	6
100	71	48	49	90	75	1	3	4	5	2	6
50	76	51	78	22	39	2	1	3	4	5	6
81	97	99	100	90	88	1	4	3	2	5	6
88	70	92	80	75	85	2	4	1	5	6	3
100	80	80	100	75	100	1	5	4	3	6	2
91	82	60	94	30	22	1	3	4	2	5	6
100	96	90	91	57	100	1	3	5	4	6	2
100	93	92	86	100	100	1	5	4	6	2	3
100	4	66	70		20	1	4	3	2	6	5
66		81	100	82	83	6	4	2	1	5	3
100	83	40	70	52	70	1	2	5	3	6	4
85	90	60	75	90	100	5	2	4	6	1	3
69	67	62	83	71	79	3	4	6	1	5	2
86	89	100	97	83	73	4	3	1	2	5	6
99	86	97	90	65	30	1	4	2	3	5	6
47	52	49	100	100	100	5	6	4	3	2	1
90	70	10	28	90	70	1	3	6	5	2	4
40	50	10	15	95	85	4	3	6	5	1	2
65	95	65	65	95	100						
61	95	32	63	93	94	5	1	6	4	2	3
100	100	100	100	100	100						
100	10	20	70	90	100	1	6	5	4	3	2
51	29	77	92	93	94	4	6	5	2	1	3
80	69	56	59	93	97	3	4	5	6	2	1
100	71	80	100	40	60						
**77.97**	**69.35**	**60.69**	**72.72**	**77.26**	**75.66**	**2.59**	**3.48**	**4.00**	**3.55**	**3.55**	**3.83**

Survey respondents were asked whether there are any other Use Cases or situations where a point-of-care test could help healthcare workers to manage young infants with possible serious bacterial infections. The following comments were received:

1. "Standards around neonates is that the majority of the time we are treating when infection is highly improbable. Hard to imagine something that can replace cultures"
2. "Use Case 3 + would ideally say which antibiotic to start; Use Case 6 should tell you which bacteria is resistant; all of these tests would depend on sensitivity or specificity"
3. "A) Umbilical cord dx - I believe this is often discarded but several studies have studied biomarkers in cord blood and seen promising results. B) I'm not sure how realistic this would be, but a diagnostic for resistance at the time of diagnosis of sepsis could help guide treatment in one visit. I know the mortality rate of neonatal sepsis is very high, and I wonder if that means that use cases 1-4 should be prioritized over use case 5/6. In my mind, a baby who is unwell (use cases 3/4) will be started on antibiotics anyways so I had those at a lower priority, but of course there are issues of resistance and misdiagnosis there too. I believe a large burden of neonatal mortality occurs soon after birth, which was my justification for putting use case 2 at the highest priority"
4. "If the test is low-cost and simple to use by community health workers, then it could be used during community outreach activities to identify a patient at the community level and refer to the nearest health facility for Abx initiation. This is similar to case #1, but starts from the community level for early identification at community/household level -> referral -> and early/immediate initiation."
5. "A baby who had other problems at admission and becomes unwell after admission (diagnosing hospital-acquired sepsis)"
6. "Treatment response: a use case that enables clinicians to non-clinically monitor response to treatment for diagnosed septic neonates. This use case could herald possible antibiotic resistance and rationalize / prioritize blood culture usage"
7. "Test to guide other intervention (e.g. supporting, referral to a higher level center)"
8. "Surgical patients those who have gone through major operations and some patients with Gastroschisis, open spina bifida. Many of these later develop signs and symptoms of sepsis. Some babies are delivered at home under unsterile procedure. Need proper tests to guide on use of antibiotics"
9. "Hospitalized premature infant with respiratory worsening (increase in ventilation or oxygenation needs)"
10. "If there are signs of infection healthcare workers will start antibiotics - hence less useful. However a more pertinent question would be what antibiotics to start - if the diagnostics could identify the bacteria that would be extremely helpful in all cases"

Figure 13: Summary of organizational affiliation for Sepsis Diagnostic Use Case (n = 33)

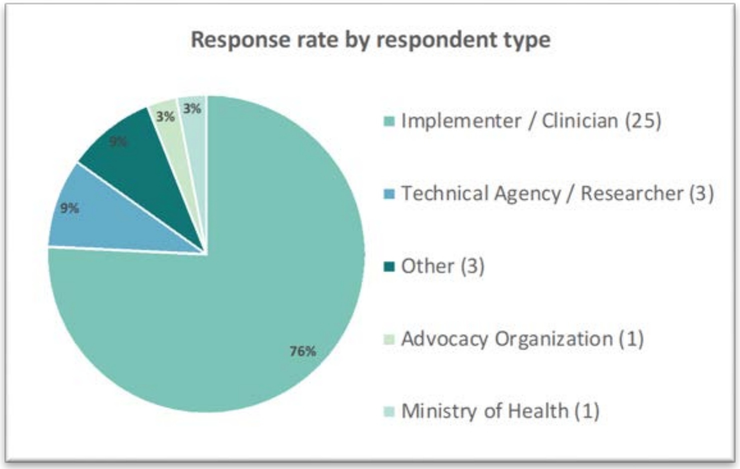

Respondent type	Percentage
Implementer / Clinician (25)	76%
Technical Agency / Researcher (3)	9%
Other (3)	9%
Advocacy Organization (1)	3%
Ministry of Health (1)	3%

Figure 14: Summary of response rate by country for Sepsis Diagnostic Use Case (n = 33)

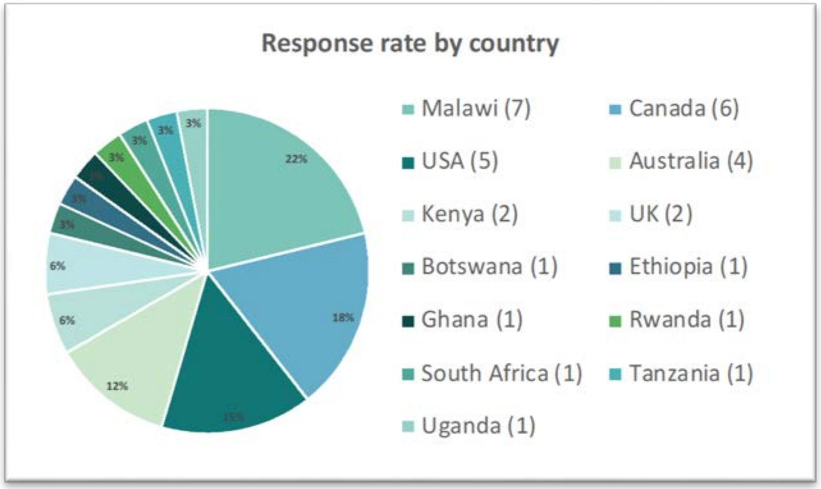

Country	Percentage
Malawi (7)	21%
Canada (6)	18%
USA (5)	15%
Australia (4)	12%
Kenya (2)	6%
UK (2)	6%
Botswana (1)	3%
Ethiopia (1)	3%
Ghana (1)	3%
Rwanda (1)	3%
South Africa (1)	3%
Tanzania (1)	3%
Uganda (1)	3%

Figure 15: Screenshot of Survey Questions

Q14.2 **Question 1.** Based upon the Use Case descriptions provided above, please prioritize Use Case 1-6. (drag the indicator to the priority score for each of the 6 Use Cases)

	Low Priority Medium Priority High Priority<br>0 10 20 30 40 50 60 70 80 90 100
Use Case 1. Start Antibiotics - Community Referral ()	
Use Case 2. Start Antibiotics - Well Baby with Risk Factors at Birth ()	
Use Case 3. Start Antibiotics - Unwell at Birth ()	
Use Case 4. Start Antibiotics - Small or Premature Baby who becomes Unwell ()	
Use Case 5. Stop Antibiotics ()	
Use Case 6: Resistance ()	

Q14.3 **Question 2:** Based upon the Use Case descriptions provided above, Rank Use Case 1-6. (drag the 6 Use Cases below into the appropriate order of rank - 1 being the highest rank, 6 being the lowest rank)

______ Use Case 1. Start Antibiotics - Community Referral (1)
______ Use Case 2. Start Antibiotics - Well Baby with Risk Factors at Birth (2)
______ Use Case 3. Start Antibiotics - Unwell at Birth (3)
______ Use Case 4. Start Antibiotics - Small or Premature Baby who becomes Unwell (4)
______ Use Case 5. Stop Antibiotics (5)
______ Use Case 6. Resistance (6)

RESPIRATORY SUPPORT

At birth, a baby's lungs must transition from fetal to neonatal life in three key ways:

1. fluid in the lungs must be absorbed and replaced with air,
2. lungs must expand fully and regular breathing must be established, and
3. pulmonary blood flow is increased.

When these three things do not happen, a baby will have respiratory distress. Respiratory distress syndrome (RDS) is when there is deficiency of surfactant that is needed to prevent alveolar collapse; this is especially common in premature newborns.

Oxygen provision is important in the care of newborn infants because many conditions that affect babies in the first days of life can result in low levels of oxygen in the body. Hypoxemia, or low levels of oxygen in the blood, is a life-threatening condition that results in increased mortality and morbidity. Prematurity and respiratory distress syndrome (surfactant deficiency), pneumonia and other severe infections, asphyxia, and difficulties in the transition from fetal to neonatal life can all result in hypoxemia. Yet, despite its importance in acute severe illnesses, hypoxemia is often not well recognized or managed in settings where resources are limited. It is therefore important for health workers to know the clinical signs that suggest the presence of hypoxemia and how supplemental oxygen can appropriately be used as an essential lifesaving treatment [32].

INTRODUCTION: CPAP

In high-resource settings, a mother is given steroids before birth if a baby is anticipated to be born preterm to help prevent respiratory distress syndrome (RDS). If RDS still occurs, assisted breathing with continuous positive airway pressure (CPAP) is started. If CPAP is not sufficient, intubation, surfactant and/or ventilation may be needed.

In low-resource settings, many facilities lack the resources to implement CPAP. While many companies make newborn CPAP devices, only a few key players design their devices to work in low-resource settings.

Bubble Continuous Positive Airway Pressure (bCPAP) therapy is a common mode of treatment for RDS in premature neonates and for respiratory illness in young children. bCPAP provides a continuous flow of pressurized air into the patient's nostrils via nasal prongs or a mask; this pressure prevents alveolar collapse during exhalation. In high-income settings, early bCPAP is now preferred over mechanical ventilation as first line therapy for respiratory distress syndrome in preterm infants. bCPAP has been shown to promote production of endogenous surfactant [33] as well as dramatically decrease progression to intubation or death in both high [34-36] and low [37,38] income settings.

In low-resource settings, there is a need for CPAP that is designed for patients who weigh between 1 and 10 kg and that includes an oxygen blender which allows users to provide 21-90% oxygen to the patient when an external oxygen source is connected to the CPAP. The CPAP should ideally contain an integrated air-compressor, blender, and patient interface. Although there are short cuts for delivering positive airway pressure to a baby without an appropriate device, these generally rely on pure oxygen sources from oxygen cylinders or concentrators. Procurement officers should consider current evidence, target level of care, provision, and context when choosing between available CPAP devices. The ability of a CPAP device to deliver positive pressure at low fractional inspired oxygen concentrations (FiO2) is a critical feature for preventing retinopathy of prematurity and chronic lung disease associated with oxygen administration [39,40]. Some CPAP units use heated and humidified gas in the circuit, although the exact benefits of humidification in non-invasive ventilation (i.e. CPAP) in terms of survival, complications from therapy and morbidity are not well established. Humidification, while a feature of some CPAP devices, remains a controversial feature of CPAP in low-resource settings, especially for CPAP devices utilizing compressed ambient air rather than gas cylinder sources.

Table 15: Final TPP for CPAP

Final Target product profile for CPAP		
**Characteristic**	**Optimal**	**Minimal**
SCOPE		
**Intended Use**	To treat respiratory distress and other forms of respiratory illness in infants up to one year of age	
**Target Operator**	For use in low- and middle-income countries by a wide variety of clinicians, including nurses, clinical officers, and pediatricians	
**Target Population**	Neonates (born at any gestational age and require ongoing care)	
**Target Setting**	Hospitals in low-resource settings	
SAFETY AND STANDARDS		
**Quality Management** [1]	ISO 13485:2016 Medical devices – Quality management systems -- Requirements for regulatory purposes	
**Regulation**	At least one of: CE marking, approved by US FDA or another stringent regulatory body of a founding member of IMDRF (e.g., Japan or Australia or Canada)	
TECHNICAL CHARACTERISTICS		
**Flow Driver**	Integrated (on-board air compressor)	
**Oxygen Flow Capacity**	0-10 L/min	
**Pressure**	5-8 cm H20	
**Total (blended) Flow**	0-10 L/min	
**Humidification**	Yes, Heated Humidification	None [2]
**Alarms**	Audio and Visual: Power, low-flow, low-pressure	Audio Power
PURCHASING CONSIDERATIONS		
**Accessories**	Non-proprietary	Proprietary [3]
**Consumables**	Reusable	Available

**Instrument Pricing**	<$1,000 ex-works	<$2,000 ex-works
**Consumable Pricing**	<$10 / patient ex-works	<$15 per patient ex-works
UTILITY REQUIREMENTS		
**Power Source**	Mains with battery backup	Mains Power
**Battery**	Rechargeable integrated battery, >6 hours on a single charge	None [4]
**Voltage**	Model must match the voltage and frequency of the purchasing country's local power grid (e.g., 110-120 VAC at 60 Hz or 220-240 VAC at 50 Hz)	
TRAINING AND MAINTENANCE		
**User Instructions**	User manual and additional training materials (checklists, videos, guides) in at least one national official language for the country of intended use. Attached to device with labels and markings where possible	User manual provided in at least one national official language
**Warranty**	5 years	1 year

[1] There was not 75% voting agreement on the Minimal characteristic. Please refer to the TPP Report discussion for additional detail.
[2] There was not 75% voting agreement on this characteristic. Please refer to the TPP Report discussion for additional detail.
[3] There was not 75% voting agreement on this characteristic. Please refer to the TPP Report discussion for additional detail.
[4] There was not 75% voting agreement on this characteristic. Please refer to the TPP Report discussion for additional detail.

CONSENSUS MEETING SUMMARY: CPAP

To arrive at the final TPP for CPAP (Table 15), we conducted a pre-meeting Delphi-like survey. Based on the pre-meeting Delphi-like survey results (Table 16), characteristics that achieved below 75% agreement were prioritized for discussion at the Consensus Meeting. An overview of the discussion is included below.

- **Humidification**
  - There was disagreement in the group on whether heated humidification was required as a Minimal characteristic.
  - Proponents of heated humidification argued that some of the advantages of heated humidification include:
    - Better outcomes
    - Reduced risk of infection (with heated humidification)
    - Increased comfort and adherence

        - Decreased upper airway mucosal injury
        - Decreased convective heat losses which may lead to hypothermia and more challenging weight gain in infants
        - Decreased lung inflammation from aspirated secretions which has unknown impact on morbidity and mortality of very low birthweight infants.
    - Some potential drawback to heated humidification include:
        - Iatrogenic infection, especially in settings where clean water may not be readily available and humidifiers, which are typically meant for one time use, are being cleaned and re-used between patients
        - High financial cost of adding heated humidified gas
        - High cost of additional consumable required and ongoing maintenance
        - High human resource costs in terms of repair and preparation of non-invasive ventilation units which may limit not only their use, but availability of this life saving technology within our setting
    - Clinicians commented that humidification helps with the avoidance of hypothermia which is becoming increasingly important. These clinicians claimed that it is likely that heated and humidified air is most important for the smallest newborns less than 1-1.25kg. Other clinicians responded that the mortality impact has never been explicitly studied.
    - A research question was created to further explore outcomes and effects with and without heated humidification.
    - *Minimal: No heated humidification*
        - Overall Vote - 58% Agree (n = 31)
        - Clinicians - 61% Agree (n = 23)
        - Excluding involvement with product development - 58% Agree (n = 24)
- **Accessories**
    - There was a discussion surrounding the number of cannulas and hats included with each machine purchased – currently a standard does not exist and therefore it is dependent on the manufacturer. A research question was created to further explore the impact of reusable accessories. An existing JHPIEGO paper "Infection Prevention and Control - Module 6. Processing Surgical Instruments and Medical Devices" was referenced in providing recommendations on how to develop guidelines on the reprocessing of single-use device [7, p. 77-81].
    - *Minimal: Proprietary*
        - Overall Vote - 74% Agree (n = 31)
        - Clinicians - 79% Agree (n = 19)
        - Excluding involvement with product development - 75% Agree (n = 24)
- **Battery**
    - Participants noted the importance of a back-up power supply. Other participants noted the impact on price if the back-up power is needed for both heated humidification and an on-board air compressor. Product developers explained the negative impact that power outages have on the product and the importance of strong utility infrastructure to withstand power outages, including the principle of grounding [41].
    - There was agreement in the room that all characteristics relating to Utility Requirements (e.g., Back-up Battery; Battery Power; Batteries; Voltage; Power Requirement; Maximum Power Consumption; Response During Power Outage; Surge Protection,

Electrical Plug) be reviewed and harmonized following the TPP meeting. In this specific case, the language used in the Optimal characteristic was adjusted during this harmonization review following the vote.

- *Optimal: Built-in rechargeable battery, autonomy >6 hours, automatic switch to battery in case of power failure, automatic recharge on connection to mains (only applicable to the electric CPAP generator model)-*
  - Overall Vote - 95% Agree (n = 12)
  - Clinicians - 100% Agree (n = 27)
  - Excluding involvement with product development - 94% Agree (n = 16)
- *Minimal: None (but assumption that facility has back up power for 6 hours)*
  - Overall Vote - 47% Agree (n = 30)
  - Clinicians - 38% Agree (n = 18)
  - Excluding involvement with product development - 43% Agree (n = 23)
- *Final post Utility Harmonization - Optimal: Rechargeable integrated battery, >6 hours on a single charge*
- *Final post Utility Harmonization - Minimal: None*

- **Instrument Pricing**
  - One participant mentioned that the pricing for commercially available products that meet this draft specification range from $1,000 - $3,000. Consensus achieved via voting.
  - *Minimal: <$2,000 ex-works*
    - Overall Vote - 71% Agree (n = 21)
    - Clinicians - 92% Agree (n = 12)
    - Excluding involvement with product development - 80% Agree (n = 15)
- **Consumable Pricing**
  - Participants commented that the minimum price was too high for single-use products, especially for certain markets where consumers may be paying out of pocket and the cost is prohibitively high.
  - *Minimal: <$15 per set ex-works*
    - Overall Vote - 79% Agree (n = 24)
    - Clinicians - 86% Agree (n = 14)
    - Excluding involvement with product development - 88% Agree (n = 17)

DELPHI-LIKE SURVEY: CPAP

Table 16: Delphi-like survey results for CPAP TPP prior to Consensus Meeting (data as of Oct 25, 2019)

	Optimal		Minimal		
**Characteristic**	**Optimal requirement**	**% agreement (n size)**	**Minimal requirement**	**% agreement (n size)**	**Collated comments from Delphi-like survey**
**Intended Use**	Optimal: To treat respiratory distress and other forms of respiratory illness in infants up to one year of age.	**95%**<br>**n = 42**	Minimal: Same as Optimal.	**95%**<br>**n = 37**	12 comments summarized below<br>• Theme: Narrow vs. Broaden Age Range<br>• Target Population is defined as neonates, but Intended Use defined as infants up to one year of age. Need to synch and/or clarify age of patient
**Target Operator**	Optimal: For use in low- and middle-income countries by a wide variety of clinicians, including nurses, clinical officers, and pediatricians.	**93%**<br>**n = 42**	Minimal: Same as Optimal	**90%**<br>**n = 39**	7 comments summarized below<br>• Theme: Training and Supervision should accompany Bubble CPAP<br>• Requires training and supervision when introducing to new clinical and nursing professionals
**Target Population**	Optimal: Neonates (<28 days)	**88%**<br>**n = 42**	Minimal: Same as Optimal.	**85%**<br>**n = 39**	16 comments summarized below<br>• Theme: Narrow vs. Broaden Age Range<br>• Target Population is neonates but Intended Use infants up to one year of age. Need to synch and/or clarify age of patient<br>• Bubble CPAP is very effective in neonatal population but also evidence suggests that has a role in respiratory illness of other causes in infants and children <5 yrs, such as pneumonia and bronchiolitis
**Target Setting**	Optimal: Hospitals in low-resource settings	**93%**<br>**n = 41**	Minimal: Same as Optimal.	**86%**<br>**n = 37**	11 comments summarized below<br>• Theme: Need to define what is meant by hospital<br>• Bubble CPAP can be used in hospitals in low-resource settings but ideally also high-functioning health centres

	Optimal		Minimal		
					• Requires training and supervision when introducing to new clinical and nursing professionals<br>• May also need to define what is needed at setting: electricity, sterilization capabilities, etc.
**International Standard**	Optimal: ISO 13485:2016 Medical devices – Quality management systems -- Requirements for regulatory purposes.	**86%**<br>**n = 22**	Minimal: Same as Optimal.	**85%**<br>**n = 20**	7 comments<br>• Theme: Low familiarity on what ISO 13485 means
**Regulation**	Optimal: CE marking or US FDA Clearance	**69%**<br>**n = 26**	Minimal: Same as Optimal.	**68%**<br>**n = 25**	14 comments summarized below<br>• Theme: Reduce regulatory options or add more flexibility<br>• CE Mark alone is sufficient<br>• Consider additional 'or' options:<br>o Other Stringent Regulatory Authorities – Japan or Australia or Canada<br>o Consider regulatory bodies of Low- and Middle-Income Countries
**Flow Driver**	Optimal: Integrated (on-board air compressor)	**90%**<br>**n = 29**	Minimal: Same as Optimal.	**86%**<br>**n = 28**	9 comments summarized below<br>• Need to clarify what is meant by flow driver and on-board air compressor and whether this impacts the Accessories or Consumables characteristics (e.g., does an integrated on-board air compressor require proprietary)
**Oxygen Flow Capability**	Optimal: 0-10 L/min	**86%**<br>**n = 37**	Minimal: Same as Optimal.	**79%**<br>**n = 33**	15 comments summarized below<br>• If the Intended Use is up to 1 year of age (or more), then flows higher than 10 L/min may be required

	Optimal		Minimal		
					• Instead of Oxygen Flow Capability, perhaps Fio2 range or Peep range should be considered
**Pressure**	Optimal: 5-8 cm H20	**84%**<br>**n = 38**	Minimal: Same as Optimal.	**80%**<br>**n = 35**	12 comments summarized below<br>• Additional ranges to consider:<br>o Weaning<br>o Older babies<br>o Extreme cases
**Total (blended) Flow**	Optimal: 0-10 L/min	**86%**<br>**n = 37**	Minimal: Same as Optimal.	**85%**<br>**n = 34**	10 comments summarized below<br>• If the Intended Use is up to 1 year of age (or more), then flows higher than 10 L/min may be required<br>• Instead of Oxygen Flow Capability, perhaps Fio2 range or Peep range should be considered
**Humidification**	Optimal: Yes, Heated Humidification	**95%**<br>**n = 38**	Minimal: None	**62%**<br>**n = 34**	17 comments summarized below<br>Some bCPAP units use heated and humidified gas in the circuit, although the exact benefits of humidification in non-invasive ventilation (i.e. bCPAP) in terms of survival, complications from therapy and morbidity are not well established.<br>Potential benefits of heating and humidification could include:<br>• Increased comfort and adherence<br>• Decreased upper airway mucosal injury<br>• Decreased convective heat losses which may lead to hypothermia and more challenging weight gain in infants<br>• Decreased lung inflammation from aspirated secretions which has unknown impact on morbidity and mortality of very low birthweight infants.<br>Potential drawbacks to heated humidification include:

	Optimal		Minimal		
					• Iatrogenic infection, especially in settings where clean water may not be readily available and humidifiers, which are typically meant for one time use, are being cleaned and re-used between patients<br>• High financial cost of adding heated humidified gas<br>• High human resource costs in terms of repair and preparation of non-invasive ventilation units which may limit not only their use, but availability of this life saving technology within our setting<br><br>It is likely that heated and humidified air is most important for the smallest newborns less than 1-1.25kg although this has never been explicitly studied.
**Alarms**	Optimal: Audio/Visual Power, low-flow, low-pressure	**90%**<br>**n = 39**	Minimal: Audio Power	**85%**<br>**n = 39**	10 comments summarized below<br>• FiO2 alarms and not necessarily flow-rate alarms may be more critical<br>• Need to clarify Audio/Visual. Is this Audio and/or Visual or Audio or Visual
**Consumables**	Optimal: Reusable	**88%**<br>**n = 41**	Minimal: Available	**82%**<br>**n = 39**	15 comments summarized below<br>• Clarify what is meant by consumable and reusable:<br>o Bottle<br>o Tubing<br>o Nasal Cannulas<br>o Hat<br>• Potential benefits of reusable consumables:<br>o Lower cost<br>o Reduces supply chain delays<br>• Potential drawbacks of reusable consumables:<br>o Infection risk (perhaps mitigated with instructions / guidance for decontamination; specify autoclavable or disinfectable with specific cleaning agent)

	Optimal		Minimal		
					o May not allow for approval by Stringent Regulatory Authority
**Accessories**	Optimal: Non-proprietary	84%<br>n = 31	Minimal: Proprietary	64%<br>n = 28	13 comments<br>• Clarify what is meant by accessories:<br>o Bottle<br>o Tubing<br>o Nasal Cannulas<br>o Hat<br>• Potential benefits of proprietary accessories:<br>o Designed to reduce user errors<br>• Potential drawbacks of proprietary accessories:<br>o Often cost more<br>o Introduces delays due to supply chain<br>o May not allow for approval by Stringent Regulatory Authority
**Back-up Battery**	Optimal: Built-in rechargeable battery, autonomy >1 hour, automatic switch to battery in case of power failure, automatic recharge on connection to mains (only applicable to the electric CPAP generator model)	89%<br>n = 38	Minimal: None	52%<br>n = 33	21 comments summarized below<br>• Potential benefits of back-up battery:<br>o Allows for use in between power outage and when the generator turns on<br>• Potential drawbacks of back-up battery:<br>o Increases the cost of device; may be best to resolve with back-up UPS
**Voltage**	Optimal: 110-240V 50-60hz	82%<br>n = 28	Minimal: 220-240V 50-60hz	83%<br>n = 29	13 comments as summarized below<br>• Voltage can always be corrected with step-up / step-down transformers; however, these come at an added cost. So whether the cost be borne by the purchaser (Caribbean, Central- or South-American

	Optimal		Minimal		
					countries w/ 120V) or the manufacturer who makes devices that can work across all contexts<br>• Frequency needs to be appropriate for frequency rating of specific country, as this is something that cannot be corrected and though 50 Hz can be used in a 60 Hz system, it is hard on the device and it will be compromised<br>• Voltage stabilizers and surge suppressors are important to consider
**User Manual**	Optimal: User manual and additional training materials (checklists, videos, guides) in English and local language. Attached to device with labels and markings where possible.	**95%**<br>**n = 41**	Minimal: User manual provided.	**77%**<br>**n = 39**	12 comments as summarized below<br>• A variety of hard and soft copy materials mentioned with particular mentions of difficulty in reading a user manual and preference for videos so people can see vs. read<br>• All claims must be filed with the regulatory dossier, so this is not as straight forward as a simple translation. Appropriate, professional translations are a must and are costly to the manufacturer. Additionally, local language varies greatly across a country and is often-times not even the official language of the country and so this may not be a reasonable ask of manufacturers
**Warranty**	Optimal: 5 years	**79%**<br>**n = 39**	Minimal: 1 year	**68%**<br>**n = 38**	19 comments as summarized below<br>• Desire to increase Minimal (1 year) but acknowledgement that this may come at a cost that donors or procurement agencies may not be ready for
**Instrument Pricing**	Optimal: <$1,000 ex-works	**82%**<br>**n = 33**	Minimal: <$2,500 ex-works	**52%**<br>**n = 31**	21 comments as summarized below<br>• Extremely price-sensitive geography and even $1,000 was viewed as too expensive by some respondents

	Optimal		Minimal		
					• Ex-works not likely a true measure of landed costs<br>• Devices below $2,000 ex-works would encounter some sort of other trade-off (no air compressor, no humidification, 1 year warranty, etc.)
**Consumable Pricing**	Optimal: <$10 / patient ex-works	**83%**<br>**n = 29**	Minimal: <$50 per patient ex-works	**42%**<br>**n = 31**	19 comments as summarized below<br>• Extremely price-sensitive geography and even $10 was viewed as too expensive by some respondents, especially for countries where patient pays out of pocket for consumables (e.g. Nigeria)<br>• Ex-works not likely a true measure of landed costs<br>• If consumables were reusable, then price point slightly higher than $10 is more realistic<br>• "$10 is too low for effective circuits"

Figure 16: Summary of organizational affiliation for CPAP TPP from Delphi-like Survey prior to Consensus Meeting (data as of Oct 25, 2019)

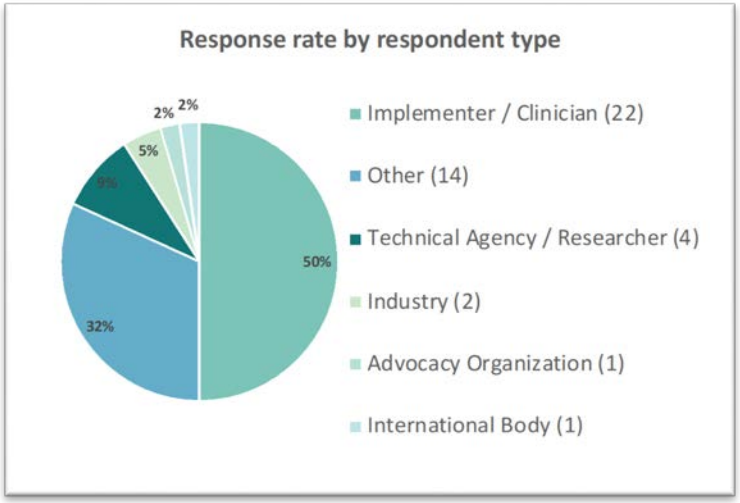

Respondent type	Percentage
Implementer / Clinician (22)	50%
Other (14)	32%
Technical Agency / Researcher (4)	9%
Industry (2)	5%
Advocacy Organization (1)	2%
International Body (1)	2%

Figure 17: Summary of response rate by country for bCPAP TPP from Delphi-like Survey prior to Consensus Meeting (data as of Oct 25, 2019)

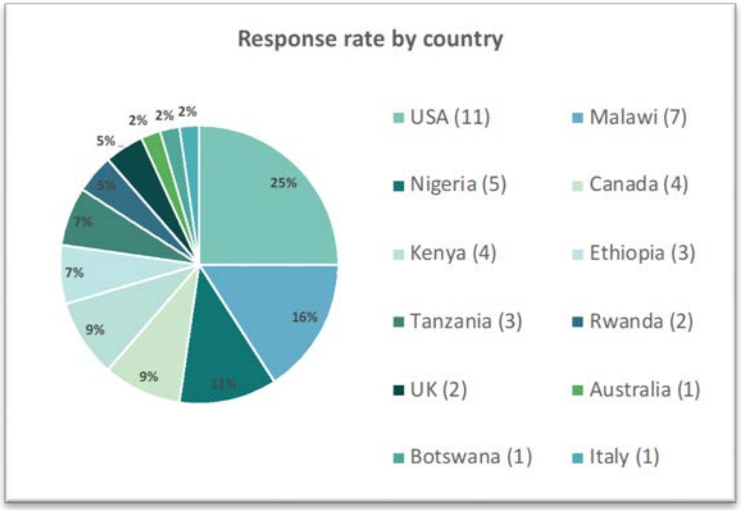

Country	Percentage
USA (11)	25%
Malawi (7)	16%
Nigeria (5)	11%
Canada (4)	9%
Kenya (4)	9%
Ethiopia (3)	7%
Tanzania (3)	7%
Rwanda (2)	5%
UK (2)	5%
Australia (1)	2%
Botswana (1)	2%
Italy (1)	2%

INTRODUCTION: FLOW SPLITTER

A flow splitter allows the output of a concentrator or other oxygen source to be split between multiple patients while independently monitoring and adjusting each flow rate. Each of the outputs should measure from 0-2 liters per minute (LPM or L/min) and should have the same FiO2 as the source gas it is attached to. Please see below for further considerations.
When using an oxygen concentrator or oxygen with neonates, low flow is critical in order to avoid preventable disability including retinopathy of prematurity (ROP) and chronic lung disease. A significant number of preventable childhood blindness due to ROP in low- and middle-income countries (LMIC) has been documented [42,43]. Importantly, this is observed in children at higher birthweights and gestational ages than children in high-income settings, suggesting an association with rapid expansion of neonatal care, perhaps without adequate attention to the quality of care or harms of oxygen administration. Neonatal units seeking to provide comprehensive care should consider the procurement of splitters and flow meters with precision adjustment at a minimum of 0.1 – 0.125 L/min. As health facilities advance, introduction of microcalibrated flow meters with precision finer than 0.1 L/min or oxygen blenders should be considered [44].

FINAL TPP: FLOW SPLITTER

Table 17: Final TPP for Flow Splitter

Final target product profile for Flow Splitter		
**Characteristic**	**Optimal**	**Minimal**
SCOPE		
**Intended Use**	To allow multiple patients to receive individually adjusted flow rates from a single source of oxygen	
**Target Operator**	For use in low- and middle-income countries by a wide variety of clinicians, including nurses, clinical officers, and pediatricians	
**Target Population**	Neonates (born at any gestational age and require ongoing care)	

**Target Setting**	Hospitals in low-resource settings, but, may be used in health facilities based on country guidelines	Hospitals in low-resource settings
SAFETY AND STANDARDS		
**Quality Management** [1]	ISO 13485:2016 Medical devices – Quality management systems -- Requirements for regulatory purposes	
**Regulation**	At least one of: CE marking, approved by US FDA or another stringent regulatory body of a founding member of IMDRF (e.g., Japan or Australia or Canada)	
TECHNICAL CHARACTERISTICS		
**Air Flow per Patient**	0-2 L/min	
**Flow Control**	Each patient has individually controlled flow rate	
**Number of Outputs**	5	2
**Indication**	Each flow rate has a visual indicator	
PURCHASING CONSIDERATIONS		
**Instrument Pricing**	<$100 ex-works	<$600 ex-works
TRAINING AND MAINTENANCE		
**Maintenance**	No/minimal maintenance	

[1] There was not 75% voting agreement on the Minimal characteristic. Please refer to the TPP Report discussion for additional detail.

CONSENSUS MEETING SUMMARY: FLOW SPLITTER

To arrive at the final TPP for Flow Splitter (Table 17), we conducted a pre-meeting survey to prioritize the items for discussion at the Consensus Meeting for characteristics that achieved below 75% agreement in the survey results (Table 18). An overview of the discussion at the Consensus Meeting of these characteristics is included below.

- **Instrument Pricing**
  - There was disagreement on the Minimal characteristic for instrument pricing as it was dependent on the number of splitters included in the device. Participants noted that there is a wide range of commercial products available ranging in price from $80 - $600. Accuracy implications remain a key concern for neonatal use. Product developers noted that ISO and CE Mark certification will require that Flow Splitter covers 30-40% accuracy, however, this may increase the price to the $600 mark with 5 ranges included. Therefore, a tradeoff exists in the current market

whereby a cost reduction would be at the expense of accuracy. One basic work-around discussed at the hospital level was to utilize an oxygen monitor which can cost around $150 but may be used for multiple use-cases.

- *Minimal: <$600 ex-works*
  - Overall Vote - 82% Agree (n = 22)
  - Clinicians - 79% Agree (n = 14)
  - Excluding involvement with product development - 82% Agree (n = 22)

DELPHI-LIKE SURVEY: FLOW SPLITTER

Table 18: Delphi-like survey results for Flow Splitter TPP prior to Consensus Meeting (data as of Oct 25, 2019)

	Optimal		Minimal		
**Characteristic**	**Optimal requirement**	**% agreement (n size)**	**Minimal requirement**	**% agreement (n size)**	**Collated comments from Delphi-like survey**
**Intended Use**	Optimal: To allow multiple patients to receive individually adjusted flow rates from a single oxygen source.	**94%**<br>**n = 17**	Minimal: Same as Optimal.	**94%**<br>**n = 16**	2 comments as summarized below<br>• Recommended for neonatal and low flow oxygen as per interagency oxygen therapy guide<br>• Preference for low-pressure piping and a separate flow meter beside each bed rather than a flow splitter and having the flow meters far from the patients
**Target Operator**	Optimal: For use in low- and middle-income countries by a wide variety of clinicians, including nurses, clinical officers, and pediatricians.	**100%**<br>**n = 16**	Minimal: Same as Optimal	**100%**<br>**n = 15**	0 comments
**Target Population**	Optimal: Neonates (<28 days)	**94%**<br>**n = 16**	Minimal: Same as Optimal.	**93%**<br>**n = 15**	4 comments as summarized below<br>• Theme: Broaden Age Range<br>• For sick and small newborns likely need different precision of flow adjustment but same over all flow range as you need for infants; Change to 6 months of age<br>• Any child requiring oxygen

	Optimal		Minimal		
**Target Setting**	Optimal: Hospitals in low-resource settings	**100%**<br>**n = 13**	Minimal: Same as Optimal.	**100%**<br>**n = 12**	3 comments as summarized below<br>• Theme: Broaden Target Setting<br>• Optimally, it would be good to have a flow splitter for transport / referrals<br>• Optimal should be health centres (primary)
**International Standard**	Optimal: ISO 13485:2016 Medical devices – Quality management systems -- Requirements for regulatory purposes.	**100%**<br>**n = 8**	Minimal: Same as Optimal.	**100%**<br>**n = 7**	2 comments
**Regulation**	Optimal: CE marking or US FDA Clearance	**83%**<br>**n = 12**	Minimal: Same as Optimal.	**82%**<br>**n = 11**	6 comments as summarized below<br>• Theme: Add more flexibility v. irrelevance of characteristic<br>• Consider additional 'or' options:<br>o Other Stringent Regulatory Authorities – Japan or Australia or Canada<br>o Consider regulatory bodies of Low- and Middle-Income Countries
**Air Flow per Patient**	Optimal: 0-2 L/min	**81%**<br>**n = 16**	Minimal: Same as Optimal.	**93%**<br>**n = 15**	5 comments as summarized below<br>• Theme: Confusion as to total Air Flow per patient versus capacity of total Flow Splitter<br>o 3-5 flow meters and max total of 10 LPM<br>• Theme: Want more than 2 L/min (for older children)<br>o 3 L/min<br>o 5 L/min

	Optimal		Minimal		
					• Theme: Confusion as to role of Flow Splitter versus Flow Meter on Oxygen Concentrator TPP<br>o Add resolution in increments on .25 LPM
**Flow Control**	Optimal: Each patient has individually controlled flow rate.	94%<br>n = 16	Minimal: Same as Optimal.	93%<br>n = 15	3 comments
**Number of Outputs**<br>*(corrected from 'Pressure')*	Optimal: 5	94%<br>n = 16	Minimal: 2	100%<br>n = 15	6 comments as summarized below<br>• Theme: Consider a range vs. an absolute<br>• Optimal: >2<br>• Minimal: At least 2
**Indication**	Optimal: Each flow rate has a visual indicator.	100%<br>n = 16	Minimal: Same as Optimal.	100%<br>n = 15	0 comments
**Maintenance**	Optimal: No/minimal maintenance.	87%<br>n = 15	Minimal: Same as Optimal.	87%<br>n = 15	4 comments as summarized below<br>• Routine cleaning with regularly available cleaning products<br>• Need to add Inlet filter to Optimal<br>• Preference for ability to replace individual flow meters
**Instrument Pricing**	Optimal: <$100 ex-works	93%<br>n = 15	Minimal: <$600 ex-works	64%<br>n = 14	6 comments as summarized below<br>• Theme: Specify capacity of splitter (e.g. $600 for 5 user splitter)<br>• Theme: Range of prices suggested for Minimal<br>• $500<br>• $125 but manufactured under ISO is key

Figure 18: Summary of organizational affiliation for Flow Splitter TPP from Delphi-like Survey prior to Consensus Meeting (data as of Oct 25, 2019)

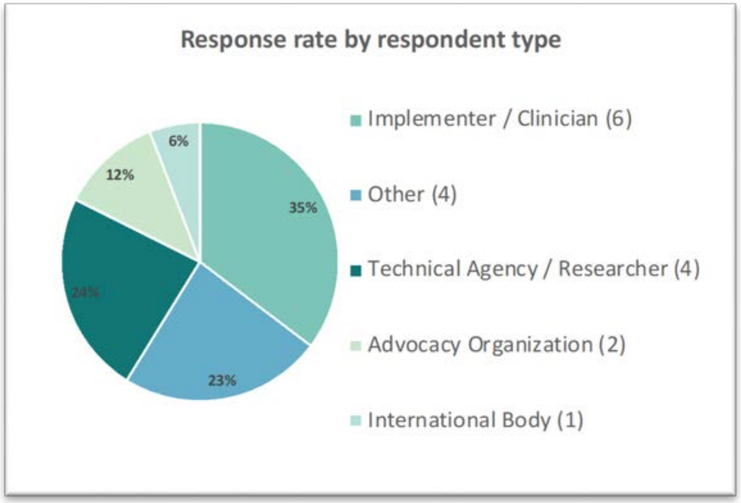

Respondent type	Percentage
Implementer / Clinician (6)	35%
Other (4)	24%
Technical Agency / Researcher (4)	24%
Advocacy Organization (2)	12%
International Body (1)	6%

Figure 19: Summary of response rate by country for Flow Splitter TPP from Delphi-like Survey prior to Consensus Meeting (data as of Oct 25, 2019)

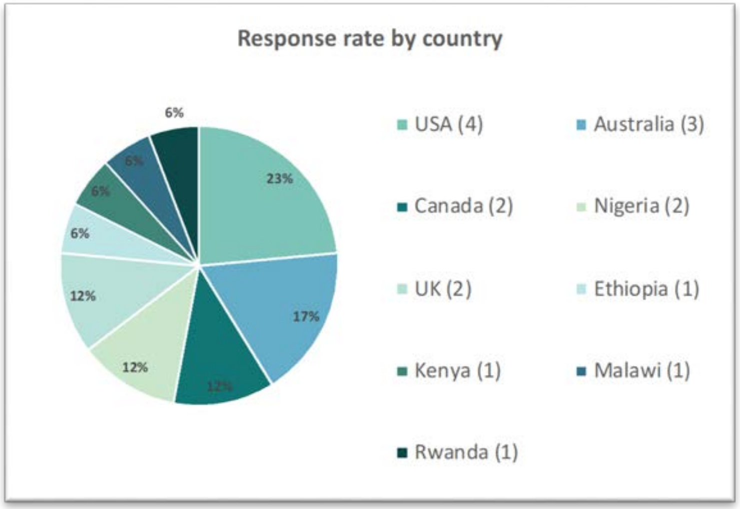

Country	Percentage
USA (4)	24%
Australia (3)	18%
Canada (2)	12%
Nigeria (2)	12%
UK (2)	12%
Ethiopia (1)	6%
Kenya (1)	6%
Malawi (1)	6%
Rwanda (1)	6%

INTRODUCTION: OXYGEN CONCENTRATOR

For newborns with breathing difficulties and/or infections, oxygen is vital to survival. Yet, access to oxygen can be scarce in low-resource settings. To meet this need, an oxygen concentrator is a device able to concentrate oxygen from the air for use with a multitude of devices. While use of concentrators is helpful, facilities should always have a power-independent oxygen source, such as a cylinder, available for back up.

Oxygen concentrators typically output oxygen between 85-100% FiO2, with flows between 2-10 LPM with typically one or two outlets. The percent oxygen a patient will receive depends on each mode of delivery (i.e., nasal prongs, nasal catheter, facemask, etc.). Passive humidification is sometimes available but recommended against by the World Health Organization [32]. A flow splitter allows the output of a concentrator to be split between multiple patients while independently monitoring and adjusting the flow rate to each. It is important to consider that high flow oxygen concentrators should be paired with an appropriate flow splitter for the safety of the neonate.

FINAL TPP: OXYGEN CONCENTRATOR

Table 19: Final TPP for Oxygen Concentrator

Final target product profile for Oxygen Concentrator		
**Characteristic**	**Optimal**	**Minimal**
SCOPE		
**Intended Use**	To provide medical oxygen for use in a healthcare setting	
**Target Operator**	For use in low- and middle-income countries by a wide variety of clinicians, including nurses, clinical officers, and pediatricians	
**Target Population**	Neonates (born at any gestational age and require ongoing care)	
**Target Setting**	Hospitals in low-resource settings, but, may be used in health facilities based on country guidelines	Hospitals in low-resource settings
SAFETY AND STANDARDS		

**Quality Management** [1]	ISO 13485:2016 Medical devices – Quality management systems -- Requirements for regulatory purposes	
**Regulation**	At least one of: CE marking, approved by US FDA or another stringent regulatory body of a founding member of IMDRF (e.g., Japan or Australia or Canada)	
TECHNICAL CHARACTERISTICS		
**Flow Meter**	At least 2 with each 0 to 10 LPM flow meter, min incremental 0.5 LPM	At least 1 [flow meter] with 0 to 10 LPM flow meter, min incremental 0.5 LPM
**Minimal Flow Rate**	0.5 LPM (if used without a flow splitter)	2 LPM
**Flow Rate**	10 LPM	8-10 LPM
**Time to Reach 95% of Specified Performance**	< 5 Min	
**Oxygen Purity**	93% ± 3%	
**Alarms**	Visual and auditory alarms	
**Indicators**	Clearly labeled or marked with pictures and language. Audible alerts and diagnostic indicator where possible	UI easy to understand, numbers and displays clearly visible
**Mobility**	Whole unit moveable with wheels on at least two feet	
**Oxygen Monitor**	Visual and audible status, preferably with color coding for early warning	Visual and audible status
**Oxygen Outlet**	Recessed, replaceable metal barbs	
**Noise Level**	≤50 decibels; low as possible	
**Weight**	<30 kg	
**Durability and Robustness**	Harsh ambient condition, temperature 5-45 °C, humidity 15% to 95%, dusty air, elevation >=2000 meters	Temperature 10-40 °C, humidity 15%-95% elevation up to 2000 meters
**Usage Meter**	Non-resettable digital or analog meter displaying cumulative hours of operation	

PURCHASING CONSIDERATIONS		
**Instrument Pricing**	<$500 ex-works	<$1600 ex-works
UTILITY REQUIREMENTS		
**Power Source**	Mains Power	Mains Power
**Power Consumption**	<275W at 5 LPM	Scales with delivery output — i.e., consumes less power at lower flow rates
**Voltage**	Model must match the voltage and frequency of the purchasing country's local power grid (e.g., 110-120 VAC at 60 Hz or 220-240 VAC at 50 Hz)	
TRAINING AND MAINTENANCE		
**User Instructions**	User manual and additional training materials (checklists, videos, guides) in at least one national official language for the country of intended use. Attached to device with labels and markings where possible	User manual provided in at least one national official language
**User Skill Level**	Minimal to none	
**Warranty**	5 years	1 year
**Decontamination**	Reduced recessed areas and need for specialized cleaning procedures or products	Easy to clean flat surfaces, compatible with common disinfecting agents
**Preventive Maintenance Interval**	Should not need preventive maintenance more than once a year	Should not need preventive maintenance more than 4 times a year (quarterly)
**Technical Skill Maintenance**	Minimally trained technician	Trained technician with training in basic operation and maintenance

**Cleaning Interval**	Provide two filters that are durable, washable, easy to remove	Device exterior to be wiped effectively with a mild solution of detergent or cleaning agent (weekly), without connection to mains power. Gross particle filter to be cleaned effectively when removed and washed with soap and water (weekly). Do not clean with alcohol. (User care needed more often in very dusty environments.) [2]
**Tools Required**	No specialized tools required	Minimal specialized tools for sieve bed and filter replacement
**Filters**	Replaceable washable reusable	

[1] *There was not 75% voting agreement on the Minimal characteristic. Please refer to the TPP Report discussion for additional detail.*
[2] *Source:* WHO-UNICEF Technical Specifications and Guidance for Oxygen Therapy Devices [6, p. 95]

CONSENSUS MEETING SUMMARY: OXYGEN CONCENTRATOR

To arrive at the final TPP for Oxygen Concentrator (Table 19), we first leveraged the extensive work conducted by PATH in the "Design for reliability: Ideal product requirement specifications for oxygen concentrators for children with hypoxemia in low-resource settings" [45]. We conducted a pre-meeting survey to prioritize the items within this existing TPP to discuss at the Consensus Meeting. Specifically, characteristics that achieved below 75% agreement in the survey results (Table 20). An overview of the discussion at the Consensus Meeting of these characteristics is included below.

- **Flow Meter**
  - For the Optimal characteristic, rather than specifying the flow should be split evenly at 0-5 LPM (Liters Per Minute) in each of the two meters, the range should be 0-10 LPM with the ability to split however the user wants across the two outputs. For the Minimal characteristic, clinicians noted that a flow meter that goes to a minimum of 0.25 increments would be beneficial since 0.5 LPM can even be high for neonates. Product developers noted that from a technical perspective, an easy range is 0-10 with 5% resolution, but that there would be inaccuracy at the lower bound and therefore, would recommend 0-1 graduations. It was noted that a flow splitter paired with an oxygen concentrator would suit requirements at low flow rates and therefore, a flow splitter should always be available with an oxygen concentrator. International agencies noted that: "Ideal setup would be to have a concentrator connected to a 5-way flow splitter, with those flowmeters ranging from 0-2 LPM, with increments of 0.25 LPM or less. In other words, if the optimal requirement of 2 flowmeters is to be able to service two neonatal patients at

once, the 0.5 LPM increments on the flowmeters may not be granular enough and so you may need an additional low-flow meter anyway...Optimal [should be] a 10 LPM unit with 2 flowmeters, up to 5 LPM each. Minimal [should be] 8 LPM unit with 1 flowmeter up to 8 LPM."

- *Minimal: Must have flow splitter with at least 1 with 0 to 10 LPM flow meter, min incremental 0.5 LPM*
  - Overall Vote - 100% Agree (n = 29)
  - Clinicians - 100% Agree (n = 22)
  - Excluding involvement with product development - 100% Agree (n = 29)

- **Flow Rate**
  - No vote required as the rate does not matter when flow splitter is required. The Pre-Meeting Survey report highlighted an emerging theme that there is a lack of clarity on why such high LPM would be used for neonates. One comment noted "I choose higher flow + splitter so that oxygen could be administered to more kids. Ideally you could do this and still titrate at least 1/2-1/4 LPM for individual children". In response, another participant commented "this is a bit of a double-edged sword because you need higher flow rates for CPAP, but low flow (< 1LPM) for standard low-flow O2 therapy. Thus, this would come down to installation, how the concentrators are used in a ward."
  - *Optimal: 10 LPM*
  - *Minimal: 8-10 LPM*
- **Time to Reach 95% of Specified Performance**
  - Consensus achieved in room (without a Mentimeter vote) that Minimal should be the same as Optimal.
  - *Minimal: < 5 Min (same as Optimal)*
- **Alarms**
  - There was a discussion on both the Optimal and Minimal alarms required, and consensus was achieved in the room without a vote. Clinicians noted that visual lights are very helpful. Clinicians requested a sounding alarm if battery or power failure and a visual alarm for flow rate and pressure (i.e., Oxygen Supply) and ideally for filter status as well. International agencies noted that the WHO-UNICEF Technical Specifications and Guidance for Oxygen Therapy Devices [6, p. 94] defines alarms as "Audible and/or visual alarms for low oxygen concentration (<82%), low battery and power supply failure. Audible and/or visual alarms for high temperature, low/high/no-flow rate and/or low/high pressure."
  - *Optimal: Visual and auditory alarms*
  - *Minimal: Same as Optimal*
- **Indicators**
  - Consensus was achieved in the room for no change to the Optimal requirement as this was covered in the standards and therefore, there was no need for a separate requirement.
  - *Optimal: Clearly labeled or marked with pictures and language. Audible alerts and diagnostic indicator where possible.*
- **Mobility**
  - A discussion on both the Optimal and Minimal characteristics centered on the mobility requirements for the oxygen concentrator. Clinicians requested two wheels only so that that the equipment cannot be as easily moved. International agencies noted that the WHO-UNICEF Technical Specifications and Guidance for Oxygen Therapy Devices [6, p. 94] defines the whole unit should be moveable with wheels on at least two legs. Product developers noted that from a technical perspective, brakes can be difficult to implement with small wheels.

- *Optimal and Minimal: Whole unit should me movable, with wheels on at least two feet*
  - Overall Vote - 100% Agree (n = 24)
  - Clinicians - 100% Agree (n = 19)
  - Excluding involvement with product development - 100% Agree (n = 24)

- **Noise Level (previously titled 'Sound Level – Operating')**
  - Consensus was achieved that the sound level characteristic was referring the operating noise level. Product developers noted that from a technical standpoint, CE mark requires that this be under 50 decibels for operating noise [46]. Consensus was achieved in the room (without a Mentimeter vote) that the "lower the decibel level, the better" and that Optimal and Minimal should be the same. The spirit of the conversation emphasized that the noise levels should be as low as possible to protect the babies hearing.
  - *Optimal: ≤50 decibels; low as possible*
  - *Minimal: Same as Optimal*

- **Cleaning Interval**
  - There was disagreement for the Optimal cleaning interval. Clinicians noted that currently the external filter must be cleaned once a week and the optimal cleaning interval would be once a month. They noted that "none required" for an Optimal cleaning interval was simply not practical. Consensus was achieved in the room (without a Mentimeter vote) that the Minimal requirement should meet the WHO-UNICEF Technical Specifications and Guidance for Oxygen Therapy Devices [6, p. 95] and the Optimal should meet those guidelines and also be durable, easy to remove, and easy to clean.
  - *Optimal: Provide two filters that are durable, washable, easy to remove*
  - *Minimal: Device exterior to be wiped effectively with a mild solution of detergent or cleaning agent (weekly), without connection to mains power. Gross particle filter to be cleaned effectively when removed and washed with soap and water (weekly). Do not clean with alcohol. (User care needed more often in very dusty environments.)* [6]

- **Preventive Maintenance Interval**
  - There was disagreement for both the Optimal and Minimal preventive maintenance interval characteristics. The discussion highlighted the importance of cost effectiveness and the risk associated with too frequent maintenance intervals given most hospitals have annual preventive maintenance processes. One idea discussed was creating a device that measures oxygen levels and once it drops below a certain level, would flag that maintenance is required. Product developers noted that manufacturers claim 30,000 hours (roughly 3 years) with regular maintenance, but often the true maintenance frequency may vary based on the wide range of operating conditions (i.e., may require more or less maintenance). One suggestion in the Pre-Meeting survey comments was to "measure oxygen concentration with a calibrated oxygen analyzer" to which another participant clarified that "not all analyzers need to be calibrated (e.g. those with ultrasonic sensors)".
  - *Optimal: Should not need preventive maintenance more than once a year*
    - Overall Vote - 83% Agree (n = 23)
    - Clinicians - 79% Agree (n = 14)
    - Excluding involvement with product development - 83% Agree (n = 23)
  - *Minimal: Should not need preventive maintenance more than 4 times a year (quarterly)*
    - Overall Vote - 85% Agree (n = 20)

- Clinicians - 92% Agree (n = 12)
- Excluding involvement with product development - 85% Agree (n = 20)

- **Replacement Parts and Consumables**
  - Given the discussion on Preventive Maintenance Interval highlighted above, participants noted that this characteristic was too detailed and proposed removing from the final TPP as it would be more applicable to a specification. In light of this, further information on the extensive list of replacement parts recommended in the WHO-UNICEF Technical Specifications and Guidance for Oxygen Therapy Devices [6, p. 94] is included below:

ACCESSORIES, CONSUMABLES, SPARE PARTS, OTHER COMPONENTS		
25	Accessories (if relevant)	The unit shall include internally and externally mounted filters for cleaning the air intake.<br>All user-removable filters shall be cleanable. Cleaning instructions for filters shall be included in the instructions for use.<br>For two or more simultaneous paediatric patients: 1 x flowmeter stand with minimum range from 0 to 2 L/min.<br>Kink-resistant oxygen tubing with standard connectors (15 m each).<br>2 x adult cannula with 2 m kink-resistant oxygen tubing with standard connectors.<br>4 x infant cannula with 2 m kink-resistant oxygen tubing with standard connectors.<br>4 x neonatal cannula with 2 m kink-resistant oxygen tubing with standard connectors.
26	Sterilization/ disinfection process for accessories (if relevant)	Disinfection for nasal prongs.
27	Consumables/ reagents (if relevant)	5-year supply recommended.<br>1-year supply (adjust quantities per patient load and usage frequency):<br>nasal prongs or nasal catheters (each size for adult, child, infant);<br>child nasal prongs: distal diameter: 1–2 mm;<br>child/infant catheters: 6 or 8 French gauge.
28	Spare parts (if relevant)	Internal and external filters and spare parts for user fitting (as described in user manual), including:<br>parts supply, including all necessary filters, for 2 years' operation at 15 hours per day.<br>1 x spare battery set for alarm system (if applicable).<br>1 x spare mains power cable, length ≥ 2.5 m.<br>2 x replacement sets of spare fuses (if non-resettable fuses are used).<br>DISS to 6 mm barbed adaptor for each outlet (if relevant).<br>Bidder must give a complete list of the specific spare parts included in their bid.<br>Other spares that may be needed: circuit breaker, printed circuit board, sieve beds, compressor service kit, valves, wheels, motor capacitor, flowmeters and fan.<br>(Spare parts are not interchangeable between devices of different brands and models, and can vary in their design and lifetime. Medical units to select spare parts ensuring compatibility with the brand and model of the equipment.)
29	Other components (if relevant)	N/A

- **User Skill Level**
  - Participants noted that oxygen concentrators were often used by a wide variety of health workers and therefore, the skill level should be "minimal to none" for both the Optimal and Minimal. Consensus was achieved in the room and no vote was taken. Several participants noted that an oxygen concentrator is a medical device whose output is a drug which can be dangerous if not used properly.
  - *Minimal: Minimal to none (same as Optimal)*

- **Power Consumption**
  - There was ample discussion on the power consumption levels. Product developers noted that all commercial machines use a similar amount of power. International agencies commented that there are recommendations in place in the WHO-UNICEF Technical Specifications and Guidance for Oxygen Therapy Devices [6, p. 94] on appropriate power consumption, which states power efficiency <70W/L/min. Participants agreed that oxygen concentrators should be as energy efficient as possible and power consumption should be proportionate with use.
  - There was agreement in the room that all characteristics relating to Utility Requirements (e.g., Back-up Battery; Battery Power; Batteries; Voltage; Power Requirement; Maximum Power Consumption; Response During Power Outage; Surge Protection, Electrical Plug) be reviewed and harmonized following the TPP meeting.
  - *Optimal: <275 W at 5 LPM*
  - *Minimal: Scales with delivery output — i.e., consumes less power at lower flow rates*
- **Instrument Pricing**
  - There was disagreement on the Minimal characteristic for instrument pricing. Clinicians stressed the importance of reducing the price to increase access. Participants noted that the average cost of an oxygen concentrator in the market is anywhere from $500 - $1,600 ex-works. Product developers agreed that at a price point of $1,600 ex-works, it would be reasonable from a technical perspective to meet the Minimal characteristics outlined in the TPP.
  - *Minimal: <$1600 ex-works*
- **Voltage**
  - Consensus was achieved in the room that since voltage requirements vary based on local conditions, users need to have the ability to use the machine based on their geographic location. Product developers noted that from a technical standpoint, it is not challenging to manufacture a product for one, or the other, voltage. However, only a stabilizer can allow a machine to do both 50 and 60 Hz.
  - There was agreement in the room that all characteristics relating to Utility Requirements (e.g., Back-up Battery; Battery Power; Batteries; Voltage; Power Requirement; Maximum Power Consumption; Response During Power Outage; Surge Protection, Electrical Plug) be reviewed and harmonized following the TPP meeting.
  - *Optimal and Minimal: Model must match the voltage and frequency of the purchasing country's local power grid (e.g., 110-120 VAC at 60 Hz or 220-240 VAC at 50 Hz)*

The following characteristic was not discussed at the TPP Consensus Meeting, however, it was determined that a new characteristic should be added to the TPP with the following justification:

- **Minimal Flow Rate**
  - Some Oxygen Concentrators will not operate below a minimum flow rate. The requirement in the Flow Meter characteristic for flow meter increments of 0.5 LPM only applies above the minimum flow rate of the device. For example, if a device's flow range is 2 LPM – 10 LPM, it is not possible to set the flow to 0.5 LPM, 1 LPM or 1.5 LPM. Rather, it is only possible to set the flow rate from 2 LPM onwards. For neonates, this is relevant if a flow splitter is not being used. If you cannot set to lower flows and there is no flow splitter being used, an Oxygen Concentrator will not prove useful for this neonate population group.

- *Optimal: 0.5 LPM (if used without a flow splitter)*
- *Minimal: 2 LPM*

The following characteristics were not discussed at the TPP Consensus Meeting explicitly, however, additional comments were received and incorporated into the discussion:

- **Oxygen Purity**
  - With regard to the Oxygen Purity range, Pre-Meeting survey voting achieved consensus for the Optimal and Minimal characteristic to be (93% ±3%). A theme emerged in the comments though expressing the need to narrow or broaden this range. While pharmacopoeia's guidelines for Oxygen specify 93%, one participant noted that this guideline is "not for individual concentrators". WHO's existing technology specification for concentrators (2015) [47] as well as ISO's 80601-2-69 specified that low oxygen concentration technical alarm condition shall activate before the concentration drops below 82% volume fraction [72]. International agencies commented that the characteristic should note applicability "at all flow settings" since "Some manufacturers will state different purities for different flow ranges, with lower max purity at the highest flow setting (e.g., 95% at 1 LPM, but 90% at 5 LPM)."
  - *Optimal and Minimal: 93% ±3%*
- **Oxygen Monitor**
  - One theme that arose in the Pre-Meeting survey was confusion on why there were three ranges of oxygen concentration in the Optimal characteristic: "Visual and audible status indicator for three ranges of oxygen concentration preferably with color coding for early warning." One participant clarified that this due to the three ranges indicated in pharmacopoeia: 99, 93 and then 'not for individual concentrators' [73]. International agencies highlighted the importance of clarifying what normal status would be for the audible status indicator.
  - *Optimal: Visual and audible status, preferably with color coding for early warning*
  - *Minimal: Visual and audible status*
- **Durability and Robustness**
  - In the Pre-Meeting survey, we received an additional comment highlighting the importance of considering both heat and humidity simultaneously. Peel's study "Evaluation of oxygen concentrators for use in countries with limited resources" emphasizes the importance of testing manufacturer claims [48]. Additionally, the WHO-UNICEF Technical Specifications and Guidance for Oxygen Therapy Devices [6, p. 95] highlight certain environmental requirements:
    - "Capable of being stored continuously in ambient temperature from 0 °C to 40 °C, RH from 15% to 95% and elevation from 0 to at least 2000 m.
    - Capable of supplying the specified oxygen concentration continuously in ambient temperature from 10 to 40 °C, RH from 15% to 95%, simultaneously, and elevation from 0 to at least 2000 m.
    - For operation at elevations higher than 2000 m, environmental requirements are less stringent; performance characteristics at such altitudes must be stated."
  - *Optimal: Harsh ambient condition, temperature 5-45 °C, humidity 15% to 95%, dusty air, elevation >=2000 meters*
  - *Minimal: Temperature 10-40 °C, humidity 15%-95% elevation up to 2000 meters*

- The following Product Specific ISO Standards were highlighted in the Pre-Meeting survey responses:
  - The product(s) shall conform to the standards stipulated by the International Organisation for Standardisation (ISO) and/or equivalent standards as recognized by any IMDRF member
  - Standards applicable to the product:
    - ISO 80601-2-69:2014 Medical electrical equipment – Part 2–69: Particular requirements for basic safety and essential performance of oxygen concentrator equipment.
    - IEC 60601-1:2012 Medical electrical equipment – Part 1: General requirements for basic safety and essential performance.
    - IEC 60601-1-2:2014 Medical electrical equipment – Part 1–2: General requirements for basic safety and essential performance – Collateral Standard: Electromagnetic disturbances – Requirements and tests.
    - IEC 60601-1-6:2013 Medical electrical equipment – Part 1–6: General requirements for basic safety and essential performance – Collateral standard: Usability.
    - IEC 60601-1-8:2012 Medical electrical equipment – Part 1–8: General requirements for basic safety and essential performance – Collateral Standard: General requirements, tests and guidance for alarm systems in medical electrical equipment and medical electrical systems.
    - IEC 60601-1-9:2013 Medical electrical equipment – Part 1–9: General requirements for basic safety and essential performance – Collateral Standard: Requirements for environmentally conscious design.
    - IEC 60601-1-11:2010 Medical electrical equipment – Part 1–11: General requirements for basic safety and essential performance – Collateral Standard: Requirements for medical electrical equipment and medical electrical systems used in the home health-care environment.
    - ISO 13485:2003 Medical devices – Quality management systems – Requirements for regulatory purposes (Australia, Canada and EU).
    - ISO 14971:2007 Medical devices – Application of risk management to medical devices.
    - Compliance with ISO 8359 may be considered.

DELPHI-LIKE SURVEY: OXYGEN CONCENTRATOR

Table 20: Delphi-like survey results for Oxygen Concentrator TPP prior to Consensus Meeting (data as of Oct 25, 2019)

	**Optimal**		**Minimal**		
**Characteristic**	**Optimal requirement**	**% agreement (n size)**	**Minimal requirement**	**% agreement (n size)**	**Collated comments from Delphi-like survey**
**Intended Use**	Optimal: To provide medical oxygen for	**100%**<br>**n = 30**	Minimal: Same as Optimal.	**100%**<br>**n = 30**	2 comments as summarized below<br>• Consider re-phrasing 'medical oxygen' to 'oxygen for clinical application in a healthcare setting'

	Optimal		Minimal		
	use in a healthcare setting.				
**Target Operator**	Optimal: For use in low- and middle-income countries by a wide variety of clinicians, including nurses, clinical officers, and pediatricians.	**97%**<br>**n = 31**	Minimal: Same as Optimal	**100%**<br>**n = 29**	3 comments as summarized below<br>• Pediatrician / Clinical Officer may decide the settings, but the nurse is the one most likely to use the machine<br>• Separate user for repairing the device / changing the filter
**Target Population**	Optimal: Neonates (<28 days)	**65%**<br>**n = 31**	Minimal: Same as Optimal.	**62%**<br>**n = 29**	14 comments as summarized below<br>• Theme: Broaden age range but consider neonates (e.g., flow rates)<br>• Include older infants, children, mothers<br>• Need to consider Flow Meter and Flow Rate characteristics
**Target Setting**	Optimal: Hospitals in low-resource settings	**77%**<br>**n = 31**	Minimal: Same as Optimal.	**77%**<br>**n = 30**	10 comments as summarized below<br>• Theme: Broaden vs. Narrow Target Setting<br>o Lower levels of the health system where supply chain does not provide oxygen cylinders and resources adequate<br>o Potentially higher income counties<br>o "On the one hand the mortality tends to be at the village level or first-contact health facility, so we should aim for the smallest health facilities that care for in-patients. On the other hand, the level of skill, training and other resources needed to care for neonates may make it impractical to go beyond the largest sub-district health centres. Whatever level we choose, it is worthwhile

	Optimal		Minimal		
					thinking about some technology to help stabilize and transport a neonate who needs referral to a more central level."<br>o Minimal: hospital in resource-limited settings, Optimal: health centres (primary)
**International Standard**	Optimal: ISO 13485:2016 Medical devices – Quality management systems -- Requirements for regulatory purposes.	**75%**<br>**n = 20**	Minimal: Same as Optimal.	**78%**<br>**n = 18**	10 comments as summarized below<br>• Theme: Add to Additional International Standards vs. Irrelevance<br>• Consider inclusion of ISO 80601-2-69 (current: 2014 though under review) is unique to concentrators, title: Medical electrical equipment -- Part 2-69: Particular requirements for basic safety and essential performance of oxygen concentrator equipment.<br>• Consider adding check additional standards from Family 11 - https://www.iso.org/ics/11/x/<br>• Requirement for CE marking<br>• Alternatively, some respondents commented that having ISO13485 does not necessarily lead to good performance in low-resource settings
**Regulation**	Optimal: CE marking or US FDA Clearance	**72%**<br>**n = 25**	Minimal: Same as Optimal.	**70%**<br>**n = 23**	11 comments as summarized below<br>• Theme: Add more flexibility v. irrelevance of characteristic<br>• Consider additional 'or' options:<br>o Other Stringent Regulatory Authorities – Japan or Australia or Canada<br>o Consider regulatory bodies of Low- and Middle-Income Countries<br>Some respondents did not think that regulatory approval necessarily translated to good performance.
**Flow Meter**	Optimal: At least 2 with each 0 to 5 SLPM flow meter, min	**75%**<br>**n = 28**	Minimal: At least 1 with 0 to 8 SLPM flow meter, min	**62%**<br>**n = 26**	10 comments as summarized below<br>• Change SLPM to LPM<br>• Theme: Merge Flow Meter and Flow Rate characteristics for clarity

	Optimal		Minimal		
	incremental<br>0.5 SLPM		incremental<br>0.5 SLPM		• Theme: Higher Flow (10 lpm) Oxygen Concentrators have advantages but may create confusion as well between flow meter and flow splitter<br>o Could be used as a back up flow generator for bCPAP during power outages<br>o If you have a splitter, could get oxygen to more babies<br>• Theme: Need smaller increments<br>o Neonates who are on long term oxygen need minimum titration capability of 1/4 liter (especially neonates with sufficient prematurity to cause chronic lung disease ... the ability to do small titrations to get them off oxygen prior to day of life 30 is important)<br>o “In level 2 nurseries we have a few modified flow meters that will let you titrate at as little as 1/8 of a liter in order to help us wean kids off oxygen”<br>• Theme: Other Suggested Alternatives<br>o “Should be at least 2 flow meters for efficiency”<br>o “I don't think we should encourage the inefficient way concentrators are typically used - moved around the ward and used for one or two children at a time”<br>o “Low-pressure piping system to distribute oxygen from a unified concentrator/low-pressure store/backup cylinder system (automatically choosing the cheapest source available at the time). So we don't really care what the concentrator's flow meter is like, and we see no value in having two flow meters. It is not widely known that a typical concentrator uses the same amount of electricity whether it is running at 0.5 LPM or 10 LPM. There is no efficiency gain in running below full capacity, so we prefer to (i) store 'excess' oxygen for use when the concentrator is off, and (ii) automatically switch the concentrator off when the store is full, to minimize electricity use.”

	Optimal		Minimal		
**Flow Rate**	Optimal: 10 SLPM	**69%**<br>**n = 29**	Minimal: 8-10 SLPM	**50%**<br>**n = 28**	15 comments as summarized below<br>• Theme: Lack of clarity on why such high LPM for neonates (likely due to separation of Flow Meter and/or Splitter)<br>o I choose higher flow + splitter so that oxygen could be administered to more kids. Ideally you could do this and still titrate at least 1/2-1/4 LPM for individual children<br>o 5 LPM (most popular mention)<br>o "No neonate requires 10 LPM"<br>o Minimal should be 8 LPM<br>o 2 LPM would be helpful<br>o Minimal flow rate can be less than 8 SLPM especially for neonates
**Time to Reach 95% of Specified Performance** *(corrected from 'Pressure')*	Optimal: < 5 Min	**85%**<br>**n = 27**	Minimal: <30 Min	**46%**<br>**n = 26**	15 comments as summarized below<br>• Theme: 30 minutes is too long<br>o 5 minutes already met with most commercially available devices<br>o 10 minutes<br>o 3 minutes
**Oxygen Purity**	Optimal: 93% +-3%	**80%**<br>**n = 30**	Minimal: Same as Optimal.	**75%**<br>**n = 28**	10 comments as summarized below<br>• Theme: Respondents expressed need to narrow or broaden this range<br>o This aligns with a few pharmacopoeia's guidelines for Oxygen 93<br>o WHO's existing tech specs for concentrators (2015) as well as ISO's 80601-2-69 have indicated greater than or equal to 82% (so alarms set etc.)<br>o FiO2 achieved from 95% would be +/- 45.5%, and FiO2 achieved from 82% would be +/-41%<br>o According to ECRI, most can meet 90% at all flow settings.<br>o For minimal, this could be relaxed to 90% +/- 3

	Optimal		Minimal		
					o This may be too strict for actual testing. As reported by manufacturer's this is fine, but the level varies depending on the flow rates and other external environmental factors
**Alarms**	Optimal: Audible and/or visual alarms for high temperature, flow rate and pressure	74%<br>n = 31	Minimal: Same as Optimal.	67%<br>n = 30	12 comments as summarized below<br>• Theme: A range of alarms were mentioned<br>o Low battery or power failure (alarm if power failure) - needs immediate response by healthcare worker<br>o Oxygen purity (alarm if <85% or < 82%) - needs rapid response by healthcare worker<br>o High or low pressure/flow/temperature (where response is to call a technician)<br>• Note: Some machines use an internal 9V battery for the alarms. If it is not replaced (as is common) then the alarms do not work
**Indicators**	Optimal: Clearly labeled or marked with pictures and language. Audible alerts and diagnostic indicator where possible	73%<br>n = 30	Minimal: UI easy to understand, numbers and displays clearly visible	86%<br>n = 28	8 comments as summarized below<br>• Theme: Provide additional clarity on what is meant by diagnostic indicator<br>o Diagnostic indicators + informing necessary action(s) are desirable<br>o Change diagnostic indicator to low oxygen indicator<br>o Electrical power input requirements (voltage, frequency, socket type)
**Mobility**	Optimal: Four antistatic swivel castors, two with brakers, integrated handle	70%<br>n = 27	Minimal: Four wheels	73%<br>n = 26	10 comments as summarize below<br>• Theme: Variation in perceived advantages of wheels and breaks<br>o UNICEF-WHO spec: Whole unit to be movable with wheels on at least two feet<br>o Not worth it if increases cost<br>o Always swivel wheels<br>o "Is there space for breaks? Wheels are so small!"

	Optimal		Minimal		
					o We discourage the moving of concentrators around the ward. In some of our installations we have had to remove or immobilise the wheels<br>o Important to be easily mobile to accommodate range of clinical situations and to move around neonatal units<br>o No concentrators have brakes on them - it is another potential failure point on the device. Suggest making minimal and Optimal the same at "four wheels"
**Oxygen Monitor**	Optimal: Visual and audible status indicator for three ranges of oxygen concentration preferably with color coding for early warning.	**82%**<br>**n = 28**	Minimal: Visual and audible status.	**89%**<br>**n = 28**	9 comments as summarized below<br>• Theme: Confusion as to why three ranges of oxygen vs. two<br>o Oxygen purity <85%<br>o Oxygen purity above or below 90%
**Oxygen Outlet**	Optimal: Recessed, replaceable metal barbs	**85%**<br>**n = 26**	Minimal: Recessed, replaceable metal or plastic barbs	**84%**<br>**n = 25**	7 comments as summarized below<br>• Nothing currently meets Optimal<br>• Plastic is easily damaged
**Sound Level**	Optimal: ≤50 decibels	**84%**<br>**n = 25**	Minimal: 50 decibels	**65%**<br>**n = 23**	12 comments as summarized below<br>• Theme: <50 dB easily obtainable by current machines vs. nothing currently meets 50 dB<br>• EC 60601-1-8 has, in the latest amendment 1 issued in 2012, a number of measurements are required according to Annex F in ISO 3744, with the measurements averaged

	Optimal		Minimal		
**Decontamination**	Optimal: Reduced recessed areas and need for specialized cleaning procedures or products	**80%** **n = 30**	Minimal: Easy to clean flat surfaces, compatible with common disinfecting agents	**97%** **n = 29**	9 comments as summarized below<br>• Theme: Access to filter and or humidity / water container<br>• Theme: Optimal and Minimal should be switched
**Weight**	Optimal: <30 kg	**79%** **n = 29**	Minimal: Same as Optimal.	**81%** **n = 27**	8 comments as summarized below<br>• Theme: Variability in perceived advantages of weight<br>o Seldom needs to be moved<br>o WHO-UNICEF interagency spec is less than 27kg so Optimal could be less<br>o Weight is not important except for freight costs<br>o Ideally less than 20 kg or 23 kg could be carried by staff
**User Instructions**	Optimal: User manual and additional training materials (checklists, videos, guides) in English and local language. Attached to device with labels and markings where possible.	**72%** **n = 29**	Minimal: Instruction manual provided.	**68%** **n = 28**	11 comments as summarized below<br>• A variety of hard and soft copy materials mentioned with particular mentions of difficulty in reading a user manual and preference for videos so people can see vs. read<br>• All claims must be filed with the regulatory dossier, so this is not as straight forward as a simple translation. Appropriate, professional translations are a must and are costly to the manufacturer. Additionally, local language varies greatly across a country and is often-times not even the official language of the country and so this may not be a reasonable ask of manufacturers.<br>• English, French and Portuguese most critical languages

	Optimal		Minimal		
**Durability and Robustness**	Optimal: Harsh ambient condition, temperature 5-45 °C, humidity 15% to 95%, dusty air, elevation >=2000 meters	**88%**<br>**n = 26**	Minimal: temperature 10-40 °C, humidity 15%-95% elevation up to 2000 meters	**88%**<br>**n = 25**	14 comments as summarized below<br>• Theme: Additional Durability and Robustness considerations mentioned<br>o Dust<br>o Dirty electricity<br>o As demonstrated by Peel, important to test manufacturer claims: https://onlinelibrary.wiley.com/doi/full/10.1111/anae.12260<br>• Theme: May be too aggressive and would require existing manufacturers to resubmit for regulatory which is not likely<br>• WHO-UNICEF spec:<br>o Capable of being stored continuously in ambient temperature from 0°C to 40°C, RH from 15% to 95% and elevation from 0 to at least 2000 m<br>o Capable of supplying the specified oxygen concentration continuously in ambient temperature from 10 to 40 °C, RH from 15% to 95%, simultaneously, and elevation from 0 to at least 2000 m<br>o For operation at elevations higher than 2000 m, environmental requirements are less stringent; performance characteristics at such altitudes must be stated
**Usage Meter**	Optimal: Non-resettable digital or analog meter displaying cumulative hours of operation.	**85%**<br>**n = 26**	Minimal: Same as Optimal.	**80%**<br>**n = 25**	4 comments as summarized below<br>• Could be useful to re-set the timer after changing the sieve bed and other spare part

	Optimal		Minimal		
**Cleaning Interval**	Optimal: None Required.	66%<br>n = 29	Minimal: Weekly cleaning of external course filter.	75%<br>n = 28	16 comments as summarized below<br>• Theme: Optimal and minimal are not realistic<br>• Optimal cleaning interval is required as dust can accumulate<br>• None required is not realistic. The concentrators that have claimed that previously have failed to deliver. I worry this will provide false reassurance. I suggest keeping Optimal same as minimal<br>• Optimal: not more than weekly cleaning of easily accessible external filter<br>• Minimal: not more than monthly cleaning of other filters/components<br>• WHO-UNICEF<br>o Device exterior to be wiped effectively with a mild solution of detergent or cleaning agent (weekly), without connection to mains power<br>o Gross particle filter to be cleaned effectively when removed and washed with soap and water (weekly)<br>o Do not clean with alcohol<br>o (User care needed more often in very dusty environments)
**Preventive Maintenance Interval**	Optimal: Minimal to none	64%<br>n = 28	Minimal: Every 24 months	68%<br>n = 25	14 comments as summarized below<br>• Theme: Optimal and minimal are not realistic<br>• Suggested Ranges<br>o 3 months<br>o 6 months<br>o 12 months<br>o Regular<br>o As per manufacturer recommendation<br>o 24 month interval is not often enough to be realistic for any current products<br>o Minimal to none is not realistic<br>• Provide suggestions for preventative maintenance<br>o Test power failure alarms

	Optimal		Minimal		
					o Measure operating pressure with pressure test gauge<br>o Measure oxygen concentration with a calibrated oxygen analyzer<br>o Repair internal components as needed<br>o Maintain spare-parts inventory
**Replacement Parts and Consumables**	Optimal: None required	**60%**<br>**n = 25**	Minimal: None required for 24 months	**71%**<br>**n = 24**	16 comments as described below<br>• Theme: Optimal and minimal are not realistic<br>• Suggested Ranges and Parts<br>o Not possible to have no parts and consumables replacement needed<br>o As per manufacturer recommendation<br>o Every 3 months<br>o 6-12 monthly replacement of filters, and >24 monthly other spare parts<br>o Fuses<br>o Recommend five years of filters and spare parts be organized at the time of purchase and replaced when used<br>▪ Internal and external filters and spare parts for user fitting (as described in user manual), including:<br>• Parts supply, including all necessary filters, for 2 years operation at 15 hours per day.<br>• 1 x spare battery set for alarm system (if applicable).<br>• 1 x spare mains power cable, length 2.5 m.<br>• 2 x replacement sets of spare fuses (if non-resettable fuses are used)<br>• DISS to 6mm barbed adaptor for each outlet (if relevant)<br>o Bidder must give a complete list of the specific spare parts included in their bid

	Optimal		Minimal		
					o Other spares that may be needed: circuit breaker, printed circuit board, sieve beds, compressor service kit, valves, wheels, motor capacitor, flowmeters and fan<br>o (Spare parts are not interchangeable between devices of different brands and models, and can vary in their design and lifetime. Medical units to select spare parts ensuring compatibility with the brand and model of the equipment.)
**Warranty**	Optimal: 5 years	**85%**<br>**n = 27**	Minimal: 1 year	**65%**<br>**n = 26**	11 comments as summarized below<br>• Theme 1 year too short | 5 years too long<br>• Suggested Ranges:<br>o 2 years<br>o "To honor a 5 year warranty, you will have to have strong in-country representation. An extended warranty is a degree of assurance of the above, and this will come at a cost. Manufactures of concentrators willing to extend a warranty from 2-5 do so at a cost. What might be more useful is that during any procurement, consideration be given to establishing a SLA with an in-country rep. In this case, you can take care of any major PPM requirements, as well as "swap out" in the event of a break-down, and there is no discussion of warranties and no need for spares and an in-country source for consumables."
**Technical Skill Maintenance**	Optimal: Minimally trained technician	**76%**<br>**n = 29**	Minimal: Trained technician with training in basic operation and maintenance	**89%**<br>**n = 27**	6 comments as summarized below<br>• Lack of clarity on what minimally trained technician means<br>• How do we quantify or measure this?

	Optimal		Minimal		
**Tools Required**	Optimal: No specialized tools required	**79%**<br>**n = 28**	Minimal: Minimal specialized tools for sieve bed and filter replacement	**81%**<br>**n = 27**	7 comments as summarized below<br>• Minimal should still be 'no specialized tools'<br>• Filter replacement should require 'no specialized tools'<br>• Will always require specialized tools, otherwise, anyone can open and tamper<br>• Manufacturer to specify which tools are required to perform maintenance tasks:<br>o Test power failure alarms<br>o Measure operating pressure with pressure test gauge<br>o Measure oxygen concentration with a calibrated oxygen analyzer<br>o Repair internal components as needed<br>o Maintain spare-parts inventory
**User Skill Level**	Optimal: Minimal to none.	**68%**<br>**n = 28**	Minimal: Same as Optimal.	**67%**<br>**n = 27**	8 comments as summarized below<br>• Lack of clarity on what minimal means<br>• None does not make sense
**Electrical Plug**	Optimal: Universal conversion power adapter, compatible with local power outlet, rated above amperage voltage requirements	**79%**<br>**n = 28**	Minimal: Compatible with local power outlet, rated above amperage voltage requirements	**93%**<br>**n = 27**	8 comments as summarized below<br>• Theme: Additional suggestions provided<br>o "Universal" adaptor will not convert 60Hz equipment to 50Hz. Machine will fail within 3 months<br>o This is always a very solvable issue. It's the actual voltage and FREQUENCY of device that's most important, as well as voltage stabilizers and surge suppressors<br>o Locally compatible plug preferred over conversion adapter to avoid misuse<br>o Need surge (up to 330 V) and dip protection
**Filters**	Optimal: Replaceable washable reusable	**86%**<br>**n = 29**	Minimal: Same as Optimal	**86%**<br>**n = 28**	5 comments as summarized below<br>• Theme: Additional suggestions provided:

	Optimal		Minimal		
					o This is adequate for the external filter. But usually there is a fine particle filter internally that is typically made of felt and needs replacement, especially after the dusty season<br>o Which filters?<br>▪ Bacteria filter definitely cannot be washed and should not really need replacing<br>▪ Gross particle definitely washable & reusable<br>▪ Air intake (compressor) filter is HEPA and washing them is not a possibility as it damages the weave or fibres that make it effective in the first place<br>o Incompatible with Cleaning Interval
**Power Consumption**	Optimal: 275 W at 5 SPLM	**68%**<br>**n = 19**	Minimal: Scales with delivery output — i.e., consumes less power at lower flow rates.	**65%**<br>**n = 20**	10 comments as summarized below<br>• Theme: Optimal and minimal are not realistic<br>• Nothing currently meets these requirements<br>• 5 LPM inconsistent with 8-10 LPM mentioned above
**Surge Protection**	Optimal: Integrated	**93%**<br>**n = 29**	Minimal: External	**79%**<br>**n = 28**	12 comments as summarized below<br>• Theme: Internal Surge Protection is not necessarily ideal<br>o Quality of surge protector depends on how terrible the power is<br>o For many African contexts, an adequate surge protector will weigh as much as the concentrator itself and be quite bulky and cost <200USD<br>o I worry this might encourage manufacturers to put in low quality surge protectors that won't actually do the job<br>o More costly?<br>• Theme: External Surge Protection is not necessarily ideal either<br>o External can be damaged, stolen, misapplied for other equipment

	Optimal		Minimal		
					o More costly?<br>• Theme: Surge protection not as important as voltage<br>o In our experience surge protection is less important than lifting low voltages towards the optimum.
**Voltage**	Optimal: 110-240 50-60hz	**83%**<br>**n = 23**	Minimal: 220-240 50-60hz	**71%**<br>**n = 21**	8 comments as summarized below<br>• As per local requirements
**Instrument Pricing**	Optimal: <$500 ex-works	**83%**<br>**n = 24**	Minimal: <$1600 ex-works	**61%**<br>**n = 23**	9 comments as summarized below<br>• Theme: Minimal seems high for an Oxygen Concentrator unless you have a flow splitter; $1,000<br>• Theme: Don't buy cheap; if you do, check manufacturers claims independently

Figure 20: Summary of organizational affiliation for Oxygen Concentrator TPP from Delphi-like Survey prior to Consensus Meeting (data as of Oct 25, 2019)

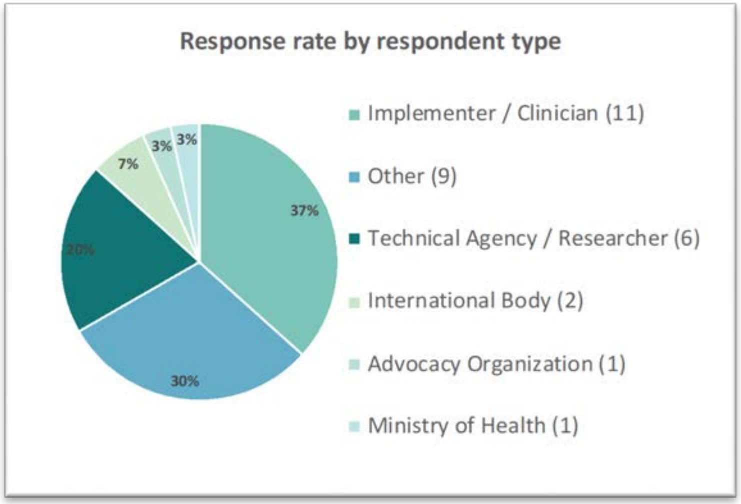

Respondent type	Percentage
Implementer / Clinician (11)	37%
Other (9)	30%
Technical Agency / Researcher (6)	20%
International Body (2)	7%
Advocacy Organization (1)	3%
Ministry of Health (1)	3%

Figure 21: Summary of response rate by country for Oxygen Concentrator TPP from Delphi-like Survey prior to Consensus Meeting (data as of Oct 25, 2019)

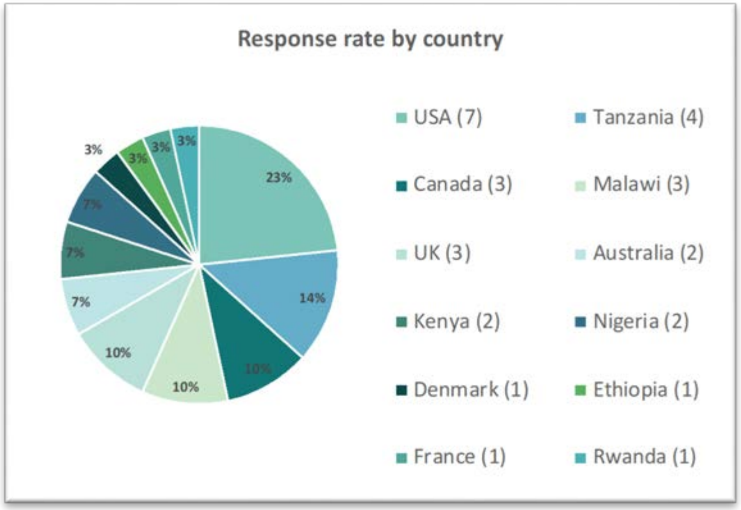

Country	Percentage
USA (7)	23%
Tanzania (4)	13%
Canada (3)	10%
Malawi (3)	10%
UK (3)	10%
Australia (2)	7%
Kenya (2)	7%
Nigeria (2)	7%
Denmark (1)	3%
Ethiopia (1)	3%
France (1)	3%
Rwanda (1)	3%

INTRODUCTION: PULSE OXIMETER (CONTINUOUS)

Pulse oximeters use a non-invasive sensor to measure pulse rate (PR) and blood oxygenation levels (SpO2) (i.e., percentage of oxygenated hemoglobin in arterial blood). While pulse oximeters do report pulse rate, their primary purpose and utility is to detect SpO2 in infants. According to the World Health Organization, pulse oximetry is the most accurate non-invasive method for detecting hypoxemia. It is used to measure the percentage of oxygenated hemoglobin in arterial blood (SpO2). The pulse oximeter consists of a computerized unit and a sensor probe which is attached to the patient's finger, toe, or earlobe. The oximeter displays the SpO2 with an audible signal for each pulse beat, a pulse rate and, in many models, a graphical display of the blood flow past the probe (the plethysmographic or pulse wave). The technology is robust and cost effective. Pulse oximeters can be used to both detect and monitor hypoxemia, make more efficient use of oxygen supplies, and improve patient monitoring [32].

Low SpO2 levels can indicate that an infant is in respiratory distress and monitoring SpO2 is important in the neonatal period as it can indicate the need for immediate, critical care interventions. Additionally, SpO2 monitoring is critical for infants receiving oxygen therapy or continuous positive airway pressure (CPAP) therapy. Low SpO2 levels during oxygen or CPAP therapy can indicate that escalation or additional care is required. On the other hand, if SpO2 remains too high (>95%) for too long (often a side effect of pure oxygen therapy), newborns can suffer from preventable disability including retinopathy of prematurity (ROP), a condition that can cause permanent blindness, and chronic lung disease [39,40]. One other consideration when using a pulse oximeter is that the reading may not be as accurate in specific situations (e.g., when a neonate's peripheries are cold, when the neonate is anemic, etc.).

FINAL TPP: PULSE OXIMETER (CONTINUOUS)

Table 21: Final TPP for Pulse Oximeter

Final target product profile for Pulse Oximeter (Continuous)		
**Characteristic**	**Optimal**	**Minimal**
SCOPE		
**Intended Use**	To continuously monitor oxygen saturation (SpO2) and pulse rate (PR) for neonatal patients	

**Target Operator**	For use in low- and middle-income countries by a wide variety of clinicians, including nurses, clinical officers, and pediatricians	
**Target Population**	Neonates (born at any gestational age and require ongoing care)	
**Target Setting**	Hospitals in low-resource settings, but, may be used in health facilities based on country guidelines	Hospitals in low-resource settings
SAFETY AND STANDARDS		
**Quality Management** [1]	ISO 13485:2016 Medical devices – Quality management systems -- Requirements for regulatory purposes	
**Regulation**	At least one of: CE marking, approved by US FDA or another stringent regulatory body of a founding member of IMDRF (e.g., Japan or Australia or Canada)	
TECHNICAL CHARACTERISTICS		
**Pulse rate**	25-250 bpm	30-240 bpm
**Pulse rate accuracy**	± 3 bpm	
**Pulse rate resolution**	1 bpm	
**Sp02 Accuracy**	± 2%	± 3%
**Sp02 Range**	0-100%	70-100%
**Alarms**	Visual and Auditory	Auditory
**Alarm Limits - PR**	Adjustable	80-180 bpm OR 100-180 bpm [2]
**Alarm Limits - Sp02**	Adjustable	
**Continuous Measurement**	Yes	
**Patient Interface**	Neonate specific, biocompatible and reusable	
**Size**	Easily moveable, not pocketable, can be secured	Handheld with dock
**Weight**	<500 grams, portable	
PURCHASING CONSIDERATIONS		
**Accessories**		

**Consumables**	>12 months before required	>6 months before required with 2 neonatal probes included in package
**Instrument Pricing**	<$150 ex-works	<$250 ex-works
**Consumable Pricing**	<$50 per year ex-works (two probes)	<$80 per year ex-works (two probes)
UTILITY REQUIREMENTS		
**Power Source**	Mains with rechargeable battery	Mains with rechargeable battery
**Battery**	Rechargeable battery, >24hr on single charge	Rechargeable battery, >6hr on single charge [3]
**Voltage**	None	Model must match the voltage and frequency of the purchasing country's local power grid (e.g., 110-120 VAC at 60 Hz or 220-240 VAC at 50 Hz)
TRAINING AND MAINTENANCE		
**User Instructions**	User manual and additional training materials (checklists, videos, guides) in at least one national official language for the country of intended use. Attached to device with labels and markings where possible	User manual provided in at least one national official language
**Training Required**	Minimal	
**Warranty**	5 years	1 year
**Decontamination**	Easy to clean with common disinfecting agents	
**Usage Meter**	Digitally stored record displaying cumulative hours of operation	Digitally stored record displaying 50 previous readings or >50 hours

[1] *There was not 75% voting agreement on the Minimal characteristic. Please refer to the TPP Report discussion for additional detail.*
[2] *There was not 75% voting agreement on this characteristic. Please refer to the TPP Report discussion for additional detail.*
[3] *There was not 75% voting agreement on this characteristic. Please refer to the TPP Report discussion for additional detail.*

To arrive at the final TPP for Pulse Oximeter (Table 21), we conducted a pre-meeting survey. Based on the pre-meeting survey results (Table 22), characteristics that achieved below 75% agreement were prioritized for discussion at the Consensus Meeting. Upon commencement of the discussion, it was agreed that the TPP in question for discussion was clarified as a Continuous Pulse Oximeter. The need for a separate TPP for a Spot-Check Pulse Oximeter was identified. An overview of the discussion is included below.

- **Pulse Rate**
  - Clinicians in the room agreed that the Minimum characteristic should be aligned with the WHO-UNICEF Technical Specifications and Guidance for Oxygen Therapy Devices [6, p. 126]. Note that for the Pulse Rate Accuracy, the WHO-UNICEF Technical Specifications and Guidance for Oxygen Therapy Devices [6, p. 126] specify ± 3 bpm. International NGOs suggested that manufacturers be more transparent in sharing clinical outputs on data accuracy so that buyers can have assurance of claim. While consensus was achieved on the values of measurement, clinicians emphasized that guidance or protocols for behavior if a value falls outside of these ranges is not currently defined and would be helpful.
  - *Minimal: 30-240 bpm*
    - Overall Vote - 100% Agree (n = 39)
    - Clinicians - 100% Agree (n = 27)
    - Excluding involvement with product development - 100% Agree (n = 35)
- **Alarms**
  - There was disagreement on whether the Minimal characteristic should require both an auditory and visual alarm. Clinicians discussed that auditory alarms are better at drawing attention, especially when wards may be short-staffed. Product developers confirmed that an auditory alarm was slightly more expensive than a visual alarm and that having both alarms added roughly $3 to the overall cost. Two concerns with auditory alarms were mentioned (alarm fatigue and noise levels impacting baby), however, clinicians agreed that this was a critical alarm and therefore, the benefits of an auditory alarm to stress the importance outweigh the concerns. Following the Consensus Meeting, one participant commented that "Inability to disable alarms for more than 2 min is a critical safety issue. The ability to configure the default alarm is critical. This will address almost all the discussion we had on this issue."
  - *Minimal: Auditory*
    - Overall Vote - 84% Agree (n = 38)
    - Clinicians - 85% Agree (n = 27)
    - Excluding involvement with product development - 86% Agree (n = 35)
- **Alarm Limits – Pulse Rate (PR)**
  - There was disagreement suggesting a wider range for the Minimal characteristic and a discussion of whether the range should be fixed or variable (i.e., users can set the range). Some clinicians felt that the range should be fixed for certain levels of care (e.g., secondary or primary level) while others thought that having a factory setting pre-programed but that could be adjusted would provide flexibility. Some users noted the flexibility would be helpful for trainings and where altitude could present challenges. Clinicians noted that they rarely vary the factory settings (when asked the last time they adjusted the setting, one replied “over four months ago”). Product developers noted that there is no impact to the alarm limits from a technical standpoint. A healthy debate ensued on whether the alarm should sound at 80 bpm or 100 bpm for the lower bound for the Minimal characteristic (agreement in room for 180 bpm for the upper bound). Those in favor of 80 bpm argued “you

don't want the alarm to constantly be going off and contributing to alarm fatigue". Consensus was ultimately not achieved on whether the lower bound should be 80 or 100 bpm.
  - *Optimal: Adjustable*
  - *Minimal: 80-180 bpm OR 100-180 bpm * see discussion above as the voting was split and consensus was not achieved **
  - *Minimal: Fixed value or variable*
    - Overall Vote - 75% voted "fixed" (n = 36)
    - Clinicians - 76% voted "fixed" (n = 25)
    - Excluding involvement with product development - 76% voted "fixed" (n = 34)
  - *Minimal: Lower bound of 80 or 100 bpm*
    - Overall Vote - 59% voted "80 bpm" (n = 27)
    - Clinicians - 59% voted "80 bpm" (n = 22)
    - Excluding involvement with product development - 58% voted "80 bpm" (n = 26)
- **Alarm Limits – Sp02**
  - There was disagreement on the Minimal characteristic with similar commentary on the concern of alarm fatigue ("it is not helpful if the alarm is sounding permanently on a sick child") and the impact of altitude on the lower range limit. There was a discussion reviewing the Pre-Meeting survey comments for the Minimal characteristic:
    - Adjustable: "You want to set the alarm according to the environment; e.g., the altitude might impact the levels you want and normal values of oximetry may be lower"
    - Non-Adjustable: Adjustability of the alarms increase risk of user error and/or use on a different patient population
    - Partially Adjustable: "Should be closed settings not fully adjustable. For example 1) neonate setting 2) infant setting 3) pediatric setting, etc."
  - Consensus was achieved in the room (without a Mentimeter vote) that the range should be adjustable for the Minimum, as well as the Optimal, to provide flexibility based on the patient type.
  - *Minimal: Adjustable*
- **Consumables**
  - Agreement was reached in the room on clarification that the consumables in question were to be specified as two neonatal probes (designed for and tested in newborns). Clinicians in the room commented that two neonatal probes should be included in the package when initially purchased. Product developers noted that measuring by a period of time can be challenging since it's often difficult to prove whether the probes have been used improperly. One consideration was changing the measurement to the strength of the probe rather than the length of time. Furthermore, product developers noted that the cabling on the sensor of the probe is the weakest part and that the lifespan will decrease if twisted around improperly. Some users mentioned a preference for reusable probes while others mentioned that disposable probes "fit better" and were therefore preferred. Consensus was achieved in the room (without a Mentimeter vote).
  - *Minimal: >6 months before required with 2 neonatal probes included in package*
- **Size**

- For the Optimal characteristic, many different configurations were noted including: easily movable; not docked, not "pocketable". Specifically, clinicians commented that the device should be "moveable, but not too small that it can be taken away from the unit". The idea of "chaining" the device in the unit to avoid being moved was mentioned. Clinicians noted that for continuous monitoring, they prefer the display screen to be larger so that it is readable from a certain distance. One participant emphasized that often times, there is limited space available in the NICU and there may be limited table space available for a benchtop device. Therefore, a handheld device that could be mounted to the side of the crib could prove useful.
- *Minimal: Easily moveable, not pocketable, can be secured (same as Optimal)*
    - Overall Vote - 96% Agree (n = 27)
    - Clinicians - 95% Agree (n = 19)
    - Excluding involvement with product development - 96% Agree (n = 26)

- **Usage Meter**
    - There was disagreement on the Minimal characteristic for the usage meter. Product developers noted that digitally storing recorded memory adds a significant cost to the device and for a Minimal standard, this would be too onerous to require manufacturers to include for a small device. From a technical standpoint, the challenge was installing the feature for measurement, not the timing (i.e., how many hours of memory were captured). Clinicians suggested storing for roughly 12 hours (overnight period) or for 6 hours (typical nurse shift). Clinicians were open to other non-digital ways to document the data since a mapping of the digitally stored patient data linked to the true record of the patient chart currently does not exist. There was a discussion as to whether the purpose of usage meter was for manufacturers to record cumulative hours of usage, or, for the clinicians to store historical data recordings. For ISO certification standard, usage data must be stored [49].
    - *Optimal*: Digitally stored record displaying cumulative hours of operation
    - *Minima*l: Digitally stored record displaying 50 previous readings or >50 hours
    - *Minimal: Do we need a digitally stored record memory?*
        - Overall Vote - 84% voted "no" (n = 32)
        - Clinicians - 91% voted "no" (n = 23)
        - Excluding involvement with product development - 84% voted "no" (n = 30)

- **Battery (previously titled 'Battery Power')**
    - Discussion on the Minimal characteristic for Battery Power (retitled to 'Battery') focused on the difference between a spot check and continuous monitoring device. For a continuous monitoring device, participants mentioned that the battery life should ideally last longer and that the device should be able to be used when plugged in and charging. The WHO tabletop specification requires more than 6 hours according to the WHO-UNICEF Technical Specifications and Guidance for Oxygen Therapy Devices [6, p. 130]. Lack of consensus in voting was likely due to the fact that for a spot-check Pulse Oximeter, >12 hours on a single charge would be preferred. However, for a continuous Pulse Oximeter, >6 hours on a single charge, consistent with the WHO-UNICEF Technical Specifications and Guidance for Oxygen Therapy Devices [6, p. 130] would suffice. Following the Consensus Meeting, one participant commented that "Battery duration of more than one hour will be very difficult (costly). You will need to specify the conditions for testing this requirement. Most battery performance deteriorate over time. Battery indicator is critical."

- There was agreement in the room that all characteristics relating to Utility Requirements (e.g., Back-up Battery; Battery Power; Batteries; Voltage; Power Requirement; Maximum Power Consumption; Response During Power Outage; Surge Protection, Electrical Plug) be reviewed and harmonized following the TPP meeting.
- *Minimal: Should the battery power last >6hr or >12hrs?*
  - Overall Vote - 59% voted ">6hr" (n = 32)
  - Clinicians - 62% voted ">6hr" (n = 21)
  - Excluding involvement with product development - 60% voted ">6hr" (n = 30)
- *Optimal: Rechargeable battery, >24hr on single charge*
- *Minimal: Rechargeable battery, >6hr on single charge*

- **Instrument Pricing**
  - There was disagreement on the Minimal characteristic for ex-works price of the device (inclusive of warranty and two probes for neonatal use). Some participants noted that the ex-works price was misleading given that there are several mark-ups added and that the landed cost may be easier for buyers to understand. Product developers noted that $100 ex-works is not feasible for a continuous measurement device (i.e., not a "finger pulse ox").
  - *Minimal: <$250 ex-works*
    - Overall Vote - 85% Agree (n = 20)
    - Clinicians - 92% Agree (n = 13)
    - Excluding involvement with product development - 85% Agree (n = 20)
- **Consumable Pricing**
  - There was disagreement on the Minimal characteristic for consumable pricing which, for the basis of the discussion, was assumed to be two neonatal probes per year. Technical developers discussed that the probes were an expensive component and that the current cost per probe is $20-$40 per probe ex-works with an average lifespan of 6 months.
  - *Minimal: <$80 per year ex-works (two probes)*
    - Overall Vote - 86% Agree (n = 14)
    - Clinicians - 88% Agree (n = 8)
    - Excluding involvement with product development - 86% Agree (n = 14)
- **Voltage**
  - As noted in the WHO-UNICEF Technical Specifications and Guidance for Oxygen Therapy Devices [6, p. 68], "In the case of oxygen therapy products, poor power conditions can significantly harm electrically powered oxygen concentrators, as well as pulse oximeters that require power directly from a mains source, or require recharging from a mains source". There was disagreement on the Minimal characteristic and whether a separate TPP was needed for a voltage stabilizer, although it was noted the WHO-UNICEF Technical Specifications and Guidance for Oxygen Therapy Devices [6, p. 133] does provide technical specifications for voltage stabilizers specific to those paired with oxygen therapy products. Agencies noted the importance of considering global ranges in development. From a technical perspective, a message to clinicians was to ensure that facilities install "grounding" (e.g., use of a metal rod). One proposal was to clear safety guidelines for medical device voltage per country.

- There was agreement in the room that all characteristics relating to Utility Requirements (e.g., Back-up Battery; Battery Power; Batteries; Voltage; Power Requirement; Maximum Power Consumption; Response During Power Outage; Surge Protection, Electrical Plug) be reviewed and harmonized following the TPP meeting.
- *Optimal: None*
- *Minimal: Model must match the voltage and frequency of the purchasing country's local power grid (e.g., 110-120 VAC at 60 Hz or 220-240 VAC at 50 Hz)*

The following characteristics were not discussed at the TPP Consensus Meeting explicitly, however, additional comments were received and incorporated into the discussion:

- **SPO2 Range**
  - With regard to the SP02 range, Pre-Meeting survey comments highlighted that "Saturation at 0% is not clinically meaningful", "there is no method available for calibrating pulse oximeters below 70%", and that "[readings are] never accurate or clinically useful below 70%". One participant responded that while oxygen therapy ideally would have started before the patient reaches these levels, there may be value and "clinical utility to ensure that the patient IS resaturating".
- **Decontamination**
  - Pre-Meeting survey comments highlighted the need to clarify appropriate disinfection agents. Comments received from an international NGO provided further clarification noting that each country has their own decontamination protocol since the WHO only provides guidance rather than explicit protocol. The guidance provided specifies super-basic mild soap solution, not submerging the device, and wipe-able in the case of contact with bodily fluid, and ability to use scheduled disinfectant [50]. While the process of decontaminating would likely be carried out by an IPC specialist, it is important for the manufacturer to control their Ingress Protection (IP) rating.
- The following Product Specific ISO Standards were highlighted in the Pre-Meeting survey responses:
  - ISO 80601-2-61(current 2017) specific to pulse oximetry, title: Medical electrical equipment -- Part 2-61: Particular requirements for basic safety and essential performance of pulse oximeter equipment, and provides guidance on accuracy claims and validation. and ISO13485
- Additional considerations received from participants are as follows:
  - "We should specify the conditions / context for accuracy testing. In newborns the within subject (breath by breath) variation in SpO2 within a single minute when the SpO2 is below 95% is > 3% RMSD. ISO only requires testing in adults. Currently ISO accuracy is < 4% RMSD. Neonates at low SpO2 will be at least this for a "minimal" requirement."
  - "Motion, perfusion, skin color and external light interference are key issues that have not been addressed."
  - "Devices need to be cleanable, waterproof (to a degree- IPX rating), drop and vibration tolerant."

DELPHI-LIKE SURVEY: PULSE OXIMETER

Table 22: Delphi-like survey results for Pulse Oximeter TPP prior to Consensus Meeting (data as of Oct 25, 2019)

	Optimal		Minimal		
**Characteristic**	**Optimal requirement**	**% agreement (n size)**	**Minimal requirement**	**% agreement (n size)**	**Collated comments from Delphi-like survey**
**Intended Use**	Optimal: To continuously monitor oxygen saturation (Sp02) and pulse rate (PR) for neonatal patients.	**91%**<br>**n = 44**	Minimal: Same as Optimal.	**86%**<br>**n = 42**	9 comments as summarized below<br>• Theme: Spot Checking vs. Continuous Monitoring<br>• Spot checking SpO2 is appropriate and adequate for assessment and monitoring of most newborns requiring oxygen therapy. A recent trial in Nigeria by Hamish Graham et al demonstrated that intermittent monitoring was also effective<br>• Closer monitoring, which may or may not involve continuous monitoring, is important for preterm neonates on oxygen (and some other very sick or deteriorating neonates)
**Target Operator**	Optimal: For use in low- and middle-income countries by a wide variety of clinicians, including nurses, clinical officers, and pediatricians.	**93%**<br>**n = 43**	Minimal: Same as Optimal	**98%**<br>**n = 42**	6 comments as summarized below<br>• Theme: Broaden Users<br>• Add 'nurse assistants' and 'community health workers'<br>• Non licensed providers make up a significant proportion of the healthcare workforce. Pulse oximetry monitoring is simple to learn so it does not exclusively require licensed providers if they are not available (i.e. in lower levels of the healthcare system)<br>• Optimal would be if a lay person could use it
**Target Population**	Optimal: Neonates (<28 days)	**80%**<br>**n = 44**	Minimal: Same as Optimal.	**80%**<br>**n = 41**	12 comments as summarized below<br>• Theme: Broaden age range or specify weight range<br>• Typically manufacturers specify a weight range not an age range

	Optimal		Minimal		
					• Pulse oximetry is useful in small hospitals and clinics where newborn care might be a small part of their workload, and any oximeter should be used also for older children<br>• Optimal/minimal would be <28days but also compatible for infants 1-6 kg<br>• Make upper weight higher if aiming to care for older sick infants (upper limit then probably 8-10 kg)
**Target Setting**	Optimal: Hospitals in low-resource settings	**77%**<br>**n = 44**	Minimal: Same as Optimal.	**74%**<br>**n = 43**	19 comments as summarize below<br>• Theme: Broaden vs. Narrow Target Setting<br>o Lower levels of the health system if oxygen available and resources adequate<br>o Other units of the hospital<br>o Potentially higher income counties<br>o Personnel in some primary hospitals (versus secondary and tertiary hospitals) are not well trained on how to use pulse oximeters<br>o In every birthing unit<br>o Community settings<br>• Minimal: hospital in resource-limited settings, Optimal: health centres (primary)
**International Standard**	Optimal: ISO 13485:2016 Medical devices – Quality management systems -- Requirements for regulatory purposes.	**81%**<br>**n = 32**	Minimal: Same as Optimal.	**77%**<br>**n = 30**	11 comments as summarized below<br>• Theme: Add to Additional International Standards vs. Irrelevance<br>• Consider inclusion of ISO 80601-2-61(current 2017) specific to pulse oximetry, title: Medical electrical equipment -- Part 2-61: Particular requirements for basic safety and essential performance of pulse oximeter equipment, and provides guidance on accuracy claims and validation.<br>• Alternatively, some respondents commented that having ISO13485 does not necessarily lead to good performance

	Optimal		Minimal		
**Regulation**	Optimal: CE marking or US FDA Clearance	**75%**<br>**n = 36**	Minimal: Same as Optimal.	**71%**<br>**n = 34**	12 comments as summarized below<br>• Theme: Add more flexibility v. irrelevance of characteristic<br>• Consider additional 'or' options:<br>o Other Stringent Regulatory Authorities – Japan or Australia or Canada<br>o Consider regulatory bodies of Low- and Middle-Income Countries<br>• Some respondents did not think that regulatory approval necessarily translated to good performance
**Pulse Rate**	Optimal: 25-250 bpm	**77%**<br>**n = 39**	Minimal: 60-200 bpm	**51%**<br>**n = 37**	23 comments as summarized below<br>• Theme: Wide Variety of Suggested Ranges<br>o WHO / UNICEF Interagency Specification is 30-240 bpm<br>o One respondent said, meaningful HR ranges for infants are:<br>▪ <60 (when compressions start)<br>▪ <100 (when ventilation support starts)<br>▪ >180 (tachycardia definition)<br>▪ >220 (concern for cardiac tachyarrythmias).<br>o Other respondents also suggested the following ranges:<br>▪ Optimal range: 40-230 bpm | minimal range: 50-200 bpm<br>▪ 25-240 bpm<br>▪ 25-120 bpm<br>▪ 25-200 bpm<br>▪ > 250 bpm, perhaps 300 bpm<br>▪ 25-250 bpm<br>▪ 30-240 bpm

	Optimal		Minimal		
					▪ 30-250 bpm<br>• Not a technical challenge
**Pulse Rate Accuracy**	Optimal: +-3 bpm	**88%**<br>**n = 41**	Minimal: Same as Optimal.	**82%**<br>**n = 38**	10 comments as summarized below<br>• Theme: Additional Suggested Ranges<br>o WHO / UNICEF Interagency Specification is +- 3 bpm<br>o +- 3 bpm at 90% is much different than at 50%<br>o +- 3 bpm should be over 10 second average<br>o +- 15%<br>o +- 2 bpm (to align with devices already on market)<br>o +- 5 bpm would be sufficient<br>o Consideration should be made for movement and low perfusion<br>o Consideration for saturation levels and average time
**Pulse Rate Resolution**<br>*(corrected from 'Pressure')*	Optimal: 1 bpm	**94%**<br>**n = 36**	Minimal: Same as Optimal.	**94%**<br>**n = 33**	3 comments as summarized below<br>• WHO / UNICEF Interagency Specification is +- 3 bpm
**Sp02 Accuracy**	Optimal: +-2%	**91%**<br>**n = 43**	Minimal: +-3%	**80%**<br>**n = 41**	12 comments as summarized below<br>• Theme: Accuracy Data at Various Perfusion / Movement Conditions<br>o UNICEF SD/WHO specs will be +/- 3% for neonates, and most devices that make claims will not go beyond this because you cannot carry-out a lab desaturation (breathdown) on a neonate to validate otherwise.

	Optimal		Minimal		
					"SpO2 accuracy (in the range at least 70-100%): within ± 2% under ideal conditions of use, and within ± 3% for all patients and perfusion/movement conditions." - For both minimal and Optimal (whatever the accuracy threshold is chosen to be for each), at least the detection range and motion/no-motion should be specified in order to compare apples to apples - Require as 'Optimal' that proof of accuracy data be available, as we have found that many are unable to provide supporting data showing compliance to ISO
**Sp02 Range**	Optimal: 0-100%	**81%**<br>**n = 42**	Minimal: 70-100%	**75%**<br>**n = 40**	18 comments as summarize below - Theme: Additional Suggested Ranges - Saturation at 0% is not clinically meaningful - There is no method available for calibrating pulse oximeters below 70% - Never accurate or clinically useful below 70% - Some cardiac conditions the SpO2 is showing lower values (in the 60ies), therefore I would prefer a range of 50 - 100%
**Alarms**	Optimal: Visual and Auditory	**98%**<br>**n = 43**	Minimal: Visual	**60%**<br>**n = 42**	21 comments as summarized below - Theme: Auditory is More Useful than Visual - Auditory might be less expensive - Consider waveform and auditory pulse tone - Lower tone as the heart rate or SPO2 lowers - Theme: Add detail on when alarms are triggered

	Optimal		Minimal		
					o ISO 80601-2-61 re. alarms: a cause for alarm when probe site must be changed (necessary on neonatal skin)<br>o WHO-UNICEF spec requires audible and visual alarms for:<br>▪ low/high saturation<br>▪ low/high pulse rate<br>▪ sensor error or disconnect<br>▪ system error<br>▪ low battery<br>o Audible and visual alarms for low/high saturation and pulse rate, threshold set by user<br>o Alarm override and temporary silencing function
**Consumables**	Optimal: >12 months before required	**88%**<br>**n = 40**	Minimal: >6 months before required	**64%**<br>**n = 39**	19 comments as summarized below<br>• Theme: Clarify what is meant by consumable<br>o Probes are accessories?<br>o Consider disposable single-use sensors as consumable<br>• Theme: Ideally consumable should last more than 6 months<br>o Deliver 12 months of stock<br>o Improve wiring at connection points without increasing costs<br>o Ideally, there would be no consumables
**Alarm Limits - PR**	Optimal: Adjustable	**95%**<br>**n = 40**	Minimal: 80-160 bpm	**70%**<br>**n = 37**	15 comments as summarized below<br>• Theme: Wide Variety of Suggested Ranges<br>o Ranges<br>▪ 50 – 200 bpm - less than 60bpm starts compressions; 200bpm would be minimal in my mind. Knowing >220 is helpful but is also

	Optimal		Minimal		
					▪ a rare case scenario (for tachyarrhythmias)<br>▪ 160 bpm is too low for an upper limit - suggest using 180 / 200 bpm as upper limit to avoid frequent alarming in the "borderline" babies with HR 160 - 180 bpm which may be due to crying or restlessness instead of illness<br>▪ 50-120 bpm<br>▪ 80-180 bpm<br>▪ I would also want the device to get an alarm at 60 bpm in any resuscitation situation<br>o Non-Adjustable - Adjustability of the alarms increase risk of user error and/or use on a different patient population<br>o Partially adjustable - should be closed settings not fully adjustable. For example 1) neonate setting 2) infant setting 3) pediatric setting, etc.<br>▪ "In a district hospital, I would want the alarms to be locked; in a tertiary I prefer the alarms to be adjustable."
**Alarm Limits - Sp02**	Optimal: Adjustable	**92%<br>n = 39**	Minimal: 88-99%	**59%<br>n = 39**	18 comments as summarized below<br>• Theme: Wide Variety of Suggested Ranges<br>o Ranges:<br>▪ <88%<br>▪ 75%<br>▪ 80%<br>▪ 85%<br>▪ 90-95% (respondent cited as WHO recommendation)

	Optimal		Minimal		
					o Adjustable:<br>▪ Make this a minimal requirement too<br>▪ Adjustment is important because you want to set the alarm according to the environment; e.g., the altitude might impact the levels you want and we have highlands in Nigeria where normal values of oximetry may be lower<br>▪ MUST ALWAYS be adjustable or at least able to turn off<br>▪ It is not helpful if the alarm is sounding permanently on a sick child<br>o Non-Adjustable - Adjustability of the alarms increase risk of user error and/or use on a different patient population<br>o Partially adjustable - should be closed settings not fully adjustable. For example 1) neonate setting 2) infant setting 3) pediatric setting, etc.
**Continuous Measurement**	Optimal: Yes	**95%**<br>**n = 41**	Minimal: Same as Optimal	**84%**<br>**n = 38**	9 comments as summarized below<br>• Theme: Spot Checking vs. Continuous Monitoring<br>• Spot checking SpO2 is appropriate and adequate for assessment and monitoring of most newborns requiring oxygen therapy<br>• Closer monitoring, which may or may not involve continuous monitoring, is important for preterm neonates on oxygen (and some other very sick or deteriorating neonates)

	Optimal		Minimal		
**Decontamination**	Optimal: Easy to clean with common disinfecting agents	**98%** **n = 43**	Minimal: Same as Optimal	**98%** **n = 40**	3 comments as summarized below<br>• Theme: Need clarity on which disinfecting agents are appropriate
**Patient Interface**	Optimal: Neonate specific, biocompatible and reusable.	**90%** **n = 41**	Minimal: Same as Optimal	**87%** **n = 38**	9 comments as summarized below<br>• Theme: Broaden range to include sensors / probes for other patient populations<br>o Older infants<br>o Children<br>o Mothers
**Size**	Optimal: Small footprint, left at bedside with dock.	**74%** **n = 42**	Minimal: Handheld with dock.	**78%** **n = 40**	14 comments as summarized below<br>• Theme: Size and/or Configuration may need to consider additional insights<br>o Comments on Handheld<br>▪ May be cheaper<br>▪ More easily displaced<br>▪ May not allow for continuous monitoring<br>▪ More easily used across patients without cleaning<br>▪ Shorter connection cables<br>▪ Shorter battery life<br>o Comments on Docking<br>▪ May prevent loss<br>▪ May limit use at bedside<br>▪ Need to ensure recharge is possible at bedside while also being used<br>o Comments on other configurations<br>▪ Rolling, portable pulse oximeters reduce loss and allow for continuous and spot-checking

	Optimal		Minimal		
**Training Required**	Optimal: Minimal	**84%**<br>**n = 43**	Minimal: Minimal	**80%**<br>**n = 41**	9 comments as summarized below<br>• Theme: 'Minimal' is too subjective; need something more specific<br>• Users need to be trained on the significance of monitoring<br>• Most of training is not on the device but the application of the sensor and the interpretation of information<br>• More specificity required, both with respect to minimum user qualifications and time - e.g., "A health care worker with at minimum a nursing degree can be trained in a 2-day workshop" or "A community health worker can be trained in a 1-week course", etc...<br>• Ideally should not require training or training built into device or easily accessible via phone
**User Manual**	Optimal: User manual and additional training materials (checklists, videos, guides) in English and local language. Attached to device with labels and markings where possible.	**85%**<br>**n = 41**	Minimal: User manual provided.	**85%**<br>**n = 40**	15 comments as summarized below<br>• Focus on limits of the pulse oximeter<br>• One manual per ward versus one per device<br>• Manual should be easily found online<br>• Not necessarily the responsibility of the manufacturer<br>• All claims must be filed with the regulatory dossier, so this is not as straight forward as a simple translation. Appropriate, professional translations are a must and are costly to the manufacturer. Additionally, local language varies greatly across a country and is often-times not even the official language of the country and so this may not be a reasonable ask of manufacturers

	Optimal		Minimal		
**Usage Meter**	Optimal: Digitally stored record displaying cumulative hours of operation.	76%<br>n = 37	Minimal: Digitally stored record displaying 50 previous readings or >50 hours.	72%<br>n = 36	17 comments as summarized below<br>• Theme: Clarify what is meant by usage meter<br>o To determine if device is used<br>o To determine if device needs to be serviced<br>o Historical record of data is helpful for continuous monitoring while record of readings is useful for spot-checking<br>o Change 'meter' to 'storage' or 'memory'<br>o Useful for research purposes for 72 hours of readings<br>o Useful for overnight readings for 12-24 hours at higher level facilities but probably out of scope for most neonatal units.<br>o Could add a lot to cost
**Voltage**	Optimal: 110-240V 50-60hz	83%<br>n = 36	Minimal: 220-240V 50-60hz	66%<br>n = 35	16 comments as summarized below<br>• Applicable to the battery charger and charging station<br>• The requirements for power input voltage/frequency and plug type of the equipment must be chosen according to the local electrical supply. Source: https://www.220-electronics.com/media/images/world-voltage-map.gif<br>• Voltage can always be corrected with step-up / step-down transformers; however, these come at an added cost. So whether the cost be borne by the purchaser (Caribbean, Central- or South-American countries w/ 120V) or the manufacturer who makes devices that can work across all contexts<br>• Frequency needs to be appropriate for frequency rating of specific country, as this is something that cannot be corrected and though 50 Hz can be used in a 60 Hz system, it is hard on the device and it will be compromised

	Optimal		Minimal		
					Voltage stabilizers and surge suppressors are important to consider
**Battery Powered**	Optimal: >24hr on single charge	**93%**<br>**n = 40**	Minimal: None	**36%**<br>**n = 36**	23 comments as summarized below<br>• Theme: Clarify what is meant by 'None':<br>o Backup power is a must have<br>o Optimal: rechargeable batteries with ability to swap out to standardly available batteries (e.g. AA)<br>o Minimal: rechargeable batteries<br>o Can device be used while charging?<br>• Theme: Wide variation in length of battery backup<br>o 30 minutes<br>o 1 hour<br>o 8 hours<br>o 12 hours (cited as UNICEF-WHO specification)<br>o 24 hours
**Weight**	Optimal: <500 grams, portable	**83%**<br>**n = 40**	Minimal: Same as Optimal	**82%**<br>**n = 39**	10 comments as summarized below<br>• Theme: Varying opinions on the need to specify weight<br>o Portable may be better for Minimal<br>o "Clinicians would rather work with a 2kg device that works well than a 200g device that doesn't"<br>o Less portable is viewed as more robust<br>o Portability may lead to disappearance of device<br>o WHO-UNICEF interagency spec is less than 400g for a handheld device (no weight maximum for tabletop device)

	Optimal		Minimal		
**Warranty**	Optimal: 5 years	**80%**<br>**n = 40**	Minimal: 1 year	**82%**<br>**n = 38**	13 comments as summarized below<br>• Theme: 5 years may be unrealistic<br>• UNICEF-WHO spec is 2 years recommended, at least 1 year mandatory<br>• Optimal should by 2 years<br>• To honor a 5 year warranty, you will have to have strong in-country representation<br>• "Any manufacturer that I have ever spoken to was more than willing to extend a warranty (to 2, maybe 3), but at a cost"<br>• "What might be more useful is that during any procurement, consideration be given to establishing a SLA with an in-country rep. In this case, you can "swap out" in the event of a break-down, and there is no discussion of warranties"
**Instrument Pricing**	Optimal: <$150 ex-works	**80%**<br>**n = 35**	Minimal: <$250 ex-works	**65%**<br>**n = 34**	12 comments as summarized below<br>• Theme: Extremely price-sensitive geography and even $250 was viewed as too expensive by some respondents<br>• Optimal price was viewed as potentially overly ambitious for bedside rather than handheld type<br>• This device needs to be better than devices sold in high-income countries so may be tough to hit target price<br>• Cheaper options available<br>• Would need to understand quality of the device before paying this much<br>• I think you could safely set "Optimal" to <$100, and "Minimal" to <$175 for ex-works, including 1 probe (min) and 1 year warranty on unit

	Optimal		Minimal		
**Consumable Pricing**	Optimal: <$50 / year ex-works	**79%**<br>**n = 33**	Minimal: <$100 per year ex-works	**47%**<br>**n = 34**	16 comments as summarized below<br>• Theme: Extremely price-sensitive geography and $100 was viewed as too expensive by some respondents<br>o "Generic probes cost much less than that, and last more than a year"<br>o Too costly if above $50 / year<br>• Theme: Provide more specificity for quantity and type of consumable<br>o Differentiate between a consumable (disposable probe) and spare (reusable probe). I am assuming that this question is about reusable probes.<br>o I think you could safely set "Optimal" to <$40, and "Minimal" to <$80 for ex-works, probes have 6 mo. warranty for 2 disposable probe and 2 reusable probe

Figure 22: Summary of organizational affiliation for Pulse Oximeter TPP from Delphi-like Survey prior to Consensus Meeting (data as of Oct 25, 2019)

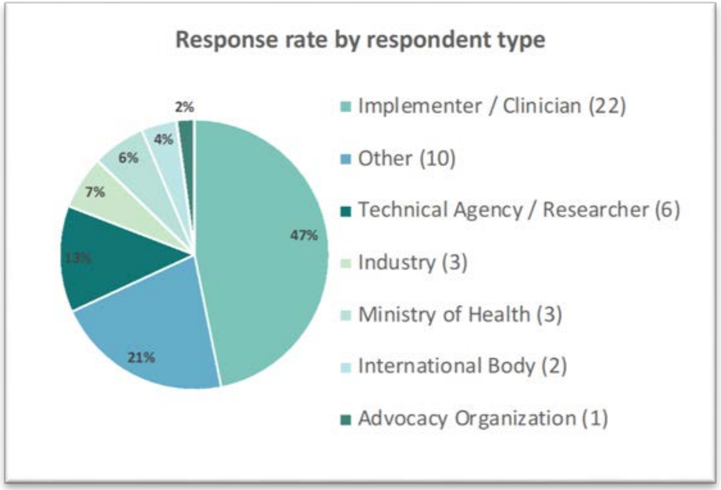

Respondent type	Percentage
Implementer / Clinician (22)	47%
Other (10)	21%
Technical Agency / Researcher (6)	13%
Industry (3)	6%
Ministry of Health (3)	6%
International Body (2)	4%
Advocacy Organization (1)	2%

Figure 23: Summary of response rate by country for Pulse Oximeter TPP from Delphi-like Survey prior to Consensus Meeting (data as of Oct 25, 2019)

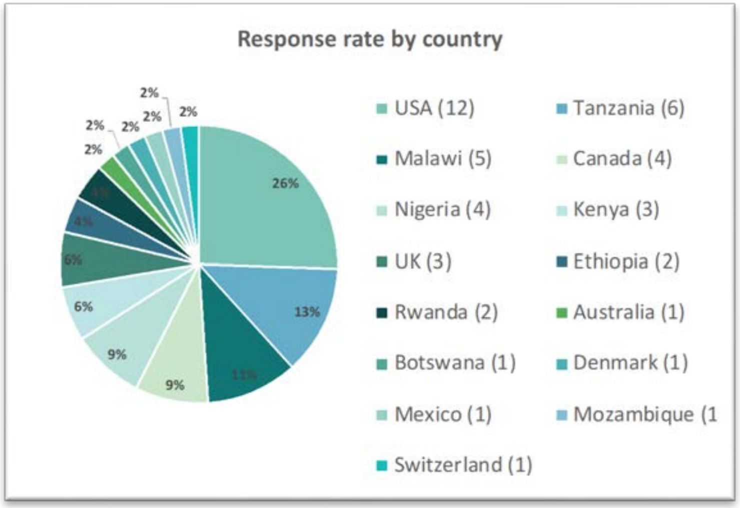

Country	Percentage
USA (12)	26%
Tanzania (6)	13%
Malawi (5)	11%
Canada (4)	9%
Nigeria (4)	9%
Kenya (3)	6%
UK (3)	6%
Ethiopia (2)	4%
Rwanda (2)	4%
Australia (1)	2%
Botswana (1)	2%
Denmark (1)	2%
Mexico (1)	2%
Mozambique (1)	2%
Switzerland (1)	2%

INTRODUCTION: RESPIRATORY RATE / APNEA MONITOR

Respiratory rate is a critical vital sign. The causes are many but are commonly due to respiratory pathology. Increased respiratory rate (> 60bpm) in newborns can indicate respiratory distress syndrome (RDS), but as with infants and children, a high respiratory rate can also indicate pneumonia, which is the primary infectious cause of childhood death worldwide.
A low respiratory rate or gaps in breathing in infants is likewise indicative of potentially severe health concerns. Apnea of prematurity is a condition in which newborns temporarily stop breathing. Many apneas resolve without intervention, but frequent apnea (often paired with bradycardia and low SpO2) can indicate an underlying condition such as sepsis, hypoglycemia, or anemia. Apnea of prematurity (AOP), a condition in which newborns temporarily stop breathing due to neurologic immaturity, affects nearly 50% of infants born earlier than 32 weeks gestational age and nearly 100% of those born at fewer than 28 weeks, and may last for several weeks [51]. AOP can be associated with dangerous decreases in heart rate and oxygenation, which, left unchecked, could lead to respiratory arrest, increased morbidity, or death.

In high-resource settings, respiratory rate is monitored using impedance pneumography, which requires expensive patient monitors and delicate electronic sensors. Alternatively in high-resource settings, AOP is monitored by using low nursing ratios (1:2) in conjunction with continuous heart rate and pulse oximetry monitoring. In this setting, a nurse or caregiver would provide a manual intervention in the event of an AOP event causing a low heart rate or oxygen saturation, in order to re-establish normal breathing. In low-resource settings, a nurse, normally faced with high nurse to patient ratios, must rely on limited continuous monitoring capability of heart rate and saturation with most infants only receiving intermittent manual monitoring. Additionally, they should observe the number of breaths a child takes in one minute, a procedure that is both time-consuming and inadequate for monitoring infants at risk of AOP.

FINAL TPP: RESPIRATORY RATE / APNEA MONITOR

Table 23: Final TPP for Respiratory Rate/Apnea Monitor

Final target product profile for Respiratory Rate Monitor / Apnea Monitor		
**Characteristic**	**Optimal**	**Minimal**
SCOPE		

**Intended Use**	To provide continuous monitoring of respiratory rate	
**Target Operator**	For use in low- and middle-income countries by a wide variety of clinicians, including nurses, clinical officers, and pediatricians	
**Target Population**	Neonates (born at any gestational age and require ongoing care)	
**Target Setting**	Hospitals in low-resource settings, but, may be used in health facilities based on country guidelines	Hospitals in low-resource settings
SAFETY AND STANDARDS		
**Quality Management** [1]	ISO 13485:2016 Medical devices – Quality management systems -- Requirements for regulatory purposes	
**Regulation**	At least one of: CE marking, approved by US FDA or another stringent regulatory body of a founding member of IMDRF (e.g., Japan or Australia or Canada)	
TECHNICAL CHARACTERISTICS		
**Apnea Detection**	Detect periods of central apnea exceeding 20s duration (at 0)	
**Respiratory Rate Accuracy**	± 2 bpm	± 5 bpm
**Respiratory Rate Range**	0-100 bpm	
**Alarm**	Visual and auditory	An alarm (visual or auditory)
**Patient Interface**	Interface is biocompatible and reusable	Interface is biocompatible
**Respiratory Rate Alarm Limits**	Automatically adjust based on patient age	30-60 bpm
**Apnea Intervention**	Yes	No
PURCHASING CONSIDERATIONS		
**Instrument Pricing**	<$100 ex-works	<$250 ex-works
UTILITY REQUIREMENTS		
**Power Source**	Mains with rechargeable battery	Mains with rechargeable battery
**Battery**	Rechargeable battery, >24hrs on a single charge	Rechargeable battery, >6hrs on a single charge

**Voltage**	Model must match the voltage and frequency of the purchasing country's local power grid (e.g., 110-120 VAC at 60 Hz or 220-240 VAC at 50 Hz)	
TRAINING AND MAINTENANCE		
**User Instructions**	User manual and additional training materials (checklists, videos, guides) in at least one national official language for the country of intended use. Attached to device with labels and markings where possible	User manual provided in at least one national official language
**Warranty**	5 years	1 year
**Decontamination**	Easy to clean with common disinfecting agents	

[1] *There was not 75% voting agreement on the Minimal characteristic. Please refer to the TPP Report discussion for additional detail.*

CONSENSUS MEETING SUMMARY: RESPIRATORY RATE / APNEA MONITOR

To arrive at the final TPP for Respiratory Rate/Apnea Monitor (Table 23), we conducted a pre-meeting survey to prioritize the items for discussion at the Consensus Meeting for characteristics that achieved below 75% agreement in the survey results (Table 24). An overview of the discussion at the Consensus Meeting of these characteristics is included below.

- **Apnea Detection**
    - Consensus was achieved in the room (without a Mentimeter vote) for both the Optimal and Minimal characteristics. Clinicians confirmed that they definitely wanted the monitor to alarm for apnea and that, additionally, it would be helpful to have the ability to adjust the interval detection frequency based on the baby. Product developers noted that this technology was not fully mature yet and challenging to improve. They explained that from a technical perspective, the rate was retrospective and therefore more complex to technically calculate the average over a historical period of time and produce a read out based on the determined algorithm. One clinician suggested that the algorithm be built so that when a period of apnea was detected, a side countdown begins and if it hits 20 seconds, an alarm would sound. Both clinicians and technical developers agreed on the importance of two separate counters: one for historical averages of respiratory rate and a second for when a baby experiences apnea, upon which a prompt warning alarm would sound. One international NGO participant mentioned an interest in better understanding 'normal' apnea patterns/trends in newborns prior to agreeing on alarm levels since desaturation could happen quite quickly.
    - *Optimal: Detect periods of central apnea exceeding 20s duration (at 0).*
    - *Minimal: Same as Optimal.*
- **Respiratory Rate Accuracy**

- Consensus was achieved in the room (without a Mentimeter vote) for the Minimal characteristic. Product developers noted that it can be challenging to conduct validation on accuracy for ±2 bpm since a gold standard does not currently exist to measure respiratory rate accuracy. A research question was developed emphasizing the need for an improved way to measure accuracy since international standards for respiratory rate accuracy do not currently exist. There is therefore a need to define gold standard for respiratory rate accuracy and standardize experimental conditions. Ethical considerations are important in evaluating and validating these standards at upper and lower ranges on neonates. One participant recommended that both SpO2 and respiratory rate accuracy thresholds be based on real clinical data (typical variability). In the Pre-Meeting report survey, one individual commented that given there was not a 'gold standard' measurement for respiratory rate, they specified a reasonable reference standard with human experts and video recordings and specifying an acceptable degree of agreement with that standard, using the 95% Limits of Agreement and the Bland-Altman plot. However, an international NGO responded that using humans as a 'reasonable reference standard' can be troublesome since they can often be inconsistent or incorrect. Furthermore, they noted that "regulators will likely not see [human experts] as a means to validate".
    - *Minimal: ±5bpm*
- **Respiratory Rate Range**
    - Consensus was achieved in the room (without a Mentimeter vote) for the Optimal characteristic to be 0-100 bpm. Clinicians confirmed that 100 bpm was sufficient at the higher end and would not impact their treatment decision. Rather, they confirmed that it is helpful to view the trend (i.e., if a baby is at 85bpm and moving up to 95bpm).
    - *Optimal: 0-100 bpm*
    - *Minimal: Same as Optimal*
- **Respiratory Rate Resolution**
    - This characteristic was not discussed as it was determined to remove from the TPP. It was noted that the characteristic was too specific for early stage development.
- **Alarm**
    - Consensus was achieved in the room (without a Mentimeter vote) that an alarm should exist for the Minimal requirement, however, flexibility could be left to the developer on the type of alarm. Some participants voiced a preference for a sound alarm while others noted that in a hospital environment where there are already a lot of sound alarms, it was important to have a visual alarm.
    - *Minimal: Yes (an alarm)*
- **Apnea Alarm Limits**
    - This characteristic was not discussed as it was determined to remove from the TPP. It was noted that the characteristic was too specific for early stage development.
- **Consumables**
    - This characteristic was not discussed as it was determined to remove from the TPP. It was noted that the characteristic was too specific for early stage development.
- **Voltage**

- There was agreement in the room that all characteristics relating to Utility Requirements (e.g., Back-up Battery; Battery Power; Batteries; Voltage; Power Requirement; Maximum Power Consumption; Response During Power Outage; Surge Protection, Electrical Plug) be reviewed and harmonized following the TPP meeting.
- *Optimal and Minimal: Model must match the voltage and frequency of the purchasing country's local power grid (e.g., 110-120 VAC at 60 Hz or 220-240 VAC at 50 Hz)*

- **Battery (previously titled 'Battery Powered')**
  - Clinicians noted that the intention is to leave the device on for 24 hours, hence the time period. Discussion in the room encouraged product developers to be creative (e.g., device could plug into wall, connect with other devices, etc.). Clinicians noted a preference to avoid wired connections to mains and emphasized that "there are already too many wires". There was agreement in the room that if the device was not connected to a mains power source, constant power for 24 hours would be required, however, if it was connected to a mains power source, then 12 hours back-up for power shedding would be sufficient for the Optimal characteristic. For the Minimal characteristic, if the device was not connected to a mains power source, constant power for 24 hours would be required, however, if the device was connected to a mains power source, then at least 6 hours of back-up for power shedding should be required. Product developers noted that the battery was more complex than a "watch battery" since certification was required for each part and supplier used in development.
  - A research question was established to review existing literature on power cuts to determine how long power supply should last. One meeting participant subsequently sent the following recommendations providing data on power cuts to share with the broader group in this report: 1) Limited electricity access in health facilities of sub-Saharan Africa: a systematic review of data on electricity access, sources, and reliability [66] 2) Oxygen insecurity and mortality in resource-constrained healthcare facilities in rural Kenya [67] and 3) Assessment of Power Availability and Development of a Low-Cost Battery-Powered Medical Oxygen Delivery System: For Use in Low-Resource Health Facilities in Developing Countries [68].
  - There was agreement in the room that all characteristics relating to Utility Requirements (e.g., Back-up Battery; Battery Power; Batteries; Voltage; Power Requirement; Maximum Power Consumption; Response During Power Outage; Surge Protection, Electrical Plug) be reviewed and harmonized following the TPP meeting.
  - *Optimal: Rechargeable battery, >24hrs on a single charge*
  - *Minimal: Rechargeable battery, >6hrs on a single charge*
- **Size**
  - This characteristic was not discussed as it was determined to remove from the TPP. It was noted that the characteristic was too specific for early stage development.
- **Weight**
  - This characteristic was not discussed as it was determined to remove from the TPP. It was noted that the characteristic was too specific for early stage development.
- **Consumable Pricing**
  - This characteristic was not discussed as it was determined to remove from the TPP. It was noted that the characteristic was too specific for early stage development.

Table 24: Delphi-like survey results for Respiratory Rate Monitor TPP prior to Consensus Meeting (data as of Oct 25, 2019)

	Optimal		Minimal		
**Characteristic**	**Optimal requirement**	**% agreement (n size)**	**Minimal requirement**	**% agreement (n size)**	**Collated comments from Delphi-like survey**
**Intended Use**	Optimal: To provide continuous monitoring of respiratory rate.	**79%**<br>**n = 14**	Minimal: Same as Optimal.	**77%**<br>**n = 13**	4 comments as summarized below<br>• Theme: Continuous not needed in all situations<br>• Theme: Clinical value<br>o "Respiratory rate monitors my experience are finicky, alarm a lot, and are only useful if there is someone there that was confident to respond to them. Theoretically you could try to get mothers to do this (respond to an alarm) if the ward is set up for them to stay with the babies (not usually the case). But I think even if the moms CAN be w/the babies 24/7 that is unrealistic expectation of them (we have trouble getting moms in the US to do this)."<br>o "Optimally: In my mind the only useful respiratory rate monitor is one that could alarm AND respond (stimulate the baby) in the event of an apnea. Otherwise, this is something I would consider more for a ICU/level 3 care technology versus comprehensive/level 2 care technology."<br>o "Not accurate and of very limited immediate need in a SCN or NICU in limited resource not enough staffing…just use sat"

	Optimal		Minimal		
**Target Operator**	Optimal: For use in low- and middle-income countries by a wide variety of clinicians, including nurses, clinical officers, and pediatricians.	100%<br>n = 12	Minimal: Same as Optimal	100%<br>n = 11	1 comment<br>• "I agree that this is the population that should be able to apply and trouble shoot a respiratory monitor - but it's not realistic in my opinion that the nurse:patient ratio will be such that they can respond to all the alarms."
**Target Population**	Optimal: Neonates (<28 days)	83%<br>n = 12	Minimal: Same as Optimal.	73%<br>n = 11	6 comments as summarized below<br>• Theme: Broaden age range or specify weight range
**Target Setting**	Optimal: Hospitals in low-resource settings	67%<br>n = 12	Minimal: Same as Optimal.	64%<br>n = 11	8 comments as summarized below<br>• Theme: Optimal would include high-functioning health centres (primary) or home-use<br>o "Could be useful in diagnoses of pneumonia (would impact Intended Use)"<br>o "How far into the periphery of the health service we can push oxygen for neonates? On the one hand the mortality tends to be at the village level or first-contact health facility, so we should aim for the smallest health facilities that care for in-patients. On the other hand, the level of skill, training and other resources needed to care for neonates may make it impractical to go beyond the largest sub-district health centres. Whatever level we choose, it is worthwhile thinking about some technology to help stabilise and transport a neonate who needs referral to a more central level."

	Optimal		Minimal		
**International Standard**	Optimal: ISO 13485:2016 Medical devices – Quality management systems -- Requirements for regulatory purposes.	**78%**<br>**n = 9**	Minimal: Same as Optimal.	**63%**<br>**n = 8**	4 comments as summarized below<br>• The standard does not define specific testing requirements for respiratory monitors. Something similar to standard of pulse oximetry would be desirable<br>• Requiring ISO may limit innovation and is not based on what is needed for low-resource settings
**Regulation**	Optimal: CE marking or US FDA Clearance	**73%**<br>**n = 11**	Minimal: Same as Optimal.	**60%**<br>**n = 10**	5 comments as summarized below<br>• Theme: Add more flexibility v. irrelevance of characteristic<br>• Consider additional 'or' options:<br>o Other Stringent Regulatory Authorities – Japan or Australia or Canada<br>o Consider regulatory bodies of Low- and Middle-Income Countries<br>Some respondents did not think that regulatory approval necessarily translated to good performance.
**Apnea Detection**	Optimal: Detect periods of central apnea exceeding 20s duration.	**70%**<br>**n = 10**	Minimal: None.	**60%**<br>**n = 10**	8 comments as summarized below<br>• Theme: Recommend removing "central" to make implicit this is for premature infants<br>• Theme: An accurate count of respiratory rate may alone be useful<br>• Consider shorter periods
**Respiratory Rate Accuracy**	Optimal: +- 2 bpm	**75%**<br>**n = 12**	Minimal: +- 10 bpm	**30%**<br>**n = 10**	9 comments as summarized below<br>• Theme: Wide variation in what is required vs. what might be technically achievable<br>o Minimal needs to be less than +- 5 bpm<br>o Optimal needs to be +- 5 bpm<br>o Impossible to achieve<br>o 10 bpm is not clinically useful / would alarm too often?

	Optimal		Minimal		
					o WHO has indicated absolute breathing rate deviance ±2 breaths/min in measuring RR. I believe what is stated here as Optimal is actually also minimal.<br>o There is not a 'gold standard' measurement of respiratory rate that allows the calculation of accuracy for a new method. On the other hand, we did manage to specify a reasonable reference standard (the best being human experts with video recordings), and we can specify an acceptable degree of agreement with that standard, using the 95% Limits of Agreement and the Bland-Altman plot
**Respiratory Rate Range**<br>*(corrected from 'Pressure')*	Optimal: 0-120 bpm	73%<br>n = 11	Minimal: 0-100 bpm	78%<br>n = 9	4 comments as summarized below<br>• Theme: Other suggested ranges were provided<br>o May be able to lower Minimal window to 0-90 bpm<br>o Change Optimal to 0-100 bpm<br>o Limit of 80 bpm is fine<br>o For a neonate, anything above 60 is a cause for concern, and PALS indicate that even in HEALTHY premies and neonates, breath rate can climb to 70 and 55 respectively. So long as there is clinical rational for such a high end on the range, then one can only ask! However, given other 'asks' in this questionnaire, I am only aware of products whose algorithms can manage an upper bound of 90
**Respiratory Rate Resolution**	Optimal: 1 bpm	100%<br>n = 11	Minimal: 2 bpm	67%<br>n = 9	5 comments as summarized below<br>• Need clarity on accuracy rate versus respiratory rate resolution

	Optimal		Minimal		
					• No technical reason to do this<br>• Minimal should be same as Optimal
**Alarm**	Optimal: Visual and auditory	100%<br>n = 13	Minimal: Visual only	67%<br>n = 12	7 comments as summarized below<br>• Theme: Auditory Only preferred over Visual Only<br>• Depends on Continuous Monitoring vs. Spot Check<br>• Minimal should be same as Optimal
**Apnea Alarm Limits**	Optimal: Adjustable	82%<br>n = 11	Minimal: None	70%<br>n = 10	6 comments as summarized below<br>• "If the system has a built in apnea alert for pauses > 20 seconds, then there shouldn't be room to adjust it, possibly to silence the alarm but not to change the limits"<br>• "What does it mean to have an "adjustable" apnea alarm? Like it only alarms if it's associated with a decrease in heart rate as well? Or do you mean that you can adjust the length of the apnea period for which it alarms? That also wouldn't really make sense to me as it seems like this would be a parameter internally set to optimize sensitivity/specificity of alarms"<br>• "What about alarms for battery, error, etc."
**Consumables**	Optimal: >12 months before required	82%<br>n = 11	Minimal: >6 months before required	60%<br>n = 10	4 comments as summarized below<br>• Theme: Need clarity on what consumables are required; prefer reusable probes or sensors
**Decontamination**	Optimal: Easy to clean with common disinfecting agents	100%<br>n = 12	Minimal: Same as Optimal.	100%<br>n = 11	2 comments as summarized below<br>• Provide guidance<br>• Needs to withstand chlorine and bleach

	Optimal		Minimal		
**User Manual**	Optimal: User manual and additional training materials (checklists, videos, guides) in English and local language. Attached to device with labels and markings where possible.	**83%**<br>**n = 12**	Minimal: User manual provided.	**82%**<br>**n = 11**	5 comments as summarized below<br>• Electronic copy is highly preferred<br>• All claims must be filed with the regulatory dossier, so this is not as straight forward as a simple translation. Appropriate, professional translations are a must and are costly to the manufacturer. Additionally, local language varies greatly across a country and is often-times not even the official language of the country (take India, for example) and so this is simply not a reasonable ask of manufacturers. "User language preference prioritized, English is mandatory." Also, any manufacturer should be encouraged to use pictograms to support user manuals
**Voltage**	Optimal: 110-240V 50-60hz	**100%**<br>**n = 10**	Minimal: 220-240V 50-60hz	**44%**<br>**n = 9**	5 comments as summarized below<br>• Theme: Lower Voltage should be considered<br>o 12 Volt might be more appropriate for this size of device<br>o This is a device with a very low power consumption so, like our laptops and our mobile phones, the Optimal should be the minimal
**Battery Powered**	Optimal: Yes, > 4 hr on a single charge	**85%**<br>**n = 13**	Minimal: No	**42%**<br>**n = 12**	9 comments as summarized below<br>• Theme: Clarify what is meant by 'None':<br>o Backup power is a must have<br>o Optimal: rechargeable batteries with ability to swap out to standardly available batteries (e.g. AA)<br>o Minimal: rechargeable batteries<br>o Can device be used while charging?<br>• Theme: Variation in length of battery backup<br>o 1 hour

	Optimal		Minimal		
					o 4 hours
**Patient Interface**	Optimal: Interface is biocompatible and reusable.	100%<br>n = 12	Minimal: Interface is biocompatible.	80%<br>n = 10	4 comments as summarized below<br>• Theme: Even low-cost consumables become a financial burden, and single-use items should be avoided wherever possible
**Respiratory Rate Alarm Limits**	Optimal: Automatically adjust based on patient age	82%<br>n = 11	Minimal: 30-60 bpm	100%<br>n = 9	6 comments as summarized below<br>• Theme: Broaden the range:<br>o Minimal needs to be 0-60 since the whole point is to detect apnea in neonates?<br>o Consider some other method besides age (e.g., weight)<br>o Not really clinically useful
**Size**	Optimal: Small footprint; can be left at bedside.	75%<br>n = 12	Minimal: Same as Optimal.	73%<br>n = 11	4 comments as summarized below<br>• Theme: Small size may need to consider additional insights<br>o More easily displaced<br>o More easily used across patients without cleaning
**Weight**	Optimal: < 500 g	73%<br>n = 11	Minimal: Same as Optimal.	78%<br>n = 9	4 comments as summarized below<br>• Theme: Varying opinions on the need to specify weight<br>o Weight on baby?<br>o Less portable is viewed as more robust<br>o Portability may lead to disappearance of device<br>WHO-UNICEF interagency spec is less than 400g for a handheld device (no weight maximum for tabletop device)

	Optimal		Minimal		
**Apnea Intervention**	Optimal: Yes	**88%**<br>**n = 8**	Minimal: No	**75%**<br>**n = 8**	6 comments as summarized below<br>• Theme: Varying opinions on Apnea Intervention<br>• Comment on Minimal: Apnea monitor without automated intervention is likely to be background noise in busy setting<br>• No clinical evidence these interventions work<br>• This is important for neonates. If a device that monitors RR has an algorithm sensitive enough to generate RR but can also discern what is apnea and not simply loss of signal, that would be great!
**Warranty**	Optimal: 5 years	**80%**<br>**n = 10**	Minimal: 1 year	**90%**<br>**n = 10**	5 comments as summarized below<br>• Theme: 5 years too long<br>• Suggested Ranges:<br>o 2 years<br>• To honor a 5 year warranty, you will have to have strong in-country representation. All an extended warranty is a degree of assurance of the above, and this will come at a cost. Manufactures of concentrators willing to extend a warranty from 2-5 do so at a cost. What might be more useful is that during any procurement, consideration be given to establishing a SLA with an in-country rep. In this case, you can take care of any major PPM requirements, as well as "swap out" in the event of a break-down, and there is no discussion of warranties and no need for spares and an in-country source for consumables.
**Instrument Pricing**	Optimal: <$100 ex-works	**90%**<br>**n = 10**	Minimal: <$250 ex-works	**78%**<br>**n = 9**	2 comments as summarized below<br>• Based on COGS, minimal should be <$150, but I am assuming RR derivation using a limited technologies (based on other questions in this survey)

	Optimal		Minimal		
**Consumable Pricing**	Optimal: <$50 per year ex-works	**80%**<br>**n = 10**	Minimal: <$100 per year ex-works	**67%**<br>**n = 9**	4 comments as summarized below<br>• Single-use items not feasible<br>• Minimal, under $80, Optimal, under $40.

Figure 24: Summary of organizational affiliation for Respiratory Rate Monitor TPP from Delphi-like Survey prior to Consensus Meeting (data as of Oct 25, 2019)

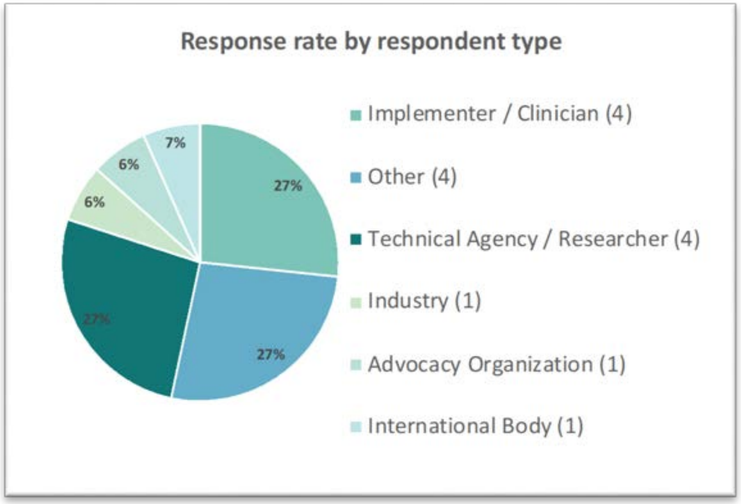

Respondent type	Percentage
Implementer / Clinician (4)	27%
Other (4)	27%
Technical Agency / Researcher (4)	27%
Industry (1)	6%
Advocacy Organization (1)	6%
International Body (1)	7%

Figure 25: Summary of response rate by country for Respiratory Rate Monitor TPP from Delphi-like Survey prior to Consensus Meeting (data as of Oct 25, 2019)

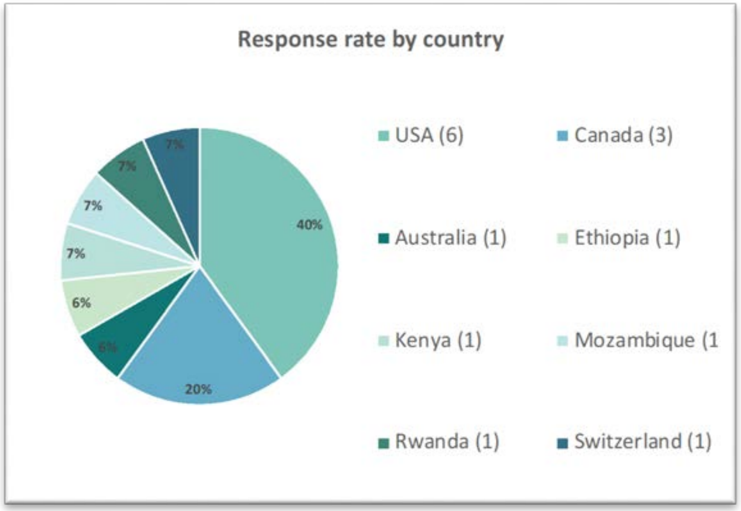

Country	Percentage
USA (6)	40%
Canada (3)	20%
Australia (1)	7%
Ethiopia (1)	7%
Kenya (1)	7%
Mozambique (1)	7%
Rwanda (1)	7%
Switzerland (1)	7%

INTRODUCTION: SUCTION PUMP

Clinicians periodically need to clear an infant's airway through the use of a suction pump. Safe ranges for neonatal suctioning depending on the size of the infant and are generally considered to be between 60-100mmHg.

FINAL TPP: SUCTION PUMP

Table 25: Final TPP for Suction Pump

Final target product profile for Suction Pump		
**Characteristic**	**Optimal**	**Minimal**
SCOPE		
**Intended Use**	Aspiration and removal of secretions, bodily fluids and foreign objects from a patient's airway or respiratory support system in the nasal, pharyngeal and tracheal areas	
**Target Operator**	For use in low- and middle-income countries by a wide variety of clinicians, including nurses, clinical officers, and pediatricians	
**Target Population**	Neonates (born at any gestational age and require ongoing care)	
**Target Setting**	Hospitals in low-resource settings, but, may be used in health facilities based on country guidelines	Hospitals in low-resource settings
SAFETY AND STANDARDS		
**Quality Management** [1]	ISO 13485:2016 Medical devices – Quality management systems -- Requirements for regulatory purposes	
**Regulation**	At least one of: CE marking, approved by US FDA or another stringent regulatory body of a founding member of IMDRF (e.g., Japan or Australia or Canada)	
TECHNICAL CHARACTERISTICS		
**Pressure**	60-120 mm Hg with continuous adjustment	

**Bottle Capacity**	1 L	
**Noise Level**	As low as possible	
**Cleaning**	Collection vessel easy to clean reusable	
**Maintenance**	No maintenance or lubrication	
**Operation Mode**	Adjustable to neonatal setting (60-100 mm Hg)	
PURCHASING CONSIDERATIONS		
**Instrument Pricing**	<$100 ex-works	<$250 ex-works
UTILITY REQUIREMENTS		
**Power Source**	Mains Power	Mains Power
**Voltage**	Model must match the voltage and frequency of the purchasing country's local power grid (e.g., 110-120 VAC at 60 Hz or 220-240 VAC at 50 Hz)	
TRAINING AND MAINTENANCE		
**User Instructions**	User manual and additional training materials (checklists, videos, guides) in at least one national official language for the country of intended use. Attached to device with labels and markings where possible	User manual provided in at least one national official language
**Warranty**	5 years	1 year

[1] *There was not 75% voting agreement on the Minimal characteristic. Please refer to the TPP Report discussion for additional detail.*

CONSENSUS MEETING SUMMARY: SUCTION PUMP

To arrive at the final TPP for Suction Pump (Table 25), we conducted a pre-meeting survey to prioritize the items for discussion at the Consensus Meeting for characteristics that achieved below 75% agreement in the survey results (Table 26). An overview of the discussion at the Consensus Meeting of these characteristics is included below.

- **Pressure**

- There was disagreement on both the Optimal and Minimal characteristic. Clinicians agreed that for the minimum end of the range, 60 mm Hg was acceptable. Product developers noted that there is not a significant incremental cost between the upper range between 100 mm Hg or 120 mm Hg. Consensus was achieved in the room (without a Mentimeter vote) that the Minimal should be the same as Optimal.
  - *Optimal: 60-120 mm Hg with continuous adjustment*
    - Overall Vote - 100% Agree (n = 18)
    - Clinicians - 100% Agree (n = 13)
    - Excluding involvement with product development - 100% Agree (n = 18)
  - *Minimal: 60-120 mm Hg with continuous adjustment (Same as Optimal)*
- **Noise Level**
  - Consensus was achieved that the sound level characteristic was referring to the operating noise level. Some product developers noted that from a technical standpoint, CE mark requires that this be under 50 decibels for operating noise, however, another participant confirmed that this was simply the minimum end of the range required and that "in operating rooms, the background noise can vary from 50 dBA to 85 dBA". Ultimately, consensus was achieved in the room (without a Mentimeter vote) for both the Optimal and Minimal characteristic to be the same and specify that the "lower the decibel level, the better". The spirit of the conversation emphasized that the noise levels should be as low as possible to protect the babies hearing.
  - *Optimal: As low as possible*
  - *Minimal: Same as Optimal*
- **Instrument Pricing**
  - Consensus was achieved in the room (without a Mentimeter vote) to reduce the Minimal price to <$250 ex-works.
  - *Minimal: <$250 ex-works*
- **Voltage**
  - There was agreement in the room that all characteristics relating to Utility Requirements (e.g., Back-up Battery; Battery Power; Batteries; Voltage; Power Requirement; Maximum Power Consumption; Response During Power Outage; Surge Protection, Electrical Plug) be reviewed and harmonized following the TPP meeting.
  - *Optimal and Minimal: Model must match the voltage and frequency of the purchasing country's local power grid (e.g., 110-120 VAC at 60 Hz or 220-240 VAC at 50 Hz)*

DELPHI-LIKE SURVEY: SUCTION PUMP

Table 26: Delphi-like survey results for Suction Pump TPP prior to Consensus Meeting (data as of Oct 25, 2019)

	Optimal		Minimal		
**Characteristic**	**Optimal requirement**	**% agreement (n size)**	**Minimal requirement**	**% agreement (n size)**	**Collated comments from Delphi-like survey**

	Optimal		Minimal		
**Intended Use**	Optimal: Aspiration and removal of secretions, bodily fluids and foreign objects from a patient's airway or respiratory support system in the nasal, pharyngeal and tracheal areas.	**92%**<br>**n = 12**	Minimal: Same as Optimal.	**91%**<br>**n = 11**	2 comments as summarized below<br>• Optimal: Ability to provide suction at different maximum settings between 80-120mmHg with variable attachments for suctioning that vary in possible depth (nasal, nasopharyngeal, nasopharyngeal-tracheal) as well as size (Children 12F I think but not sure upper size limit? I'd have to check that one. Infants are 10Fr, neonates are 6-8Fr)<br>• Minimal: (for neonates): Ability to provide suction at different maximum settings between 80-100mmHg with variable attachments for suctioning that vary in possible depth (nasal, nasopharyngeal) as well as size (infants I think are 10Fr, neonates are 6-8Fr)
**Target Operator**	Optimal: For use in low- and middle-income countries by a wide variety of clinicians, including nurses, clinical officers, and pediatricians.	**100%**<br>**n = 12**	Minimal: Same as Optimal	**100%**<br>**n = 10**	0 comments
**Target Population**	Optimal: Neonates (<28 days)	**91%**<br>**n = 11**	Minimal: Same as Optimal.	**80%**<br>**n = 10**	4 comments as summarized below<br>• Theme: Broaden Age Range<br>o Children<br>o Adults<br>• Theme: Small vs. Sick Newborns<br>o Small newborn (<2.5kg) parameters will be slightly different from sick newborn parameters. Small newborns need 6-8Fr catheters, 60-80mmHg. Sick (but not small) newborn parameters would be 60-100mmHg 8-10Fr catheters for nasopharyngeal suctioning

	Optimal		Minimal		
**Target Setting**	Optimal: Hospitals in low-resource settings	92%<br>n = 12	Minimal: Same as Optimal.	90%<br>n = 10	2 comments as summarized below<br>• Theme: Broaden Target Setting<br>• Optimal should be health centres (primary)
**International Standard**	Optimal: ISO 13485:2016 Medical devices – Quality management systems -- Requirements for regulatory purposes.	86%<br>n = 7	Minimal: Same as Optimal.	83%<br>n = 6	1 comments<br>• More specific standards for suction vs. blanket ISO 13485
**Regulation**	Optimal: CE marking or US FDA Clearance	86%<br>n = 7	Minimal: Same as Optimal.	86%<br>n = 7	1 comments<br>• CE Marking in the medical domain
**Pressure**	Optimal: 60-100 mm Hg with continuous adjustment	73%<br>n = 11	Minimal: 60-100 mm Hg	67%<br>n = 9	4 comments<br>• Optimal: 60-120 mm Hg<br>• Optimal: recommend adding - continuous adjustment within the full range<br>• Canadian policy states 80-100 mm Hg<br>• Need clinical input
**Bottle Capacity**	Optimal: 1 L	92%<br>n = 13	Minimal: Same as Optimal.	92%<br>n = 12	2 comments as summarized below<br>• For neonatal application only<br>• "I think this depends on how it gets cleaned. I'm not sure that 1L capacity is really necessary? The most you're ever going to suction from a kid is a few mL ... I'd guess 50mL generously. So I'd says 50 x # of patients you can suction without cleaning anything is the capacity?"
**Noise Level**	Optimal: <65 dB	57%<br>n = 7	Minimal: 65 dB	50%<br>n = 6	4 comments as summarized below<br>• Recommendation to change Optimal: <=60 and Minimal: <50

	Optimal		Minimal		
**Cleaning**	Optimal: Collection vessel easy to clean reusable.	**100%**<br>**n = 13**	Minimal: Same as Optimal.	**100%**<br>**n = 12**	2 comments as summarized below<br>• Collection and patient interface easy to clean and reusable
**Maintenance**	Optimal: No maintenance or lubrication.	**85%**<br>**n = 13**	Minimal: Same as Optimal.	**82%**<br>**n = 11**	4 comments as summarized below<br>• Clarity on what is meant by lubrication – nasal saline prior to suctioning?<br>• Maintenance should be required but minimal and/or easy
**Operation Mode**	Optimal: Adjustable to neonatal setting (60-100 mm Hg)	**77%**<br>**n = 13**	Minimal: Same as Optimal.	**90%**<br>**n = 10**	4 comments as summarized below<br>• Optimal: 60-120 mm Hg<br>• Optimal: recommend adding - continuous adjustment within the full range<br>• Need clarity as to why this is linked to the pressure only. I would maybe think of battery or mains operation mode or electrical or manual operation mode, or adult, pediatric or neonatal operation mode
**User Manual**	Optimal: User manual and additional training materials (checklists, videos, guides) in English and local language. Attached to device with labels and markings where possible.	**83%**<br>**n = 12**	Minimal: User manual provided.	**82%**<br>**n = 11**	2 comments as summarized below<br>• Manuals of limited use<br>• English and/or French would be sufficient
**Voltage**	Optimal: 110-240V 50-60hz	**83%**<br>**n = 12**	Minimal: 220-240V 50-60hz	**70%**<br>**n = 10**	3 comments as summarized below<br>• Most LMICs use high voltage power<br>• Is there some built-in surge protection? 220V power fluctuates from 200-250 depending per country. Not many data available. It's worth doing some data collection in the countries you work<br>• Kenya single phase voltage is 240V

	Optimal		Minimal		
					• 110-240v, 50-60 is good for different rating for different countries
**Warranty**	Optimal: 5 years	**83%**<br>**n = 12**	Minimal: 1 year	**64%**<br>**n = 11**	3 comments as summarized below<br>• Need to be tender for more than 1yr warranty and service. Maybe you pay for it separately<br>• 1 year is good<br>• Warranties are not useful
**Instrument Pricing**	Optimal: <$100 ex-works	**91%**<br>**n = 11**	Minimal: <$300 ex-works	**70%**<br>**n = 10**	4 comments as summarized below<br>• Theme: Discrepancy on whether this is reasonable or not<br>• We just purchased some suction pumps (manual & electric) for within these ranges<br>• Should be cheaper<br>• It's difficult to produce a good quality pump for that price. What about the warranty and training cost, does that come on top of this? I guess so.<br>• What about consumables?

Figure 26: Summary of organizational affiliation for Suction Pump TPP from Delphi-like Survey prior to Consensus Meeting (data as of Oct 25, 2019)

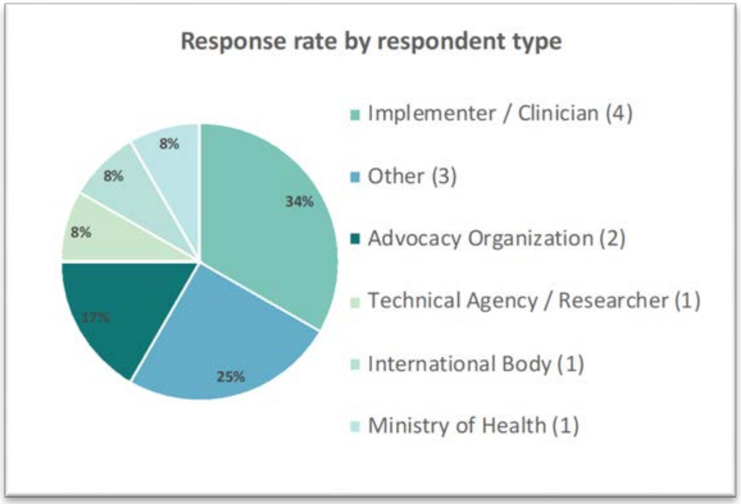

Respondent type	Percentage
Implementer / Clinician (4)	33%
Other (3)	25%
Advocacy Organization (2)	17%
Technical Agency / Researcher (1)	8%
International Body (1)	8%
Ministry of Health (1)	8%

Figure 27: Summary of response rate by country for Suction Pump TPP from Delphi-like Survey prior to Consensus Meeting (data as of Oct 25, 2019)

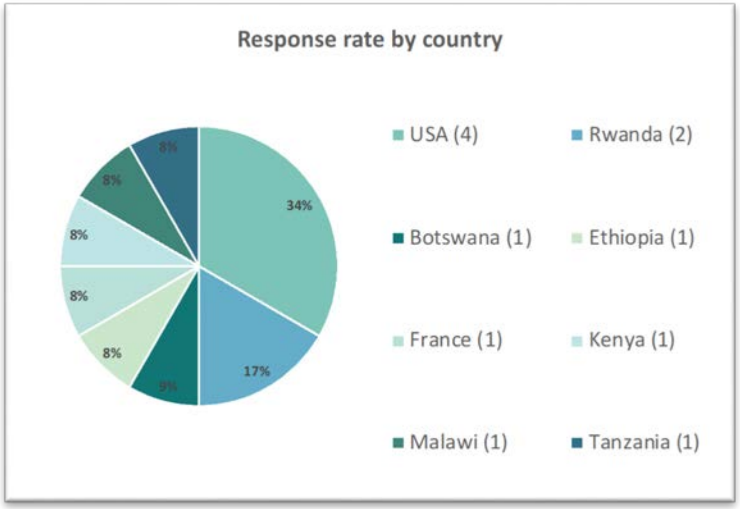

Country	Percentage
USA (4)	34%
Rwanda (2)	17%
Botswana (1)	9%
Ethiopia (1)	8%
France (1)	8%
Kenya (1)	8%
Malawi (1)	8%
Tanzania (1)	8%

THERMAL MANAGEMENT

In general, newborns require a warmer environment than adults and the smaller the newborn, the higher the temperature needs to be. A newborn's ability to stay warm can be easily compromised by the temperature of its surroundings since newborn infants regulate body temperature much less efficiently than adults and lose heat more easily. Low birth weight and premature babies often face even greater risk [74].

As many as 85% of infants born in hospitals in low-resource settings become cold (defined as <36.5°C) [52]. Mortality rates increase with each degree Celsius of temperature lost. While the risks of being too cold are well recognized, hypothermia remains a largely invisible problem in overcrowded newborn units in low-resource settings. Hypothermia in newborns requires rapid diagnosis, which is often difficult in crowded and understaffed wards. Hypothermia not only increases the chances of acidosis, sepsis and RDS, but may indicate the presence of system illness such as infection or hypoglycemia.

Hypothermia can be treated using Kangaroo Mother Care (KMC), blankets/hats, warming cribs, warming mattresses, and radiant warmers. While hypothermia can be treated using KMC, infants and their caregivers may not be eligible for reasons such as, but not limited to: mother is recovering from surgery or the infant is in need of intensive care.

Attempts to warm a cold baby without monitoring temperatures carefully can result in hyperthermia. Rapid swings in temperature – known as thermal shock – can lead to negative outcomes, including death. Additionally, unrecognized fever in infants may lead to delays in treating neonatal sepsis and resulting in increased morbidity.

In high-resource settings, these negative outcomes are prevented by using incubators which continuously monitor and adjust temperature, or, with intermittent monitoring (every 3-4 hours) for infants who are in open cribs. However, incubators cost thousands of dollars and often require delicate sensors and expensive consumables. Existing temperature monitoring devices that are affordable in lower resource settings do not have the features necessary for the accurate detection of hypothermia or are not designed for a clinical setting.

In addition to the risks of hypothermia, pre-term infants and children are at high risk of infection, which can cause hyperthermia. A diagnosis of fever is not conclusive for any of these conditions, but it is a critical early sign of potentially severe illness. In combination with a respiratory rate monitor and pulse oximeter, continuous temperature monitoring can provide guidance to

clinicians on what type of treatment to pursue; once treatment has begun, it can indicate whether treatment is working or needs to be increased.

INTRODUCTION: RADIANT WARMER

Hypothermia can be prevented using radiant warmers that carefully control heat based on manual settings or the infant's own temperature. Radiant warmers provide heat using an overhead heating source and are preferred for infants who may require greater access or closer short-term monitoring. Radiant warmers are preferred, in the short term, to warming cribs/incubators for infants who are unstable and may require significant intervention (such as resuscitation or invasive procedures).

FINAL TPP: RADIANT WARMER

Table 27: Final TPP for Radiant Warmer

Final target product profile for Radiant Warmer		
**Characteristic**	**Optimal**	**Minimal**
SCOPE		
**Intended Use**	Treatment and prevention of hypothermia in neonates requiring intensive thermal care	
**Target Operator**	For use in low- and middle-income countries by a wide variety of clinicians, including nurses, clinical officers, and pediatricians	
**Target Population**	Neonates (born at any gestational age and require ongoing care)	
**Target Setting**	Hospitals in low-resource settings	
SAFETY AND STANDARDS		
**Quality Management** [1]	ISO 13485:2016 Medical devices – Quality management systems -- Requirements for regulatory purposes	
**Regulation**	At least one of: CE marking, approved by US FDA or another stringent regulatory body of a founding member of IMDRF (e.g., Japan or Australia or Canada)	
TECHNICAL CHARACTERISTICS		
**Benchtop Measurement Accuracy**	±0.1°C	

**Clinical Measurement Accuracy**	±0.3°C	
**Stability**	< 0.5°C	
**Includes Timer**	Yes	
**Includes Scale**	Yes	No
**Mobility**	Has wheels; can be moved by one person	
**Time to Indicate Accurate Temperature**	< 1 minute	< 90 seconds
**Uniformity**	< 1°C	
**Alarm Characteristics**	Visual and Auditory	
**Alarm Limits**	Adjustable	36.5°C-37.5°C
**Operating Temperature**	Harsh ambient condition, temperature 5-45 °C, humidity 15% to 95%, dusty air, elevation >=2000 meters	Harsh ambient temperature 10-40 °C, humidity 15%-95%, dusty air, elevation up to 2000 meters
**Patient Interface**	Interface is biocompatible and reusable	Interface is biocompatible
**Patient Accessibility and Visibility**	Patient is visible and accessible to healthcare worker	
**Temperature Control**	Based on infant's temperature and includes fail-safe mode	
PURCHASING CONSIDERATIONS		
**Consumables**	> 12 months before required	> 6 months before required
**Instrument Pricing**	<$500 ex-works	<$1,500 ex-works
**Consumable Pricing**	<$50 per year ex-works (includes two probes)	<$100 per year ex-works (includes two probes)
UTILITY REQUIREMENTS		
**Power Source**	Mains Power	Mains Power
**Power Consumption**	<250W maximum	<800W maximum

**Voltage**	Model must match the voltage and frequency of the purchasing country's local power grid (e.g., 110-120 VAC at 60 Hz or 220-240 VAC at 50 Hz)	
TRAINING AND MAINTENANCE		
**User Instructions**	User manual and additional training materials (checklists, videos, guides) in at least one national official language for the country of intended use. Attached to device with labels and markings where possible	User manual provided in at least one national official language
**Warranty**	5 years	1 year
**Decontamination**	Easy to clean with common disinfecting agents	

[1] *There was not 75% voting agreement on the Minimal characteristic. Please refer to the TPP Report discussion for additional detail.*

CONSENSUS MEETING SUMMARY: RADIANT WARMER

To arrive at the final TPP for Radiant Warmer (Table 27), we conducted a pre-meeting survey to prioritize the items for discussion at the Consensus Meeting for characteristics that achieved below 75% agreement in the survey results (Table 28). An overview of the discussion at the Consensus Meeting of these characteristics is included below.

- **Clinical Measurement Accuracy**
  - Consensus was achieved in the room (without a Mentimeter vote) to adjust the Optimal and Minimal characteristic to ±0.3°C. Product developers noted that ±0.3°C is required for ISO certification [81].
  - *Optimal:* ±0.3°C
  - *Minimal: Same as Optimal*
- **Includes Timer**
  - Consensus was achieved in the room (without a Mentimeter vote) for the Minimal characteristic to equal the Optimal characteristic. There was agreement in the room to remove the word APGAR from the characteristic and re-title to Includes Timer. The rationale was that when a baby arrives in the NICU they are beyond the APGAR stage. Product developers noted that there is no additional cost to add APGAR timer as it is simply "10-20 lines of code". One clinician mentioned that the challenge is that existing timers do not have an option to alarm at 2 minutes, but rather options for 1 minute, 5 minutes, and 10 minutes.
  - *Optimal: Yes*
  - *Minimal: Same as Optimal*

- **Time to Indicate Accurate Temperature**
    - Consensus was achieved in the room (without a Mentimeter vote) to table further discussion on this characteristic until further information is available as additional criteria is needed. Product developers noted that from a technical perspective, given heat transfer and surface temperature, it was challenging to read the temperature of the baby if the sensor was cold or not previously attached to the baby and that the timing would be "constrained by the laws of physics". Clinicians noted that ideally, they would like the temperature to be read in under 60 seconds. A research question to further explore the time required to indicate the accurate temperature of the baby and to measure the time in a standardized way was created.
- **Alarm Characteristics**
    - Consensus was achieved in the room (without a Mentimeter vote) to that the Minimal characteristic should equal the Optimal of Visual and Auditory alarms.
    - *Optimal: Visual and Auditory*
    - *Minimal: Visual*
- **Power Consumption**
    - There was agreement in the room that all characteristics relating to Utility Requirements (e.g., Back-up Battery; Battery Power; Batteries; Voltage; Power Requirement; Maximum Power Consumption; Response During Power Outage; Surge Protection, Electrical Plug) be reviewed and harmonized following the TPP meeting.
    - Consensus was achieved in the room (without a Mentimeter vote) for <800 Watts for the Minimal characteristic. Discussion in the room focused on the fact that the title of the characteristic could vary based on individual interpretation and therefore should be redefined based on the equipment definition.
    - *Optimal: <250W maximum*
    - *Minimal: <800W maximum*
- **Instrument Pricing**
    - Consensus was achieved in the room (without a Mentimeter vote) for the Optimal Instrument Pricing to be <$500 ex-works and the Minimal Instrument Pricing to be <$1,500 ex-works. Participants in the room commented that finding a device on the market below $1,000 is a challenge but innovators should strive for a lower price. Product developers noted that pricing can be reduced when the number of units purchased increases and economies of scale can be realized.
    - *Optimal: <$500 ex-works*
    - *Minimal: <$1,500 ex-works*
- **Consumable Pricing**
    - Consensus was achieved in the room (without a Mentimeter vote) for the Optimal Consumable Pricing to be <$50 per year ex-works and defined as including two probes and the Minimal Instrument Pricing to be <$100 per year ex-works (including two probes). Participants in the room clarified that each probe should last six months, hence two would be adequate for a one year supply. Product developers noted that probes are often damaged due to user misuse (e.g., forcing them in the wrong way) or overload and stressed the importance of "teaching people to treat medical device products like you would treat your iPhone as this is an essential tool".
    - *Optimal: <$50 per year ex-works (includes two probes)*

- *Minimal: <$100 per year ex-works (includes two probes)*

DELPHI-LIKE SURVEY: RADIANT WARMER

Table 28: Delphi-like survey results for Radiant Warmer TPP prior to Consensus Meeting (data as of Oct 25, 2019)

	Optimal		Minimal		
**Characteristic**	**Optimal requirement**	**% agreement (n size)**	**Minimal requirement**	**% agreement (n size)**	**Collated comments from Delphi-like survey**
**Intended Use**	Optimal: Treatment and prevention of hypothermia in neonates requiring intensive thermal care.	**88%**<br>**n = 17**	Minimal: Same as Optimal.	**94%**<br>**n = 16**	5 comments as summarized below<br>• Theme: A variety of proposed Intended Use language<br>o Place to keep infants warm while doing acute resuscitation (either directly following birth or when they come in septic)<br>o Optimal: Treatment and prevention of hypothermia in neonates requiring intensive thermal care when clinician access is needed<br>o Should also say "not eligible for KMC"<br>o Also for newborns requiring resuscitation immediately after birth (who may not necessarily require 'intensive thermal care' once stabilized)
**Target Operator**	Optimal: For use in low- and middle-income countries by a wide variety of clinicians, including nurses, clinical officers, and pediatricians.	**94%**<br>**n = 16**	Minimal: Same as Optimal	**93%**<br>**n = 15**	2 comments as summarized below<br>• Technology is required regardless of country income

	Optimal		Minimal		
**Target Population**	Optimal: Neonates (<28 days)	**93%**<br>**n = 15**	Minimal: Same as Optimal.	**100%**<br>**n = 14**	2 comments as summarized below<br>• There are some babies >28 days who may need to use a radiant warmer e.g. KMC babies who clinically deteriorate
**Target Setting**	Optimal: Hospitals in low-resource settings	**81%**<br>**n = 16**	Minimal: Same as Optimal.	**80%**<br>**n = 15**	5 comments as summarized below<br>• Technology is required in all hospitals with intensive or intermediate neonatal care regardless of country income<br>• Theme: Broaden range to include other levels of the health system<br>o Radiant warmers are also necessary at the health center level, and recommend to avail the equipment at that level as well<br>o I believe it should be available in all CEMONC facilities. In some countries, health centers (lower level that hospitals) can provide CEMONC services<br>o Some health centres have deliveries so radiant warmers should be accessible in these settings
**International Standard**	Optimal: ISO 13485:2016 Medical devices – Quality management systems -- Requirements for regulatory purposes.	**100%**<br>**n = 11**	Minimal: Same as Optimal.	**100%**<br>**n = 10**	0 comments
**Regulation**	Optimal: CE marking or US FDA Clearance	**82%**<br>**n = 11**	Minimal: Same as Optimal.	**90%**<br>**n = 10**	3 comments as summarized below<br>• CE is essential; FDA is not needed for low income countries, and it is very expensive to obtain

	Optimal		Minimal		
**Benchtop Measurement Accuracy**	Optimal: ±0.1°C	**92%**<br>**n = 13**	Minimal: Same as Optimal.	**100%**<br>**n = 12**	4 comments as summarized below<br>• Unclear what benchtop vs clinical accuracy means and how that would be measured/reported. Potentially combine?<br>• Clarify if servo or manual<br>• Theme: Overly stringent<br>o Update to ±0.3 °C (should be the same as warming crib, ±0.1°C is way too strict)<br>o Seems overly stringent
**Clinical Measurement Accuracy**	Optimal: ±0.2°C	**73%**<br>**n = 15**	Minimal: ±0.5°C	**79%**<br>**n = 14**	5 comments as summarized below<br>• Theme: Overly stringent vs. Not strict enough<br>o Optimal: ± 0.1°C | Minimal: Same as Optimal<br>o Optimal: ±0.5°C | Minimal: Same as Optimal<br>o Should match warming crib, way too strict
**Stability**	Optimal: < 0.5°C	**93%**<br>**n = 14**	Minimal: Same as Optimal.	**92%**<br>**n = 13**	2 comments<br>• ± 0.1°C
**Includes APGAR timer**	Optimal: Yes	**94%**<br>**n = 16**	Minimal: No	**73%**<br>**n = 15**	3 comments as summarized below<br>• Apgar is absolutely necessary during resuscitation<br>• Timing functionality is useful
**Includes Scale**	Optimal: Yes	**88%**<br>**n = 16**	Minimal: No	**80%**<br>**n = 15**	3 comments as described below<br>• Scale should only be included if it is very reliable, easily calibrated and robust overtime. Otherwise will just be needless complexity that introduces error with very little added efficiency.<br>• It is a "great to be" tool, but not absolutely necessary

	Optimal		Minimal		
**Mobility**	Optimal: Has wheels; can be moved by one person	88%<br>n = 17	Minimal: Same as Optimal.	88%<br>n = 16	3 comments as described below<br>• Has 4 wheels with locking castors. This is standard for radiant warmers and required. Use language that was in 02 concentrator TPP<br>• Can be fixed on a wall or on wheels
**Time to Indicate Accurate Temperature**	Optimal: < 90 seconds	71%<br>n = 17	Minimal: < 3 minutes	69%<br>n = 16	6 comments as described below<br>• A variety of alternative ranges were provided<br>o < 30 seconds<br>o < 90 seconds should be Optimal and minimal standard<br>o Time to Indicate Accurate Clinical Temperature:<br>▪ Optimal: < 3 minutes<br>▪ Minimal: < 5 minutes<br>o Too strict
**Uniformity**	Optimal: < 1°C	100%<br>n = 14	Minimal: Same as Optimal.	100%<br>n = 13	2 comments as summarized below<br>• "I'm not really sure over what surface area you're referring to it having this uniformity. Seems like more focused surface area, higher requirement of uniformity - if we're talking about the whole bed including the edges (where they baby shouldn't be anyway) then less stringent."
**Alarm Characteristics**	Optimal: Visual and Auditory	100%<br>n = 17	Minimal: Visual	56%<br>n = 16	9 comments as summarized below<br>• Theme: Minimal should include audio
**Alarm Limits**	Optimal: Adjustable	82%<br>n = 17	Minimal: 36.5°C-37.5°C	75%<br>n = 16	8 comments as summarize below<br>• Minimal: might increase range a bit depending on accuracy of the instrument<br>• For minimal, would suggest having slightly wider limits (e.g., 36-38°C)

	Optimal		Minimal		
					• Not realistic, would suggest updating to:<br>o Optimal: +/-0.5 C from baby set temp | Minimal: 36.5°C-37.5°C<br>o Alarm should be able to notify clinicians of hypothermia too<br>• Alarm limits should reflect normothermia<br>• Adjustable is not necessarily preferred. If adjustable pre-set options should be available to avoid user error<br>• The alarm limits should be adjustable as minimal specifications<br>• It is typical to not be able to independently adjust alarms, only to be able to adjust desired temperature of baby. Alarms adjust with desired baby temperature (when temp. is higher or lower than desired)"
**Consumables**	Optimal: > 12 months before required	**87%**<br>**n = 15**	Minimal: > 6 months before required	**79%**<br>**n = 14**	3 comments as summarized below<br>• Reference is not clear. A set of sufficient consumables should be included during technology incorporation to healthcare facility.
**Decontamination**	Optimal: Easy to clean with common disinfecting agents	**100%**<br>**n = 17**	Minimal: Same as Optimal.	**100%**<br>**n = 16**	0 comments
**Maximum Power Consumption**	Optimal: <250 Watts	**82%**<br>**n = 11**	Minimal: <800 Watts	**60%**<br>**n = 10**	4 comments as summarized below<br>• Raise minimal to <1000 Watts<br>• The power consumption could be higher than 800 Watts<br>• Ideally should be less

	Optimal		Minimal		
**Voltage**	Optimal: 110-240V 50-60hz	**86%**<br>**n = 14**	Minimal: 220-240V 50-60hz	**75%**<br>**n = 12**	4 comments as summarized below<br>• 220V is much more important than 110V in low resource countries<br>• 220V applies just to some countries. Minimal should be same as Optimal<br>• Different countries have different voltage rating
**Operating Temperature**	Optimal: Harsh ambient condition, temperature 5-45 °C, humidity 15% to 95%, dusty air, elevation >=2000 meters	**85%**<br>**n = 13**	Minimal: Harsh ambient temperature 10-40 °C, humidity 15%-95%, dusty air, elevation up to 2000 meters	**83%**<br>**n = 12**	3 comments as summarized below<br>• Suggest making less strict and more realistic<br>• Optimal: Harsh ambient condition, indoor temperature (20-40°C), humidity 30% to 80%, dusty air, elevation <=2000 meters<br>• Should work in any setting / environment<br>• Even it can be beyond this range
**Patient Interface**	Optimal: Interface is biocompatible and reusable	**93%**<br>**n = 15**	Minimal: Interface is biocompatible	**86%**<br>**n = 14**	3 comments as summarized below<br>• Optimal should be single patient use, to avoid cross-contamination. | Minimal should be reusable.<br>• Should be reusable
**Patient Accessibility and Visibility**	Optimal: Patient is visible and accessible to healthcare worker.	**88%**<br>**n = 17**	Minimal: Same as Optimal.	**94%**<br>**n = 16**	4 comments as summarized below<br>• Optimal: patient is visible, accessible but also secured (there are side rails that can be put up or down so they don't roll off) on the radiant warmer<br>• Disagree that this should be included in radiant warmer. A radiant warmer by default is open and accessible so this requirement seems unnecessary to include<br>• Need to define "accessibility and visibility' for developers

	Optimal		Minimal		
**Temperature Control**	Optimal: Based on infant's temperature and includes fail-safe mode	82%<br>n = 17	Minimal: Same as Optimal.	81%<br>n = 16	4 comments as summarized below<br>• "So incubators and radiant warmers ... getting people to use servo vs manual is super difficult. EVERYONE in my experience is relying on fail-safes or manual mode because we either don't have or are not sure that servo probes for the patient are actually working. Need to think hard about usability vs added functionality on this"<br>• It's recommended to incorporate the term "Servo-controlled"<br>• Also include as minimal and Optimal "a servo and manual mode"
**User Manual**	Optimal: User manual and additional training materials (checklists, videos, guides) in English and local language. Attached to device with labels and markings where possible.	100%<br>n = 17	Minimal: User manual provided.	94%<br>n = 16	2 comments as summarized below<br>• Warmers can be dangerous when not administered properly. Patient safety issue requires proper training
**Warranty**	Optimal: 5 years	76%<br>n = 17	Minimal: 1 year	94%<br>n = 16	5 comments as summarized below<br>• 5 years is too long, it is too hard for the companies to ensure that, but 1 year too short. 3 years is the actual expected best standard of warranty<br>• Warranty extensions usually impact on final pricing. Two years warranties are industry accepted<br>• One year is good<br>• No supplier will provide 5 years warranty<br>• Warmer functional issues come up frequently
**Instrument Pricing**	Optimal: <$500 ex-works	71%<br>n = 14	Minimal: <$1,000 ex-works	62%<br>n = 13	7 comments as summarized below<br>• I believe $1,000 should be the minimal requirement in order to have a quality product

	Optimal		Minimal		
					• Technology cost is above the $1,000 USD mark<br>• Raise to $1500 or $2000?<br>• Will still be expensive for many resource countries<br>• We are talking about low resource setting, and high prices for the equipment will not be feasible for this countries.<br>• Should ideally be as cheap as possible as facilities are likely to require numerous<br>• Depends on manufacturer model
**Consumable Pricing**	Optimal: <$50 per year ex-works	**79%**<br>**n = 14**	Minimal: <$100 per year ex-works	**62%**<br>**n = 13**	4 comments as summarized below<br>• It should be specified the consumable presentation: box/piece/set<br>• Should be as cheap as possible - temperature probes easily break and will be used heavily<br>• We are talking about low resource setting, and high prices for the equipment will not be feasible for this countries

Figure 28: Summary of organizational affiliation for Radiant Warmer TPP from Delphi-like Survey prior to Consensus Meeting (data as of Oct 25, 2019)

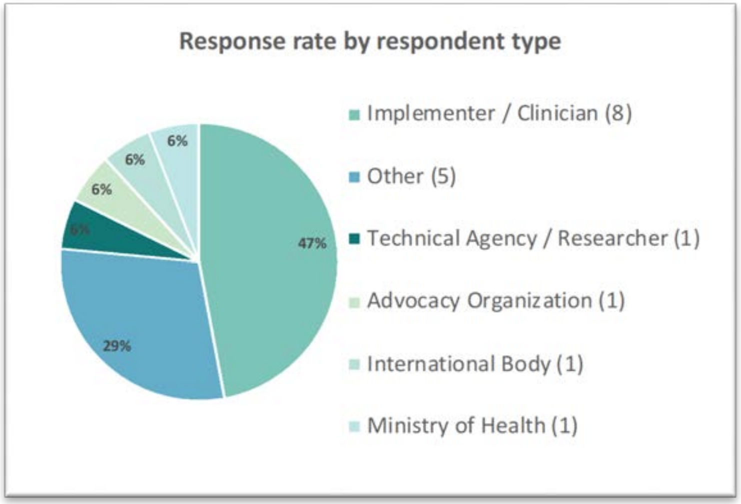

Respondent type	Percentage
Implementer / Clinician (8)	47%
Other (5)	29%
Technical Agency / Researcher (1)	6%
Advocacy Organization (1)	6%
International Body (1)	6%
Ministry of Health (1)	6%

Figure 29: Summary of response rate by country for Radiant Warmer TPP from Delphi-like Survey prior to Consensus Meeting (data as of Oct 25, 2019)

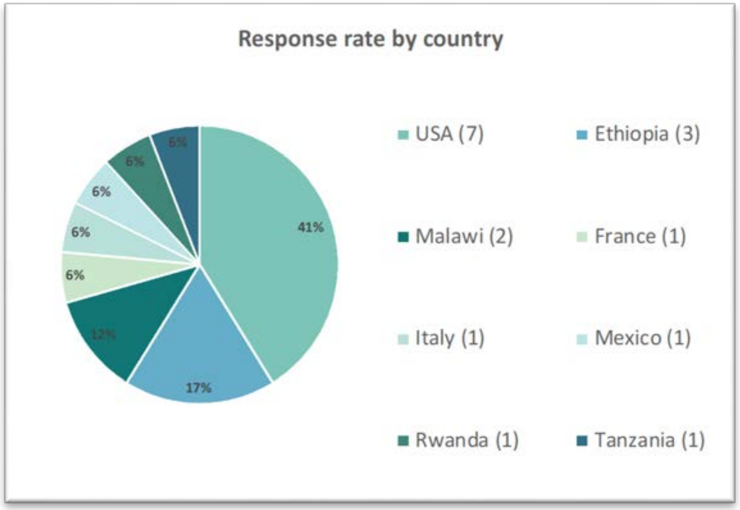

Country	Percentage
USA (7)	41%
Ethiopia (3)	18%
Malawi (2)	12%
France (1)	6%
Italy (1)	6%
Mexico (1)	6%
Rwanda (1)	6%
Tanzania (1)	6%

TEMPERATURE MONITOR (CONTINUOUS)

INTRODUCTION: TEMPERATURE MONITOR (CONTINUOUS)

Given that temperatures less than 36.5°C have been shown to be an independent risk factor for death in neonates [53], early recognition and treatment of hypothermia is critical. In overcrowded and understaffed hospital wards, where nursing to patient ratios are often in excess of 1:10 and most infants are not in incubators which continuously record temperature, obtaining temperature readings even 3-4 times per day can be challenging.

In high-resource settings, low nursing to patient ratios and availability of incubators, which continuously monitor temperatures, allows for close monitoring. In settings with high nurse to patient ratios, where incubators are limited, KMC is the preferential warming option. However, some infants require closer monitoring of temperature in open cribs and the ability to continuously monitor temperature and notify staff when an intervention is needed could greatly reduce hypothermia and increase recognition of neonatal fever associated morbidity and mortality.

FINAL TPP: TEMPERATURE MONITOR (CONTINUOUS)

Table 29: Final TPP for Temperature Monitor (Continuous)

Final target product profile for Temperature Monitor (Continuous)		
**Characteristic**	**Optimal**	**Minimal**
SCOPE		
**Intended Use**	To provide ongoing diagnoses and monitoring of treatment of hypo- and hyperthermia	
**Target Operator**	For use in low- and middle-income countries by a wide variety of clinicians, including nurses, clinical officers, and pediatricians	
**Target Population**	Neonates (born at any gestational age and require ongoing care)	
**Target Setting**	Hospitals in low-resource settings, but, may be used in health facilities based on country guidelines	Hospitals in low-resource settings
SAFETY AND STANDARDS		

**Quality Management** [1]	ISO 13485:2016 Medical devices – Quality management systems -- Requirements for regulatory purposes	
**Regulation**	At least one of: CE marking, approved by US FDA or another stringent regulatory body of a founding member of IMDRF (e.g., Japan or Australia or Canada)	
TECHNICAL CHARACTERISTICS		
**Benchtop Measurement Accuracy**	±0.1°C	
**Clinical Measurement Accuracy**	±0.2°C	±0.3°C
**Time to Indicate Accurate Temperature**	< 60 seconds	< 90 seconds
**Alarm Characteristics**	Visual and Auditory	
**Alarm Limits**	Adjustable	36.5°C-37.5°C
**Patient Interface**	Interface is biocompatible and reusable	Interface is biocompatible
**Size**	Small footprint; portable and can be left at bedside	Same as Optimal
**Weight**	<500 grams	Same as Optimal
PURCHASING CONSIDERATIONS		
**Consumables**	> 12 months before required	> 6 months before required
**Instrument Pricing**	<$100 ex-works	<$200 ex-works
**Consumable Pricing**	<$50 per year ex-works	
UTILITY REQUIREMENTS		
**Power Source**	Mains with rechargeable battery	Mains with rechargeable battery
**Battery**	Rechargeable battery, >24hrs on a single charge	Rechargeable battery, >6hrs on a single charge
**Voltage**	Model must match the voltage and frequency of the purchasing country's local power grid (e.g., 110-120 VAC at 60 Hz or 220-240 VAC at 50 Hz)	
TRAINING AND MAINTENANCE		

**User Instructions**	User manual and additional training materials (checklists, videos, guides) in at least one national official language for the country of intended use. Attached to device with labels and markings where possible	User manual provided in at least one national official language
**Warranty**	5 years	1 year
**Decontamination**	Easy to clean with common disinfecting agents	Same as Optimal

[1] *There was not 75% voting agreement on the Minimal characteristic. Please refer to the TPP Report discussion for additional detail.*

CONSENSUS MEETING SUMMARY: TEMPERATURE MONITOR (CONTINUOUS)

To arrive at the final TPP for Temperature Monitor (Table 29), we conducted a pre-meeting survey to prioritize the items for discussion at the Consensus Meeting for characteristics that achieved below 75% agreement in the survey results (Table 30). An overview of the discussion at the Consensus Meeting of these characteristics is included below.

- **Clinical Measurement Accuracy**
  - Consensus was achieved in the room (without a Mentimeter vote) for the Minimal characteristic to be ±0.3°C.
  - *Minimal: ±0.3°C*
- **Time to Indicate Accurate Temperature**
  - Consensus was achieved in the room (without a Mentimeter vote) for the Optimal and Minimal characteristic.
  - *Optimal: < 60 seconds*
  - *Minimal: < 90 seconds*
- **Alarm Characteristics**
  - Consensus was achieved in the room (without a Mentimeter vote) for the Minimal characteristic to be both Visual and Auditory and equal to the Optimal characteristic. Clinicians noted that the ability to silence the auditory alarm (e.g., if baby has a fever) but maintain a visual alarm would be useful. Product developers noted that according to the International Standards, "means shall be provided to inactive the alarms" [46].
  - *Optimal: Visual and Auditory*
  - *Minimal: Visual and Auditory (same as Optimal)*
- **Alarm Limits**
  - Consensus was achieved in the room for the Minimal characteristic to be 36.5°C-37.5°C. A vote was conducted to determine the lower bound of this range limit for the Minimal characteristic.
  - *Minimal: Lower bound of 36.5°C or 36°C*

        - Overall Vote - 74% voted “36.5°C” (n = 19)
        - Clinicians - 81% voted “36.5°C” (n = 16)
        - Excluding involvement with product development - 75% voted “36.5°C” (n = 16)
- **Battery (previously titled 'Battery Power')**
    - There was agreement in the room that all characteristics relating to Utility Requirements (e.g., Back-up Battery; Battery Power; Batteries; Voltage; Power Requirement; Maximum Power Consumption; Response During Power Outage; Surge Protection, Electrical Plug) be reviewed and harmonized following the TPP meeting.
    - There was a discussion in the room emphasizing the importance of reliable power supply for minimum of 24 hours. Clinicians noted that the intention is to leave the device on for 24 hours, hence the time period. Discussion in the room encouraged product developers to be creative (e.g., device could plug into wall, connect with other devices, etc.). Clinicians noted a preference to avoid wired connections to mains and emphasized that “there are already too many wires”. There was agreement in the room that if the device was not connected to a mains power source, constant power for 24 hours would be required, however, if it was connected to a mains power source, then 12 hours back-up for power shedding would be sufficient for the Optimal characteristic. For the Minimal characteristic, if the device was not connected to a mains power source, constant power for 24 hours would be required, however, if the device was connected to a mains power source, then at least 6 hours of back-up for power shedding should be required.
    - *Optimal: Rechargeable battery, >24hrs on a single charge*
    - *Minimal: Rechargeable battery, >6hrs on a single charge*
- **Voltage**
    - There was agreement in the room that all characteristics relating to Utility Requirements (e.g., Back-up Battery; Battery Power; Batteries; Voltage; Power Requirement; Maximum Power Consumption; Response During Power Outage; Surge Protection, Electrical Plug) be reviewed and harmonized following the TPP meeting.
    - *Optimal and Minimal: Model must match the voltage and frequency of the purchasing country’s local power grid (e.g., 110-120 VAC at 60 Hz or 220-240 VAC at 50 Hz)*
- **Instrument Pricing**
    - Consensus was achieved in the room (without a Mentimeter vote) for the Minimal characteristic to remain unchanged at <$200 ex-works for Instrument Pricing. Participants noted that since no products currently exist on the monitor to continuously monitor temperature (i.e., not a spot check thermometer) it is difficult to quantify a price.
    - *Minimal: <$200 ex-works*

DELPHI-LIKE SURVEY: TEMPERATURE MONITOR

Table 30: Delphi-like survey results for Temperature Monitors TPP prior to Consensus Meeting (data as of Oct 25, 2019)

	Optimal		Minimal		
**Characteristic**	**Optimal requirement**	**% agreement (n size)**	**Minimal requirement**	**% agreement (n size)**	**Collated comments from Delphi-like survey**
**Intended Use**	Optimal: To provide ongoing diagnoses and monitoring of treatment of hypo- and hyperthermia.	**91%**<br>**n = 11**	Minimal: Same as Optimal.	**90%**<br>**n = 10**	3 comments as summarized below<br>• Intended use of this is hard to imagine.<br>• Possibilities are: preventing HYPO thermal in babies who:<br>o (1) don't have mother's available for KMC<br>o (2) are too sick for KMC<br>o (3) are transitioning from KMC to more time open crib<br>• If the aim is to build Comprehensive, NOT intensive newborn care units, temperature monitoring (if worth the lift, which I'm not convinced it is) would be really targeted?<br>• Diagnosis is different than measurement--> diagnosis can be up to the clinician, but the temperature monitor should provide an accurate readout that informs the diagnosis.<br>• Need to define skin temp vs. core temp
**Target Operator**	Optimal: For use in low- and middle-income countries by a wide variety of clinicians, including nurses, clinical officers, and pediatricians.	**100%**<br>**n = 10**	Minimal: Same as Optimal	**100%**<br>**n = 9**	2 comments as summarized below<br>• Maybe refer to training levels or years of training<br>• There could be application for home-use of CHWs
**Target Population**	Optimal: Neonates (<28 days)	**100%**<br>**n = 10**	Minimal: Same as Optimal.	**78%**<br>**n = 9**	2 comments as summarized below<br>• May be useful if accurate in older children.<br>• Perhaps population should also include all young infants under 3 months of age

	Optimal		Minimal		
**Target Setting**	Optimal: Hospitals in low-resource settings	**90%**<br>**n = 10**	Minimal: Same as Optimal.	**67%**<br>**n = 9**	3 comments as summarized below<br>• Useful in additional settings<br>o Home<br>o Community<br>o Lower-level health centres<br>o Transport of small and sick newborns
**International Standard**	Optimal: ISO 13485:2016 Medical devices – Quality management systems -- Requirements for regulatory purposes.	**86%**<br>**n = 7**	Minimal: Same as Optimal.	**83%**<br>**n = 6**	2 comments as summarized below<br>• "Accuracy less of an issue"<br>• See feedback from other Product Categories
**Regulation**	Optimal: CE marking or US FDA Clearance	**71%**<br>**n = 7**	Minimal: Same as Optimal.	**67%**<br>**n = 6**	2 comments<br>• These devices require minimal regulation
**Benchtop Measurement Accuracy**	Optimal: ±0.1°C	**86%**<br>**n = 7**	Minimal: Same as Optimal.	**83%**<br>**n = 6**	4 comments as described below<br>• Should increase to +/-0.3 C to match warming crib and radiant warmers<br>• 0.1 C is way too strict<br>• Theme: Benchtop vs. Clinical is not understood<br>• This will not alter a clinical decision
**Clinical Measurement Accuracy**	Optimal: ±0.2°C	**78%**<br>**n = 9**	Minimal: ±0.5°C	**71%**<br>**n = 7**	2 comments as summarized below<br>• Should increase, way too strict, should match warming crib and radiant warmer<br>o Optimal: ±0.5°C<br>o Minimal: Same as Optimal<br>• 0.1. For a home thermometer its 0.2
**Time to Indicate Accurate Temperature**	Optimal: < 90 seconds	**50%**<br>**n = 8**	Minimal: < 3 minutes	**29%**<br>**n = 7**	5 comments as summarized below<br>• I'm not sure I understand this parameter? Is this how often the monitor refreshes? If so, then I think the interval could be longer

	Optimal		Minimal		
					• Presuming this device would also be used for spot check of temperature (as opposed to continuous monitoring), I would suggest <30 seconds for Optimal and <90 seconds for minimal-essential for clinicians to be able to obtain temperature measurements quickly in a high-volume, resource-constrained environment<br>• Too long<br>• Much faster. For a home thermometer it's 3s<br>• Way too short! Also should specific clinical (not benchtop) - manufacturers could list benchtop instead because it is a lot faster. Suggest update to: Time to Indicate Accurate Clinical Temperature<br>o Optimal: < 3 minutes<br>o Minimal: < 5 minutes
**Alarm Characteristics**	Optimal: Visual and Auditory	**80%**<br>**n = 10**	Minimal: Visual	**56%**<br>**n = 9**	5 comments as summarized below<br>• Theme: Minimal should include audio<br>• Depends on the use case. Almost never needed unless continuous monitoring
**Alarm Limits**	Optimal: Adjustable	**78%**<br>**n = 9**	Minimal: 36.5°C-37.5°C	**57%**<br>**n = 7**	4 comments as summarized below<br>• Not sure why this would be adjustable? Seems like it should just be set at the cut offs for fever and hypothermia? 36.5 and 38?<br>• Technically easy to make this wider<br>• This depends on the type of alarm (visual or auditory), but as with all alarms they should be pre-set.<br>• Take this down to 35.5C-37.5°C
**Consumables**	Optimal: > 12 months before required	**89%**<br>**n = 9**	Minimal: > 6 months before required	**75%**<br>**n = 8**	2 comments as summarized below<br>• Depends on use... How will you quantify? Are there shelf life considerations?

	Optimal		Minimal		
**Decontamination**	Optimal: Easy to clean with common disinfecting agents	**90%** **n = 10**	Minimal: Same as Optimal.	**89%** **n = 9**	1 comment as summarized below<br>• Cleaning and disinfecting is not the same
**Battery Power**	Optimal: >4 hour on single charge	**80%** **n = 10**	Minimal: None	**56%** **n = 9**	5 comments as summarized below<br>• Must have battery<br>• Optimal battery life of 7 days (with sampling frequency of 5 minutes). A study of a related device in India reported battery life up to 28 days with sampling frequency of 5 minutes (https://innovations.bmj.com/content/4/2/60).<br>• Minimal battery life of 24 hours (with sampling frequency of 5 minutes)
**Voltage**	Optimal: 110-240V 50-60hz	**89%** **n = 9**	Minimal: 220-240V 50-60hz	**63%** **n = 8**	3 comments as summarized below<br>• Consider whether it is actually possible to have 110-220v? isn't it switched from one to the other before use if it's made for both?
**Patient Interface**	Optimal: Interface is biocompatible and reusable	**100%** **n = 10**	Minimal: Interface is biocompatible	**88%** **n = 8**	0 comments
**Size**	Optimal: Small footprint; portable and can be left at bedside	**100%** **n = 10**	Minimal: Same as Optimal.	**78%** **n = 9**	4 comments summarized below<br>• Could the minimum standard be a handheld portable temperature monitor and probe with a dock?<br>• Small footprint is difficult to measure<br>• Small increases likelihood of disappearing
**Weight**	Optimal: <500 grams	**100%** **n = 9**	Minimal: Same as Optimal.	**88%** **n = 8**	1 comment<br>See feedback from other Product Categories with Weight

	Optimal		Minimal		
**User Manual**	Optimal: User manual and additional training materials (checklists, videos, guides) in English and local language. Attached to device with labels and markings where possible.	**89%**<br>**n = 9**	Minimal: User manual provided.	**88%**<br>**n = 8**	2 comments<br>See feedback from other Product Categories with User Manual
**Warranty**	Optimal: 5 years	**100%**<br>**n = 9**	Minimal: 1 year	**71%**<br>**n = 7**	3 comments as summarized below<br>• 5 years is too long | 1 year is too short
**Instrument Pricing**	Optimal: <$100 ex-works	**75%**<br>**n = 8**	Minimal: <$200 ex-works	**57%**<br>**n = 7**	4 comments as summarized below<br>• Raise minimal price to $300?<br>• I wonder if I would want a dedicated temperature monitor at all.... why not have one that also monitors SPO2, heart rate?<br>• If it's just temp, should be much less, the tech isn't that crazy<br>• Difficult to suggest target price, as unclear whether this monitor would provide continuous temperature monitoring and, if so, at what frequency measurements would take place. Further, it would be helpful to know if the device would include Bluetooth or a related wireless system to enable data storage and/or remote monitoring (this would be ideal). A digital neonatal thermometer costs less than $5 in most low-resource settings ($5 for pack of 8 in Uganda), whereas combination monitor (temp/HR/SpO2) is estimated to cost ~$50/device including sensor and tablet interface. Presuming this temp monitor would provide continuous measurements, I would provisionally suggest an Optimal target price of <$50 though clearly depends

	Optimal		Minimal		
					on measurement frequency and wireless data transmission capability
**Consumable Pricing**	Optimal: <$50 per year ex-works	**86%**<br>**n = 7**	Minimal: Same as Optimal.	**83%**<br>**n = 6**	2 comments as summarized below<br>• Ideally it's reusable and doesn't require consumables<br>• Depends on use. Impossible to say

Figure 30: Summary of organizational affiliation for Temperature Monitor TPP from Delphi-like Survey prior to Consensus Meeting (data as of Oct 25, 2019)

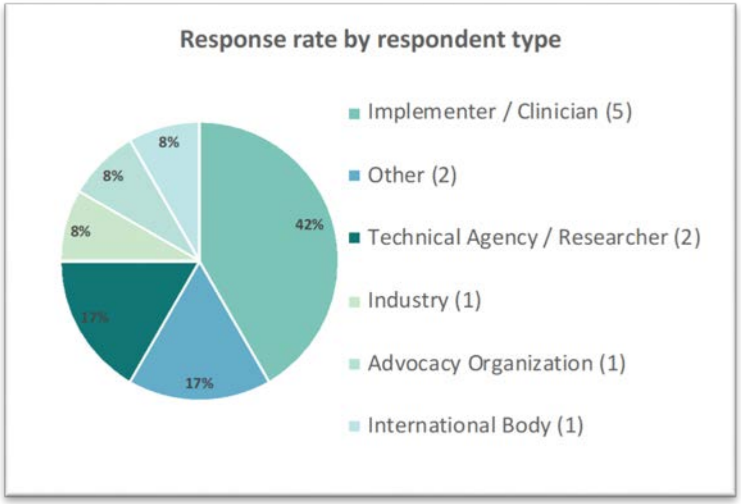

Respondent type	Percentage
Implementer / Clinician (5)	42%
Other (2)	17%
Technical Agency / Researcher (2)	17%
Industry (1)	8%
Advocacy Organization (1)	8%
International Body (1)	8%

Figure 31: Summary of response rate by country for Temperature Monitor TPP from Delphi-like Survey prior to Consensus Meeting (data as of Oct 25, 2019)

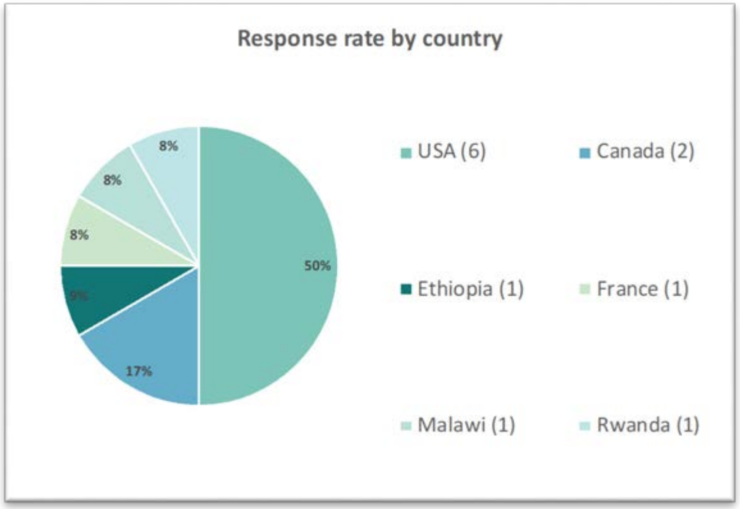

Country	Percentage
USA (6)	50%
Canada (2)	17%
Ethiopia (1)	8%
France (1)	8%
Malawi (1)	8%
Rwanda (1)	8%

CONDUCTIVE WARMER

INTRODUCTION: CONDUCTIVE WARMER

Since low birth weight or sick newborns are most vulnerable to hypothermia, the World Health Organization has outlined various methods that can be used to keep high-risk babies warm including kangaroo-mother care, "warm rooms", heated mattresses, radiant warmers, and incubators. These methods vary in their response to addressing the four different ways in which newborns lose heat: radiation, convection, evaporation, and conduction [74].

Conductive warmers provide conductive heating either below or around the patient while also allowing health care workers with visibility and access to the baby. Given the high cost of some warming devices (e.g., incubator, radiant warmer), a need exists in low-resource settings for a technology that is both affordable and easy to use, and that can accurately detect hypothermia while keeping the newborn warm. The advantages of using warming devices include the fact that extra warmth can be given locally instead of having to warm the whole room; temperature control is easier; and newborns can be fully observed and visible. The World Health Organization explains that different devices serve different purposes and advises that incubators are the proper choice for the care of very small newborns during the first few days or weeks. When these babies no longer have acute problems, they can be cared for safely on heated water-filled mattresses. Radiant heaters are best used for resuscitation and interventions where a number of people are involved [74].

Negative outcomes associated with hypothermia can be prevented using warming cribs that carefully control heat. Conductive warmers may be called warming cribs however are distinct from incubators. The intent in the development of this TPP was to provide developers with the opportunity to be innovative in the design process rather than be constrained by existing technologies or preconceived notions that a "crib" must be enclosed.

A need for the creation of a separate TPP for an incubator was identified at the Consensus Meeting. Incubators are the conventional method for maintaining normothermia in preterm and low birthweight neonates. Risks associated with incubator care include hypothermia [54,55]; hyperthermia [56]; nosocomial infections, related to lack of effective cleaning standards [57-59]; and cross-infection from other neonates when incubators are shared, a common practice in low-resource facilities. Failure of incubators to properly regulate temperature may be related to malfunction (e.g., over- or under-heating) [56,59-62], loss of electrical supply [63], ignorance of how to regulate set-points [56], as well as environmental factors [60]. In low- and middle-income countries, where there may be few nurses and doctors available, neonates in incubators may not receive adequate

monitoring and serious events (e.g., apnea) may not be detected in time. Due to high purchase cost and poor routine maintenance practices, hospitals in such settings commonly face shortages of functional incubators [52,63-65].

FINAL TPP: CONDUCTIVE WARMER

Table 31: Final TPP for Conductive Warmer

Final target product profile for Conductive Warmer		
**Characteristic**	**Optimal**	**Minimal**
SCOPE		
**Intended Use**	Treatment and prevention of hypothermia in neonates requiring thermal care	
**Target Operator**	For use in low- and middle-income countries by a wide variety of clinicians, including nurses, clinical officers, and pediatricians	
**Target Population**	Neonates (born at any gestational age and require ongoing care)	
**Target Setting**	Hospitals in low-resource settings	
SAFETY AND STANDARDS		
**Quality Management** [6]	ISO 13485:2016 Medical devices – Quality management systems -- Requirements for regulatory purposes	
**Regulation**	At least one of: CE marking, approved by US FDA or another stringent regulatory body of a founding member of IMDRF (e.g., Japan or Australia or Canada)	
TECHNICAL CHARACTERISTICS		
**Form Factor**	Enclosed or not enclosed (no preference)	
**Benchtop Measurement Accuracy Conductive Surface Temperature**<br><br>**Temperature of Baby** (required if servo-controlled)	Conductive Surface Temperature: Accuracy of control of contact surface temperature (measured) = ± 1°C (and not exceeding 41°C) [1]<br><br>Accuracy of baby's temperature: +0.2°C [2]	

**Clinical Measurement Accuracy** (Compare to another gold standard)	Known	Not required
**Maximum CO2 Concentration** (If Enclosed Device)	0.50%	
**Maximum Temperature** (of the conductive surface)	40°C [3]	
**Humidification** (If Enclosed device)	Humidity control for babies less than 1kg	None
**Surface Temperature overshoot when the temperature control is set to its maximum setting**	1°C [4]	
**Time to Indicate Accurate Temperature of baby**	< 90 seconds	<5 minutes [2]
**Uniformity** (If Enclosed, then uniformity of air) (If Not Enclosed, then uniformity of mattress)	Air Temperature: < 0.8°C (for enclosed only) [5]<br>Conductive Surface (for enclosed and not enclosed) [2]:<br>• High Heat: < 1°C<br>• Low Heat: < 0.5°C	
**Alarm Characteristics**	Visual and Auditory	
**Patient Interface**	Interface is biocompatible and reusable	Interface is biocompatible
**Patient Accessibility and Visibility**	Patient is visible and accessible to healthcare worker	
**Temperature Control**	Based on infant's temperature and includes manual and failsafe mode	Manual control and includes fail-safe mode
**Operating Conditions**	Describe in user manual how warming device is impacted by ambient temperatures in the operating environment	

PURCHASING CONSIDERATIONS		
**Consumables** (probes)	> 12 months before required	> 6 months before required
**Instrument Pricing**	<$500 ex-works	<$1,000 ex-works
**Consumable Pricing**	<$50 per year ex-works	<$100 per year ex-works
UTILITY REQUIREMENTS		
**Power Source**	Mains Power	Mains Power
**Power Consumption**	<250W maximum	<800W maximum
**Voltage**	Model must match the voltage and frequency of the purchasing country's local power grid (e.g., 110-120 VAC at 60 Hz or 220-240 VAC at 50 Hz)	
TRAINING AND MAINTENANCE		
**User Manual**	User manual and additional training materials (checklists, videos, guides) in at least one national official language for the country of intended use. Attached to device with labels and markings where possible	User manual provided in at least one national official language
**Warranty**	5 years	1 year
**Decontamination**	Easy to clean with common disinfecting agents	

[1] *Source: IEC 80601-2-35, Section 201.12.4.104 [75]*

[2] *Source: ISO 80601-2-56, Section 201.101.3 [76]*

[3] *Source: IEC 80601-2-35, Section 201.11.1.2.1.101.1 [77]*

[4] *Source: IEC 80601-2-35, Section 201.12.4.103 [78]*

[5] *Source: IEC 60601-2-19, Section 201.12.1.102 [79]*

[6] *There was not 75% voting agreement on the Minimal characteristic. Please refer to the TPP Report discussion for additional detail.*

CONSENSUS MEETING SUMMARY: CONDUCTIVE WARMER

To arrive at the final TPP for Conductive Warmer (Table 31), a smaller group convened at the TPP Consensus meeting to determine which characteristics should be included in a brand new TPP for a Conductive Warmer. A need for a new TPP arose when it was determined that there separate TPPs were required based on the method of heating. Three methods of heating were outlined:

1) Radiant Heat (e.g., Radiant Warmer / resuscitaire)
2) Conductive Heat (e.g., Conductive Warmer)
3) Convective Heat (e.g., Incubator)

The smaller group discussion focused on the Conductive Warmer TPP as standards for incubators in high-resource settings currently exist. It was noted that there is a potential need for adjustment of these incubator standards for low-resource settings. Note that a pre-meeting survey for a Warming Crib was conducted and survey results are included in Table 32.

The following Product Specific Standards were highlighted:

- IEC 80601-2-35, Section 201.12.4.104 [75]
- ISO 80601-2-56, Section 201.101.3 [76]
- IEC 80601-2-35, Section 201.11.1.2.1.101.1 [77]
- IEC 80601-2-35, Section 201.12.4.103 [78]
- IEC 60601-2-19, Section 201.12.1.102 [79]

DELPHI-LIKE SURVEY: WARMING CRIB

Table 32: Delphi-like survey results for Warming Crib TPP prior to Consensus Meeting (data as of Oct 25, 2019)

	Optimal		Minimal		
**Characteristic**	**Optimal requirement**	**% agreement (n size)**	**Minimal requirement**	**% agreement (n size)**	**Collated comments from Delphi-like survey**
**Intended Use**	Optimal: Treatment and prevention of hypothermia in neonates requiring intensive thermal care.	73%<br>n = 11	Minimal: Same as Optimal.	88%<br>n = 8	4 comments as summarized below<br>• Theme: Remove the word "intensive"<br>• Theme: A variety of proposed Intended Use language<br>o Optimal: treatment and prevention of hypothermia in stable and unstable at risk neonates not receiving (mother/caretaker

	Optimal		Minimal		
					o not available) or not eligible (too sick or too small) to receive KMC<br>o Optimal: Treatment and prevention of hypothermia in neonates requiring thermal care<br>o Defining treatment as rapidly warming a patient and preventing hypothermia by safely keeping the baby normothermic - I would accept that a warming crib could only prevent hypothermia, as long as another device was available to rapidly warm a patient e.g. radiant warmer
**Target Operator**	Optimal: For use in low- and middle-income countries by a wide variety of clinicians, including nurses, clinical officers, and pediatricians.	**100%**<br>**n = 10**	Minimal: Same as Optimal	**100%**<br>**n = 9**	0 comments
**Target Population**	Optimal: Neonates (<28 days)	**91%**<br>**n = 11**	Minimal: Same as Optimal.	**100%**<br>**n = 10**	2 comments as summarized below<br>• Minimal: Neonates <28 days<br>• Optimal: Minimal + treat babies over 28 days old e.g. KMC babies who have clinically deteriorated
**Target Setting**	Optimal: Hospitals in low-resource settings	**82%**<br>**n = 11**	Minimal: Same as Optimal.	**100%**<br>**n = 10**	4 comments as summarized below<br>• Theme: Broaden Target Setting<br>o Minimal: hospital in resource-limited settings, Optimal: health centres (primary)<br>o Transport
**International Standard**	Optimal: ISO 13485:2016 Medical devices – Quality management systems -- Requirements for regulatory purposes.	**100%**<br>**n = 5**	Minimal: Same as Optimal.	**100%**<br>**n = 4**	0 comments

	Optimal		Minimal		
**Regulation**	Optimal: CE marking or US FDA Clearance	**86%**<br>**n = 7**	Minimal: Same as Optimal.	**67%**<br>**n = 6**	2 comments as summarized below<br>• Theme: Reduce regulatory options or add more flexibility<br>• CE Mark alone is sufficient
**Benchtop Measurement Accuracy**	Optimal: ±0.3°C	**80%**<br>**n = 10**	Minimal: Same as Optimal.	**89%**<br>**n = 9**	2 comments<br>• Unclear what benchtop vs clinical accuracy means and how that would be measured/reported. Potentially combine?<br>• +- 0.1 is Optimal
**Clinical Measurement Accuracy**	Optimal: ±0.5°C	**80%**<br>**n = 10**	Minimal: Same as Optimal.	**89%**<br>**n = 9**	2 comments<br>• Assuming that this refers to the temperature of the baby e.g. through skin temperature probe, it is difficult to comment on what this number should be as we do not know deviance between bench testing (as above) and real-world. We can only really design to meet a bench-testing level<br>• +-0.1
**Heat Retention**<br>*(corrected from 'Pressure')*	Optimal: < 5°C loss over 4 hours	**78%**<br>**n = 9**	Minimal: None.	**88%**<br>**n = 8**	2 comments<br>• In my mind, a warming crib should not lose heat but stay at a constant temperature<br>• Is this the retention of heat within the baby or the device (mattress/air etc.)
**Maximum CO2 Concentration**	Optimal: 0.005%	**100%**<br>**n = 7**	Minimal: Same as Optimal.	**100%**<br>**n = 6**	1 comment<br>• The CO2 concentration in air is approx. 0.04% so I think you may have an extra zero in the number<br>• With incubators being a closed environment, the CO2 concentration will be higher at times. The International Standards leave manufacturers to specify a CO2 level following a specified test (IEC 60601-2-19 clause 201.12.4.2.101)

	Optimal		Minimal		
					• Atom, a recognized Japanese incubator manufacturer, state that the CO_2 level for their V-2100 incubator is 0.4% following this test. Quote "CO_2 concentration when stability has been achieved after administering air mixed with 4% CO_2 to a point 10cm above the center of the mattress at 750mL/min doesn't exceed 0.4%."
**Maximum Rate of Change in Infant's Temperature**	Optimal: 0.5°C/hour	**75%**<br>**n = 8**	Minimal: Same as Optimal.	**86%**<br>**n = 7**	3 comments as summarized below<br>• 1 degree per hour should be better<br>• This is assuming closed-loop control with sensor. That was not mentioned above so may be confusing<br>• In the case of incubators, there are specific standards to follow and we are not in a position to comment on how quickly a baby will warm up or lose heat as this will depend on their clinical state
**Maximum Temperature**	Optimal: 38.0°C	**56%**<br>**n = 9**	Minimal: Same as Optimal.	**75%**<br>**n = 8**	5 comments as summarized below<br>• Should specify that this is referencing "Maximum Air Temperature"<br>• Need to clarify what temperature this is. Is it baby, air, pad?<br>• Maximum temperature should be 37.5°C<br>• This may be specific to incubators (most of which actually go as high as 39°C) but feedback from all users indicate that they are never set above 36.5°C and rarely higher than 36°C (note, I refer to the temperature of the air, not the baby)
**Overshoot**	Optimal: < 2°C	**57%**<br>**n = 7**	Minimal: Same as Optimal.	**50%**<br>**n = 6**	3 comments as summarized below<br>• Clarity on the parameter needed<br>• Additional values were suggested<br>o <0.5<br>o +-1 C

	Optimal		Minimal		
**Time to Indicate Accurate Temperature**	Optimal: < 90 seconds	**80%**<br>**n = 10**	Minimal: < 3 minutes	**44%**<br>**n = 9**	5 comments as summarized below<br>• Clarity on the parameter needed<br>• 3 minutes viewed as too long by respondents (e.g., 30 seconds suggested) but may not be technically feasible<br>• Need to specify that it's clinical (not benchtop) and these are more realistic thresholds<br>• Time to Indicate Accurate Clinical Temperature<br>o Optimal: < 3 minutes<br>o Minimal: < 5 minutes
**Uniformity**	Optimal: < 1°C	**100%**<br>**n = 8**	Minimal: Same as Optimal.	**100%**<br>**n = 7**	0 comments
**Alarm Characteristics**	Optimal: Visual and Auditory	**91%**<br>**n = 11**	Minimal: Visual	**30%**<br>**n = 10**	5 comments as summarized below<br>• Theme: Minimal should include audio<br>• Minimal could be turns itself off if certain temperature reached<br>• What is alarm for? Baby temp?
**Alarm Limits**	Optimal: Adjustable	**64%**<br>**n = 11**	Minimal: 36.5°C-37.5°C	**89%**<br>**n = 9**	5 comments as summarized below<br>• Clarify if air or baby temperature. Assuming this is air temperature rather than skin temperature, users would want the alarm to sound on deviation from set temperature<br>• Adjustable may not be an advantage<br>• Listing the alarm limits as adjustable is misleading, would propose updating to:<br>o Optimal: +/-0.5 of baby set temperature<br>o Minimal: 36.5°C-37.5°C

	Optimal		Minimal		
**Consumables**	Optimal: > 12 months before required	**100%**<br>**n = 9**	Minimal: > 6 months before required	**100%**<br>**n = 8**	3 comments as summarized below<br>• Agree for almost everything, the exception being air filters which should be checked and possibly replaced after 3 months<br>• Optimal would add some sort of automated features that lets you know when consumables needs to be replaced
**Decontamination**	Optimal: Easy to clean with common disinfecting agents	**100%**<br>**n = 11**	Minimal: Same as Optimal.	**100%**<br>**n = 10**	1 comment<br>• Would it be helpful to use a "time to clean/disinfect"?
**Maximum Power Consumption**	Optimal: <250 Watts	**100%**<br>**n = 8**	Minimal: <800 Watts	**67%**<br>**n = 6**	2 comments as summarized below<br>• 800 still high and not feasible at a solar system<br>• Target minimal <500
**Voltage**	Optimal: 110-240V 50-60hz	**83%**<br>**n = 6**	Minimal: 220-240V 50-60hz	**100%**<br>**n = 5**	
**Operating Temperature**	Optimal: Harsh ambient condition, temperature 5-45 °C, humidity 15% to 95%, dusty air, elevation >=2000 meters	**78%**<br>**n = 9**	Minimal: Harsh ambient temperature 10-40 °C, humidity 15%-95%, dusty air, elevation up to 2000 meters	**75%**<br>**n = 8**	3 comments as summarized below<br>• Too strict and not realistic environmental conditions, would suggest changing to: Optimal: Harsh ambient condition, indoor temperature (20-40 °C), humidity 30% to 80%, dusty air, elevation <=2000 meters<br>• An interesting question is raised when ambient temperature is greater than set temperature of the incubator
**Patient Interface**	Optimal: Interface is biocompatible and reusable	**100%**<br>**n = 10**	Minimal: Interface is biocompatible	**78%**<br>**n = 9**	2 comments as summarized below<br>• Reusable should be part of the minimal requirement

	Optimal		Minimal		
**Patient Accessibility and Visibility**	Optimal: Patient is visible and accessible to healthcare worker.	91%<br>n = 11	Minimal: Same as Optimal.	90%<br>n = 10	2 comments as summarized below<br>• Define visible and accessible
**Patient Size**	Optimal: Should fit a single infant <10kg	73%<br>n = 11	Minimal: Same as Optimal.	60%<br>n = 10	6 comments as summarized below<br>• Theme: Should the warming crib fit more than one baby or not<br>• This will be most critical in septic and new, preterm infants. So you need a lower limit (1kg) for which the warming crib also works<br>• 10 kg seems large for a neonate<br>• Should correspond to babies <28 days - 6kg max, 8 with contingency
**Temperature Control**	Optimal: Based on infant's temperature and includes fail-safe mode	90%<br>n = 10	Minimal: Same as Optimal.	78%<br>n = 9	2 comments as summarized below<br>• For the incubator, temp control is based on air temperature. User research early on identified risks with patient temp control e.g. probes not properly attached. Agree fail-safe mode required - if temp runs higher than set temp<br>• Should this also include a manual mode with simple settings? How does this spec limit developers to address the risks of multiple babies in one device?
**User Manual**	Optimal: User manual and additional training materials (checklists, videos, guides) in English and local language. Attached to device with labels and markings where possible.	100%<br>n = 11	Minimal: User manual provided.	90%<br>n = 10	1 comment<br>• User manuals are not used

	Optimal		Minimal		
**Warranty**	Optimal: 5 years	**91%**<br>**n = 11**	Minimal: 1 year	**70%**<br>**n = 10**	2 comments<br>• Theme: 5 years too long | 1 year too short
**Instrument Pricing**	Optimal: <$500 ex-works	**78%**<br>**n = 9**	Minimal: <$1,000 ex-works	**63%**<br>**n = 8**	4 comments<br>• These limits would not be relevant for Incubators, with volume, in the long-term, getting close to $1,000 could be achievable.<br>• This is extremely expensive for a resource poor setting, considering the large number of patients which would benefit from this device
**Consumable Pricing**	Optimal: <$50 per year ex-works	**88%**<br>**n = 8**	Minimal: <$100 per year ex-works	**86%**<br>**n = 7**	2 comments<br>• If referring to temperature probes these should ideally be cheaper as they will receive intensive use, thus requiring frequent replacement<br>• The requirement for a battery may increase this. Excluding this $50 is aspirational and $100 is achievable but challenging.

Figure 32: Summary of organizational affiliation for Warming Crib TPP from Delphi-like Survey prior to Consensus Meeting (data as of Oct 25, 2019)

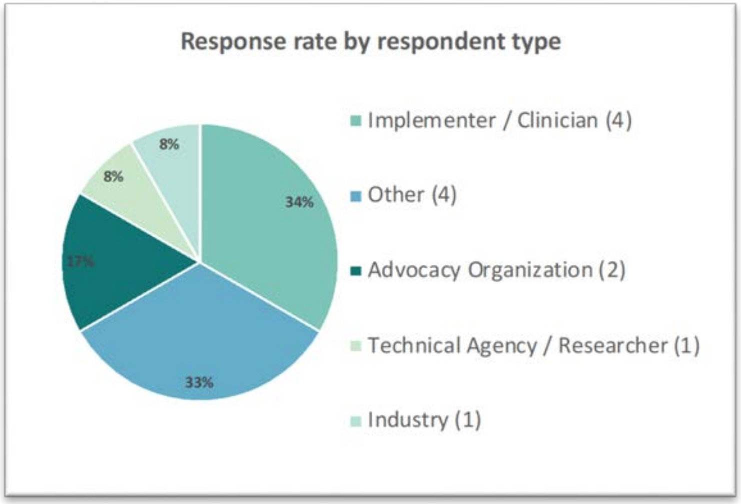

Respondent type	Percentage
Implementer / Clinician (4)	33%
Other (4)	33%
Advocacy Organization (2)	17%
Technical Agency / Researcher (1)	8%
Industry (1)	8%

Figure 33: Summary of response rate by country for Warming Crib TPP from Delphi-like Survey prior to Consensus Meeting (data as of Oct 25, 2019)

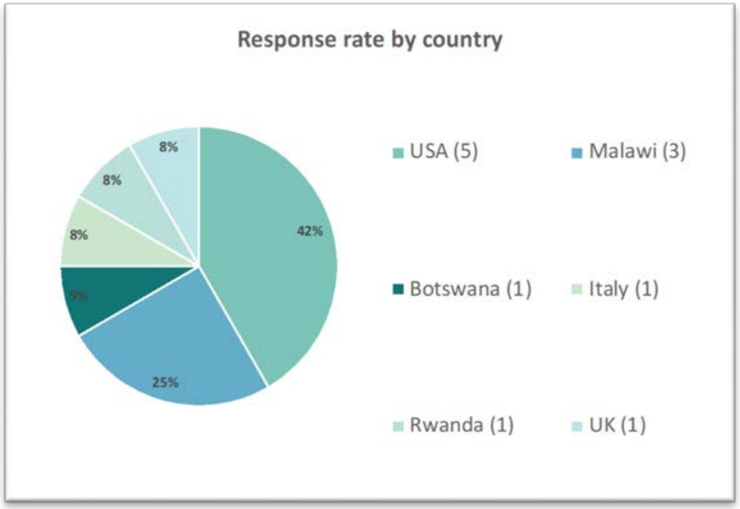

Country	Percentage
USA (5)	42%
Malawi (3)	25%
Botswana (1)	8%
Italy (1)	8%
Rwanda (1)	8%
UK (1)	8%

APPENDIX A: DELPHI-LIKE SURVEY RESPONDENT ORGANIZATIONAL DESIGNATION

3rd Stone Design
Abuja University Teaching Hospital
Alex Ekwueme Federal University Teaching Hospital Abakaliki
Baylor College of Medicine
BC Children's Hospital
Burnet Institute
CCBRT Dar es Salaam
CENETEC-Salud
Center for Public Health and Development (CPHD)
Children's Hospital of Philadelphia
Christian Medical College, Vellore
Clinton Health Access Initiative
College of Medicine, University of Lagos
College of Medicine, University of Malawi
Dartmouth
Day One Health
Diamedica UK Ltd
D-Rev
Egerton University - Nakuru County Referral Hospital
ETH Zurich
Fishtail Consulting
FREO2 Foundation Australia
Global Strategies
Hawassa University
Independent Biomedical Engineer
Institute for Healthcare Improvement
intelms.com
Kamuzu Central Hospital
Kamuzu College of Nursing
Kemri-Wellcome Trust
Kenya Paediatric Association
Komfo Anokye Teaching Hospital
Malawi-Liverpool Wellcome Trust
Mama Lucy Hospital
Masimo
Mbarara University of Science and Technology
McGill University Health Centre
McMaster University
Medecins Sans Frontieres
Mediquip Global Limited
Ministry of Health, Senegal
mOm Incubators
MRC Gambia at LSHTM
Muhimbili National Hospital
Muhimbili University of Health and Allied Sciences (MUHAS)
Neopenda
No designation listed (10)
Pediatric and Child Health Association in Malawi
Pumwani Hospital
Queen Elizabeth Central Hospital
Rice 360 Institute for Global Health
Royal Children's Hospital and Centre for International Child Health (University of Melbourne)
Save The Children
Texas Children's Hospital
The University of Queensland
UCSF and London School of Hygiene & Tropical Medicine
UNICEF
University of Alabama at Birmingham
University of British Columbia
University of Global Health Equity
University of Maiduguri Teaching Hospital, Maiduguri
University of Nairobi
UNTH, Enugu

APPENDIX B: CONSENSUS MEETING PARTICIPATION

Albert Manasyan (University of Alabama Birmingham)
Anna Worm
Antke Zuechner (CCBRT)
Audrey Chepkemoi (Moi Teaching and Referral Hospital)
Bentry Tembo (Kamuzu Central Hospital)
Bev Bradley (UNICEF)
Casey Trubo (D-Rev)
Chishamiso Mudenyanga (Clinton Health Access Initiative)
Danica Kumara (3rd Stone Design)
Daniel Wald (D-Rev)
Edith Gicheha (Kenya Pediatric Research Consortium)
Emily Ciccone (University of North Carolina - Chapel Hill)
Emmie Mbale (PACHA)
Grace Irimu (University of Nairobi)
Guy Dumont (The University of British Columbia)
Helga Naburi (Muhimbili National Hospital)
Jeffrey Pernica (McMaster University)
John Appiah (Kumfo Anokye Teaching Hospital)
Jonathan Strysko (Children's Hospital of Philadelphia/Princess Marina Hospital)
Joy Lawn (London School of Hygiene and Tropical Medicine)
Lincetto Ornella (WHO)
Liz Molyneux (College of Medicine, Malawi)
Lizel Lloyd (Stellenbosch University)
Mamiki Chise
Marc Myszkowski
Maria Oden (Rice University)
Martha Franklin Mkony (Muhimbili National Hospital)
Martha Gartley (Clinton Health Access Initiative)
Mary Waiyego (Pumwani Maternity Hospital)
Matthew Khoory (mOm Incubators)
Melissa Medvedev (University of California, San Francisco; London School of Hygiene and Tropical Medicine)
Msandeni Chiume (Kamuzu Central Hospital)
Naomi Spotswood (Burnet Institute)
Norman Lufesi (Ministry of Health Malawi)
Pascal Lavoie (University of British Columbia)
Queen Dube (College of Medicine, Malawi)
Rachel Mbuthia (GE Healthcare)
Rebecca Richards-Kortum (Rice University)
Rhoda Chifisi (Kamuzu Central Hospital)
Rita Owino (GE Healthcare)
Robert Moshiro (Muhimbili National Hospital)
Ronald Mbwasi (Kilimanjaro Christian Medical Centre)
Sam Akech (KEMRI-Wellcome Trust Research Programme)
Sara Liaghati-Mobarhan (Rice University)
Sona Shah (Neopenda)
Steffen Reschwamm (MTTS)
Steve Adudans (CPHD/MQG)
Thabiso Mogotsi (University of Botswana)
Walter Karlen (ETH Zurich)
Zelalem Demeke (Clinton Health Access Initiative)

APPENDIX C: ABBREVIATIONS

°C	Degrees Celsius
bCPAP	Bubble continuous positive airway pressure
bpm	Beats per minute / Breaths per minute
CE Mark	Conformité Européenne – certification mark
cm	Centimeters
cm^2	Centimeter squared
CRP	C-reactive protein
CPAP	Continuous positive airway pressure
DHS	Demographic and health survey
FDA	Food and Drug Administration
HIS	Health information system
Hz	Hertz
IMR	Infant mortality rate
ISO	International Standards Organization
IV	Intravenous
KMC	Kangaroo Mother Care
kg	Kilogram
LPM	Liters per minute
LRS	Low-resource settings
MCH	Maternal and child health
MDG	Millennium Development Goal
Mg/dL	Milligrams per deciliter
mL/hr	Milliliters per hour
mmol/L	Millimoles per liter
µmol/L	Micromoles per liter
MMR	Maternal mortality rate
MNCH	Maternal, newborn, and child health
MNH	Maternal and neonatal health
nm	Nanometer
NMR	Neonatal mortality rate
PCT	Procalcitonin
PEEP	Positive end-expiratory pressure
PR	Pulse rate
RDS	Respiratory distress syndrome
ROP	Retinopathy of prematurity
SpO2	Peripheral saturation of oxygen
SDG	Sustainable Development Goal
TFR	Total fertility rate
U5MR	Under-5 mortality rate
UNFPA	United Nations Population Fund
USAID	U.S. Agency for International Development
uW	Micro Watts
W	Watt
WHO	World Health Organization

REFERENCES

To cite this article:

Kirby, R. & Palamountain, K. (2020). *Target product profiles for newborn care in low-resource settings (v1.0).*

[1] World Health Organization. (2019). Children: Reducing mortality [Fact sheet]. Retrieved from https://www.who.int/news-room/fact-sheets/detail/children-reducing-mortality.

[2] UNICEF Data. (2019). Delivery care. [Graph illustration of global delivery care coverage and trends from joint UNICEF/WHO database of skilled health personnel, 2019]. Retrieved from https://data.unicef.org/topic/maternal-health/delivery-care/.

[3] World Health Organization. (n.d.). Global Health Observatory (GHO) data: Neonatal mortality. Retrieved from https://www.who.int/gho/child_health/mortality/neonatal_text/en/.

[4] United Nations General Assembly resolution 70/1, Transforming our world: the 2030 Agenda for Sustainable Development, A/RES/70/1 (21 October 2015), available from https://undocs.org/en/A/RES/70/1.

[5] World Health Organization. (2019). Newborns: Reducing mortality [Fact sheet]. Retrieved from https://www.who.int/news-room/fact-sheets/detail/newborns-reducing-mortality.

[6] World Health Organization & the United Nations Children's Fund. (2019). *WHO-UNICEF technical specifications and guidance for oxygen therapy devices.* WHO medical device technical series. Licence: CC BY-NC-SA 3.0 IGO. Geneva, Switzerland: World Health Organization. Retrieved from https://apps.who.int/iris/bitstream/handle/10665/329874/9789241516914-eng.pdf.

[7] Caston-Gaa, A. & Ruparelia, C. S. (2018). Processing surgical instruments and medical devices (module 6). In M. S. Curless, C. S. Ruparelia, E. Thompson, & P. A. Trexler (Eds.), *Infection prevention and control: Reference manual for health care facilities with limited resources.* Baltimore, MD: JHPIEGO. Retrieved from http://reprolineplus.org/system/files/resources/IPC_M6_Instruments.pdf.

[8] The low-birth-weight infant. (1989). *Bulletin of the World Health Organization, 67*(Suppl), 68–84. Available from https://apps.who.int/iris/handle/10665/264624.

[9] Chawla, D., Agarwal, R., Deorari, A. K. & Paul, V. K. (2008). Fluid and electrolyte management in term and preterm neonates. *The Journal of Indian Pediatrics, 75*, 255–259. https://doi.org/10.1007/s12098-008-0055-0.

[10] Slusher, T., Vaucher, Y., Zamora, T. & Curtis, B. (2012). Feeding and fluids in the premature and sick newborns in the low-middle income countries (chapter 2). In: Ozdemir O (Ed.), *Contemporary Pediatrics*. Minneapolis, MN: IntechOpen. https://doi.org/10.5772/34879.

[11] Maynard, K. R., Causey, L., Kawaza, K., Dube, Q., Lufesi, N., Oden, Z. M., … & Molyneux, E. M. (2015). New technologies for essential newborn care in under-resourced areas: What is needed and how to deliver it. *Paediatrics and International Child Health, 35,* 192-205. https://doi.org/10.1179/2046905515Y.0000000034.

[12] March of Dimes; The Partnership for Maternal, Newborn & Child Health; Save the Children; & World Health Organization. (2012). *Born Too Soon: The Global Action Report on Preterm Birth.* Howson, C. P., Kinney, M. V., & Lawn, J. E. (Eds). Geneva, Switzerland: World Health Organization. Retrieved from https://www.who.int/pmnch/media/news/2012/201204_borntoosoon-report.pdf.

[13] U.S. Food & Drug Administration. (2018). Infusion Pumps. Retrieved from https://www.fda.gov/medical-devices/general-hospital-devices-and-supplies/infusion-pumps.

[14] Keay, S. & Callander, C. (2004). The safe use of infusion devices. *Continuing Education in Anaesthesia Critical Care & Pain, 4,* 81-85. https://doi.org/10.1093/bjaceaccp/mkh022.

[15] Clinical Laboratory Improvement Amendments of 1988 (CLIA) Proficiency Testing Regulations Related to Analytes and Acceptable Performance, 84 Fed. Reg. 1536 (proposed February 4, 2019). Retrieved from https://www.federalregister.gov/documents/2019/02/04/2018-28363/clinical-laboratory-improvement-amendments-of-1988-clia-proficiency-testing-regulations-related-to.

[16] Kumar, P., Chawla, D., & Deorari, A. (2011). Light-emitting diode phototherapy for unconjugated hyperbilirubinaemia in neonates. *Cochrane Database of Systematic Reviews, 2011*(12). https://doi.org/10.1002/14651858.CD007969.pub2.

[17] Morris, B. H., Tyson, J. E., Stevenson, D. K., Oh, W., Phelps, D. L., O'Shea, T. M., … & Higgins, R. D. (2013). Efficacy of phototherapy devices and outcomes among extremely low birth weight infants: Multi-center observational study. *Journal of Perinatology, 33,* 126-133. https://doi.org/10.1038/jp.2012.39.

[18] Eggert, P., Stick, C., & Schröder, H. (1984). On the distribution of irradiation intensity in phototherapy: Measurements of effective irradiance in an incubator. *European Journal of Pediatrics, 142,* 58-61. https://doi.org/10.1007/bf00442593.

[19] Mabey, D. C., Sollis, K. A., Kelly, H. A., Benzaken, A. S., Bitarakwate, E., Changalucha, J., … & Peeling, R. W. (2012). Point-of-care tests to strengthen health systems and save newborn lives: The case of syphilis. *PLoS Medicine, 9*(6). https://doi.org/10.1371/journal.pmed.1001233.

[20] Garcia, P. J., You, P., Fridley, G., Mabey, D. & Peeling, R. (2015). Point-of-care diagnostic tests for low-resource settings. *The Lancet Global Health, 3,* e257-e258. https://doi.org/10.1016/S2214-109X(15)70089-6.

[21] Peeling, R. W. & Mabey, D. (2010). Point-of-care tests for diagnosing infections in the developing world. *Clinical Microbiology & Infection, 16*(8),1062–1069. https://doi.org/10.1111/j.1469-0691.2010.03279.x.

[22] Kariya, T., Ito, N., Kitamura, T., & Yamada, Y. (2015). Recovery from extreme hemodilution (hemoglobin level of 0.6 g/dL) in cadaveric liver transplantation. *Anesthesia & Analgesia Case Reports, 4,* 132-136. https://doi.org/10.1213/XAA.0000000000000132.

[23] Lawn, J. E., Kerber, K., Enweronu-Laryea, C. & Cousens, S. (2010). 3.6 million neonatal deaths—what is progressing and what is not? *Seminars in Perinatology, 34,* 371–386. https://doi.org/10.1053/j.semperi.2010.09.011.

[24] Duke, T. (2005). Neonatal pneumonia in developing countries. *Archives of Disease in Childhood – Fetal and Neonatal Edition, 90,* F211–F219. https://doi.org/10.1136/adc.2003.048108.

[25] Bhutta, Z. A., Yusuf, K. & Khan, I. A. (1999). Is management of neonatal respiratory distress syndrome feasible in developing countries? Experience from Karachi (Pakistan). *Pediatric Pulmonology, 27,* 305–11. https://doi.org/10.1002/(SICI)1099-0496(199905)27:5<305::AID-PPUL2>3.0.CO;2-Q.

[26] Beck, S., Wojdyla, D., Say, L., Betran, A. P., Merialdi, M., Requejo, J. H, … & Van Look, P. F. (2010). The worldwide incidence of preterm birth: a systematic review of maternal mortality and morbidity. *Bulletin of the World Health Organization, 88*, 31–38. https://doi.org/10.2471/BLT.08.062554.

[27] World Health Organization. (2017). *WHO recommendations on newborn health: Guidelines approved by the WHO Guidelines Review Committee*. License: CC BY-NC-SA 3.0 IGO. Geneva, Switzerland. Retrieved from https://apps.who.int/iris/bitstream/handle/10665/259269/WHO-MCA-17.07-eng.pdf;jsessionid=F6B18E06280D4C3DE771613EE89573DF?sequence=1.

[28] World Health Organization. (2016). *WHO recommendations on antenatal care for a positive pregnancy experience.* Geneva, Switzerland. Retrieved from https://apps.who.int/iris/bitstream/handle/10665/250796/9789241549912-eng.pdf?sequence=1.

[29] Every Preemie SCALE, United States Agency for International Development, Project Concern International, Global Alliance to Prevent Prematurity and Stillbirth, & American College of Nurse-Midwives. (2019). *Management of Newborn Infections During Inpatient Care.* Do No Harm Technical Brief. Washington, DC: Every Preemie SCALE. Retrieved from https://www.everypreemie.org/wp-content/uploads/2019/07/DNH_TechBrief_MgmtNewbornInfection_7.15.19.pdf.

[30] Wagner, T. A., Gravett, C. A., Healy, S., Soma, V., Patterson, J. C., Gravett, M. G., & Rubens, C. E. (2011). Emerging biomarkers for the diagnosis of severe neonatal infections applicable to low resource settings. *Journal of Global Health, 1,* 210-223. Retrieved from http://www.jogh.org/documents/issue201102/JGH2-10_A5_Wagner.pdf.

[31] Saha, S. K., Schrag, S. J., El Arifeen, S., Mullany, L. C., Islam, M. S., Shang, N., … & Baqui, A. H. (2018). Causes and incidence of community-acquired serious infections among young children in south Asia (ANISA): an observational cohort study. *The Lancet, 392,* 145-159. https://doi.org/10.1016/S0140-6736(18)31127-9.

[32] World Health Organization. (2016). *Oxygen therapy for children.* Geneva, Switzerland. Retrieved from https://apps.who.int/iris/bitstream/handle/10665/204584/9789241549554_eng.pdf?sequence=1.

[33] Mulrooney, N., Champion, Z., Moss, T. J., Nitsos, I., Ikegami, M., & Jobe, A. H. (2005). Surfactant and physiologic responses of preterm lambs to continuous positive airway pressure. *American Journal of Respiratory and Critical Care Medicine, 171,* 488-493. https://doi.org/10.1164/rccm.200406-774OC.

[34] Subramaniam, P., Ho, J. J., & Davis, P. G. (2016). Prophylactic nasal continuous positive airway pressure for preventing morbidity and mortality in very preterm infants. *Cochrane Database of Systematic Reviews, 2016*(6). https://doi.org/10.1002/14651858.CD001243.pub3.

[35] Ho, J. J., Subramaniam, P., & Davis, P. G. (2015). Continuous distending pressure for respiratory distress in preterm infants. *Cochrane Database of Systematic Reviews, 2015*(7). https://doi.org/10.1002/14651858.CD002271.pub2.

[36] Sekar, K. C. & Corff, K. E. (2009). To tube or not to tube babies with respiratory distress syndrome. *Journal of Perinatology, 29,* S68-S72. https://doi.org/10.1038/jp.2009.28.

[37] Kawaza, K., Machen, H. E., Brown, J., Mwanza, Z., Iniguez, S., Gest, A., … & Molyneux, E. (2014). Efficacy of a low-cost bubble CPAP system in treatment of respiratory distress in a neonatal ward in Malawi. *PLoS One, 9,* 1-8. https://doi.org/10.1371/journal.pone.0086327.

[38] Koyamaibole, L., Kado, J., Qovu, J. D., Colquhoun, S., & Duke, T. (2006). An evaluation of bubble-CPAP in a neonatal unit in a developing country: Effective respiratory support that can be applied by nurses. *Journal of Tropical Pediatrics, 52,* 249-253. https://doi.org/10.1093/tropej/fmi109.

[39] World Health Organization. (2015). *WHO recommendations on interventions to improve preterm birth outcomes.* Geneva, Switzerland. Retrieved from https://apps.who.int/iris/bitstream/handle/10665/183037/9789241508988_eng.pdf?sequence=1.

[40] Every Preemie SCALE, United States Agency for International Development, Project Concern International, Global Alliance to Prevent Prematurity and Stillbirth, & American College of Nurse-Midwives. (2017). *Safe and Effective Oxygen Use for Inpatient Care of Newborns.* Do No Harm Technical Brief. Washington, DC: Every Preemie SCALE. Retrieved from https://www.everypreemie.org/wp-content/uploads/2019/09/SafeOxygen_english_7.6.17.pdf.

[41] The Institute of Electrical and Electronics Engineers. (2006). *IEEE Recommended Practice for Powering and Grounding Electronic Equipment* (IEEE Std 1100-2005, Revision of IEEE Std 1100-1999). https://doi.org/10.1109/IEEESTD.2006.216391.

[42] Gilbert, C., Malik, A. N. J., Nahar, N., Das, S. K., Visser, L., Sitati, S., Ademola-Popoola, D. S. (2019). Epidemiology of ROP update - Africa is the new frontier. *Seminars in Perinatology, 43,* 317-322. https://doi.org/10.1053/j.semperi.2019.05.002.

[43] Blencowe, H., Lawn, J. E., Vazquez, T., Fielder, A., & Gilbert, C. (2013). Preterm-associated visual impairment and estimates of retinopathy of prematurity at regional and global levels for 2010. *Pediatric Research*, *74,* 35-49. https://doi.org/10.1038/pr.2013.205.

[44] Benaron, D. A. & Benitz, W. E. (1994). Maximizing the stability of oxygen delivered via nasal cannula. *Archives of Pediatrics and Adolescent Medicine, 148,* 294-300. https://doi.org/10.1001/archpedi.1994.02170030064015.

[45] PATH. (2015). *Design for reliability: Ideal product requirement specifications for oxygen concentrators for children with hypoxemia in lowresource settings*. Seattle, WA. Retrieved from https://path.azureedge.net/media/documents/DT_oxygen_concentrators_ideal_design.pdf.

[46] International Electrotechnical Commission. (2012). *Medical electrical equipment – Part 1-8: General requirements for basic safety and essential performance – Collateral Standard: General requirements, tests and guidance for alarm systems in medical electrical equipment and medical electrical systems* (IEC 60601-1-8: 2012). Retrieved from https://global.ihs.com/doc_detail.cfm?document_name=IEC%2060601%2D1%2D8&item_s_key=00415558&csf=ASA#referenced-documents.

[47] World Health Organization. (2015). *Technical specifications for oxygen concentrators.* WHO medical device technical series. Geneva, Switzerland. Retrieved from https://apps.who.int/iris/bitstream/handle/10665/199326/9789241509886_eng.pdf?sequence=1.

[48] Peel, D., Neighbour, R., & Eltringham, R. J. (2013). Evaluation of oxygen concentrators for use in countries with limited resources. *Anaesthesia, 68,* 706-712. https://doi.org/10.1111/anae.12260.

[49] The International Organization for Standardization & the Institute of Electrical and Electronics Engineers. (2019). *Health informatics – Personal health device communication – Part 10404: Device specialization – Pulse oximeter.* (ISO/IEEE Std 11073-10404). Retrieved from https://ieeexplore.ieee.org/servlet/opac?punumber=8725993.

[50] World Health Organization. (2016). *Decontamination and reprocessing of medical devices for health-care facilities.* Geneva, Switzerland. Retrieved from https://apps.who.int/iris/bitstream/handle/10665/250232/9789241549851-eng.pdf?sequence=1.

[51] Zhao, J., Gonzalez, F., & Mu, D. (2011). Apnea of prematurity: From cause to treatment. *European Journal of Pediatrics, 170*(9), 1097-1105. https://doi.org/10.1007/s00431-011-1409-6.

[52] Lunze, K., Bloom, D. E., Jamison, D. T., & Hamer, D. H. (2013). The global burden of neonatal hypothermia: systematic review of a major challenge for newborn survival. *BMC Medicine, 11,* 24. https://doi.org/10.1186/1741-7015-11-24.

[53] Perlman, J. M., Wiley, J., Kattwinkel, J., Wyckoff, M. H., Aziz, K., Guinsburg, R., … & Velaphi, S. (2015). Part 7: Neonatal resuscitation: 2015 international consensus on cardiopulmonary resuscitation and emergency cardiovascular care science with treatment recommendations. *Circulation, 132*(suppl 1), S204-S241. https://doi.org/10.1161/CIR.0000000000000276.

[54] Ibe, O. E., Austin, T., Sullivan, K., Fabanwo, O., Disu, E., & Costello, A. M. (2004). A comparison of kangaroo mother care and conventional incubator care for thermal regulation of infants <2000 g in Nigeria using continuous ambulatory temperature monitoring. *Annals of Tropical Paediatrics, 24,* 245–251. https://doi.org/10.1179/027249304225019082.

[55] Bergman, N. J., Linley, L. L., & Fawcus, S. R. (2004). Randomized controlled trial of skin-to-skin contact from birth versus conventional incubator for physiological stabilization in 1200- to 2199-gram newborns. *Acta Paediatrica, 93,* 779–785. https://doi.org/10.1111/j.1651-2227.2004.tb03018.x.

[56] Amadi, H. O., Olateju, E. K., Alabi, P., Kawuwa, M. B., Ibadin, M. O., & Osibogun, A. O. (2015). Neonatal hyperthermia and thermal stress in low- and middle-income countries: a hidden cause of death in extremely low-birthweight neonates. *Paediatrics and International Child Health, 35,* 273–281. https://doi.org/10.1179/2046905515Y.0000000030.

[57] Abdel-Hady, H., Hawas, S., El-Daker, M., & El-Kady, R. (2008). Extended-spectrum β-lactamase producing Klebsiella pneumoniae in neonatal intensive care unit. *Journal of Perinatology, 28,* 685–690. https://doi.org/10.1038/jp.2008.73.

[58] Iregbu, K. C. & Anwaal, U. (2007). Extended spectrum Beta-Lactamase-producing Klebsiella pneumoniae septicaemia outbreak in the Neonatal Intensive Care Unit of a tertiary hospital in Nigeria. *African Journal of Medicine and Medical Sciences, 36,* 225-228. Retrieved from https://www.ncbi.nlm.nih.gov/pubmed/18390061.

[59] Amadi, H. O. (2012). Neonatal Thermoneutrality in a Tropical Climate (chapter 28). In: Rodriguez-Morales, A. (Ed.), *Current Topics in Tropical Medicine*. Minneapolis, MN: IntechOpen. https://doi.org/10.5772/26063.

[60] Amadi, H. O., Mohammed, L. I., Kawuwa, M. B., Oyedokun, A., & Mohammed, H. (2014). Synthesis and validation of a weatherproof nursery design that eliminates tropical evening-Fever syndrome in neonates. *International Journal of Pediatrics, 2014*, 986760. https://doi.org/10.1155/2014/986760.

[61] Christensson, K., Bhat, G. J., Eriksson, B., Shilalukey-Ngoma, M. P., Sterky, G. (1995). The effect of routine hospital care on the health of hypothermic newborn infants in Zambia. *Journal of Tropical Pediatrics, 41,* 210–214. https://doi.org/10.1093/tropej/41.4.210.

[62] Amadi, H. O., Mokuolu, O. A., Adimora, G. N., Pam, S. D., Etawo, U. S., Ohadugha, C. O., & Adesiyun, O. O. (2007). Digitally recycled incubators: better economic alternatives to modern systems in low-income countries. *Annals of Tropical Paediatrics, 27,* 207–214. https://doi.org/10.1179/146532807X220325.

[63] Bhat, S. R., Meng, N. F., Kumar, K., Nagesh, K. N., Kawale, A., Bhutani, V. K. (2015). Keeping babies warm: a non-inferiority trial of a conductive thermal mattress. *Archives of Disease in Childhood Fetal and Neonatal Edition, 100,* F309–F312. https://doi.org/10.1136/archdischild-2014-306269.

[64] Howitt, P., Darzi, A., Yang, G. Z., Ashrafian, H., Atun, R., Barlow, J., …& Wilson, E. (2012). Technologies for global health. *The Lancet, 380,* 507–535. https://doi.org/10.1016/S0140-6736(12)61127-1.

[65] Olson, K. & Caldwell, A. (2010). Designing an early stage prototype using readily available material for a neonatal incubator for poor settings. In: *2010 Annual International Conference of the IEEE Engineering in Medicine and Biology Society,* 1100–1103. https://doi.org/10.1109/IEMBS.2010.5627347.

[66] Adair-Rohani, H., Zukor, K., Bonjour, S., Wilburn, S., Kuesel, A. C., Hebert, R., & Fletcher, E. R. (2013). Limited electricity access in health facilities of sub-Saharan Africa: a systematic review of data on electricity access, sources, and reliability. *Global Health: Science & Practice, 1*, 249-261. https://doi.org/10.9745/GHSP-D-13-00037.

[67] Otiangala, D., Agai, N. O., Olayo, B., Adudans, S., Ng, C. H., Calderon, R., … & Hawkes, M. (2020). Oxygen insecurity and mortality in resource-constrained healthcare facilities in rural Kenya. *Pediatric Pulmonology, 55,* 1043-1049. https://doi.org/10.1002/ppul.24679.

[68] Beverly Bradley, Yu-Ling Cheng, David Peel, Shauna Mullally, and Stephen Howie. 2011. Assessment of Power Availability and Development of a Low-Cost Battery-Powered Medical Oxygen Delivery System: For Use in Low-Resource Health Facilities in Developing Countries. In Proceedings of the 2011 IEEE Global Humanitarian Technology Conference (GHTC '11). *IEEE Computer Society*, USA, 148–153. https://doi.org/10.1109/GHTC.2011.25.

[69] World Health Organization. (2016). *WHO technical specifications of neonatal resuscitation devices.* Geneva, Switzerland. Retrieved from https://apps.who.int/medicinedocs/documents/s22389en/s22389en.pdf/.

[70] International Electrotechnical Commission. (2000). *Medical electrical equipment - Part 2-50: Particular requirements for the basic safety and essential performance of infant phototherapy equipment.* (IEC 60601-2-50). Retrieved from https://webstore.iec.ch/publication/2669.

[71] American Academy of Pediatrics Subcommittee on Hyperbilirubinemia. (2004). Management of hyperbilirubinemia in the newborn infant 35 or more weeks of gestation. *Pediatrics, 114*, 297-316. https://doi.org/10.1542/peds.114.1.297.

[72] The International Organization for Standardization. (2014). *Medical electrical equipment – Part 2-69: Particular requirements for basic safety and essential performance of oxygen concentrator equipment* (ISO 80601-2-69). Retrieved from https://www.iso.org/standard/59978.html.

[73] Council of Europe. (2011). Oxygen (93 percent). *European Pharmacopoeia, 7.1*(04/2011:2455), 3445-3447. Retrieved from http://www2.sol.it/AreaClienti/solconsulting/consulting/farmacopea/dwn/oss93.pdf.

[74] World Health Organization Maternal & Newborn Health/Safe Motherhood Unit. (1997). *Thermal protection of the newborn: A practical guide.* Geneva, Switzerland: World Health Organization. Retrieved from https://apps.who.int/iris/bitstream/handle/10665/63986/WHO_RHT_MSM_97.2.pdf;jsessionid=346E0D06554B03D0CA90BB771599155C?sequence=1.

[75] International Electrotechnical Commission. (2009). Accuracy of the control of the contact surface temperature (section 201.12.4.104). *Medical electrical equipment — Part 2-35: Particular requirements for the basic safety and essential performance of heating devices using blankets, pads or mattresses and intended for heating in medical use.* (IEC 80601-2-35). Retrieved from https://www.iso.org/standard/51975.html.

[76] The International Organization for Standardization. (2017). Time response for a continuous clinical thermometer (section 201.101.3). *Medical electrical equipment — Part 2-56: Particular requirements for basic safety and essential performance of clinical thermometers for body temperature measurement.* (ISO 80601-2-56). Retrieved from https://www.iso.org/standard/67348.html.

[77] International Electrotechnical Commission. (2009). Requirements for heating devices other than forced air devices; Maximum contact surface temperature in normal condition (section 201.11.1.2.1.101.1). *Medical electrical equipment — Part 2-35: Particular requirements for the basic safety and essential performance of heating devices using blankets, pads or mattresses and intended for heating in medical use.* (IEC 80601-2-35). Retrieved from https://www.iso.org/standard/51975.html.

[78] International Electrotechnical Commission. (2009). Temperature overshoot when the temperature control is set to its maximum setting (section 201.12.4.103). *Medical electrical equipment — Part 2-35: Particular requirements for the basic safety and essential performance of*

heating devices using blankets, pads or mattresses and intended for heating in medical use. (IEC 80601-2-35). Retrieved from https://www.iso.org/standard/51975.html.

[79] International Electrotechnical Commission. (2009). Uniformity of incubator temperature (section 201.12.1.102). *Medical electrical equipment - Part 2-19: Particular requirements for the basic safety and essential performance of infant incubators.* (IEC 60601-2-19). Retrieved from http://oedk.rice.edu/Resources/Documents/Standards/iec60601-2-19%7Bed2.0%7Db.pdf.

[80] The International Organization for Standardization. (2016). *Medical devices — Quality management systems — Requirements for regulatory purposes* (ISO 13485). Retrieved from https://www.iso.org/standard/59752.html.

[81] The International Organization for Standardization. (2017). Time response for a continuous clinical thermometer (section 201.101.2). *Medical electrical equipment — Part 2-56: Particular requirements for basic safety and essential performance of clinical thermometers for body temperature measurement.* (ISO 80601-2-56). Retrieved from https://www.iso.org/standard/67348.html.
