# Supplementary material for: Target product profiles for neonatal care devices: systematic development and outcomes with NEST360 and UNICEF
Source: BMC Pediatr. 2023 Nov 15;23(Suppl 2):564. doi: 10.1186/s12887-023-04342-1 (PMC10647088; doi:10.1186/s12887-023-04342-1)
Supplement: Supplementary file 4 — Additional file 4. Target Product Profile Qualtrics survey. Qualtrics survey for all of the TPPs. [file 12887_2023_4342_MOESM4_ESM.pdf]

NEST 360 TPPs

Start of Block: Default Question Block

Q1.1 What country are you from?

____

Q1.2 Please tell us your name (not obligatory)

____

Q1.3 Please describe yourself/organization

- Advocacy Organization (1)
- Technical Agency / Researcher (2)
- Funder (3)
- Implementer / Clinician (4)
- Ministry of Health (5)
- International Body (6)
- Industry (7)
- Other (8)

Page Break

Q1.4 Please select the newborn technology (ies) that you would like to provide feedback on. (Please Select Up to 3. You can select more if you are so inclined.)

- ☐ Bubble CPAP (1)
- ☐ Pulse Oximeter (2)
- ☐ Oxygen Concentrator (3)
- ☐ Flow Splitter (4)
- ☐ Suction Pump (5)
- ☐ Respiratory Rate Monitors (6)
- ☐ Serum Bilirubin Test (7)
- ☐ Phototherapy Light (8)
- ☐ Warming Crib (9)
- ☐ Radiant Warmer (10)
- ☐ Temperature Monitor (11)
- ☐ Syringe Pump (12)
- ☐ Sepsis Test (13)
- ☐ Hemoglobin Test (14)
- ☐ Glucose Test (15)
- ☐ pH Test (19)
- ☐ Other (20)

Q1.5 Describe your interaction or efforts with the newborn technology (ies) that you selected above.

____

____

____

____

____

End of Block: Default Question Block

Start of Block: CPAP

Display This Question:

If Please select the newborn technology (ies) that you would like to provide feedback on. (Please Se... = Bubble CPAP

Q2.1 Bubble CPAP

Display This Question:

If Please select the newborn technology (ies) that you would like to provide feedback on. (Please Se... = Bubble CPAP

Q2.2 1. Intended Use: Please rate your level of agreement with the statements under optimal and minimal. *Note: The optimal and minimal requirements define a range.*

	1-Disagree (1)	2-Somewhat Disagree (2)	3-Neither Agree nor Disagree (3)	4-Mostly Agree (4)	5-Fully agree (5)	Other - Do not have the expertise to comment (6)
**Optimal:** To treat respiratory distress and other forms of respiratory illness in infants up to one year of age. (1)	○	○	○	○	○	○
**Minimal:** Same as Optimal. (2)	○	○	○	○	○	○

Display This Question:

If Please select the newborn technology (ies) that you would like to provide feedback on. (Please Se... = Bubble CPAP

Q2.3 2. Intended Use: Please provide reasoning if you have chosen 3 or below.

____

____

____

____

____

Display This Question:

If Please select the newborn technology (ies) that you would like to provide feedback on. (Please Se... = Bubble CPAP

Q2.4 3. Target Operator: Please rate your level of agreement with the statements under optimal and minimal. *Note: The optimal and minimal requirements define a range.*

	1-Disagree (1)	2-Somewhat Disagree (2)	3-Neither agree nor disagree (3)	4-Mostly Agree (4)	5-Fully Agree (5)	Other - Do not have the expertise to comment (6)
**Optimal:** For use in low- and middle-income countries by a wide variety of clinicians, including nurses, clinical officers, and pediatricians. (1)	○	○	○	○	○	○
**Minimal:** Same as Optimal (2)	○	○	○	○	○	○

Display This Question:

If Please select the newborn technology (ies) that you would like to provide feedback on. (Please Se... = Bubble CPAP

Q2.5 4. Target Operator: Please provide reasoning if you have chosen 3 or below.

____

Display This Question:

If Please select the newborn technology (ies) that you would like to provide feedback on. (Please Se... = Bubble CPAP

Q2.6 5. Target Population: Please rate your level of agreement with the statements under optimal and minimal. *Note: The optimal and minimal requirements define a range.*

	1-Disagree (1)	2-Somewhat Disagree (2)	3-Neither Agree nor Disagree (3)	4-Mostly Agree (4)	5-Fully agree (5)	Other - Do not have the expertise to comment (6)
**Optimal:** Neonates ((1)	o	o	o	o	o	o
**Minimal:** Same as Optimal. (2)	o	o	o	o	o	o

Display This Question:

If Please select the newborn technology (ies) that you would like to provide feedback on. (Please Se... = Bubble CPAP

Q2.7 6. Target Population: Please provide reasoning if you have chosen 3 or below.

____

Display This Question:

If Please select the newborn technology (ies) that you would like to provide feedback on. (Please Se... = Bubble CPAP

Q2.8 7. Target Setting: Please rate your level of agreement with the statements under optimal and minimal. *Note: The optimal and minimal requirements define a range.*

	1-Disagree (1)	2-Somewhat Disagree (2)	3-Neither Agree nor Disagree (3)	4-Mostly Agree (4)	5-Fully agree (5)	Other - Do not have the expertise to comment (6)
**Optimal:** Hospitals in low-resource settings (1)	○	○	○	○	○	○
**Minimal:** Same as Optimal. (2)	○	○	○	○	○	○

Display This Question:

If Please select the newborn technology (ies) that you would like to provide feedback on. (Please Se... = Bubble CPAP

Q2.9 8. Target Setting: Please provide reasoning if you have chosen 3 or below.

____

____

____

____

____

Display This Question:

If Please select the newborn technology (ies) that you would like to provide feedback on. (Please Se... = Bubble CPAP

Q2.10 9. International Standard: Please rate your level of agreement with the statements under optimal and minimal. *Note: The optimal and minimal requirements define a range.*

	1-Disagree (1)	2-Somewhat Disagree (2)	3-Neither Agree nor Disagree (3)	4-Mostly Agree (4)	5-Fully agree (5)	Other - Do not have the expertise to comment (6)
**Optimal:** ISO 13485:2016 Medical devices – Quality management systems -- Requirements for regulatory purposes. (1)	○	○	○	○	○	○
**Minimal:** Same as Optimal. (2)	○	○	○	○	○	○

Display This Question:

If Please select the newborn technology (ies) that you would like to provide feedback on. (Please Se... = Bubble CPAP

Q2.11 10. International Standard: Please provide reasoning if you have chosen 3 or below.

____

____

____

____

____

Display This Question:

If Please select the newborn technology (ies) that you would like to provide feedback on. (Please Se... = Bubble CPAP

Q2.12 11. Regulation: Please rate your level of agreement with the statements under optimal and minimal. *Note: The optimal and minimal requirements define a range.*

	1-Disagree (1)	2-Somewhat Disagree (2)	3-Neither Agree nor Disagree (3)	4-Mostly Agree (4)	5-Fully agree (5)	Other - Do not have the expertise to comment (6)
**Optimal:** CE marking or US FDA Clearance (1)	○	○	○	○	○	○
**Minimal:** Same as Optimal. (2)	○	○	○	○	○	○

Display This Question:

If Please select the newborn technology (ies) that you would like to provide feedback on. (Please Se... = Bubble CPAP

Q2.13 12. Regulation: Please provide reasoning if you have chosen 3 or below.

____

____

____

____

____

Display This Question:

If Please select the newborn technology (ies) that you would like to provide feedback on. (Please Se... = Bubble CPAP

Q2.14 13. Flow Driver: Please rate your level of agreement with the statements under optimal and minimal. *Note: The optimal and minimal requirements define a range.*

	1-Disagree (1)	2-Somewhat Disagree (2)	3-Neither Agree nor Disagree (3)	4-Mostly Agree (4)	5-Fully agree (5)	Other - Do not have the expertise to comment (6)
**Optimal:** Integrated (on-board air compressor) (1)	○	○	○	○	○	○
**Minimal:** Same as Optimal. (2)	○	○	○	○	○	○

Display This Question:

If Please select the newborn technology (ies) that you would like to provide feedback on. (Please Se... = Bubble CPAP

Q2.15 14. Flow Driver: Please provide reasoning if you have chosen 3 or below.

____

____

____

____

____

Display This Question:

If Please select the newborn technology (ies) that you would like to provide feedback on. (Please Se... = Bubble CPAP

Q2.16 15. Oxygen Flow Capability: Please rate your level of agreement with the statements under optimal and minimal. *Note: The optimal and minimal requirements define a range.*

	1-Disagree (1)	2-Somewhat Disagree (2)	3-Neither Agree nor Disagree (3)	4-Mostly Agree (4)	5-Fully agree (5)	Other - Do not have the expertise to comment (6)
**Optimal:** 0-10 L/min (1)	○	○	○	○	○	○
**Minimal:** Same as Optimal. (2)	○	○	○	○	○	○

Display This Question:

If Please select the newborn technology (ies) that you would like to provide feedback on. (Please Se... = Bubble CPAP

Q2.17 16. Oxygen Flow Capability: Please provide reasoning if you have chosen 3 or below.

____

____

____

____

____

Display This Question:

If Please select the newborn technology (ies) that you would like to provide feedback on. (Please Se... = Bubble CPAP

Q2.18 17. Pressure: Please rate your level of agreement with the statements under optimal and minimal. *Note: The optimal and minimal requirements define a range.*

	1-Disagree (1)	2-Somewhat Disagree (2)	3-Neither Agree nor Disagree (3)	4-Mostly Agree (4)	5-Fully agree (5)	Other - Do not have the expertise to comment (6)
**Optimal:** 5-8 cm H20 (1)	○	○	○	○	○	○
**Minimal:** Same as Optimal. (2)	○	○	○	○	○	○

Display This Question:

If Please select the newborn technology (ies) that you would like to provide feedback on. (Please Se... = Bubble CPAP

Q2.19 18. Pressure: Please provide reasoning if you have chosen 3 or below.

____

____

____

____

____

Display This Question:

If Please select the newborn technology (ies) that you would like to provide feedback on. (Please Se... = Bubble CPAP

Q2.20 19. Total (blended) Flow: Please rate your level of agreement with the statements under optimal and minimal. *Note: The optimal and minimal requirements define a range.*

	1-Disagree (1)	2-Somewhat Disagree (2)	3-Neither Agree nor Disagree (3)	4-Mostly Agree (4)	5-Fully agree (5)	Other - Do not have the expertise to comment (6)
**Optimal:** 0-10 L/min (1)	o	o	o	o	o	o
**Minimal:** Same as Optimal. (2)	o	o	o	o	o	o

Display This Question:

If Please select the newborn technology (ies) that you would like to provide feedback on. (Please Se... = Bubble CPAP

Q2.21 20. Total (blended) Flow: Please provide reasoning if you have chosen 3 or below.

____

____

____

____

____

Display This Question:

If Please select the newborn technology (ies) that you would like to provide feedback on. (Please Se... = Bubble CPAP

Q2.22 21. Humidification: Please rate your level of agreement with the statements under optimal and minimal. *Note: The optimal and minimal requirements define a range.*

	1-Disagree (1)	2-Somewhat Disagree (2)	3-Neither Agree nor Disagree (3)	4-Mostly Agree (4)	5-Fully agree (5)	Other - Do not have the expertise to comment (6)
**Optimal:** Yes, Heated Humidification (1)	o	o	o	o	o	o
**Minimal:** None (2)	o	o	o	o	o	o

Display This Question:

If Please select the newborn technology (ies) that you would like to provide feedback on. (Please Se... = Bubble CPAP

Q2.23 22. Humidification: Please provide reasoning if you have chosen 3 or below.

____

____

____

____

____

Display This Question:

If Please select the newborn technology (ies) that you would like to provide feedback on. (Please Se... = Bubble CPAP

Q2.24 23. Alarms: Please rate your level of agreement with the statements under optimal and minimal. *Note: The optimal and minimal requirements define a range.*

	1-Disagree (1)	2-Somewhat Disagree (2)	3-Neither Agree nor Disagree (3)	4-Mostly Agree (4)	5-Fully agree (5)	Other - Do not have the expertise to comment (6)
**Optimal:** Audio/Visual Power, low-flow, low-pressure (1)	o	o	o	o	o	o
**Minimal:** Audio Power (2)	o	o	o	o	o	o

Display This Question:

If Please select the newborn technology (ies) that you would like to provide feedback on. (Please Se... = Bubble CPAP

Q2.25 24. Alarms: Please provide reasoning if you have chosen 3 or below.

____

____

____

____

____

Display This Question:

If Please select the newborn technology (ies) that you would like to provide feedback on. (Please Se... = Bubble CPAP

Q2.26 25. Consumables: Please rate your level of agreement with the statements under optimal and minimal. *Note: The optimal and minimal requirements define a range.*

	1-Disagree (1)	2-Somewhat Disagree (2)	3-Neither Agree nor Disagree (3)	4-Mostly Agree (4)	5-Fully agree (5)	Other - Do not have the expertise to comment (6)
**Optimal:** Reusable (1)	o	o	o	o	o	o
**Minimal:** Available (2)	o	o	o	o	o	o

Display This Question:

If Please select the newborn technology (ies) that you would like to provide feedback on. (Please Se... = Bubble CPAP

Q2.27 26. Consumables: Please provide reasoning if you have chosen 3 or below.

____

____

____

____

____

Display This Question:

If Please select the newborn technology (ies) that you would like to provide feedback on. (Please Se... = Bubble CPAP

Q2.28 25. Accessories: Please rate your level of agreement with the statements under optimal and minimal. *Note: The optimal and minimal requirements define a range.*

	1-Disagree (1)	2-Somewhat Disagree (2)	3-Neither Agree nor Disagree (3)	4-Mostly Agree (4)	5-Fully agree (5)	Other - Do not have the expertise to comment (6)
**Optimal:** Non-proprietary (1)	○	○	○	○	○	○
**Minimal:** Proprietary (2)	○	○	○	○	○	○

Display This Question:

If Please select the newborn technology (ies) that you would like to provide feedback on. (Please Se... = Bubble CPAP

Q2.29 27. Accessories: Please provide reasoning if you have chosen 3 or below.

____

____

____

____

____

Display This Question:

If Please select the newborn technology (ies) that you would like to provide feedback on. (Please Se... = Bubble CPAP

Q2.30 28. Back-up Battery: Please rate your level of agreement with the statements under optimal and minimal. *Note: The optimal and minimal requirements define a range.*

	1-Disagree (1)	2-Somewhat Disagree (2)	3-Neither Agree nor Disagree (3)	4-Mostly Agree (4)	5-Fully agree (5)	Other - Do not have the expertise to comment (6)
**Optimal:** Built-in rechargeable battery, autonomy >1 hour, automatic switch to battery in case of power failure, automatic recharge on connection to mains (only applicable to the electric CPAP generator model) (1)	○	○	○	○	○	○
**Minimal:** None (2)	○	○	○	○	○	○

Display This Question:

If Please select the newborn technology (ies) that you would like to provide feedback on. (Please Se... = Bubble CPAP

Q2.31 29. Back-up Battery: Please provide reasoning if you have chosen 3 or below.

____

____

____

____

____

Display This Question:

If Please select the newborn technology (ies) that you would like to provide feedback on. (Please Se... = Bubble CPAP

Q2.32 30. Voltage: Please rate your level of agreement with the statements under optimal and minimal. *Note: The optimal and minimal requirements define a range.*

	1-Disagree (1)	2-Somewhat Disagree (2)	3-Neither Agree nor Disagree (3)	4-Mostly Agree (4)	5-Fully agree (5)	Other - Do not have the expertise to comment (6)
**Optimal:** 110-240V 50-60hz (1)	o	o	o	o	o	o
**Minimal:** 220-240V 50-60hz (2)	o	o	o	o	o	o

Display This Question:

If Please select the newborn technology (ies) that you would like to provide feedback on. (Please Se... = Bubble CPAP

Q2.33 31. Voltage: Please provide reasoning if you have chosen 3 or below.

____

____

____

____

____

Display This Question:

If Please select the newborn technology (ies) that you would like to provide feedback on. (Please Se... = Bubble CPAP

Q2.34 32. User Manual: Please rate your level of agreement with the statements under optimal and minimal. *Note: The optimal and minimal requirements define a range.*

	1-Disagree (1)	2-Somewhat Disagree (2)	3-Neither Agree nor Disagree (3)	4-Mostly Agree (4)	5-Fully agree (5)	Other - Do not have the expertise to comment (6)
**Optimal:** User manual and additional training materials (checklists, videos, guides) in English and local language. Attached to device with labels and markings where possible. (1)	o	o	o	o	o	o
**Minimal:** User manual provided. (2)	o	o	o	o	o	o

Display This Question:

If Please select the newborn technology (ies) that you would like to provide feedback on. (Please Se... = Bubble CPAP

Q2.35 33. User Manual: Please provide reasoning if you have chosen 3 or below.

____

____

____

____

____

Display This Question:

If Please select the newborn technology (ies) that you would like to provide feedback on. (Please Se... = Bubble CPAP

Q2.36 32. Warranty: Please rate your level of agreement with the statements under optimal and minimal. *Note: The optimal and minimal requirements define a range.*

	1-Disagree (1)	2-Somewhat Disagree (2)	3-Neither Agree nor Disagree (3)	4-Mostly Agree (4)	5-Fully agree (5)	Other - Do not have the expertise to comment (6)
**Optimal:** 5 years (1)	○	○	○	○	○	○
**Minimal:** 1 year (2)	○	○	○	○	○	○

Display This Question:

If Please select the newborn technology (ies) that you would like to provide feedback on. (Please Se... = Bubble CPAP

Q2.37 35. Warranty: Please provide reasoning if you have chosen 3 or below.

____

____

____

____

____

Display This Question:

If Please select the newborn technology (ies) that you would like to provide feedback on. (Please Se... = Bubble CPAP

Q2.38 36. Instrument Pricing: Please rate your level of agreement with the statements under optimal and minimal. *Note: The optimal and minimal requirements define a range.*

	1-Disagree (1)	2-Somewhat Disagree (2)	3-Neither Agree nor Disagree (3)	4-Mostly Agree (4)	5-Fully agree (5)	Other - Do not have the expertise to comment (6)
**Optimal:** (1)	o	o	o	o	o	o
**Minimal:** (2)	o	o	o	o	o	o

Display This Question:

If Please select the newborn technology (ies) that you would like to provide feedback on. (Please Se... = Bubble CPAP

Q2.39 37. Instrument Pricing: Please provide reasoning if you have chosen 3 or below.

____

____

____

____

____

Display This Question:

If Please select the newborn technology (ies) that you would like to provide feedback on. (Please Se... = Bubble CPAP

Q2.40 38. Consumable Pricing: Please rate your level of agreement with the statements under optimal and minimal. *Note: The optimal and minimal requirements define a range.*

	1-Disagree (1)	2-Somewhat Disagree (2)	3-Neither Agree nor Disagree (3)	4-Mostly Agree (4)	5-Fully agree (5)	Other - Do not have the expertise to comment (6)
**Optimal:** (1)	○	○	○	○	○	○
**Minimal:** (2)	○	○	○	○	○	○

Display This Question:

If Please select the newborn technology (ies) that you would like to provide feedback on. (Please Se... = Bubble CPAP

Q2.41 39. Consumable Pricing: Please provide reasoning if you have chosen 3 or below.

____

____

____

____

____

End of Block: CPAP

Start of Block: Pulse Oximeter

Display This Question:

If Please select the newborn technology (ies) that you would like to provide feedback on. (Please Se... = Pulse Oximeter

Q3.1 Pulse Oximeter

Display This Question:

If Please select the newborn technology (ies) that you would like to provide feedback on. (Please Se... = Pulse Oximeter

Q3.2 1. Intended Use: Please rate your level of agreement with the statements under optimal and minimal. *Note: The optimal and minimal requirements define a range.*

	1-Disagree (1)	2-Somewhat Disagree (2)	3-Neither Agree nor Disagree (3)	4-Mostly Agree (4)	5-Fully agree (5)	Other - Do not have the expertise to comment (6)
**Optimal:** To continuously monitor oxygen saturation (Sp02) and pulse rate (PR) for neonatal patients. (1)	o	o	o	o	o	o
**Minimal:** Same as Optimal. (2)	o	o	o	o	o	o

Display This Question:

If Please select the newborn technology (ies) that you would like to provide feedback on. (Please Se... = Pulse Oximeter

Q3.3 2. Intended Use: Please provide reasoning if you have chosen 3 or below.

____

____

____

____

____

Display This Question:

If Please select the newborn technology (ies) that you would like to provide feedback on. (Please Se... = Pulse Oximeter

Q3.4 3. Target Operator: Please rate your level of agreement with the statements under optimal and minimal. *Note: The optimal and minimal requirements define a range.*

	1-Disagree (1)	2-Somewhat Disagree (2)	3-Neither agree nor disagree (3)	4-Mostly Agree (4)	5-Fully Agree (5)	Other - Do not have the expertise to comment (6)
**Optimal:** For use in low- and middle-income countries by a wide variety of clinicians, including nurses, clinical officers, and pediatricians. (1)	○	○	○	○	○	○
**Minimal:** Same as Optimal (2)	○	○	○	○	○	○

Display This Question:

If Please select the newborn technology (ies) that you would like to provide feedback on. (Please Se... = Pulse Oximeter

Q3.5 4. Target Operator: Please provide reasoning if you have chosen 3 or below.

____

Display This Question:

If Please select the newborn technology (ies) that you would like to provide feedback on. (Please Se... = Pulse Oximeter

Q3.6 5. Target Population: Please rate your level of agreement with the statements under optimal and minimal. *Note: The optimal and minimal requirements define a range.*

	1-Disagree (1)	2-Somewhat Disagree (2)	3-Neither Agree nor Disagree (3)	4-Mostly Agree (4)	5-Fully agree (5)	Other - Do not have the expertise to comment (6)
**Optimal:** Neonates ((1)	○	○	○	○	○	○
**Minimal:** Same as Optimal. (2)	○	○	○	○	○	○

Display This Question:

If Please select the newborn technology (ies) that you would like to provide feedback on. (Please Se... = Pulse Oximeter

Q3.7 6. Target Population: Please provide reasoning if you have chosen 3 or below.

____

Display This Question:

If Please select the newborn technology (ies) that you would like to provide feedback on. (Please Se... = Pulse Oximeter

Q3.8 7. Target Setting: Please rate your level of agreement with the statements under optimal and minimal. *Note: The optimal and minimal requirements define a range.*

	1-Disagree (1)	2-Somewhat Disagree (2)	3-Neither Agree nor Disagree (3)	4-Mostly Agree (4)	5-Fully agree (5)	Other - Do not have the expertise to comment (6)
**Optimal:** Hospitals in low-resource settings (1)	o	o	o	o	o	o
**Minimal:** Same as Optimal. (2)	o	o	o	o	o	o

Display This Question:

If Please select the newborn technology (ies) that you would like to provide feedback on. (Please Se... = Pulse Oximeter

Q3.9 8. Target Setting: Please provide reasoning if you have chosen 3 or below.

____

____

____

____

____

Display This Question:

If Please select the newborn technology (ies) that you would like to provide feedback on. (Please Se... = Pulse Oximeter

Q3.10 9. International Standard: Please rate your level of agreement with the statements under optimal and minimal. *Note: The optimal and minimal requirements define a range.*

	1-Disagree (1)	2-Somewhat Disagree (2)	3-Neither Agree nor Disagree (3)	4-Mostly Agree (4)	5-Fully agree (5)	Other - Do not have the expertise to comment (6)
**Optimal:** ISO 13485:2016 Medical devices – Quality management systems -- Requirements for regulatory purposes. (1)	o	o	o	o	o	o
**Minimal:** Same as Optimal. (2)	o	o	o	o	o	o

Display This Question:

If Please select the newborn technology (ies) that you would like to provide feedback on. (Please Se... = Pulse Oximeter

Q3.11 10. International Standard: Please provide reasoning if you have chosen 3 or below.

____

____

____

____

____

Display This Question:

If Please select the newborn technology (ies) that you would like to provide feedback on. (Please Se... = Pulse Oximeter

Q3.12 11. Regulation: Please rate your level of agreement with the statements under optimal and minimal. *Note: The optimal and minimal requirements define a range.*

	1-Disagree (1)	2-Somewhat Disagree (2)	3-Neither Agree nor Disagree (3)	4-Mostly Agree (4)	5-Fully agree (5)	Other - Do not have the expertise to comment (6)
**Optimal:** CE marking or US FDA Clearance (1)	○	○	○	○	○	○
**Minimal:** Same as Optimal. (2)	○	○	○	○	○	○

Display This Question:

If Please select the newborn technology (ies) that you would like to provide feedback on. (Please Se... = Pulse Oximeter

Q3.13 12. Regulation: Please provide reasoning if you have chosen 3 or below.

____

____

____

____

____

Display This Question:

If Please select the newborn technology (ies) that you would like to provide feedback on. (Please Se... = Pulse Oximeter

Q3.14 13. Pulse Rate: Please rate your level of agreement with the statements under optimal and minimal. *Note: The optimal and minimal requirements define a range.*

	1-Disagree (1)	2-Somewhat Disagree (2)	3-Neither Agree nor Disagree (3)	4-Mostly Agree (4)	5-Fully agree (5)	Other - Do not have the expertise to comment (6)
**Optimal:** 25-250 bpm (1)	o	o	o	o	o	o
**Minimal:** 60-200 bpm (2)	o	o	o	o	o	o

Display This Question:

If Please select the newborn technology (ies) that you would like to provide feedback on. (Please Se... = Pulse Oximeter

Q3.15 14. Pulse Rate: Please provide reasoning if you have chosen 3 or below.

____

____

____

____

____

Display This Question:

If Please select the newborn technology (ies) that you would like to provide feedback on. (Please Se... = Pulse Oximeter

Q3.16 15. Pulse Rate Accuracy: Please rate your level of agreement with the statements under optimal and minimal. *Note: The optimal and minimal requirements define a range.*

	1-Disagree (1)	2-Somewhat Disagree (2)	3-Neither Agree nor Disagree (3)	4-Mostly Agree (4)	5-Fully agree (5)	Other - Do not have the expertise to comment (6)
**Optimal:** +-3 bpm (1)	o	o	o	o	o	o
**Minimal:** Same as Optimal. (2)	o	o	o	o	o	o

Display This Question:

If Please select the newborn technology (ies) that you would like to provide feedback on. (Please Se... = Pulse Oximeter

Q3.17 16. Pulse Rate Accuracy: Please provide reasoning if you have chosen 3 or below.

____

____

____

____

____

Display This Question:

If Please select the newborn technology (ies) that you would like to provide feedback on. (Please Se... = Pulse Oximeter

Q3.18 17. Pulse Rate Resolution: Please rate your level of agreement with the statements under optimal and minimal. *Note: The optimal and minimal requirements define a range.*

	1-Disagree (1)	2-Somewhat Disagree (2)	3-Neither Agree nor Disagree (3)	4-Mostly Agree (4)	5-Fully agree (5)	Other - Do not have the expertise to comment (6)
**Optimal:** 1 bpm (1)	o	o	o	o	o	o
**Minimal:** Same as Optimal. (2)	o	o	o	o	o	o

Display This Question:

If Please select the newborn technology (ies) that you would like to provide feedback on. (Please Se... = Pulse Oximeter

Q3.19 18. Pulse Rate Resolution: Please provide reasoning if you have chosen 3 or below.

____

____

____

____

____

Display This Question:

If Please select the newborn technology (ies) that you would like to provide feedback on. (Please Se... = Pulse Oximeter

Q3.20 19. Sp02 Accuracy: Please rate your level of agreement with the statements under optimal and minimal. *Note: The optimal and minimal requirements define a range.*

	1-Disagree (1)	2-Somewhat Disagree (2)	3-Neither Agree nor Disagree (3)	4-Mostly Agree (4)	5-Fully agree (5)	Other - Do not have the expertise to comment (6)
**Optimal:** +-2% (1)	○	○	○	○	○	○
**Minimal:** +-3% (2)	○	○	○	○	○	○

Display This Question:

If Please select the newborn technology (ies) that you would like to provide feedback on. (Please Se... = Pulse Oximeter

Q3.21 20. Sp02 Accuracy: Please provide reasoning if you have chosen 3 or below.

____

____

____

____

____

Display This Question:

If Please select the newborn technology (ies) that you would like to provide feedback on. (Please Se... = Pulse Oximeter

Q3.22 21. Sp02 Range: Please rate your level of agreement with the statements under optimal and minimal. *Note: The optimal and minimal requirements define a range.*

	1-Disagree (1)	2-Somewhat Disagree (2)	3-Neither Agree nor Disagree (3)	4-Mostly Agree (4)	5-Fully agree (5)	Other - Do not have the expertise to comment (6)
**Optimal:** 0-100% (1)	○	○	○	○	○	○
**Minimal:** 70-100% (2)	○	○	○	○	○	○

Display This Question:

If Please select the newborn technology (ies) that you would like to provide feedback on. (Please Se... = Pulse Oximeter

Q3.23 22. Sp02 Range: Please provide reasoning if you have chosen 3 or below.

____

____

____

____

____

Display This Question:

If Please select the newborn technology (ies) that you would like to provide feedback on. (Please Se... = Pulse Oximeter

Q3.24 23. Alarms: Please rate your level of agreement with the statements under optimal and minimal. *Note: The optimal and minimal requirements define a range.*

	1-Disagree (1)	2-Somewhat Disagree (2)	3-Neither Agree nor Disagree (3)	4-Mostly Agree (4)	5-Fully agree (5)	Other - Do not have the expertise to comment (6)
**Optimal:** Visual and Auditory (1)	○	○	○	○	○	○
**Minimal:** Visual (2)	○	○	○	○	○	○

Display This Question:

If Please select the newborn technology (ies) that you would like to provide feedback on. (Please Se... = Pulse Oximeter

Q3.25 24. Alarms: Please provide reasoning if you have chosen 3 or below.

____

____

____

____

____

Display This Question:

If Please select the newborn technology (ies) that you would like to provide feedback on. (Please Se... = Pulse Oximeter

Q3.26 25. Consumables: Please rate your level of agreement with the statements under optimal and minimal. *Note: The optimal and minimal requirements define a range.*

	1-Disagree (1)	2-Somewhat Disagree (2)	3-Neither Agree nor Disagree (3)	4-Mostly Agree (4)	5-Fully agree (5)	Other - Do not have the expertise to comment (6)
**Optimal:** >12 months before required (1)	◯	◯	◯	◯	◯	◯
**Minimal:** >6 months before required (2)	◯	◯	◯	◯	◯	◯

Display This Question:

If Please select the newborn technology (ies) that you would like to provide feedback on. (Please Se... = Pulse Oximeter

Q3.27 26. Consumables: Please provide reasoning if you have chosen 3 or below.

____

____

____

____

____

Display This Question:

If Please select the newborn technology (ies) that you would like to provide feedback on. (Please Se... = Pulse Oximeter

Q3.28 27. Alarm Limits - PR: Please rate your level of agreement with the statements under optimal and minimal. *Note: The optimal and minimal requirements define a range.*

	1-Disagree (1)	2-Somewhat Disagree (2)	3-Neither Agree nor Disagree (3)	4-Mostly Agree (4)	5-Fully agree (5)	Other - Do not have the expertise to comment (6)
**Optimal:** Adjustable (1)	o	o	o	o	o	o
**Minimal:** 80-160 bpm (2)	o	o	o	o	o	o

Display This Question:

If Please select the newborn technology (ies) that you would like to provide feedback on. (Please Se... = Pulse Oximeter

Q3.29 28. Alarm Limits - PR: Please provide reasoning if you have chosen 3 or below.

____

____

____

____

____

Display This Question:

If Please select the newborn technology (ies) that you would like to provide feedback on. (Please Se... = Pulse Oximeter

Q3.30 29. Alarm Limits - Sp02: Please rate your level of agreement with the statements under optimal and minimal. *Note: The optimal and minimal requirements define a range.*

	1-Disagree (1)	2-Somewhat Disagree (2)	3-Neither Agree nor Disagree (3)	4-Mostly Agree (4)	5-Fully agree (5)	Other - Do not have the expertise to comment (6)
**Optimal:** Adjustable (1)	o	o	o	o	o	o
**Minimal:** 88-99% (2)	o	o	o	o	o	o

Display This Question:

If Please select the newborn technology (ies) that you would like to provide feedback on. (Please Se... = Pulse Oximeter

Q3.31 30. Alarm Limits - Sp02: Please provide reasoning if you have chosen 3 or below.

____

____

____

____

____

Display This Question:

If Please select the newborn technology (ies) that you would like to provide feedback on. (Please Se... = Pulse Oximeter

Q3.32 31. Continuous Measurement: Please rate your level of agreement with the statements under optimal and minimal. *Note: The optimal and minimal requirements define a range.*

	1-Disagree (1)	2-Somewhat Disagree (2)	3-Neither Agree nor Disagree (3)	4-Mostly Agree (4)	5-Fully agree (5)	Other - Do not have the expertise to comment (6)
**Optimal:** Yes (1)	○	○	○	○	○	○
**Minimal:** Same as optimal (2)	○	○	○	○	○	○

Display This Question:

If Please select the newborn technology (ies) that you would like to provide feedback on. (Please Se... = Pulse Oximeter

Q3.33 32. Continuous Measurement: Please provide reasoning if you have chosen 3 or below.

____

____

____

____

____

Display This Question:

If Please select the newborn technology (ies) that you would like to provide feedback on. (Please Se... = Pulse Oximeter

Q3.34 33. Decontamination: Please rate your level of agreement with the statements under optimal and minimal. *Note: The optimal and minimal requirements define a range.*

	1-Disagree (1)	2-Somewhat Disagree (2)	3-Neither Agree nor Disagree (3)	4-Mostly Agree (4)	5-Fully agree (5)	Other - Do not have the expertise to comment (6)
**Optimal:** Easy to clean with common disinfecting agents (1)	○	○	○	○	○	○
**Minimal:** Same as optimal (2)	○	○	○	○	○	○

Display This Question:

If Please select the newborn technology (ies) that you would like to provide feedback on. (Please Se... = Pulse Oximeter

Q3.35 34. Decontamination: Please provide reasoning if you have chosen 3 or below.

____

____

____

____

____

Display This Question:

If Please select the newborn technology (ies) that you would like to provide feedback on. (Please Se... = Pulse Oximeter

Q3.36 35. Patient Interface: Please rate your level of agreement with the statements under optimal and minimal. *Note: The optimal and minimal requirements define a range.*

	1-Disagree (1)	2-Somewhat Disagree (2)	3-Neither Agree nor Disagree (3)	4-Mostly Agree (4)	5-Fully agree (5)	Other - Do not have the expertise to comment (6)
**Optimal:** Neonate specific, biocompatible and reusable. (1)	○	○	○	○	○	○
**Minimal:** Same as optimal (2)	○	○	○	○	○	○

Display This Question:

If Please select the newborn technology (ies) that you would like to provide feedback on. (Please Se... = Pulse Oximeter

Q3.37 36. Patient Interface: Please provide reasoning if you have chosen 3 or below.

____

____

____

____

____

Display This Question:

If Please select the newborn technology (ies) that you would like to provide feedback on. (Please Se... = Pulse Oximeter

Q3.38 37. Size: Please rate your level of agreement with the statements under optimal and minimal. *Note: The optimal and minimal requirements define a range.*

	1-Disagree (1)	2-Somewhat Disagree (2)	3-Neither Agree nor Disagree (3)	4-Mostly Agree (4)	5-Fully agree (5)	Other - Do not have the expertise to comment (6)
**Optimal:** Small footprint, left at bedside with dock. (1)	o	o	o	o	o	o
**Minimal:** Handheld with dock. (2)	o	o	o	o	o	o

Display This Question:

If Please select the newborn technology (ies) that you would like to provide feedback on. (Please Se... = Pulse Oximeter

Q3.39 38. Size: Please provide reasoning if you have chosen 3 or below.

____

____

____

____

____

Display This Question:

If Please select the newborn technology (ies) that you would like to provide feedback on. (Please Se... = Pulse Oximeter

Q3.40 39. Training Required: Please rate your level of agreement with the statements under optimal and minimal. *Note: The optimal and minimal requirements define a range.*

	1-Disagree (1)	2-Somewhat Disagree (2)	3-Neither Agree nor Disagree (3)	4-Mostly Agree (4)	5-Fully agree (5)	Other - Do not have the expertise to comment (6)
**Optimal:** Minimal (1)	o	o	o	o	o	o
**Minimal:** Minimal (2)	o	o	o	o	o	o

Display This Question:

If Please select the newborn technology (ies) that you would like to provide feedback on. (Please Se... = Pulse Oximeter

Q3.41 40. Training Required: Please provide reasoning if you have chosen 3 or below.

____

____

____

____

____

Display This Question:

If Please select the newborn technology (ies) that you would like to provide feedback on. (Please Se... = Pulse Oximeter

Q3.42 41. User Manual: Please rate your level of agreement with the statements under optimal and minimal. *Note: The optimal and minimal requirements define a range.*

	1-Disagree (1)	2-Somewhat Disagree (2)	3-Neither Agree nor Disagree (3)	4-Mostly Agree (4)	5-Fully agree (5)	Other - Do not have the expertise to comment (6)
**Optimal:** User manual and additional training materials (checklists, videos, guides) in English and local language. Attached to device with labels and markings where possible. (1)	○	○	○	○	○	○
**Minimal:** User manual provided. (2)	○	○	○	○	○	○

Display This Question:

If Please select the newborn technology (ies) that you would like to provide feedback on. (Please Se... = Pulse Oximeter

Q3.43 42. User Manual: Please provide reasoning if you have chosen 3 or below.

____

____

____

____

____

Display This Question:

If Please select the newborn technology (ies) that you would like to provide feedback on. (Please Se... = Pulse Oximeter

Q3.44 43. Usage Meter: Please rate your level of agreement with the statements under optimal and minimal. *Note: The optimal and minimal requirements define a range.*

	1-Disagree (1)	2-Somewhat Disagree (2)	3-Neither Agree nor Disagree (3)	4-Mostly Agree (4)	5-Fully agree (5)	Other - Do not have the expertise to comment (6)
**Optimal:** Digitally stored record displaying cumulative hours of operation. (1)	o	o	o	o	o	o
**Minimal:** Digitally stored record displaying 50 previous readings or >50 hours. (2)	o	o	o	o	o	o

Display This Question:

If Please select the newborn technology (ies) that you would like to provide feedback on. (Please Se... = Pulse Oximeter

Q3.45 44. Usage Meter: Please provide reasoning if you have chosen 3 or below.

____

____

____

____

____

Display This Question:

If Please select the newborn technology (ies) that you would like to provide feedback on. (Please Se... = Pulse Oximeter

Q3.46 45. Voltage: Please rate your level of agreement with the statements under optimal and minimal. *Note: The optimal and minimal requirements define a range.*

	1-Disagree (1)	2-Somewhat Disagree (2)	3-Neither Agree nor Disagree (3)	4-Mostly Agree (4)	5-Fully agree (5)	Other - Do not have the expertise to comment (6)
**Optimal:** 110-240V 50-60hz (1)	o	o	o	o	o	o
**Minimal:** 220-240V 50-60hz (2)	o	o	o	o	o	o

Display This Question:

If Please select the newborn technology (ies) that you would like to provide feedback on. (Please Se... = Pulse Oximeter

Q3.47 46. Voltage: Please provide reasoning if you have chosen 3 or below.

____

____

____

____

____

Display This Question:

If Please select the newborn technology (ies) that you would like to provide feedback on. (Please Se... = Pulse Oximeter

Q3.48 47. Battery Powered: Please rate your level of agreement with the statements under optimal and minimal. *Note: The optimal and minimal requirements define a range.*

	1-Disagree (1)	2-Somewhat Disagree (2)	3-Neither Agree nor Disagree (3)	4-Mostly Agree (4)	5-Fully agree (5)	Other - Do not have the expertise to comment (6)
**Optimal:** >24hr on single charge (1)	○	○	○	○	○	○
**Minimal:** None (2)	○	○	○	○	○	○

Display This Question:

If Please select the newborn technology (ies) that you would like to provide feedback on. (Please Se... = Pulse Oximeter

Q3.49 48. Battery Powered: Please provide reasoning if you have chosen 3 or below.

____

____

____

____

____

Display This Question:

If Please select the newborn technology (ies) that you would like to provide feedback on. (Please Se... = Pulse Oximeter

Q3.50 49. Weight: Please rate your level of agreement with the statements under optimal and minimal. *Note: The optimal and minimal requirements define a range.*

	1-Disagree (1)	2-Somewhat Disagree (2)	3-Neither Agree nor Disagree (3)	4-Mostly Agree (4)	5-Fully agree (5)	Other - Do not have the expertise to comment (6)
**Optimal:** (1)	○	○	○	○	○	○
**Minimal:** Same as optimal (2)	○	○	○	○	○	○

Display This Question:

If Please select the newborn technology (ies) that you would like to provide feedback on. (Please Se... = Pulse Oximeter

Q3.51 50. Weight: Please provide reasoning if you have chosen 3 or below.

____

____

____

____

____

Display This Question:

If Please select the newborn technology (ies) that you would like to provide feedback on. (Please Se... = Pulse Oximeter

Q3.52 51. Warranty: Please rate your level of agreement with the statements under optimal and minimal. *Note: The optimal and minimal requirements define a range.*

	1-Disagree (1)	2-Somewhat Disagree (2)	3-Neither Agree nor Disagree (3)	4-Mostly Agree (4)	5-Fully agree (5)	Other - Do not have the expertise to comment (6)
**Optimal:** 5 years (1)	○	○	○	○	○	○
**Minimal:** 1 year (2)	○	○	○	○	○	○

Display This Question:

If Please select the newborn technology (ies) that you would like to provide feedback on. (Please Se... = Pulse Oximeter

Q3.53 52. Warranty: Please provide reasoning if you have chosen 3 or below.

____

____

____

____

____

Display This Question:

If Please select the newborn technology (ies) that you would like to provide feedback on. (Please Se... = Pulse Oximeter

Q3.54 53. Instrument Pricing: Please rate your level of agreement with the statements under optimal and minimal. *Note: The optimal and minimal requirements define a range.*

	1-Disagree (1)	2-Somewhat Disagree (2)	3-Neither Agree nor Disagree (3)	4-Mostly Agree (4)	5-Fully agree (5)	Other - Do not have the expertise to comment (6)
**Optimal:** (1)	○	○	○	○	○	○
**Minimal:** (2)	○	○	○	○	○	○

Display This Question:

If Please select the newborn technology (ies) that you would like to provide feedback on. (Please Se... = Pulse Oximeter

Q3.55 54. Instrument Pricing: Please provide reasoning if you have chosen 3 or below.

____

____

____

____

____

Display This Question:

If Please select the newborn technology (ies) that you would like to provide feedback on. (Please Se... = Pulse Oximeter

Q3.56 55. Consumable Pricing: Please rate your level of agreement with the statements under optimal and minimal. *Note: The optimal and minimal requirements define a range.*

	1-Disagree (1)	2-Somewhat Disagree (2)	3-Neither Agree nor Disagree (3)	4-Mostly Agree (4)	5-Fully agree (5)	Other - Do not have the expertise to comment (6)
**Optimal:** (1)	○	○	○	○	○	○
**Minimal:** (2)	○	○	○	○	○	○

Display This Question:

If Please select the newborn technology (ies) that you would like to provide feedback on. (Please Se... = Pulse Oximeter

Q3.57 56. Consumable Pricing: Please provide reasoning if you have chosen 3 or below.

____

____

____

____

____

End of Block: Pulse Oximeter

Start of Block: Oxygen Concentrator

Display This Question:

If Please select the newborn technology (ies) that you would like to provide feedback on. (Please Se... = Oxygen Concentrator

Q4.1 Oxygen Concentrator

Display This Question:

If Please select the newborn technology (ies) that you would like to provide feedback on. (Please Se... = Oxygen Concentrator

Q4.2 1. Intended Use: Please rate your level of agreement with the statements under optimal and minimal. *Note: The optimal and minimal requirements define a range.*

	1-Disagree (1)	2-Somewhat Disagree (2)	3-Neither Agree nor Disagree (3)	4-Mostly Agree (4)	5-Fully agree (5)	Other - Do not have the expertise to comment (6)
**Optimal:** To provide medical oxygen for use in a healthcare setting. (1)	○	○	○	○	○	○
**Minimal:** Same as Optimal. (2)	○	○	○	○	○	○

Display This Question:

If Please select the newborn technology (ies) that you would like to provide feedback on. (Please Se... = Oxygen Concentrator

Q4.3 2. Intended Use: Please provide reasoning if you have chosen 3 or below.

____

____

____

____

____

Display This Question:

If Please select the newborn technology (ies) that you would like to provide feedback on. (Please Se... = Oxygen Concentrator

Q4.4 3. Target Operator: Please rate your level of agreement with the statements under optimal and minimal. *Note: The optimal and minimal requirements define a range.*

	1-Disagree (1)	2-Somewhat Disagree (2)	3-Neither agree nor disagree (3)	4-Mostly Agre (4)	5-Fully Agree (5)	Other - Do not have the expertise to comment (6)
**Optimal:** For use in low- and middle-income countries by a wide variety of clinicians, including nurses, clinical officers, and pediatricians. (1)	○	○	○	○	○	○
**Minimal:** Same as Optimal (2)	○	○	○	○	○	○

Display This Question:

If Please select the newborn technology (ies) that you would like to provide feedback on. (Please Se... = Oxygen Concentrator

Q4.5 4. Target Operator: Please provide reasoning if you have chosen 3 or below.

____

Display This Question:

If Please select the newborn technology (ies) that you would like to provide feedback on. (Please Se... = Oxygen Concentrator

Q4.6 5. Target Population: Please rate your level of agreement with the statements under optimal and minimal. *Note: The optimal and minimal requirements define a range.*

	1-Disagree (1)	2-Somewhat Disagree (2)	3-Neither Agree nor Disagree (3)	4-Mostly Agree (4)	5-Fully agree (5)	Other - Do not have the expertise to comment (6)
**Optimal:** Neonates ((1)	○	○	○	○	○	○
**Minimal:** Same as Optimal. (2)	○	○	○	○	○	○

Display This Question:

If Please select the newborn technology (ies) that you would like to provide feedback on. (Please Se... = Oxygen Concentrator

Q4.7 6. Target Population: Please provide reasoning if you have chosen 3 or below.

____

Display This Question:

If Please select the newborn technology (ies) that you would like to provide feedback on. (Please Se... = Oxygen Concentrator

Q4.8 7. Target Setting: Please rate your level of agreement with the statements under optimal and minimal. *Note: The optimal and minimal requirements define a range.*

	1-Disagree (1)	2-Somewhat Disagree (2)	3-Neither Agree nor Disagree (3)	4-Mostly Agree (4)	5-Fully agree (5)	Other - Do not have the expertise to comment (6)
**Optimal:** Hospitals in low-resource settings (1)	o	o	o	o	o	o
**Minimal:** Same as Optimal. (2)	o	o	o	o	o	o

Display This Question:

If Please select the newborn technology (ies) that you would like to provide feedback on. (Please Se... = Oxygen Concentrator

Q4.9 8. Target Setting: Please provide reasoning if you have chosen 3 or below.

____

____

____

____

____

Display This Question:

If Please select the newborn technology (ies) that you would like to provide feedback on. (Please Se... = Oxygen Concentrator

Q4.10 9. International Standard: Please rate your level of agreement with the statements under optimal and minimal. *Note: The optimal and minimal requirements define a range.*

	1-Disagree (1)	2-Somewhat Disagree (2)	3-Neither Agree nor Disagree (3)	4-Mostly Agree (4)	5-Fully agree (5)	Other - Do not have the expertise to comment (6)
**Optimal:** ISO 13485:2016 Medical devices – Quality management systems -- Requirements for regulatory purposes. (1)	o	o	o	o	o	o
**Minimal:** Same as Optimal. (2)	o	o	o	o	o	o

Display This Question:

If Please select the newborn technology (ies) that you would like to provide feedback on. (Please Se... = Oxygen Concentrator

Q4.11 10. International Standard: Please provide reasoning if you have chosen 3 or below.

____

____

____

____

____

Display This Question:

If Please select the newborn technology (ies) that you would like to provide feedback on. (Please Se... = Oxygen Concentrator

Q4.12 11. Regulation: Please rate your level of agreement with the statements under optimal and minimal. *Note: The optimal and minimal requirements define a range.*

	1-Disagree (1)	2-Somewhat Disagree (2)	3-Neither Agree nor Disagree (3)	4-Mostly Agree (4)	5-Fully agree (5)	Other - Do not have the expertise to comment (6)
**Optimal:** CE marking or US FDA Clearance (1)	○	○	○	○	○	○
**Minimal:** Same as Optimal. (2)	○	○	○	○	○	○

Display This Question:

If Please select the newborn technology (ies) that you would like to provide feedback on. (Please Se... = Oxygen Concentrator

Q4.13 12. Regulation: Please provide reasoning if you have chosen 3 or below.

____

____

____

____

____

Display This Question:

If Please select the newborn technology (ies) that you would like to provide feedback on. (Please Se... = Oxygen Concentrator

Q4.14 13. Flow Meter: Please rate your level of agreement with the statements under optimal and minimal. *Note: The optimal and minimal requirements define a range.*

	1-Disagree (1)	2-Somewhat Disagree (2)	3-Neither Agree nor Disagree (3)	4-Mostly Agree (4)	5-Fully agree (5)	Other - Do not have the expertise to comment (6)
**Optimal:** At least 2 with each 0 to 5 SLPM flow meter, min incremental 0.5 SLPM (1)	○	○	○	○	○	○
**Minimal:** At least 1 with 0 to 8 SLPM flow meter, min incremental 0.5 SLPM (2)	○	○	○	○	○	○

Display This Question:

If Please select the newborn technology (ies) that you would like to provide feedback on. (Please Se... = Oxygen Concentrator

Q4.15 14. Flow Meter: Please provide reasoning if you have chosen 3 or below.

____

____

____

____

____

Display This Question:

If Please select the newborn technology (ies) that you would like to provide feedback on. (Please Se... = Oxygen Concentrator

Q4.16 15. Flow Rate: Please rate your level of agreement with the statements under optimal and minimal. *Note: The optimal and minimal requirements define a range.*

	1-Disagree (1)	2-Somewhat Disagree (2)	3-Neither Agree nor Disagree (3)	4-Mostly Agree (4)	5-Fully agree (5)	Other - Do not have the expertise to comment (6)
**Optimal:** 10 SLPM (1)	○	○	○	○	○	○
**Minimal:** 8-10 SLPM (2)	○	○	○	○	○	○

Display This Question:

If Please select the newborn technology (ies) that you would like to provide feedback on. (Please Se... = Oxygen Concentrator

Q4.17 16. Flow Rate: Please provide reasoning if you have chosen 3 or below.

____

____

____

____

____

Display This Question:

If Please select the newborn technology (ies) that you would like to provide feedback on. (Please Se... = Oxygen Concentrator

Q4.18 17. Time to Reach 95% of Specified Performance: Please rate your level of agreement with the statements under optimal and minimal. *Note: The optimal and minimal requirements define a range.*

	1-Disagree (1)	2-Somewhat Disagree (2)	3-Neither Agree nor Disagree (3)	4-Mostly Agree (4)	5-Fully agree (5)	Other - Do not have the expertise to comment (6)
**Optimal:** < 5 Min (1)	○	○	○	○	○	○
**Minimal:** (2)	○	○	○	○	○	○

Display This Question:

If Please select the newborn technology (ies) that you would like to provide feedback on. (Please Se... = Oxygen Concentrator

Q4.19 18. Time to Reach 95% of Specified Performance: Please provide reasoning if you have chosen 3 or below.

____

____

____

____

____

Display This Question:

If Please select the newborn technology (ies) that you would like to provide feedback on. (Please Se... = Oxygen Concentrator

Q4.20 19. Oxygen Purity: Please rate your level of agreement with the statements under optimal and minimal. *Note: The optimal and minimal requirements define a range.*

	1-Disagree (1)	2-Somewhat Disagree (2)	3-Neither Agree nor Disagree (3)	4-Mostly Agree (4)	5-Fully agree (5)	Other - Do not have the expertise to comment (6)
**Optimal:** 93% +-3% (1)	o	o	o	o	o	o
**Minimal:** Same as Optimal. (2)	o	o	o	o	o	o

Display This Question:

If Please select the newborn technology (ies) that you would like to provide feedback on. (Please Se... = Oxygen Concentrator

Q4.21 20. Oxygen Purity: Please provide reasoning if you have chosen 3 or below.

____

____

____

____

____

Display This Question:

If Please select the newborn technology (ies) that you would like to provide feedback on. (Please Se... = Oxygen Concentrator

Q4.22 21. Alarms: Please rate your level of agreement with the statements under optimal and minimal. *Note: The optimal and minimal requirements define a range.*

	1-Disagree (1)	2-Somewhat Disagree (2)	3-Neither Agree nor Disagree (3)	4-Mostly Agree (4)	5-Fully agree (5)	Other - Do not have the expertise to comment (6)
**Optimal:** Audible and/or visual alarms for high temperature, flow rate and pressure (1)	o	o	o	o	o	o
**Minimal:** Same as Optimal. (2)	o	o	o	o	o	o

Display This Question:

If Please select the newborn technology (ies) that you would like to provide feedback on. (Please Se... = Oxygen Concentrator

Q4.23 22. Alarms: Please provide reasoning if you have chosen 3 or below.

____

____

____

____

____

Display This Question:

If Please select the newborn technology (ies) that you would like to provide feedback on. (Please Se... = Oxygen Concentrator

Q4.24 23. Indicators: Please rate your level of agreement with the statements under optimal and minimal. *Note: The optimal and minimal requirements define a range.*

	1-Disagree (1)	2-Somewhat Disagree (2)	3-Neither Agree nor Disagree (3)	4-Mostly Agree (4)	5-Fully agree (5)	Other - Do not have the expertise to comment (6)
**Optimal:** Clearly labeled or marked with pictures and language. Audible alerts and diagnostic indicator where possible (1)	o	o	o	o	o	o
**Minimal:** UI easy to understand, numbers and displays clearly visible (2)	o	o	o	o	o	o

Display This Question:

If Please select the newborn technology (ies) that you would like to provide feedback on. (Please Se... = Oxygen Concentrator

Q4.25 24. Indicators: Please provide reasoning if you have chosen 3 or below.

____

____

____

____

____

Display This Question:

If Please select the newborn technology (ies) that you would like to provide feedback on. (Please Se... = Oxygen Concentrator

Q4.26 25. Mobility: Please rate your level of agreement with the statements under optimal and minimal. *Note: The optimal and minimal requirements define a range.*

	1-Disagree (1)	2-Somewhat Disagree (2)	3-Neither Agree nor Disagree (3)	4-Mostly Agree (4)	5-Fully agree (5)	Other - Do not have the expertise to comment (6)
**Optimal:** Four antistatic swivel castors, two with brakers, integrated handle (1)	○	○	○	○	○	○
**Minimal:** Four wheels (2)	○	○	○	○	○	○

Display This Question:

If Please select the newborn technology (ies) that you would like to provide feedback on. (Please Se... = Oxygen Concentrator

Q4.27 26. Mobility: Please provide reasoning if you have chosen 3 or below.

____

____

____

____

____

Display This Question:

If Please select the newborn technology (ies) that you would like to provide feedback on. (Please Se... = Oxygen Concentrator

Q4.28 27. Oxygen Monitor: Please rate your level of agreement with the statements under optimal and minimal. *Note: The optimal and minimal requirements define a range.*

	1-Disagree (1)	2-Somewhat Disagree (2)	3-Neither Agree nor Disagree (3)	4-Mostly Agree (4)	5-Fully agree (5)	Other - Do not have the expertise to comment (6)
**Optimal:** Visual and audible status indicator for three ranges of oxygen concentration preferably with color coding for early warning. (1)	○	○	○	○	○	○
**Minimal:** Visual and audible status. (2)	○	○	○	○	○	○

Display This Question:

If Please select the newborn technology (ies) that you would like to provide feedback on. (Please Se... = Oxygen Concentrator

Q4.29 28. Oxygen Monitor: Please provide reasoning if you have chosen 3 or below.

____

____

____

____

____

Display This Question:

If Please select the newborn technology (ies) that you would like to provide feedback on. (Please Se... = Oxygen Concentrator

Q4.30 29. Oxygen Outlet: Please rate your level of agreement with the statements under optimal and minimal. *Note: The optimal and minimal requirements define a range.*

	1-Disagree (1)	2-Somewhat Disagree (2)	3-Neither Agree nor Disagree (3)	4-Mostly Agree (4)	5-Fully agree (5)	Other - Do not have the expertise to comment (6)
**Optimal:** Recessed, replaceable metal barbs (1)	o	o	o	o	o	o
**Minimal:** Recessed, replaceable metal or plastic barbs (2)	o	o	o	o	o	o

Display This Question:

If Please select the newborn technology (ies) that you would like to provide feedback on. (Please Se... = Oxygen Concentrator

Q4.31 30. Oxygen Outlet: Please provide reasoning if you have chosen 3 or below.

____

____

____

____

____

Display This Question:

If Please select the newborn technology (ies) that you would like to provide feedback on. (Please Se... = Oxygen Concentrator

Q4.32 31. Sound Level: Please rate your level of agreement with the statements under optimal and minimal. *Note: The optimal and minimal requirements define a range.*

	1-Disagree (1)	2-Somewhat Disagree (2)	3-Neither Agree nor Disagree (3)	4-Mostly Agree (4)	5-Fully agree (5)	Other - Do not have the expertise to comment (6)
**Optimal:** ≤50 decibels (1)	o	o	o	o	o	o
**Minimal:** 50 decibels (2)	o	o	o	o	o	o

Display This Question:

If Please select the newborn technology (ies) that you would like to provide feedback on. (Please Se... = Oxygen Concentrator

Q4.33 32. Sound Level: Please provide reasoning if you have chosen 3 or below.

____

____

____

____

____

Display This Question:

If Please select the newborn technology (ies) that you would like to provide feedback on. (Please Se... = Oxygen Concentrator

Q4.34 33. Decontamination: Please rate your level of agreement with the statements under optimal and minimal. *Note: The optimal and minimal requirements define a range.*

	1-Disagree (1)	2- Somewhat Disagree (2)	3-Neither Agree nor Disagree (3)	4-Mostly Agree (4)	5-Fully agree (5)	Other - Do not have the expertise to comment (6)
**Optimal:** Reduced recessed areas and need for specialized cleaning procedures or products (1)	○	○	○	○	○	○
**Minimal:** Easy to clean flat surfaces, compatible with common disinfecting agents (2)	○	○	○	○	○	○

Display This Question:

If Please select the newborn technology (ies) that you would like to provide feedback on. (Please Se... = Oxygen Concentrator

Q4.35 34. Decontamination: Please provide reasoning if you have chosen 3 or below.

____

____

____

____

____

Display This Question:

If Please select the newborn technology (ies) that you would like to provide feedback on. (Please Se... = Oxygen Concentrator

Q4.36 35. Weight: Please rate your level of agreement with the statements under optimal and minimal. *Note: The optimal and minimal requirements define a range.*

	1-Disagree (1)	2-Somewhat Disagree (2)	3-Neither Agree nor Disagree (3)	4-Mostly Agree (4)	5-Fully agree (5)	Other - Do not have the expertise to comment (6)
**Optimal:** (1)	○	○	○	○	○	○
**Minimal:** Same as Optimal. (2)	○	○	○	○	○	○

Display This Question:

If Please select the newborn technology (ies) that you would like to provide feedback on. (Please Se... = Oxygen Concentrator

Q4.37 36. Weight: Please provide reasoning if you have chosen 3 or below.

____

____

____

____

____

Display This Question:

If Please select the newborn technology (ies) that you would like to provide feedback on. (Please Se... = Oxygen Concentrator

Q4.38 37. User Instructions: Please rate your level of agreement with the statements under optimal and minimal. *Note: The optimal and minimal requirements define a range.*

	1-Disagree (1)	2-Somewhat Disagree (2)	3-Neither Agree nor Disagree (3)	4-Mostly Agree (4)	5-Fully agree (5)	Other - Do not have the expertise to comment (6)
**Optimal:** User manual and additional training materials (checklists, videos, guides) in English and local language. Attached to device with labels and markings where possible. (1)	○	○	○	○	○	○
**Minimal:** Instruction manual provided. (2)	○	○	○	○	○	○

Display This Question:

If Please select the newborn technology (ies) that you would like to provide feedback on. (Please Se... = Oxygen Concentrator

Q4.39 38. User Instructions: Please provide reasoning if you have chosen 3 or below.

____

____

____

____

____

Display This Question:

If Please select the newborn technology (ies) that you would like to provide feedback on. (Please Se... = Oxygen Concentrator

Q4.40 39. Durability and Robustness: Please rate your level of agreement with the statements under optimal and minimal. *Note: The optimal and minimal requirements define a range.*

	1-Disagree (1)	2-Somewhat Disagree (2)	3-Neither Agree nor Disagree (3)	4-Mostly Agree (4)	5-Fully agree (5)	Other - Do not have the expertise to comment (6)
**Optimal:** Harsh ambient condition, temperature 5-45 °C, humidity 15% to 95%, dusty air, elevation >=2000 meters (1)	o	o	o	o	o	o
**Minimal:** temperature 10-40 °C, humidity 15%-95% elevation up to 2000 meters (2)	o	o	o	o	o	o

Display This Question:

If Please select the newborn technology (ies) that you would like to provide feedback on. (Please Se... = Oxygen Concentrator

Q4.41 40. Durability and Robustness: Please provide reasoning if you have chosen 3 or below.

____

____

____

____

____

Display This Question:

If Please select the newborn technology (ies) that you would like to provide feedback on. (Please Se... = Oxygen Concentrator

Q4.42 41. Usage Meter: Please rate your level of agreement with the statements under optimal and minimal. *Note: The optimal and minimal requirements define a range.*

	1-Disagree (1)	2-Somewhat Disagree (2)	3-Neither Agree nor Disagree (3)	4-Mostly Agree (4)	5-Fully agree (5)	Other - Do not have the expertise to comment (6)
**Optimal:** Non-resettable digital or analog meter displaying cumulative hours of operation. (1)	o	o	o	o	o	o
**Minimal:** Same as Optimal. (2)	o	o	o	o	o	o

Display This Question:

If Please select the newborn technology (ies) that you would like to provide feedback on. (Please Se... = Oxygen Concentrator

Q4.43 42. Usage Meter: Please provide reasoning if you have chosen 3 or below.

____

____

____

____

____

Display This Question:

If Please select the newborn technology (ies) that you would like to provide feedback on. (Please Se... = Oxygen Concentrator

Q4.44 43. Cleaning Interval: Please rate your level of agreement with the statements under optimal and minimal. *Note: The optimal and minimal requirements define a range.*

	1-Disagree (1)	2-Somewhat Disagree (2)	3-Neither Agree nor Disagree (3)	4-Mostly Agree (4)	5-Fully agree (5)	Other - Do not have the expertise to comment (6)
**Optimal:** None Required. (1)	o	o	o	o	o	o
**Minimal:** Weekly cleaning of external course filter. (2)	o	o	o	o	o	o

Display This Question:

If Please select the newborn technology (ies) that you would like to provide feedback on. (Please Se... = Oxygen Concentrator

Q4.45 44. Cleaning Interval: Please provide reasoning if you have chosen 3 or below.

____

____

____

____

____

Display This Question:

If Please select the newborn technology (ies) that you would like to provide feedback on. (Please Se... = Oxygen Concentrator

Q4.46 45. Preventive Maintenance Interval: Please rate your level of agreement with the statements under optimal and minimal. *Note: The optimal and minimal requirements define a range.*

	1-Disagree (1)	2-Somewhat Disagree (2)	3-Neither Agree nor Disagree (3)	4-Mostly Agree (4)	5-Fully agree (5)	Other - Do not have the expertise to comment (6)
**Optimal:** Minimal to none (1)	o	o	o	o	o	o
**Minimal:** Every 24 months (2)	o	o	o	o	o	o

Display This Question:

If Please select the newborn technology (ies) that you would like to provide feedback on. (Please Se... = Oxygen Concentrator

Q4.47 46. Preventive Maintenance Interval: Please provide reasoning if you have chosen 3 or below.

____

____

____

____

____

Display This Question:

If Please select the newborn technology (ies) that you would like to provide feedback on. (Please Se... = Oxygen Concentrator

Q4.48 47. Replacement Parts and Consumables: Please rate your level of agreement with the statements under optimal and minimal. *Note: The optimal and minimal requirements define a range.*

	1-Disagree (1)	2-Somewhat Disagree (2)	3-Neither Agree nor Disagree (3)	4-Mostly Agree (4)	5-Fully agree (5)	Other - Do not have the expertise to comment (6)
**Optimal:** None required (1)	○	○	○	○	○	○
**Minimal:** None required for 24 months (2)	○	○	○	○	○	○

Display This Question:

If Please select the newborn technology (ies) that you would like to provide feedback on. (Please Se... = Oxygen Concentrator

Q4.49 48. Replacement Parts and Consumables: Please provide reasoning if you have chosen 3 or below.

____

____

____

____

____

Display This Question:

If Please select the newborn technology (ies) that you would like to provide feedback on. (Please Se... = Oxygen Concentrator

Q4.50 49. Warranty: Please rate your level of agreement with the statements under optimal and minimal. *Note: The optimal and minimal requirements define a range.*

	1-Disagree (1)	2-Somewhat Disagree (2)	3-Neither Agree nor Disagree (3)	4-Mostly Agree (4)	5-Fully agree (5)	Other - Do not have the expertise to comment (6)
**Optimal:** 5 years (1)	○	○	○	○	○	○
**Minimal:** 1 year (2)	○	○	○	○	○	○

Display This Question:

If Please select the newborn technology (ies) that you would like to provide feedback on. (Please Se... = Oxygen Concentrator

Q4.51 50. Warranty: Please provide reasoning if you have chosen 3 or below.

____

____

____

Display This Question:

If Please select the newborn technology (ies) that you would like to provide feedback on. (Please Se... = Oxygen Concentrator

Q4.52 51. Technical Skill Maintenance: Please rate your level of agreement with the statements under optimal and minimal. *Note: The optimal and minimal requirements define a range.*

	1-Disagree (1)	2-Somewhat Disagree (2)	3-Neither Agree nor Disagree (3)	4-Mostly Agree (4)	5-Fully agree (5)	Other - Do not have the expertise to comment (6)
**Optimal:** Minimally trained technician (1)	○	○	○	○	○	○
**Minimal:** Trained technician with training in basic operation and maintenance (2)	○	○	○	○	○	○

Display This Question:

If Please select the newborn technology (ies) that you would like to provide feedback on. (Please Se... = Oxygen Concentrator

Q4.53 52. Technical Skill Maintenance: Please provide reasoning if you have chosen 3 or below.

____

____

____

____

____

Display This Question:

If Please select the newborn technology (ies) that you would like to provide feedback on. (Please Se... = Oxygen Concentrator

Q4.54 53. Tools Required: Please rate your level of agreement with the statements under optimal and minimal. *Note: The optimal and minimal requirements define a range.*

	1-Disagree (1)	2-Somewhat Disagree (2)	3-Neither Agree nor Disagree (3)	4-Mostly Agree (4)	5-Fully agree (5)	Other - Do not have the expertise to comment (6)
**Optimal:** No specialized tools required (1)	○	○	○	○	○	○
**Minimal:** Minimal specialized tools for sieve bed and filter replacement (2)	○	○	○	○	○	○

Display This Question:

If Please select the newborn technology (ies) that you would like to provide feedback on. (Please Se... = Oxygen Concentrator

Q4.55 54. Tools Required: Please provide reasoning if you have chosen 3 or below.

____

____

____

____

____

Display This Question:

If Please select the newborn technology (ies) that you would like to provide feedback on. (Please Se... = Oxygen Concentrator

Q4.56 55. User Skill Level: Please rate your level of agreement with the statements under optimal and minimal. *Note: The optimal and minimal requirements define a range.*

	1-Disagree (1)	2-Somewhat Disagree (2)	3-Neither Agree nor Disagree (3)	4-Mostly Agree (4)	5-Fully agree (5)	Other - Do not have the expertise to comment (6)
**Optimal:** Minimal to none. (1)	o	o	o	o	o	o
**Minimal:** Same as Optimal. (2)	o	o	o	o	o	o

Display This Question:

If Please select the newborn technology (ies) that you would like to provide feedback on. (Please Se... = Oxygen Concentrator

Q4.57 56. User Skill Level: Please provide reasoning if you have chosen 3 or below.

____

____

____

____

____

Display This Question:

If Please select the newborn technology (ies) that you would like to provide feedback on. (Please Se... = Oxygen Concentrator

Q4.58 57. Electrical Plug: Please rate your level of agreement with the statements under optimal and minimal. *Note: The optimal and minimal requirements define a range.*

	1-Disagree (1)	2-Somewhat Disagree (2)	3-Neither Agree nor Disagree (3)	4-Mostly Agree (4)	5-Fully agree (5)	Other - Do not have the expertise to comment (6)
**Optimal:** Universal conversion power adapter, compatible with local power outlet, rated above amperage voltage requirements (1)	o	o	o	o	o	o
**Minimal:** Compatible with local power outlet, rated above amperage voltage requirements (2)	o	o	o	o	o	o

Display This Question:

If Please select the newborn technology (ies) that you would like to provide feedback on. (Please Se... = Oxygen Concentrator

Q4.59 58. Electrical Plug: Please provide reasoning if you have chosen 3 or below.

____

____

____

____

____

Display This Question:

If Please select the newborn technology (ies) that you would like to provide feedback on. (Please Se... = Oxygen Concentrator

Q4.60 59. Filters: Please rate your level of agreement with the statements under optimal and minimal. *Note: The optimal and minimal requirements define a range.*

	1-Disagree (1)	2-Somewhat Disagree (2)	3-Neither Agree nor Disagree (3)	4-Mostly Agree (4)	5-Fully agree (5)	Other - Do not have the expertise to comment (6)
**Optimal:** Replaceable washable reusable (1)	o	o	o	o	o	o
**Minimal:** Same as Optimal (2)	o	o	o	o	o	o

Display This Question:

If Please select the newborn technology (ies) that you would like to provide feedback on. (Please Se... = Oxygen Concentrator

Q4.61 60. Filters: Please provide reasoning if you have chosen 3 or below.

____

____

____

____

____

Display This Question:

If Please select the newborn technology (ies) that you would like to provide feedback on. (Please Se... = Oxygen Concentrator

Q4.62 61. Power Consumption: Please rate your level of agreement with the statements under optimal and minimal. *Note: The optimal and minimal requirements define a range.*

	1-Disagree (1)	2-Somewhat Disagree (2)	3-Neither Agree nor Disagree (3)	4-Mostly Agree (4)	5-Fully agree (5)	Other - Do not have the expertise to comment (6)
**Optimal:** 275 W at 5 SPLM (1)	o	o	o	o	o	o
**Minimal:** Scales with delivery output — i.e., consumes less power at lower flow rates. (2)	o	o	o	o	o	o

Display This Question:

If Please select the newborn technology (ies) that you would like to provide feedback on. (Please Se... = Oxygen Concentrator

Q4.63 62. Power Consumption: Please provide reasoning if you have chosen 3 or below.

____

____

____

____

____

Display This Question:

If Please select the newborn technology (ies) that you would like to provide feedback on. (Please Se... = Oxygen Concentrator

Q4.64 63. Surge Protection: Please rate your level of agreement with the statements under optimal and minimal. *Note: The optimal and minimal requirements define a range.*

	1-Disagree (1)	2-Somewhat Disagree (2)	3-Neither Agree nor Disagree (3)	4-Mostly Agree (4)	5-Fully agree (5)	Other - Do not have the expertise to comment (6)
**Optimal:** Integrated (1)	○	○	○	○	○	○
**Minimal:** External (2)	○	○	○	○	○	○

Display This Question:

If Please select the newborn technology (ies) that you would like to provide feedback on. (Please Se... = Oxygen Concentrator

Q4.65 64. Surge Protection: Please provide reasoning if you have chosen 3 or below.

____

____

____

____

____

Display This Question:

If Please select the newborn technology (ies) that you would like to provide feedback on. (Please Se... = Oxygen Concentrator

Q4.66 63. Voltage: Please rate your level of agreement with the statements under optimal and minimal. *Note: The optimal and minimal requirements define a range.*

	1-Disagree (1)	2-Somewhat Disagree (2)	3-Neither Agree nor Disagree (3)	4-Mostly Agree (4)	5-Fully agree (5)	Other - Do not have the expertise to comment (6)
**Optimal:** 110-240 50-60hz (1)	○	○	○	○	○	○
**Minimal:** 220-240 50-60hz (2)	○	○	○	○	○	○

Display This Question:

If Please select the newborn technology (ies) that you would like to provide feedback on. (Please Se... = Oxygen Concentrator

Q4.67 64. Voltage: Please provide reasoning if you have chosen 3 or below.

____

____

____

____

____

Display This Question:

If Please select the newborn technology (ies) that you would like to provide feedback on. (Please Se... = Oxygen Concentrator

Q4.68 65. Instrument Pricing: Please rate your level of agreement with the statements under optimal and minimal. *Note: The optimal and minimal requirements define a range.*

	1-Disagree (1)	2-Somewhat Disagree (2)	3-Neither Agree nor Disagree (3)	4-Mostly Agree (4)	5-Fully agree (5)	Other - Do not have the expertise to comment (6)
**Optimal:** (1)	○	○	○	○	○	○
**Minimal:** (2)	○	○	○	○	○	○

Display This Question:

If Please select the newborn technology (ies) that you would like to provide feedback on. (Please Se... = Oxygen Concentrator

Q4.69 66. Instrument Pricing: Please provide reasoning if you have chosen 3 or below.

____

____

____

____

____

End of Block: Oxygen Concentrator

Start of Block: Flow Splitter

Display This Question:

If Please select the newborn technology (ies) that you would like to provide feedback on. (Please Se... = Flow Splitter

Q5.1 Flow Splitter

Display This Question:

If Please select the newborn technology (ies) that you would like to provide feedback on. (Please Se... = Flow Splitter

Q5.2 1. Intended Use: Please rate your level of agreement with the statements under optimal and minimal. *Note: The optimal and minimal requirements define a range.*

	1-Disagree (1)	2-Somewhat Disagree (2)	3-Neither Agree nor Disagree (3)	4-Mostly Agree (4)	5-Fully agree (5)	Other - Do not have the expertise to comment (6)
**Optimal:** To allow multiple patients to receive individually adjusted flow rates from a single oxygen source. (1)	○	○	○	○	○	○
**Minimal:** Same as Optimal. (2)	○	○	○	○	○	○

Display This Question:

If Please select the newborn technology (ies) that you would like to provide feedback on. (Please Se... = Flow Splitter

Q5.3 2. Intended Use: Please provide reasoning if you have chosen 3 or below.

____

____

____

____

____

Display This Question:

If Please select the newborn technology (ies) that you would like to provide feedback on. (Please Se... = Flow Splitter

Q5.4 3. Target Operator: Please rate your level of agreement with the statements under optimal and minimal. *Note: The optimal and minimal requirements define a range.*

	1-Disagree (1)	2-Somewhat Disagree (2)	3-Neither agree nor disagree (3)	4-Mostly Agre (4)	5-Fully Agree (5)	Other - Do not have the expertise to comment (6)
**Optimal:** For use in low- and middle-income countries by a wide variety of clinicians, including nurses, clinical officers, and pediatricians. (1)	○	○	○	○	○	○
**Minimal:** Same as Optimal (2)	○	○	○	○	○	○

Display This Question:

If Please select the newborn technology (ies) that you would like to provide feedback on. (Please Se... = Flow Splitter

Q5.5 4. Target Operator: Please provide reasoning if you have chosen 3 or below.

____

Display This Question:

If Please select the newborn technology (ies) that you would like to provide feedback on. (Please Se... = Flow Splitter

Q5.6 5. Target Population: Please rate your level of agreement with the statements under optimal and minimal. *Note: The optimal and minimal requirements define a range.*

	1-Disagree (1)	2-Somewhat Disagree (2)	3-Neither Agree nor Disagree (3)	4-Mostly Agree (4)	5-Fully agree (5)	Other - Do not have the expertise to comment (6)
**Optimal:** Neonates ((1)	○	○	○	○	○	○
**Minimal:** Same as Optimal. (2)	○	○	○	○	○	○

Display This Question:

If Please select the newborn technology (ies) that you would like to provide feedback on. (Please Se... = Flow Splitter

Q5.7 6. Target Population: Please provide reasoning if you have chosen 3 or below.

____

Display This Question:

If Please select the newborn technology (ies) that you would like to provide feedback on. (Please Se... = Flow Splitter

Q5.8 7. Target Setting: Please rate your level of agreement with the statements under optimal and minimal. *Note: The optimal and minimal requirements define a range.*

	1-Disagree (1)	2-Somewhat Disagree (2)	3-Neither Agree nor Disagree (3)	4-Mostly Agree (4)	5-Fully agree (5)	Other - Do not have the expertise to comment (6)
**Optimal:** Hospitals in low-resource settings (1)	○	○	○	○	○	○
**Minimal:** Same as Optimal. (2)	○	○	○	○	○	○

Display This Question:

If Please select the newborn technology (ies) that you would like to provide feedback on. (Please Se... = Flow Splitter

Q5.9 8. Target Setting: Please provide reasoning if you have chosen 3 or below.

____

____

____

____

____

Display This Question:

If Please select the newborn technology (ies) that you would like to provide feedback on. (Please Se... = Flow Splitter

Q5.10 9. International Standard: Please rate your level of agreement with the statements under optimal and minimal. *Note: The optimal and minimal requirements define a range.*

	1-Disagree (1)	2-Somewhat Disagree (2)	3-Neither Agree nor Disagree (3)	4-Mostly Agree (4)	5-Fully agree (5)	Other - Do not have the expertise to comment (6)
**Optimal:** ISO 13485:2016 Medical devices – Quality management systems -- Requirements for regulatory purposes. (1)	o	o	o	o	o	o
**Minimal:** Same as Optimal. (2)	o	o	o	o	o	o

Display This Question:

If Please select the newborn technology (ies) that you would like to provide feedback on. (Please Se... = Flow Splitter

Q5.11 10. International Standard: Please provide reasoning if you have chosen 3 or below.

____

____

____

____

____

Display This Question:

If Please select the newborn technology (ies) that you would like to provide feedback on. (Please Se... = Flow Splitter

Q5.12 11. Regulation: Please rate your level of agreement with the statements under optimal and minimal. *Note: The optimal and minimal requirements define a range.*

	1-Disagree (1)	2-Somewhat Disagree (2)	3-Neither Agree nor Disagree (3)	4-Mostly Agree (4)	5-Fully agree (5)	Other - Do not have the expertise to comment (6)
**Optimal:** CE marking or US FDA Clearance (1)	○	○	○	○	○	○
**Minimal:** Same as Optimal. (2)	○	○	○	○	○	○

Display This Question:

If Please select the newborn technology (ies) that you would like to provide feedback on. (Please Se... = Flow Splitter

Q5.13 12. Regulation: Please provide reasoning if you have chosen 3 or below.

____

____

____

____

____

Display This Question:

If Please select the newborn technology (ies) that you would like to provide feedback on. (Please Se... = Flow Splitter

Q5.14 13. Air Flow per Patient: Please rate your level of agreement with the statements under optimal and minimal. *Note: The optimal and minimal requirements define a range.*

	1-Disagree (1)	2-Somewhat Disagree (2)	3-Neither Agree nor Disagree (3)	4-Mostly Agree (4)	5-Fully agree (5)	Other - Do not have the expertise to comment (6)
**Optimal:** 0-2 L/min (1)	o	o	o	o	o	o
**Minimal:** Same as Optimal. (2)	o	o	o	o	o	o

Display This Question:

If Please select the newborn technology (ies) that you would like to provide feedback on. (Please Se... = Flow Splitter

Q5.15 14. Air Flow per Patient: Please provide reasoning if you have chosen 3 or below.

____

____

____

____

____

Display This Question:

If Please select the newborn technology (ies) that you would like to provide feedback on. (Please Se... = Flow Splitter

Q5.16 15. Flow Control: Please rate your level of agreement with the statements under optimal and minimal. *Note: The optimal and minimal requirements define a range.*

	1-Disagree (1)	2-Somewhat Disagree (2)	3-Neither Agree nor Disagree (3)	4-Mostly Agree (4)	5-Fully agree (5)	Other - Do not have the expertise to comment (6)
**Optimal:** Each patient has individually controlled flow rate. (1)	○	○	○	○	○	○
**Minimal:** Same as Optimal. (2)	○	○	○	○	○	○

Display This Question:

If Please select the newborn technology (ies) that you would like to provide feedback on. (Please Se... = Flow Splitter

Q5.17 16. Flow Control: Please provide reasoning if you have chosen 3 or below.

____

____

____

____

____

Display This Question:

If Please select the newborn technology (ies) that you would like to provide feedback on. (Please Se... = Flow Splitter

Q5.18 17. Number of Outputs: Please rate your level of agreement with the statements under optimal and minimal. *Note: The optimal and minimal requirements define a range.*

	1-Disagree (1)	2-Somewhat Disagree (2)	3-Neither Agree nor Disagree (3)	4-Mostly Agree (4)	5-Fully agree (5)	Other - Do not have the expertise to comment (6)
**Optimal:** 5 (1)	○	○	○	○	○	○
**Minimal:** 2 (2)	○	○	○	○	○	○

Display This Question:

If Please select the newborn technology (ies) that you would like to provide feedback on. (Please Se... = Flow Splitter

Q5.19 18. Number of Outputs: Please provide reasoning if you have chosen 3 or below.

____

____

____

____

____

Display This Question:

If Please select the newborn technology (ies) that you would like to provide feedback on. (Please Se... = Flow Splitter

Q5.20 19. Indication: Please rate your level of agreement with the statements under optimal and minimal. *Note: The optimal and minimal requirements define a range.*

	1-Disagree (1)	2-Somewhat Disagree (2)	3-Neither Agree nor Disagree (3)	4-Mostly Agree (4)	5-Fully agree (5)	Other - Do not have the expertise to comment (6)
**Optimal:** Each flow rate has a visual indicator. (1)	o	o	o	o	o	o
**Minimal:** Same as Optimal. (2)	o	o	o	o	o	o

Display This Question:

If Please select the newborn technology (ies) that you would like to provide feedback on. (Please Se... = Flow Splitter

Q5.21 20. Indication: Please provide reasoning if you have chosen 3 or below.

____

____

____

____

____

Display This Question:

If Please select the newborn technology (ies) that you would like to provide feedback on. (Please Se... = Flow Splitter

Q5.22 21. Maintenance: Please rate your level of agreement with the statements under optimal and minimal. *Note: The optimal and minimal requirements define a range.*

	1-Disagree (1)	2-Somewhat Disagree (2)	3-Neither Agree nor Disagree (3)	4-Mostly Agree (4)	5-Fully agree (5)	Other - Do not have the expertise to comment (6)
**Optimal:** No/minimal maintenance. (1)	○	○	○	○	○	○
**Minimal:** Same as Optimal. (2)	○	○	○	○	○	○

Display This Question:

If Please select the newborn technology (ies) that you would like to provide feedback on. (Please Se... = Flow Splitter

Q5.23 22. Maintenance: Please provide reasoning if you have chosen 3 or below.

____

____

____

____

____

Display This Question:

If Please select the newborn technology (ies) that you would like to provide feedback on. (Please Se... = Flow Splitter

Q5.24 23. Instrument Pricing: Please rate your level of agreement with the statements under optimal and minimal. *Note: The optimal and minimal requirements define a range.*

	1-Disagree (1)	2-Somewhat Disagree (2)	3-Neither Agree nor Disagree (3)	4-Mostly Agree (4)	5-Fully agree (5)	Other - Do not have the expertise to comment (6)
**Optimal:** (1)	o	o	o	o	o	o
**Minimal:** (2)	o	o	o	o	o	o

Display This Question:

If Please select the newborn technology (ies) that you would like to provide feedback on. (Please Se... = Flow Splitter

Q5.25 24. Instrument Pricing: Please provide reasoning if you have chosen 3 or below.

____

____

____

____

____

End of Block: Flow Splitter

Start of Block: Suction Pump

Display This Question:

If Please select the newborn technology (ies) that you would like to provide feedback on. (Please Se... = Suction Pump

Q6.1 Suction Pump

Display This Question:

If Please select the newborn technology (ies) that you would like to provide feedback on. (Please Se... = Suction Pump

Q6.2 1. Intended Use: Please rate your level of agreement with the statements under optimal and minimal. *Note: The optimal and minimal requirements define a range.*

	1-Disagree (1)	2-Somewhat Disagree (2)	3-Neither Agree nor Disagree (3)	4-Mostly Agree (4)	5-Fully agree (5)	Other - Do not have the expertise to comment (6)
**Optimal:** Aspiration and removal of secretions, bodily fluids and foreign objects from a patient's airway or respiratory support system in the nasal, pharyngeal and tracheal areas. (1)	○	○	○	○	○	○
**Minimal:** Same as Optimal. (2)	○	○	○	○	○	○

Display This Question:

If Please select the newborn technology (ies) that you would like to provide feedback on. (Please Se... = Suction Pump

Q6.3 2. Intended Use: Please provide reasoning if you have chosen 3 or below.

____

____

____

____

____

Display This Question:

If Please select the newborn technology (ies) that you would like to provide feedback on. (Please Se... = Suction Pump

Q6.4 3. Target Operator: Please rate your level of agreement with the statements under optimal and minimal. *Note: The optimal and minimal requirements define a range.*

	1-Disagree (1)	2-Somewhat Disagree (2)	3-Neither agree nor disagree (3)	4-Mostly Agre (4)	5-Fully Agree (5)	Other - Do not have the expertise to comment (6)
**Optimal:** For use in low- and middle-income countries by a wide variety of clinicians, including nurses, clinical officers, and pediatricians. (1)	○	○	○	○	○	○
**Minimal:** Same as Optimal (2)	○	○	○	○	○	○

Display This Question:

If Please select the newborn technology (ies) that you would like to provide feedback on. (Please Se... = Suction Pump

Q6.5 4. Target Operator: Please provide reasoning if you have chosen 3 or below.

____

Display This Question:

If Please select the newborn technology (ies) that you would like to provide feedback on. (Please Se... = Suction Pump

Q6.6 5. Target Population: Please rate your level of agreement with the statements under optimal and minimal. *Note: The optimal and minimal requirements define a range.*

	1-Disagree (1)	2- Somewhat Disagree (2)	3-Neither Agree nor Disagree (3)	4-Mostly Agree (4)	5-Fully agree (5)	Other - Do not have the expertise to comment (6)
**Optimal:** Neonates ((1)	○	○	○	○	○	○
**Minimal:** Same as Optimal. (2)	○	○	○	○	○	○

Display This Question:

If Please select the newborn technology (ies) that you would like to provide feedback on. (Please Se... = Suction Pump

Q6.7 6. Target Population: Please provide reasoning if you have chosen 3 or below.

____

Display This Question:

If Please select the newborn technology (ies) that you would like to provide feedback on. (Please Se... = Suction Pump

Q6.8 7. Target Setting: Please rate your level of agreement with the statements under optimal and minimal. *Note: The optimal and minimal requirements define a range.*

	1-Disagree (1)	2-Somewhat Disagree (2)	3-Neither Agree nor Disagree (3)	4-Mostly Agree (4)	5-Fully agree (5)	Other - Do not have the expertise to comment (6)
**Optimal:** Hospitals in low-resource settings (1)	○	○	○	○	○	○
**Minimal:** Same as Optimal. (2)	○	○	○	○	○	○

Display This Question:

If Please select the newborn technology (ies) that you would like to provide feedback on. (Please Se... = Suction Pump

Q6.9 8. Target Setting: Please provide reasoning if you have chosen 3 or below.

____

____

____

____

____

Display This Question:

If Please select the newborn technology (ies) that you would like to provide feedback on. (Please Se... = Suction Pump

Q6.10 9. International Standard: Please rate your level of agreement with the statements under optimal and minimal. *Note: The optimal and minimal requirements define a range.*

	1-Disagree (1)	2-Somewhat Disagree (2)	3-Neither Agree nor Disagree (3)	4-Mostly Agree (4)	5-Fully agree (5)	Other - Do not have the expertise to comment (6)
**Optimal:** ISO 13485:2016 Medical devices – Quality management systems -- Requirements for regulatory purposes. (1)	○	○	○	○	○	○
**Minimal:** Same as Optimal. (2)	○	○	○	○	○	○

Display This Question:

If Please select the newborn technology (ies) that you would like to provide feedback on. (Please Se... = Suction Pump

Q6.11 10. International Standard: Please provide reasoning if you have chosen 3 or below.

____

____

____

____

____

Display This Question:

If Please select the newborn technology (ies) that you would like to provide feedback on. (Please Se... = Suction Pump

Q6.12 11. Regulation: Please rate your level of agreement with the statements under optimal and minimal. *Note: The optimal and minimal requirements define a range.*

	1-Disagree (1)	2-Somewhat Disagree (2)	3-Neither Agree nor Disagree (3)	4-Mostly Agree (4)	5-Fully agree (5)	Other - Do not have the expertise to comment (6)
**Optimal:** CE marking or US FDA Clearance (1)	○	○	○	○	○	○
**Minimal:** Same as Optimal. (2)	○	○	○	○	○	○

Display This Question:

If Please select the newborn technology (ies) that you would like to provide feedback on. (Please Se... = Suction Pump

Q6.13 12. Regulation: Please provide reasoning if you have chosen 3 or below.

____

____

____

____

____

Display This Question:

If Please select the newborn technology (ies) that you would like to provide feedback on. (Please Se... = Suction Pump

Q6.14 13. Pressure: Please rate your level of agreement with the statements under optimal and minimal. *Note: The optimal and minimal requirements define a range.*

	1-Disagree (1)	2-Somewhat Disagree (2)	3-Neither Agree nor Disagree (3)	4-Mostly Agree (4)	5-Fully agree (5)	Other - Do not have the expertise to comment (6)
**Optimal:** 60-100 mm Hg with continuous adjustment (1)	○	○	○	○	○	○
**Minimal:** 60-100 mm Hg (2)	○	○	○	○	○	○

Display This Question:

If Please select the newborn technology (ies) that you would like to provide feedback on. (Please Se... = Suction Pump

Q6.15 14. Pressure: Please provide reasoning if you have chosen 3 or below.

____

____

____

____

____

Display This Question:

If Please select the newborn technology (ies) that you would like to provide feedback on. (Please Se... = Suction Pump

Q6.16 15. Bottle Capacity: Please rate your level of agreement with the statements under optimal and minimal. *Note: The optimal and minimal requirements define a range.*

	1-Disagree (1)	2-Somewhat Disagree (2)	3-Neither Agree nor Disagree (3)	4-Mostly Agree (4)	5-Fully agree (5)	Other - Do not have the expertise to comment (6)
**Optimal:** 1 L (1)	○	○	○	○	○	○
**Minimal:** Same as Optimal. (2)	○	○	○	○	○	○

Display This Question:

If Please select the newborn technology (ies) that you would like to provide feedback on. (Please Se... = Suction Pump

Q6.17 16. Bottle Capacity: Please provide reasoning if you have chosen 3 or below.

____

____

____

____

____

Display This Question:

If Please select the newborn technology (ies) that you would like to provide feedback on. (Please Se... = Suction Pump

Q6.18 17. Longevity: Please rate your level of agreement with the statements under optimal and minimal. *Note: The optimal and minimal requirements define a range.*

	1-Disagree (1)	2-Somewhat Disagree (2)	3-Neither Agree nor Disagree (3)	4-Mostly Agree (4)	5-Fully agree (5)	Other - Do not have the expertise to comment (6)
**Optimal:** Mechanism to prevent aspirated liquids from reaching pump. (1)	o	o	o	o	o	o
**Minimal:** Same as Optimal. (2)	o	o	o	o	o	o

Display This Question:

If Please select the newborn technology (ies) that you would like to provide feedback on. (Please Se... = Suction Pump

Q6.19 18. Longevity: Please provide reasoning if you have chosen 3 or below.

____

____

____

____

____

Display This Question:

If Please select the newborn technology (ies) that you would like to provide feedback on. (Please Se... = Suction Pump

Q6.20 19. Noise Level: Please rate your level of agreement with the statements under optimal and minimal. *Note: The optimal and minimal requirements define a range.*

	1-Disagree (1)	2-Somewhat Disagree (2)	3-Neither Agree nor Disagree (3)	4-Mostly Agree (4)	5-Fully agree (5)	Other - Do not have the expertise to comment (6)
**Optimal:** (1)	○	○	○	○	○	○
**Minimal:** 65 dB (2)	○	○	○	○	○	○

Display This Question:

If Please select the newborn technology (ies) that you would like to provide feedback on. (Please Se... = Suction Pump

Q6.21 20. Noise Level: Please provide reasoning if you have chosen 3 or below.

____

____

____

____

____

Display This Question:

If Please select the newborn technology (ies) that you would like to provide feedback on. (Please Se... = Suction Pump

Q6.22 21. Cleaning: Please rate your level of agreement with the statements under optimal and minimal. *Note: The optimal and minimal requirements define a range.*

	1-Disagree (1)	2-Somewhat Disagree (2)	3-Neither Agree nor Disagree (3)	4-Mostly Agree (4)	5-Fully agree (5)	Other - Do not have the expertise to comment (6)
**Optimal:** Collection vessel easy to clean reusable. (1)	○	○	○	○	○	○
**Minimal:** Same as Optimal. (2)	○	○	○	○	○	○

Display This Question:

If Please select the newborn technology (ies) that you would like to provide feedback on. (Please Se... = Suction Pump

Q6.23 22. Cleaning: Please provide reasoning if you have chosen 3 or below.

____

____

____

____

____

Display This Question:

If Please select the newborn technology (ies) that you would like to provide feedback on. (Please Se... = Suction Pump

Q6.24 23. Maintenance: Please rate your level of agreement with the statements under optimal and minimal. *Note: The optimal and minimal requirements define a range.*

	1-Disagree (1)	2-Somewhat Disagree (2)	3-Neither Agree nor Disagree (3)	4-Mostly Agree (4)	5-Fully agree (5)	Other - Do not have the expertise to comment (6)
**Optimal:** No maintenance or lubrication. (1)	o	o	o	o	o	o
**Minimal:** Same as Optimal. (2)	o	o	o	o	o	o

Display This Question:

If Please select the newborn technology (ies) that you would like to provide feedback on. (Please Se... = Suction Pump

Q6.25 24. Maintenance: Please provide reasoning if you have chosen 3 or below.

____

____

____

____

____

Display This Question:

If Please select the newborn technology (ies) that you would like to provide feedback on. (Please Se... = Suction Pump

Q6.26 25. Operation Mode: Please rate your level of agreement with the statements under optimal and minimal. *Note: The optimal and minimal requirements define a range.*

	1-Disagree (1)	2-Somewhat Disagree (2)	3-Neither Agree nor Disagree (3)	4-Mostly Agree (4)	5-Fully agree (5)	Other - Do not have the expertise to comment (6)
**Optimal:** Adjustable to neonatal setting (60-100 mm Hg) (1)	o	o	o	o	o	o
**Minimal:** Same as Optimal. (2)	o	o	o	o	o	o

Display This Question:

If Please select the newborn technology (ies) that you would like to provide feedback on. (Please Se... = Suction Pump

Q6.27 26. Operation Mode: Please provide reasoning if you have chosen 3 or below.

____

____

____

____

____

Display This Question:

If Please select the newborn technology (ies) that you would like to provide feedback on. (Please Se... = Suction Pump

Q6.28 27. User Manual: Please rate your level of agreement with the statements under optimal and minimal. *Note: The optimal and minimal requirements define a range.*

	1-Disagree (1)	2-Somewhat Disagree (2)	3-Neither Agree nor Disagree (3)	4-Mostly Agree (4)	5-Fully agree (5)	Other - Do not have the expertise to comment (6)
**Optimal:** User manual and additional training materials (checklists, videos, guides) in English and local language. Attached to device with labels and markings where possible. (1)	○	○	○	○	○	○
**Minimal:** User manual provided. (2)	○	○	○	○	○	○

Display This Question:

If Please select the newborn technology (ies) that you would like to provide feedback on. (Please Se... = Suction Pump

Q6.29 28. User Manual: Please provide reasoning if you have chosen 3 or below.

____

____

____

____

____

Display This Question:

If Please select the newborn technology (ies) that you would like to provide feedback on. (Please Se... = Suction Pump

Q6.30 29. Voltage: Please rate your level of agreement with the statements under optimal and minimal. *Note: The optimal and minimal requirements define a range.*

	1-Disagree (1)	2-Somewhat Disagree (2)	3-Neither Agree nor Disagree (3)	4-Mostly Agree (4)	5-Fully agree (5)	Other - Do not have the expertise to comment (6)
**Optimal:** 110-240V 50-60hz (1)	○	○	○	○	○	○
**Minimal:** 220-240V 50-60hz (2)	○	○	○	○	○	○

Display This Question:

If Please select the newborn technology (ies) that you would like to provide feedback on. (Please Se... = Suction Pump

Q6.31 30. Voltage: Please provide reasoning if you have chosen 3 or below.

____

____

____

____

____

Display This Question:

If Please select the newborn technology (ies) that you would like to provide feedback on. (Please Se... = Suction Pump

Q6.32 31. Warranty: Please rate your level of agreement with the statements under optimal and minimal. *Note: The optimal and minimal requirements define a range.*

	1-Disagree (1)	2-Somewhat Disagree (2)	3-Neither Agree nor Disagree (3)	4-Mostly Agree (4)	5-Fully agree (5)	Other - Do not have the expertise to comment (6)
**Optimal:** 5 years (1)	o	o	o	o	o	o
**Minimal:** 1 year (2)	o	o	o	o	o	o

Display This Question:

If Please select the newborn technology (ies) that you would like to provide feedback on. (Please Se... = Suction Pump

Q6.33 32. Warranty: Please provide reasoning if you have chosen 3 or below.

____

____

____

____

____

Display This Question:

If Please select the newborn technology (ies) that you would like to provide feedback on. (Please Se... = Suction Pump

Q6.34 33. Instrument Pricing: Please rate your level of agreement with the statements under optimal and minimal. *Note: The optimal and minimal requirements define a range.*

	1-Disagree (1)	2-Somewhat Disagree (2)	3-Neither Agree nor Disagree (3)	4-Mostly Agree (4)	5-Fully agree (5)	Other - Do not have the expertise to comment (6)
**Optimal:** (1)	○	○	○	○	○	○
**Minimal:** (2)	○	○	○	○	○	○

Display This Question:

If Please select the newborn technology (ies) that you would like to provide feedback on. (Please Se... = Suction Pump

Q6.35 34. Instrument Pricing: Please provide reasoning if you have chosen 3 or below.

____

____

____

____

____

End of Block: Suction Pump

Start of Block: Respiratory Rate Monitor

Display This Question:

If Please select the newborn technology (ies) that you would like to provide feedback on. (Please Se... = Respiratory Rate Monitors

Q7.1 Respiratory Rate Monitor

Display This Question:

If Please select the newborn technology (ies) that you would like to provide feedback on. (Please Se... = Respiratory Rate Monitors

Q7.2 1. Intended Use: Please rate your level of agreement with the statements under optimal and minimal. *Note: The optimal and minimal requirements define a range.*

	1-Disagree (1)	2-Somewhat Disagree (2)	3-Neither Agree nor Disagree (3)	4-Mostly Agree (4)	5-Fully agree (5)	Other - Do not have the expertise to comment (6)
**Optimal:** To provide continuous monitoring of respiratory rate. (1)	○	○	○	○	○	○
**Minimal:** Same as Optimal. (2)	○	○	○	○	○	○

Display This Question:

If Please select the newborn technology (ies) that you would like to provide feedback on. (Please Se... = Respiratory Rate Monitors

Q7.3 2. Intended Use: Please provide reasoning if you have chosen 3 or below.

____

____

____

____

____

Display This Question:

If Please select the newborn technology (ies) that you would like to provide feedback on. (Please Se... = Respiratory Rate Monitors

Q7.4 3. Target Operator: Please rate your level of agreement with the statements under optimal and minimal. *Note: The optimal and minimal requirements define a range.*

	1-Disagree (1)	2-Somewhat Disagree (2)	3-Neither agree nor disagree (3)	4-Mostly Agre (4)	5-Fully Agree (5)	Other - Do not have the expertise to comment (6)
**Optimal:** For use in low- and middle-income countries by a wide variety of clinicians, including nurses, clinical officers, and pediatricians. (1)	○	○	○	○	○	○
**Minimal:** Same as Optimal (2)	○	○	○	○	○	○

Display This Question:

If Please select the newborn technology (ies) that you would like to provide feedback on. (Please Se... = Respiratory Rate Monitors

Q7.5 4. Target Operator: Please provide reasoning if you have chosen 3 or below.

____

Display This Question:

If Please select the newborn technology (ies) that you would like to provide feedback on. (Please Se... = Respiratory Rate Monitors

Q7.6 5. Target Population: Please rate your level of agreement with the statements under optimal and minimal. *Note: The optimal and minimal requirements define a range.*

	1-Disagree (1)	2-Somewhat Disagree (2)	3-Neither Agree nor Disagree (3)	4-Mostly Agree (4)	5-Fully agree (5)	Other - Do not have the expertise to comment (6)
**Optimal:** Neonates ((1)	○	○	○	○	○	○
**Minimal:** Same as Optimal. (2)	○	○	○	○	○	○

Display This Question:

If Please select the newborn technology (ies) that you would like to provide feedback on. (Please Se... = Respiratory Rate Monitors

Q7.7 6. Target Population: Please provide reasoning if you have chosen 3 or below.

____

Display This Question:

If Please select the newborn technology (ies) that you would like to provide feedback on. (Please Se... = Respiratory Rate Monitors

Q7.8 7. Target Setting: Please rate your level of agreement with the statements under optimal and minimal. *Note: The optimal and minimal requirements define a range.*

	1-Disagree (1)	2-Somewhat Disagree (2)	3-Neither Agree nor Disagree (3)	4-Mostly Agree (4)	5-Fully agree (5)	Other - Do not have the expertise to comment (6)
**Optimal:** Hospitals in low-resource settings (1)	o	o	o	o	o	o
**Minimal:** Same as Optimal. (2)	o	o	o	o	o	o

Display This Question:

If Please select the newborn technology (ies) that you would like to provide feedback on. (Please Se... = Respiratory Rate Monitors

Q7.9 8. Target Setting: Please provide reasoning if you have chosen 3 or below.

____

____

____

____

____

Display This Question:

If Please select the newborn technology (ies) that you would like to provide feedback on. (Please Se... = Respiratory Rate Monitors

Q7.10 9. International Standard: Please rate your level of agreement with the statements under optimal and minimal. *Note: The optimal and minimal requirements define a range.*

	1-Disagree (1)	2-Somewhat Disagree (2)	3-Neither Agree nor Disagree (3)	4-Mostly Agree (4)	5-Fully agree (5)	Other - Do not have the expertise to comment (6)
**Optimal:** ISO 13485:2016 Medical devices – Quality management systems -- Requirements for regulatory purposes. (1)	○	○	○	○	○	○
**Minimal:** Same as Optimal. (2)	○	○	○	○	○	○

Display This Question:

If Please select the newborn technology (ies) that you would like to provide feedback on. (Please Se... = Respiratory Rate Monitors

Q7.11 10. International Standard: Please provide reasoning if you have chosen 3 or below.

____

____

____

____

____

Display This Question:

If Please select the newborn technology (ies) that you would like to provide feedback on. (Please Se... = Respiratory Rate Monitors

Q7.12 11. Regulation: Please rate your level of agreement with the statements under optimal and minimal. *Note: The optimal and minimal requirements define a range.*

	1-Disagree (1)	2-Somewhat Disagree (2)	3-Neither Agree nor Disagree (3)	4-Mostly Agree (4)	5-Fully agree (5)	Other - Do not have the expertise to comment (6)
**Optimal:** CE marking or US FDA Clearance (1)	○	○	○	○	○	○
**Minimal:** Same as Optimal. (2)	○	○	○	○	○	○

Display This Question:

If Please select the newborn technology (ies) that you would like to provide feedback on. (Please Se... = Respiratory Rate Monitors

Q7.13 12. Regulation: Please provide reasoning if you have chosen 3 or below.

____

____

____

____

____

Display This Question:

If Please select the newborn technology (ies) that you would like to provide feedback on. (Please Se... = Respiratory Rate Monitors

Q7.14 13. Apnea Detection: Please rate your level of agreement with the statements under optimal and minimal. *Note: The optimal and minimal requirements define a range.*

	1-Disagree (1)	2-Somewhat Disagree (2)	3-Neither Agree nor Disagree (3)	4-Mostly Agree (4)	5-Fully agree (5)	Other - Do not have the expertise to comment (6)
**Optimal:** Detect periods of central apnea exceeding 20s duration. (1)	o	o	o	o	o	o
**Minimal:** None. (2)	o	o	o	o	o	o

Display This Question:

If Please select the newborn technology (ies) that you would like to provide feedback on. (Please Se... = Respiratory Rate Monitors

Q7.15 14. Apnea Detection: Please provide reasoning if you have chosen 3 or below.

____

____

____

____

____

Display This Question:

If Please select the newborn technology (ies) that you would like to provide feedback on. (Please Se... = Respiratory Rate Monitors

Q7.16 15. Respiratory Rate Accuracy: Please rate your level of agreement with the statements under optimal and minimal. *Note: The optimal and minimal requirements define a range.*

	1-Disagree (1)	2-Somewhat Disagree (2)	3-Neither Agree nor Disagree (3)	4-Mostly Agree (4)	5-Fully agree (5)	Other - Do not have the expertise to comment (6)
**Optimal:** +- 2 bpm (1)	○	○	○	○	○	○
**Minimal:** +- 10 bpm (2)	○	○	○	○	○	○

Display This Question:

If Please select the newborn technology (ies) that you would like to provide feedback on. (Please Se... = Respiratory Rate Monitors

Q7.17 16. Respiratory Rate Accuracy: Please provide reasoning if you have chosen 3 or below.

____

____

____

____

____

Display This Question:

If Please select the newborn technology (ies) that you would like to provide feedback on. (Please Se... = Respiratory Rate Monitors

Q7.18 17. Respiratory Rate Range: Please rate your level of agreement with the statements under optimal and minimal. *Note: The optimal and minimal requirements define a range.*

	1-Disagree (1)	2-Somewhat Disagree (2)	3-Neither Agree nor Disagree (3)	4-Mostly Agree (4)	5-Fully agree (5)	Other - Do not have the expertise to comment (6)
**Optimal:** 0-120 bpm (1)	○	○	○	○	○	○
**Minimal:** 0-100 bpm (2)	○	○	○	○	○	○

Display This Question:

If Please select the newborn technology (ies) that you would like to provide feedback on. (Please Se... = Respiratory Rate Monitors

Q7.19 18. Respiratory Rate Range: Please provide reasoning if you have chosen 3 or below.

____

____

____

____

____

Display This Question:

If Please select the newborn technology (ies) that you would like to provide feedback on. (Please Se... = Respiratory Rate Monitors

Q7.20 19. Respiratory Rate Resolution: Please rate your level of agreement with the statements under optimal and minimal. *Note: The optimal and minimal requirements define a range.*

	1-Disagree (1)	2-Somewhat Disagree (2)	3-Neither Agree nor Disagree (3)	4-Mostly Agree (4)	5-Fully agree (5)	Other - Do not have the expertise to comment (6)
**Optimal:** 1 bpm (1)	○	○	○	○	○	○
**Minimal:** 2 bpm (2)	○	○	○	○	○	○

Display This Question:

If Please select the newborn technology (ies) that you would like to provide feedback on. (Please Se... = Respiratory Rate Monitors

Q7.21 20. Respiratory Rate Resolution: Please provide reasoning if you have chosen 3 or below.

____

____

____

____

____

Display This Question:

If Please select the newborn technology (ies) that you would like to provide feedback on. (Please Se... = Respiratory Rate Monitors

Q7.22 21. Alarm: Please rate your level of agreement with the statements under optimal and minimal. *Note: The optimal and minimal requirements define a range.*

	1-Disagree (1)	2-Somewhat Disagree (2)	3-Neither Agree nor Disagree (3)	4-Mostly Agree (4)	5-Fully agree (5)	Other - Do not have the expertise to comment (6)
**Optimal:** Visual and auditory (1)	○	○	○	○	○	○
**Minimal:** Visual only (2)	○	○	○	○	○	○

Display This Question:

If Please select the newborn technology (ies) that you would like to provide feedback on. (Please Se... = Respiratory Rate Monitors

Q7.23 22. Alarm: Please provide reasoning if you have chosen 3 or below.

____

____

____

____

____

Display This Question:

If Please select the newborn technology (ies) that you would like to provide feedback on. (Please Se... = Respiratory Rate Monitors

Q7.24 23. Apnea Alarm Limits: Please rate your level of agreement with the statements under optimal and minimal. *Note: The optimal and minimal requirements define a range.*

	1-Disagree (1)	2-Somewhat Disagree (2)	3-Neither Agree nor Disagree (3)	4-Mostly Agree (4)	5-Fully agree (5)	Other - Do not have the expertise to comment (6)
**Optimal:** Adjustable (1)	o	o	o	o	o	o
**Minimal:** None (2)	o	o	o	o	o	o

Display This Question:

If Please select the newborn technology (ies) that you would like to provide feedback on. (Please Se... = Respiratory Rate Monitors

Q7.25 24. Apnea Alarm Limits: Please provide reasoning if you have chosen 3 or below.

____

____

____

____

____

Display This Question:

If Please select the newborn technology (ies) that you would like to provide feedback on. (Please Se... = Respiratory Rate Monitors

Q7.26 25. Consumables: Please rate your level of agreement with the statements under optimal and minimal. *Note: The optimal and minimal requirements define a range.*

	1-Disagree (1)	2-Somewhat Disagree (2)	3-Neither Agree nor Disagree (3)	4-Mostly Agree (4)	5-Fully agree (5)	Other - Do not have the expertise to comment (6)
**Optimal:** >12 months before required (1)	o	o	o	o	o	o
**Minimal:** >6 months before required (2)	o	o	o	o	o	o

Display This Question:

If Please select the newborn technology (ies) that you would like to provide feedback on. (Please Se... = Respiratory Rate Monitors

Q7.27 26. Consumables: Please provide reasoning if you have chosen 3 or below.

____

____

____

____

____

Display This Question:

If Please select the newborn technology (ies) that you would like to provide feedback on. (Please Se... = Respiratory Rate Monitors

Q7.28 27. Decontamination: Please rate your level of agreement with the statements under optimal and minimal. *Note: The optimal and minimal requirements define a range.*

	1-Disagree (1)	2-Somewhat Disagree (2)	3-Neither Agree nor Disagree (3)	4-Mostly Agree (4)	5-Fully agree (5)	Other - Do not have the expertise to comment (6)
**Optimal:** Easy to clean with common disinfecting agents (1)	o	o	o	o	o	o
**Minimal:** Same as Optimal. (2)	o	o	o	o	o	o

Display This Question:

If Please select the newborn technology (ies) that you would like to provide feedback on. (Please Se... = Respiratory Rate Monitors

Q7.29 27. Decontamination: Please provide reasoning if you have chosen 3 or below.

____

____

____

____

____

Display This Question:

If Please select the newborn technology (ies) that you would like to provide feedback on. (Please Se... = Respiratory Rate Monitors

Q7.30 28. User Manual: Please rate your level of agreement with the statements under optimal and minimal. *Note: The optimal and minimal requirements define a range.*

	1-Disagree (1)	2-Somewhat Disagree (2)	3-Neither Agree nor Disagree (3)	4-Mostly Agree (4)	5-Fully agree (5)	Other - Do not have the expertise to comment (6)
**Optimal:** User manual and additional training materials (checklists, videos, guides) in English and local language. Attached to device with labels and markings where possible. (1)	○	○	○	○	○	○
**Minimal:** User manual provided. (2)	○	○	○	○	○	○

Display This Question:

If Please select the newborn technology (ies) that you would like to provide feedback on. (Please Se... = Respiratory Rate Monitors

Q7.31 29. User Manual: Please provide reasoning if you have chosen 3 or below.

____

____

____

____

____

Display This Question:

If Please select the newborn technology (ies) that you would like to provide feedback on. (Please Se... = Respiratory Rate Monitors

Q7.32 30. Voltage: Please rate your level of agreement with the statements under optimal and minimal. *Note: The optimal and minimal requirements define a range.*

	1-Disagree (1)	2-Somewhat Disagree (2)	3-Neither Agree nor Disagree (3)	4-Mostly Agree (4)	5-Fully agree (5)	Other - Do not have the expertise to comment (6)
**Optimal:** 110-240V 50-60hz (1)	○	○	○	○	○	○
**Minimal:** 220-240V 50-60hz (2)	○	○	○	○	○	○

Display This Question:

If Please select the newborn technology (ies) that you would like to provide feedback on. (Please Se... = Respiratory Rate Monitors

Q7.33 31. Voltage: Please provide reasoning if you have chosen 3 or below.

____

____

____

____

____

Display This Question:

If Please select the newborn technology (ies) that you would like to provide feedback on. (Please Se... = Respiratory Rate Monitors

Q7.34 32. Battery Powered: Please rate your level of agreement with the statements under optimal and minimal. *Note: The optimal and minimal requirements define a range.*

	1-Disagree (1)	2-Somewhat Disagree (2)	3-Neither Agree nor Disagree (3)	4-Mostly Agree (4)	5-Fully agree (5)	Other - Do not have the expertise to comment (6)
**Optimal:** Yes, > 4 hr on a single charge (1)	○	○	○	○	○	○
**Minimal:** No (2)	○	○	○	○	○	○

Display This Question:

If Please select the newborn technology (ies) that you would like to provide feedback on. (Please Se... = Respiratory Rate Monitors

Q7.35 33. Battery Powered: Please provide reasoning if you have chosen 3 or below.

____

____

____

____

____

Display This Question:

If Please select the newborn technology (ies) that you would like to provide feedback on. (Please Se... = Respiratory Rate Monitors

Q7.36 34. Patient Interface: Please rate your level of agreement with the statements under optimal and minimal. *Note: The optimal and minimal requirements define a range.*

	1-Disagree (1)	2-Somewhat Disagree (2)	3-Neither Agree nor Disagree (3)	4-Mostly Agree (4)	5-Fully agree (5)	Other - Do not have the expertise to comment (6)
**Optimal:** Interface is biocompatible and reusable. (1)	o	o	o	o	o	o
**Minimal:** Interface is biocompatible. (2)	o	o	o	o	o	o

Display This Question:

If Please select the newborn technology (ies) that you would like to provide feedback on. (Please Se... = Respiratory Rate Monitors

Q7.37 35. Patient Interface: Please provide reasoning if you have chosen 3 or below.

____

____

____

____

____

Display This Question:

If Please select the newborn technology (ies) that you would like to provide feedback on. (Please Se... = Respiratory Rate Monitors

Q7.38 36. Respiratory Rate Alarm Limits: Please rate your level of agreement with the statements under optimal and minimal. *Note: The optimal and minimal requirements define a range.*

	1-Disagree (1)	2-Somewhat Disagree (2)	3-Neither Agree nor Disagree (3)	4-Mostly Agree (4)	5-Fully agree (5)	Other - Do not have the expertise to comment (6)
**Optimal:** Automatically adjust based on patient age (1)	o	o	o	o	o	o
**Minimal:** 30-60 bpm (2)	o	o	o	o	o	o

Display This Question:

If Please select the newborn technology (ies) that you would like to provide feedback on. (Please Se... = Respiratory Rate Monitors

Q7.39 38. Respiratory Rate Alarm Limits: Please provide reasoning if you have chosen 3 or below.

____

____

____

____

____

Display This Question:

If Please select the newborn technology (ies) that you would like to provide feedback on. (Please Se... = Respiratory Rate Monitors

Q7.40 39. Size: Please rate your level of agreement with the statements under optimal and minimal. *Note: The optimal and minimal requirements define a range.*

	1-Disagree (1)	2-Somewhat Disagree (2)	3-Neither Agree nor Disagree (3)	4-Mostly Agree (4)	5-Fully agree (5)	Other - Do not have the expertise to comment (6)
**Optimal:** Small footprint; can be left at bedside. (1)	○	○	○	○	○	○
**Minimal:** Same as Optimal. (2)	○	○	○	○	○	○

Display This Question:

If Please select the newborn technology (ies) that you would like to provide feedback on. (Please Se... = Respiratory Rate Monitors

Q7.41 40. Size: Please provide reasoning if you have chosen 3 or below.

____

____

____

____

____

Display This Question:

If Please select the newborn technology (ies) that you would like to provide feedback on. (Please Se... = Respiratory Rate Monitors

Q7.42 41. Weight: Please rate your level of agreement with the statements under optimal and minimal. *Note: The optimal and minimal requirements define a range.*

	1-Disagree (1)	2-Somewhat Disagree (2)	3-Neither Agree nor Disagree (3)	4-Mostly Agree (4)	5-Fully agree (5)	Other - Do not have the expertise to comment (6)
**Optimal:** < 500 g (1)	○	○	○	○	○	○
**Minimal:** Same as Optimal. (2)	○	○	○	○	○	○

Display This Question:

If Please select the newborn technology (ies) that you would like to provide feedback on. (Please Se... = Respiratory Rate Monitors

Q7.43 42. Weight: Please provide reasoning if you have chosen 3 or below.

____

____

____

____

____

Display This Question:

If Please select the newborn technology (ies) that you would like to provide feedback on. (Please Se... = Respiratory Rate Monitors

Q7.44 43. Apnea Intervention: Please rate your level of agreement with the statements under optimal and minimal. *Note: The optimal and minimal requirements define a range.*

	1-Disagree (1)	2-Somewhat Disagree (2)	3-Neither Agree nor Disagree (3)	4-Mostly Agree (4)	5-Fully agree (5)	Other - Do not have the expertise to comment (6)
**Optimal:** Yes (1)	○	○	○	○	○	○
**Minimal:** No (2)	○	○	○	○	○	○

Display This Question:

If Please select the newborn technology (ies) that you would like to provide feedback on. (Please Se... = Respiratory Rate Monitors

Q7.45 44. Apnea Intervention: Please provide reasoning if you have chosen 3 or below.

____

____

____

____

____

Display This Question:

If Please select the newborn technology (ies) that you would like to provide feedback on. (Please Se... = Respiratory Rate Monitors

Q7.46 45. Warranty: Please rate your level of agreement with the statements under optimal and minimal. *Note: The optimal and minimal requirements define a range.*

	1-Disagree (1)	2-Somewhat Disagree (2)	3-Neither Agree nor Disagree (3)	4-Mostly Agree (4)	5-Fully agree (5)	Other - Do not have the expertise to comment (6)
**Optimal:** 5 years (1)	○	○	○	○	○	○
**Minimal:** 1 year (2)	○	○	○	○	○	○

Display This Question:

If Please select the newborn technology (ies) that you would like to provide feedback on. (Please Se... = Respiratory Rate Monitors

Q7.47 46. Warranty: Please provide reasoning if you have chosen 3 or below.

____

____

____

____

____

Display This Question:

If Please select the newborn technology (ies) that you would like to provide feedback on. (Please Se... = Respiratory Rate Monitors

Q7.48 47. Instrument Pricing: Please rate your level of agreement with the statements under optimal and minimal. *Note: The optimal and minimal requirements define a range.*

	1-Disagree (1)	2-Somewhat Disagree (2)	3-Neither Agree nor Disagree (3)	4-Mostly Agree (4)	5-Fully agree (5)	Other - Do not have the expertise to comment (6)
**Optimal:** (1)	○	○	○	○	○	○
**Minimal:** (2)	○	○	○	○	○	○

Display This Question:

If Please select the newborn technology (ies) that you would like to provide feedback on. (Please Se... = Respiratory Rate Monitors

Q7.49 48. Instrument Pricing: Please provide reasoning if you have chosen 3 or below.

____

____

____

____

____

Display This Question:

If Please select the newborn technology (ies) that you would like to provide feedback on. (Please Se... = Respiratory Rate Monitors

Q7.50 49. Consumable Pricing: Please rate your level of agreement with the statements under optimal and minimal. *Note: The optimal and minimal requirements define a range.*

	1-Disagree (1)	2-Somewhat Disagree (2)	3-Neither Agree nor Disagree (3)	4-Mostly Agree (4)	5-Fully agree (5)	Other - Do not have the expertise to comment (6)
**Optimal:** (1)	o	o	o	o	o	o
**Minimal:** (2)	o	o	o	o	o	o

Display This Question:

If Please select the newborn technology (ies) that you would like to provide feedback on. (Please Se... = Respiratory Rate Monitors

Q7.51 50. Consumable Pricing: Please provide reasoning if you have chosen 3 or below.

____

____

____

____

____

End of Block: Respiratory Rate Monitor

Start of Block: Serum Bilirubin Test

Display This Question:

If Please select the newborn technology (ies) that you would like to provide feedback on. (Please Se... = Serum Bilirubin Test

Q8.1 Serum Bilirubin Test

Display This Question:

If Please select the newborn technology (ies) that you would like to provide feedback on. (Please Se... = Serum Bilirubin Test

Q8.2 1. Intended Use: Please rate your level of agreement with the statements under optimal and minimal. *Note: The optimal and minimal requirements define a range.*

	1-Disagree (1)	2-Somewhat Disagree (2)	3-Neither Agree nor Disagree (3)	4-Mostly Agree (4)	5-Fully agree (5)	Other - Do not have the expertise to comment (6)
**Optimal:** Quantification of total serum bilirubin in neonates for the diagnosis and management of jaundice at the patient's bedside (1)	o	o	o	o	o	o
**Minimal:** Same as Optimal. (2)	o	o	o	o	o	o

Display This Question:

If Please select the newborn technology (ies) that you would like to provide feedback on. (Please Se... = Serum Bilirubin Test

Q8.3 2. Intended Use: Please provide reasoning if you have chosen 3 or below.

____

____

____

____

____

Display This Question:

If Please select the newborn technology (ies) that you would like to provide feedback on. (Please Se... = Serum Bilirubin Test

Q8.4 3. Target Operator: Please rate your level of agreement with the statements under optimal and minimal. *Note: The optimal and minimal requirements define a range.*

	1-Disagree (1)	2-Somewhat Disagree (2)	3-Neither agree nor disagree (3)	4-Mostly Agre (4)	5-Fully Agree (5)	Other - Do not have the expertise to comment (6)
**Optimal:** For use in low- and middle-income countries by a wide variety of clinicians, including nurses, clinical officers, and pediatricians. (1)	○	○	○	○	○	○
**Minimal:** Same as Optimal (2)	○	○	○	○	○	○

Display This Question:

If Please select the newborn technology (ies) that you would like to provide feedback on. (Please Se... = Serum Bilirubin Test

Q8.5 4. Target Operator: Please provide reasoning if you have chosen 3 or below.

____

Display This Question:

If Please select the newborn technology (ies) that you would like to provide feedback on. (Please Se... = Serum Bilirubin Test

Q8.6 5. Target Population: Please rate your level of agreement with the statements under optimal and minimal. *Note: The optimal and minimal requirements define a range.*

	1-Disagree (1)	2-Somewhat Disagree (2)	3-Neither Agree nor Disagree (3)	4-Mostly Agree (4)	5-Fully agree (5)	Other - Do not have the expertise to comment (6)
**Optimal:** Neonates ((1)	○	○	○	○	○	○
**Minimal:** Same as Optimal. (2)	○	○	○	○	○	○

Display This Question:

If Please select the newborn technology (ies) that you would like to provide feedback on. (Please Se... = Serum Bilirubin Test

Q8.7 6. Target Population: Please provide reasoning if you have chosen 3 or below.

____

Display This Question:

If Please select the newborn technology (ies) that you would like to provide feedback on. (Please Se... = Serum Bilirubin Test

Q8.8 7. Target Setting: Please rate your level of agreement with the statements under optimal and minimal. *Note: The optimal and minimal requirements define a range.*

	1-Disagree (1)	2-Somewhat Disagree (2)	3-Neither Agree nor Disagree (3)	4-Mostly Agree (4)	5-Fully agree (5)	Other - Do not have the expertise to comment (6)
**Optimal:** Hospitals in low-resource settings (1)	o	o	o	o	o	o
**Minimal:** Same as Optimal. (2)	o	o	o	o	o	o

Display This Question:

If Please select the newborn technology (ies) that you would like to provide feedback on. (Please Se... = Serum Bilirubin Test

Q8.9 8. Target Setting: Please provide reasoning if you have chosen 3 or below.

____

____

____

____

____

Display This Question:

If Please select the newborn technology (ies) that you would like to provide feedback on. (Please Se... = Serum Bilirubin Test

Q8.10 9. International Standard: Please rate your level of agreement with the statements under optimal and minimal. *Note: The optimal and minimal requirements define a range.*

	1-Disagree (1)	2-Somewhat Disagree (2)	3-Neither Agree nor Disagree (3)	4-Mostly Agree (4)	5-Fully agree (5)	Other - Do not have the expertise to comment (6)
**Optimal:** ISO 13485:2016 Medical devices – Quality management systems -- Requirements for regulatory purposes. (1)	o	o	o	o	o	o
**Minimal:** Same as Optimal. (2)	o	o	o	o	o	o

Display This Question:

If Please select the newborn technology (ies) that you would like to provide feedback on. (Please Se... = Serum Bilirubin Test

Q8.11 10. International Standard: Please provide reasoning if you have chosen 3 or below.

____

____

____

____

____

Display This Question:

If Please select the newborn technology (ies) that you would like to provide feedback on. (Please Se... = Serum Bilirubin Test

Q8.12 11. Regulation: Please rate your level of agreement with the statements under optimal and minimal. *Note: The optimal and minimal requirements define a range.*

	1-Disagree (1)	2-Somewhat Disagree (2)	3-Neither Agree nor Disagree (3)	4-Mostly Agree (4)	5-Fully agree (5)	Other - Do not have the expertise to comment (6)
**Optimal:** CE marking or US FDA Clearance (1)	○	○	○	○	○	○
**Minimal:** Same as Optimal. (2)	○	○	○	○	○	○

Display This Question:

If Please select the newborn technology (ies) that you would like to provide feedback on. (Please Se... = Serum Bilirubin Test

Q8.13 12. Regulation: Please provide reasoning if you have chosen 3 or below.

____

____

____

____

____

Display This Question:

If Please select the newborn technology (ies) that you would like to provide feedback on. (Please Se... = Serum Bilirubin Test

Q8.14 13. Linear Range: Please rate your level of agreement with the statements under optimal and minimal. *Note: The optimal and minimal requirements define a range.*

	1-Disagree (1)	2-Somewhat Disagree (2)	3-Neither Agree nor Disagree (3)	4-Mostly Agree (4)	5-Fully agree (5)	Other - Do not have the expertise to comment (6)
**Optimal:** 0-40 mg/dL (1)	○	○	○	○	○	○
**Minimal:** 0-30 mg/dL (2)	○	○	○	○	○	○

Display This Question:

If Please select the newborn technology (ies) that you would like to provide feedback on. (Please Se... = Serum Bilirubin Test

Q8.15 14. Linear Range: Please provide reasoning if you have chosen 3 or below.

____

____

____

____

____

Display This Question:

If Please select the newborn technology (ies) that you would like to provide feedback on. (Please Se... = Serum Bilirubin Test

Q8.16 15. Accuracy: Please rate your level of agreement with the statements under optimal and minimal. *Note: The optimal and minimal requirements define a range.*

	1-Disagree (1)	2-Somewhat Disagree (2)	3-Neither Agree nor Disagree (3)	4-Mostly Agree (4)	5-Fully agree (5)	Other - Do not have the expertise to comment (6)
**Optimal:** Within 20% or 0.4 mg/dL, whichever is greater (1)	○	○	○	○	○	○
**Minimal:** Same as Optimal. (2)	○	○	○	○	○	○

Display This Question:

If Please select the newborn technology (ies) that you would like to provide feedback on. (Please Se... = Serum Bilirubin Test

Q8.17 16. Accuracy: Please provide reasoning if you have chosen 3 or below.

____

____

____

____

____

Display This Question:

If Please select the newborn technology (ies) that you would like to provide feedback on. (Please Se... = Serum Bilirubin Test

Q8.18 17. Result Format: Please rate your level of agreement with the statements under optimal and minimal. *Note: The optimal and minimal requirements define a range.*

	1-Disagree (1)	2-Somewhat Disagree (2)	3-Neither Agree nor Disagree (3)	4-Mostly Agree (4)	5-Fully agree (5)	Other - Do not have the expertise to comment (6)
**Optimal:** Quantitative across whole linear range (1)	o	o	o	o	o	o
**Minimal:** Quantitative; semi quantitative below 2 or above 20 mg/dL (2)	o	o	o	o	o	o

Display This Question:

If Please select the newborn technology (ies) that you would like to provide feedback on. (Please Se... = Serum Bilirubin Test

Q8.19 18. Result Format: Please provide reasoning if you have chosen 3 or below.

____

____

____

____

____

Display This Question:

If Please select the newborn technology (ies) that you would like to provide feedback on. (Please Se... = Serum Bilirubin Test

Q8.20 19. Result Units: Please rate your level of agreement with the statements under optimal and minimal. *Note: The optimal and minimal requirements define a range.*

	1-Disagree (1)	2-Somewhat Disagree (2)	3-Neither Agree nor Disagree (3)	4-Mostly Agree (4)	5-Fully agree (5)	Other - Do not have the expertise to comment (6)
**Optimal:** mg/dL and mmol/L (1)	○	○	○	○	○	○
**Minimal:** Same as Optimal. (2)	○	○	○	○	○	○

Display This Question:

If Please select the newborn technology (ies) that you would like to provide feedback on. (Please Se... = Serum Bilirubin Test

Q8.21 20. Result Units: Please provide reasoning if you have chosen 3 or below.

____

____

____

____

____

Display This Question:

If Please select the newborn technology (ies) that you would like to provide feedback on. (Please Se... = Serum Bilirubin Test

Q8.22 21. Precision: Please rate your level of agreement with the statements under optimal and minimal. *Note: The optimal and minimal requirements define a range.*

	1-Disagree (1)	2-Somewhat Disagree (2)	3-Neither Agree nor Disagree (3)	4-Mostly Agree (4)	5-Fully agree (5)	Other - Do not have the expertise to comment (6)
**Optimal:** 4% CV (1)	○	○	○	○	○	○
**Minimal:** 15% CV (2)	○	○	○	○	○	○

Display This Question:

If Please select the newborn technology (ies) that you would like to provide feedback on. (Please Se... = Serum Bilirubin Test

Q8.23 22. Precision: Please provide reasoning if you have chosen 3 or below.

____

____

____

____

____

Display This Question:

If Please select the newborn technology (ies) that you would like to provide feedback on. (Please Se... = Serum Bilirubin Test

Q8.24 23. Sample: Please rate your level of agreement with the statements under optimal and minimal. *Note: The optimal and minimal requirements define a range.*

	1-Disagree (1)	2-Somewhat Disagree (2)	3-Neither Agree nor Disagree (3)	4-Mostly Agree (4)	5-Fully agree (5)	Other - Do not have the expertise to comment (6)
**Optimal:** whole blood heel-stick sample (1)	○	○	○	○	○	○
**Minimal:** Same as Optimal. (2)	○	○	○	○	○	○

Display This Question:

If Please select the newborn technology (ies) that you would like to provide feedback on. (Please Se... = Serum Bilirubin Test

Q8.25 24. Sample: Please provide reasoning if you have chosen 3 or below.

____

____

____

____

____

Display This Question:

If Please select the newborn technology (ies) that you would like to provide feedback on. (Please Se... = Serum Bilirubin Test

Q8.26 25. Number of Steps: Please rate your level of agreement with the statements under optimal and minimal. *Note: The optimal and minimal requirements define a range.*

	1-Disagree (1)	2-Somewhat Disagree (2)	3-Neither Agree nor Disagree (3)	4-Mostly Agree (4)	5-Fully agree (5)	Other - Do not have the expertise to comment (6)
**Optimal:** No more than 1-4 steps (requiring operator intervention) (1)	○	○	○	○	○	○
**Minimal:** No more than 4-6 steps (requiring operator intervention) (2)	○	○	○	○	○	○

Display This Question:

If Please select the newborn technology (ies) that you would like to provide feedback on. (Please Se... = Serum Bilirubin Test

Q8.27 26. Number of Steps: Please provide reasoning if you have chosen 3 or below.

____

____

____

____

____

Display This Question:

If Please select the newborn technology (ies) that you would like to provide feedback on. (Please Se... = Serum Bilirubin Test

Q8.28 27. Calibration: Please rate your level of agreement with the statements under optimal and minimal. *Note: The optimal and minimal requirements define a range.*

	1-Disagree (1)	2-Somewhat Disagree (2)	3-Neither Agree nor Disagree (3)	4-Mostly Agree (4)	5-Fully agree (5)	Other - Do not have the expertise to comment (6)
**Optimal:** No calibration (1)	o	o	o	o	o	o
**Minimal:** Minimal user calibration required (2)	o	o	o	o	o	o

Display This Question:

If Please select the newborn technology (ies) that you would like to provide feedback on. (Please Se... = Serum Bilirubin Test

Q8.29 28. Calibration: Please provide reasoning if you have chosen 3 or below.

____

____

____

____

____

Display This Question:

If Please select the newborn technology (ies) that you would like to provide feedback on. (Please Se... = Serum Bilirubin Test

Q8.30 29. Kit Stability & Storage: Please rate your level of agreement with the statements under optimal and minimal. *Note: The optimal and minimal requirements define a range.*

	1-Disagree (1)	2-Somewhat Disagree (2)	3-Neither Agree nor Disagree (3)	4-Mostly Agree (4)	5-Fully agree (5)	Other - Do not have the expertise to comment (6)
**Optimal:** Stable for >12 months with harsh ambient conditions (temperature 5-45 °C, humidity 15% to 95%, dusty air, elevation >=2000 meters) and transport stress (48h with fluctuations up to 50°C and down to 0°C) (1)	o	o	o	o	o	o
**Minimal:** Stable for 12 months with harsh ambient conditions (temperature 10-40 °C, humidity 15%-95% elevation up to 2000 meters) and transport stress (48h with fluctuations up to 50°C and down to	o	o	o	o	o	o

0°C) (2)	

Display This Question:

If Please select the newborn technology (ies) that you would like to provide feedback on. (Please Se... = Serum Bilirubin Test

Q8.31 30. Kit Stability & Storage: Please provide reasoning if you have chosen 3 or below.

____

____

____

____

____

Display This Question:

If Please select the newborn technology (ies) that you would like to provide feedback on. (Please Se... = Serum Bilirubin Test

Q8.32 31. Equipment Required: Please rate your level of agreement with the statements under optimal and minimal. *Note: The optimal and minimal requirements define a range.*

	1-Disagree (1)	2-Somewhat Disagree (2)	3-Neither Agree nor Disagree (3)	4-Mostly Agree (4)	5-Fully agree (5)	Other - Do not have the expertise to comment (6)
**Optimal:** Small, portable or hand-held device; device-free/disposable preferred; does not require centrifuge (1)	o	o	o	o	o	o
**Minimal:** Small, table-top device; portable device optional; does not require centrifuge (2)	o	o	o	o	o	o

Display This Question:

If Please select the newborn technology (ies) that you would like to provide feedback on. (Please Se... = Serum Bilirubin Test

Q8.33 32. Equipment Required: Please provide reasoning if you have chosen 3 or below.

____

____

____

____

____

Display This Question:

If Please select the newborn technology (ies) that you would like to provide feedback on. (Please Se... = Serum Bilirubin Test

Q8.34 33. Power Requirement: Please rate your level of agreement with the statements under optimal and minimal. *Note: The optimal and minimal requirements define a range.*

	1-Disagree (1)	2-Somewhat Disagree (2)	3-Neither Agree nor Disagree (3)	4-Mostly Agree (4)	5-Fully agree (5)	Other - Do not have the expertise to comment (6)
**Optimal:** None (i.e. a disposable test that requires no electricity) (1)	o	o	o	o	o	o
**Minimal:** 110-220V AC current; DC power with rechargeable battery lasting up to 8 hours of testing (2)	o	o	o	o	o	o

---

Display This Question:

If Please select the newborn technology (ies) that you would like to provide feedback on. (Please Se... = Serum Bilirubin Test

Q8.35 34. Power Requirement: Please provide reasoning if you have chosen 3 or below.

____

____

____

____

____

Display This Question:

If Please select the newborn technology (ies) that you would like to provide feedback on. (Please Se... = Serum Bilirubin Test

Q8.36 35. Instrument Pricing: Please rate your level of agreement with the statements under optimal and minimal. *Note: The optimal and minimal requirements define a range.*

	1-Disagree (1)	2-Somewhat Disagree (2)	3-Neither Agree nor Disagree (3)	4-Mostly Agree (4)	5-Fully agree (5)	Other - Do not have the expertise to comment (6)
**Optimal:** (1)	o	o	o	o	o	o
**Minimal:** (2)	o	o	o	o	o	o

Display This Question:

If Please select the newborn technology (ies) that you would like to provide feedback on. (Please Se... = Serum Bilirubin Test

Q8.37 36. Instrument Pricing: Please provide reasoning if you have chosen 3 or below.

____

____

____

____

____

Display This Question:

If Please select the newborn technology (ies) that you would like to provide feedback on. (Please Se... = Serum Bilirubin Test

Q8.38 37. Consumable Pricing: Please rate your level of agreement with the statements under optimal and minimal. *Note: The optimal and minimal requirements define a range.*

	1-Disagree (1)	2-Somewhat Disagree (2)	3-Neither Agree nor Disagree (3)	4-Mostly Agree (4)	5-Fully agree (5)	Other - Do not have the expertise to comment (6)
**Optimal:** (1)	◯	◯	◯	◯	◯	◯
**Minimal:** $1.50 per test ex-works (2)	◯	◯	◯	◯	◯	◯

Display This Question:

If Please select the newborn technology (ies) that you would like to provide feedback on. (Please Se... = Serum Bilirubin Test

Q8.39 38. Consumable Pricing: Please provide reasoning if you have chosen 3 or below.

____

____

____

____

____

End of Block: Serum Bilirubin Test

Start of Block: Phototherapy Lights

Display This Question:

If Please select the newborn technology (ies) that you would like to provide feedback on. (Please Se... = Phototherapy Light

Q9.1 Phototherapy Lights

Display This Question:

If Please select the newborn technology (ies) that you would like to provide feedback on. (Please Se... = Phototherapy Light

Q9.2 1. Intended Use: Please rate your level of agreement with the statements under optimal and minimal. *Note: The optimal and minimal requirements define a range.*

	1-Disagree (1)	2-Somewhat Disagree (2)	3-Neither Agree nor Disagree (3)	4-Mostly Agree (4)	5-Fully agree (5)	Other - Do not have the expertise to comment (6)
**Optimal:** Treatment of hyperbilirubinemia in neonates. (1)	○	○	○	○	○	○
**Minimal:** Same as Optimal. (2)	○	○	○	○	○	○

Display This Question:

If Please select the newborn technology (ies) that you would like to provide feedback on. (Please Se... = Phototherapy Light

Q9.3 2. Intended Use: Please provide reasoning if you have chosen 3 or below.

____

____

____

____

____

Display This Question:

If Please select the newborn technology (ies) that you would like to provide feedback on. (Please Se... = Phototherapy Light

Q9.4 3. Target Operator: Please rate your level of agreement with the statements under optimal and minimal. *Note: The optimal and minimal requirements define a range.*

	1-Disagree (1)	2-Somewhat Disagree (2)	3-Neither agree nor disagree (3)	4-Mostly Agre (4)	5-Fully Agree (5)	Other - Do not have the expertise to comment (6)
**Optimal:** For use in low- and middle-income countries by a wide variety of clinicians, including nurses, clinical officers, and pediatricians. (1)	○	○	○	○	○	○
**Minimal:** Same as Optimal (2)	○	○	○	○	○	○

Display This Question:

If Please select the newborn technology (ies) that you would like to provide feedback on. (Please Se... = Phototherapy Light

Q9.5 4. Target Operator: Please provide reasoning if you have chosen 3 or below.

____

Display This Question:

If Please select the newborn technology (ies) that you would like to provide feedback on. (Please Se... = Phototherapy Light

Q9.6 5. Target Population: Please rate your level of agreement with the statements under optimal and minimal. *Note: The optimal and minimal requirements define a range.*

	1-Disagree (1)	2-Somewhat Disagree (2)	3-Neither Agree nor Disagree (3)	4-Mostly Agree (4)	5-Fully agree (5)	Other - Do not have the expertise to comment (6)
**Optimal:** Neonates ((1)	○	○	○	○	○	○
**Minimal:** Same as Optimal. (2)	○	○	○	○	○	○

Display This Question:

If Please select the newborn technology (ies) that you would like to provide feedback on. (Please Se... = Phototherapy Light

Q9.7 6. Target Population: Please provide reasoning if you have chosen 3 or below.

____

Display This Question:

If Please select the newborn technology (ies) that you would like to provide feedback on. (Please Se... = Phototherapy Light

Q9.8 7. Target Setting: Please rate your level of agreement with the statements under optimal and minimal. *Note: The optimal and minimal requirements define a range.*

	1-Disagree (1)	2-Somewhat Disagree (2)	3-Neither Agree nor Disagree (3)	4-Mostly Agree (4)	5-Fully agree (5)	Other - Do not have the expertise to comment (6)
**Optimal:** Hospitals in low-resource settings (1)	○	○	○	○	○	○
**Minimal:** Same as Optimal. (2)	○	○	○	○	○	○

Display This Question:

If Please select the newborn technology (ies) that you would like to provide feedback on. (Please Se... = Phototherapy Light

Q9.9 8. Target Setting: Please provide reasoning if you have chosen 3 or below.

____

____

____

____

____

Display This Question:

If Please select the newborn technology (ies) that you would like to provide feedback on. (Please Se... = Phototherapy Light

Q9.10 9. International Standard: Please rate your level of agreement with the statements under optimal and minimal. *Note: The optimal and minimal requirements define a range.*

	1-Disagree (1)	2-Somewhat Disagree (2)	3-Neither Agree nor Disagree (3)	4-Mostly Agree (4)	5-Fully agree (5)	Other - Do not have the expertise to comment (6)
**Optimal:** ISO 13485:2016 Medical devices – Quality management systems -- Requirements for regulatory purposes. (1)	○	○	○	○	○	○
**Minimal:** Same as Optimal. (2)	○	○	○	○	○	○

Display This Question:

If Please select the newborn technology (ies) that you would like to provide feedback on. (Please Se... = Phototherapy Light

Q9.11 10. International Standard: Please provide reasoning if you have chosen 3 or below.

____

____

____

____

____

Display This Question:

If Please select the newborn technology (ies) that you would like to provide feedback on. (Please Se... = Phototherapy Light

Q9.12 11. Regulation: Please rate your level of agreement with the statements under optimal and minimal. *Note: The optimal and minimal requirements define a range.*

	1-Disagree (1)	2-Somewhat Disagree (2)	3-Neither Agree nor Disagree (3)	4-Mostly Agree (4)	5-Fully agree (5)	Other - Do not have the expertise to comment (6)
**Optimal:** CE marking or US FDA Clearance (1)	o	o	o	o	o	o
**Minimal:** Same as Optimal (2)	o	o	o	o	o	o

Display This Question:

If Please select the newborn technology (ies) that you would like to provide feedback on. (Please Se... = Phototherapy Light

Q9.13 12. Regulation: Please provide reasoning if you have chosen 3 or below.

____

____

____

____

____

Display This Question:

If Please select the newborn technology (ies) that you would like to provide feedback on. (Please Se... = Phototherapy Light

Q9.14 13. Irradiance: Please rate your level of agreement with the statements under optimal and minimal. *Note: The optimal and minimal requirements define a range.*

	1-Disagree (1)	2-Somewhat Disagree (2)	3-Neither Agree nor Disagree (3)	4-Mostly Agree (4)	5-Fully agree (5)	Other - Do not have the expertise to comment (6)
**Optimal:** Standard Phototherapy: 8-10 uW/cm2/nm AND Intensive Phototherapy: >30 uW/cm2/nm (1)	○	○	○	○	○	○
**Minimal:** Same as Optimal (4)	○	○	○	○	○	○

Display This Question:

If Please select the newborn technology (ies) that you would like to provide feedback on. (Please Se... = Phototherapy Light

Q9.15 14. Irradiance: Please provide reasoning if you have chosen 3 or below.

____

____

____

____

____

Display This Question:

If Please select the newborn technology (ies) that you would like to provide feedback on. (Please Se... = Phototherapy Light

Q9.16 15. Effective Treatment Area: Please rate your level of agreement with the statements under optimal and minimal. *Note: The optimal and minimal requirements define a range.*

	1-Disagree (1)	2-Somewhat Disagree (2)	3-Neither Agree nor Disagree (3)	4-Mostly Agree (4)	5-Fully agree (5)	Other - Do not have the expertise to comment (6)
**Optimal:** >1300 cm2 (1)	○	○	○	○	○	○
**Minimal:** Same as Optimal. (2)	○	○	○	○	○	○

Display This Question:

If Please select the newborn technology (ies) that you would like to provide feedback on. (Please Se... = Phototherapy Light

Q9.17 16. Effective Treatment Area: Please provide reasoning if you have chosen 3 or below.

____

____

____

____

____

Display This Question:

If Please select the newborn technology (ies) that you would like to provide feedback on. (Please Se... = Phototherapy Light

Q9.18 17. Peak Wavelength Please rate your level of agreement with the statements under optimal and minimal. *Note: The optimal and minimal requirements define a range.*

	1-Disagree (1)	2-Somewhat Disagree (2)	3-Neither Agree nor Disagree (3)	4-Mostly Agree (4)	5-Fully agree (5)	Other - Do not have the expertise to comment (6)
**Optimal:** 430-490 nm (1)	o	o	o	o	o	o
**Minimal:** Same as Optimal. (2)	o	o	o	o	o	o

Display This Question:

If Please select the newborn technology (ies) that you would like to provide feedback on. (Please Se... = Phototherapy Light

Q9.19 18. Peak Wavelength: Please provide reasoning if you have chosen 3 or below.

____

____

____

____

____

Display This Question:

If Please select the newborn technology (ies) that you would like to provide feedback on. (Please Se... = Phototherapy Light

Q9.20 19. Light Source: Please rate your level of agreement with the statements under optimal and minimal. *Note: The optimal and minimal requirements define a range.*

	1-Disagree (1)	2-Somewhat Disagree (2)	3-Neither Agree nor Disagree (3)	4-Mostly Agree (4)	5-Fully agree (5)	Other - Do not have the expertise to comment (6)
**Optimal:** LED (1)	○	○	○	○	○	○
**Minimal:** Same as Optimal. (2)	○	○	○	○	○	○

Display This Question:

If Please select the newborn technology (ies) that you would like to provide feedback on. (Please Se... = Phototherapy Light

Q9.21 20. Light Source: Please provide reasoning if you have chosen 3 or below.

____

____

____

____

____

Display This Question:

If Please select the newborn technology (ies) that you would like to provide feedback on. (Please Se... = Phototherapy Light

Q9.22 21. Bulb Lifetime: Please rate your level of agreement with the statements under optimal and minimal. *Note: The optimal and minimal requirements define a range.*

	1-Disagree (1)	2-Somewhat Disagree (2)	3-Neither Agree nor Disagree (3)	4-Mostly Agree (4)	5-Fully agree (5)	Other - Do not have the expertise to comment (6)
**Optimal:** 60,000 hours (1)	○	○	○	○	○	○
**Minimal:** 44,000 hours (2)	○	○	○	○	○	○

Display This Question:

If Please select the newborn technology (ies) that you would like to provide feedback on. (Please Se... = Phototherapy Light

Q9.23 22. Bulb Lifetime: Please provide reasoning if you have chosen 3 or below.

____

____

____

____

____

Display This Question:

If Please select the newborn technology (ies) that you would like to provide feedback on. (Please Se... = Phototherapy Light

Q9.24 23. Ease of Replacing Bulbs: Please rate your level of agreement with the statements under optimal and minimal. *Note: The optimal and minimal requirements define a range.*

	1-Disagree (1)	2-Somewhat Disagree (2)	3-Neither Agree nor Disagree (3)	4-Mostly Agree (4)	5-Fully agree (5)	Other - Do not have the expertise to comment (6)
**Optimal:** Capable of being replaced by a technician with minimal training and basic tools (screwdrivers) (1)	o	o	o	o	o	o
**Minimal:** Same as Optimal. (2)	o	o	o	o	o	o

Display This Question:

If Please select the newborn technology (ies) that you would like to provide feedback on. (Please Se... = Phototherapy Light

Q9.25 24. Ease of Replacing Bulbs: Please provide reasoning if you have chosen 3 or below.

____

____

____

____

____

Display This Question:

If Please select the newborn technology (ies) that you would like to provide feedback on. (Please Se... = Phototherapy Light

Q9.26 25. Irradiance Meter: Please rate your level of agreement with the statements under optimal and minimal. *Note: The optimal and minimal requirements define a range.*

	1-Disagree (1)	2-Somewhat Disagree (2)	3-Neither Agree nor Disagree (3)	4-Mostly Agree (4)	5-Fully agree (5)	Other - Do not have the expertise to comment (6)
**Optimal:** Included (1)	○	○	○	○	○	○
**Minimal:** Same as Optimal. (2)	○	○	○	○	○	○

Display This Question:

If Please select the newborn technology (ies) that you would like to provide feedback on. (Please Se... = Phototherapy Light

Q9.27 26. Irradiance Meter: Please provide reasoning if you have chosen 3 or below.

____

____

____

____

____

Display This Question:

If Please select the newborn technology (ies) that you would like to provide feedback on. (Please Se... = Phototherapy Light

Q9.28 27. Voltage: Please rate your level of agreement with the statements under optimal and minimal. *Note: The optimal and minimal requirements define a range.*

	1-Disagree (1)	2-Somewhat Disagree (2)	3-Neither Agree nor Disagree (3)	4-Mostly Agree (4)	5-Fully agree (5)	Other - Do not have the expertise to comment (6)
**Optimal:** 110-240 50-60hz (1)	○	○	○	○	○	○
**Minimal:** 220-240 50-60hz (2)	○	○	○	○	○	○

Display This Question:

If Please select the newborn technology (ies) that you would like to provide feedback on. (Please Se... = Phototherapy Light

Q9.29 28. Voltage: Please provide reasoning if you have chosen 3 or below.

____

____

____

____

____

Display This Question:

If Please select the newborn technology (ies) that you would like to provide feedback on. (Please Se... = Phototherapy Light

Q9.30 29. Response During Power Outage: Please rate your level of agreement with the statements under optimal and minimal. *Note: The optimal and minimal requirements define a range.*

	1-Disagree (1)	2-Somewhat Disagree (2)	3-Neither Agree nor Disagree (3)	4-Mostly Agree (4)	5-Fully agree (5)	Other - Do not have the expertise to comment (6)
**Optimal:** Provides battery backup internal to device (1)	o	o	o	o	o	o
**Minimal:** Is not damaged by cycling of power/voltage spikes (2)	o	o	o	o	o	o

Display This Question:

If Please select the newborn technology (ies) that you would like to provide feedback on. (Please Se... = Phototherapy Light

Q9.31 30. Response During Power Outage: Please provide reasoning if you have chosen 3 or below.

____

____

____

____

____

Display This Question:

If Please select the newborn technology (ies) that you would like to provide feedback on. (Please Se... = Phototherapy Light

Q9.32 31. User Instructions: Please rate your level of agreement with the statements under optimal and minimal. *Note: The optimal and minimal requirements define a range.*

	1-Disagree (1)	2-Somewhat Disagree (2)	3-Neither Agree nor Disagree (3)	4-Mostly Agree (4)	5-Fully agree (5)	Other - Do not have the expertise to comment (6)
**Optimal:** User manual and additional training materials (checklists, videos, guides) in English and local language. Attached to device with labels and markings where possible. (1)	o	o	o	o	o	o
**Minimal:** User manual provided. (2)	o	o	o	o	o	o

Display This Question:

If Please select the newborn technology (ies) that you would like to provide feedback on. (Please Se... = Phototherapy Light

Q9.33 32. User Instructions: Please provide reasoning if you have chosen 3 or below.

____

____

____

____

____

Display This Question:

If Please select the newborn technology (ies) that you would like to provide feedback on. (Please Se... = Phototherapy Light

Q9.34 33. Warranty: Please rate your level of agreement with the statements under optimal and minimal. *Note: The optimal and minimal requirements define a range.*

	1-Disagree (1)	2-Somewhat Disagree (2)	3-Neither Agree nor Disagree (3)	4-Mostly Agree (4)	5-Fully agree (5)	Other - Do not have the expertise to comment (6)
**Optimal:** >5 years (1)	o	o	o	o	o	o
**Minimal:** ≥1 year (2)	o	o	o	o	o	o

Display This Question:

If Please select the newborn technology (ies) that you would like to provide feedback on. (Please Se... = Phototherapy Light

Q9.35 34. Warranty: Please provide reasoning if you have chosen 3 or below.

____

____

____

____

____

Display This Question:

If Please select the newborn technology (ies) that you would like to provide feedback on. (Please Se... = Phototherapy Light

Q9.36 35. Instrument Pricing: Please rate your level of agreement with the statements under optimal and minimal. *Note: The optimal and minimal requirements define a range.*

	1-Disagree (1)	2-Somewhat Disagree (2)	3-Neither Agree nor Disagree (3)	4-Mostly Agree (4)	5-Fully agree (5)	Other - Do not have the expertise to comment (6)
**Optimal:** (1)	○	○	○	○	○	○
**Minimal:** (2)	○	○	○	○	○	○

Display This Question:

If Please select the newborn technology (ies) that you would like to provide feedback on. (Please Se... = Phototherapy Light

Q9.37 36. Instrument Pricing: Please provide reasoning if you have chosen 3 or below.

____

____

____

____

____

End of Block: Phototherapy Lights

Start of Block: Warming Crib

Display This Question:

If Please select the newborn technology (ies) that you would like to provide feedback on. (Please Se... = Warming Crib

Q10.1 Warming Crib

Display This Question:

If Please select the newborn technology (ies) that you would like to provide feedback on. (Please Se... = Warming Crib

Q10.2 1. Intended Use: Please rate your level of agreement with the statements under optimal and minimal. *Note: The optimal and minimal requirements define a range.*

	1-Disagree (1)	2-Somewhat Disagree (2)	3-Neither Agree nor Disagree (3)	4-Mostly Agree (4)	5-Fully agree (5)	Other - Do not have the expertise to comment (6)
**Optimal:** Treatment and prevention of hypothermia in neonates requiring intensive thermal care. (1)	o	o	o	o	o	o
**Minimal:** Same as Optimal. (2)	o	o	o	o	o	o

Display This Question:

If Please select the newborn technology (ies) that you would like to provide feedback on. (Please Se... = Warming Crib

Q10.3 2. Intended Use: Please provide reasoning if you have chosen 3 or below.

____

____

____

____

____

Display This Question:

If Please select the newborn technology (ies) that you would like to provide feedback on. (Please Se... = Warming Crib

Q10.4 3. Target Operator: Please rate your level of agreement with the statements under optimal and minimal. *Note: The optimal and minimal requirements define a range.*

	1-Disagree (1)	2-Somewhat Disagree (2)	3-Neither agree nor disagree (3)	4-Mostly Agre (4)	5-Fully Agree (5)	Other - Do not have the expertise to comment (6)
**Optimal:** For use in low- and middle-income countries by a wide variety of clinicians, including nurses, clinical officers, and pediatricians. (1)	○	○	○	○	○	○
**Minimal:** Same as Optimal (2)	○	○	○	○	○	○

Display This Question:

If Please select the newborn technology (ies) that you would like to provide feedback on. (Please Se... = Warming Crib

Q10.5 4. Target Operator: Please provide reasoning if you have chosen 3 or below.

____

Display This Question:

If Please select the newborn technology (ies) that you would like to provide feedback on. (Please Se... = Warming Crib

Q10.6 5. Target Population: Please rate your level of agreement with the statements under optimal and minimal. *Note: The optimal and minimal requirements define a range.*

	1-Disagree (1)	2-Somewhat Disagree (2)	3-Neither Agree nor Disagree (3)	4-Mostly Agree (4)	5-Fully agree (5)	Other - Do not have the expertise to comment (6)
**Optimal:** Neonates ((1)	○	○	○	○	○	○
**Minimal:** Same as Optimal. (2)	○	○	○	○	○	○

Display This Question:

If Please select the newborn technology (ies) that you would like to provide feedback on. (Please Se... = Warming Crib

Q10.7 6. Target Population: Please provide reasoning if you have chosen 3 or below.

____

Display This Question:

If Please select the newborn technology (ies) that you would like to provide feedback on. (Please Se... = Warming Crib

Q10.8 7. Target Setting: Please rate your level of agreement with the statements under optimal and minimal. *Note: The optimal and minimal requirements define a range.*

	1-Disagree (1)	2-Somewhat Disagree (2)	3-Neither Agree nor Disagree (3)	4-Mostly Agree (4)	5-Fully agree (5)	Other - Do not have the expertise to comment (6)
**Optimal:** Hospitals in low-resource settings (1)	○	○	○	○	○	○
**Minimal:** Same as Optimal. (2)	○	○	○	○	○	○

Display This Question:

If Please select the newborn technology (ies) that you would like to provide feedback on. (Please Se... = Warming Crib

Q10.9 8. Target Setting: Please provide reasoning if you have chosen 3 or below.

____

____

____

____

____

Display This Question:

If Please select the newborn technology (ies) that you would like to provide feedback on. (Please Se... = Warming Crib

Q10.10 9. International Standard: Please rate your level of agreement with the statements under optimal and minimal. *Note: The optimal and minimal requirements define a range.*

	1-Disagree (1)	2-Somewhat Disagree (2)	3-Neither Agree nor Disagree (3)	4-Mostly Agree (4)	5-Fully agree (5)	Other - Do not have the expertise to comment (6)
**Optimal:** ISO 13485:2016 Medical devices – Quality management systems -- Requirements for regulatory purposes. (1)	○	○	○	○	○	○
**Minimal:** Same as Optimal. (2)	○	○	○	○	○	○

Display This Question:

If Please select the newborn technology (ies) that you would like to provide feedback on. (Please Se... = Warming Crib

Q10.11 10. International Standard: Please provide reasoning if you have chosen 3 or below.

____

____

____

____

____

Display This Question:

If Please select the newborn technology (ies) that you would like to provide feedback on. (Please Se... = Warming Crib

Q10.12 11. Regulation: Please rate your level of agreement with the statements under optimal and minimal. *Note: The optimal and minimal requirements define a range.*

	1-Disagree (1)	2-Somewhat Disagree (2)	3-Neither Agree nor Disagree (3)	4-Mostly Agree (4)	5-Fully agree (5)	Other - Do not have the expertise to comment (6)
**Optimal:** CE marking or US FDA Clearance (1)	○	○	○	○	○	○
**Minimal:** Same as Optimal. (2)	○	○	○	○	○	○

Display This Question:

If Please select the newborn technology (ies) that you would like to provide feedback on. (Please Se... = Warming Crib

Q10.13 12. Regulation: Please provide reasoning if you have chosen 3 or below.

____

____

____

____

____

Display This Question:

If Please select the newborn technology (ies) that you would like to provide feedback on. (Please Se... = Warming Crib

Q10.14 13. Benchtop Measurement Accuracy: Please rate your level of agreement with the statements under optimal and minimal. *Note: The optimal and minimal requirements define a range.*

	1-Disagree (1)	2-Somewhat Disagree (2)	3-Neither Agree nor Disagree (3)	4-Mostly Agree (4)	5-Fully agree (5)	Other - Do not have the expertise to comment (6)
**Optimal:** ±0.3°C (1)	○	○	○	○	○	○
**Minimal:** Same as Optimal. (2)	○	○	○	○	○	○

Display This Question:

If Please select the newborn technology (ies) that you would like to provide feedback on. (Please Se... = Warming Crib

Q10.15 14. Benchtop Measurement Accuracy: Please provide reasoning if you have chosen 3 or below.

____

____

____

____

____

Display This Question:

If Please select the newborn technology (ies) that you would like to provide feedback on. (Please Se... = Warming Crib

Q10.16 15. Clinical Measurement Accuracy: Please rate your level of agreement with the statements under optimal and minimal. *Note: The optimal and minimal requirements define a range.*

	1-Disagree (1)	2-Somewhat Disagree (2)	3-Neither Agree nor Disagree (3)	4-Mostly Agree (4)	5-Fully agree (5)	Other - Do not have the expertise to comment (6)
**Optimal:** ±0.5°C (1)	○	○	○	○	○	○
**Minimal:** Same as Optimal. (2)	○	○	○	○	○	○

Display This Question:

If Please select the newborn technology (ies) that you would like to provide feedback on. (Please Se... = Warming Crib

Q10.17 16. Clinical Measurement Accuracy: Please provide reasoning if you have chosen 3 or below.

____

____

____

____

____

Display This Question:

If Please select the newborn technology (ies) that you would like to provide feedback on. (Please Se... = Warming Crib

Q10.18 17. Heat Retention: Please rate your level of agreement with the statements under optimal and minimal. *Note: The optimal and minimal requirements define a range.*

	1-Disagree (1)	2-Somewhat Disagree (2)	3-Neither Agree nor Disagree (3)	4-Mostly Agree (4)	5-Fully agree (5)	Other - Do not have the expertise to comment (6)
**Optimal:** < 5°C loss over 4 hours (1)	○	○	○	○	○	○
**Minimal:** None. (2)	○	○	○	○	○	○

Display This Question:

If Please select the newborn technology (ies) that you would like to provide feedback on. (Please Se... = Warming Crib

Q10.19 18. Heat Retention: Please provide reasoning if you have chosen 3 or below.

____

____

____

____

____

Display This Question:

If Please select the newborn technology (ies) that you would like to provide feedback on. (Please Se... = Warming Crib

Q10.20 19. Maximum CO_2 Concentration: Please rate your level of agreement with the statements under optimal and minimal. *Note: The optimal and minimal requirements define a range.*

	1-Disagree (1)	2-Somewhat Disagree (2)	3-Neither Agree nor Disagree (3)	4-Mostly Agree (4)	5-Fully agree (5)	Other - Do not have the expertise to comment (6)
**Optimal:** 0.005% (1)	○	○	○	○	○	○
**Minimal:** Same as Optimal. (2)	○	○	○	○	○	○

Display This Question:

If Please select the newborn technology (ies) that you would like to provide feedback on. (Please Se... = Warming Crib

Q10.21 20. Maximum CO_2 Concentration: Please provide reasoning if you have chosen 3 or below.

____

____

____

____

____

Display This Question:

If Please select the newborn technology (ies) that you would like to provide feedback on. (Please Se... = Warming Crib

Q10.22 21. Maximum Rate of Change in Infant's Temperature: Please rate your level of agreement with the statements under optimal and minimal. *Note: The optimal and minimal requirements define a range.*

	1-Disagree (1)	2-Somewhat Disagree (2)	3-Neither Agree nor Disagree (3)	4-Mostly Agree (4)	5-Fully agree (5)	Other - Do not have the expertise to comment (6)
**Optimal:** 0.5°C/hour (1)	○	○	○	○	○	○
**Minimal:** Same as Optimal. (2)	○	○	○	○	○	○

Display This Question:

If Please select the newborn technology (ies) that you would like to provide feedback on. (Please Se... = Warming Crib

Q10.23 22. Maximum Rate of Change in Infant's Temperature: Please provide reasoning if you have chosen 3 or below.

____

____

____

____

____

Display This Question:

If Please select the newborn technology (ies) that you would like to provide feedback on. (Please Se... = Warming Crib

Q10.24 23. Maximum Temperature: Please rate your level of agreement with the statements under optimal and minimal. *Note: The optimal and minimal requirements define a range.*

	1-Disagree (1)	2-Somewhat Disagree (2)	3-Neither Agree nor Disagree (3)	4-Mostly Agree (4)	5-Fully agree (5)	Other - Do not have the expertise to comment (6)
**Optimal:** 38.0°C (1)	○	○	○	○	○	○
**Minimal:** Same as Optimal. (2)	○	○	○	○	○	○

Display This Question:

If Please select the newborn technology (ies) that you would like to provide feedback on. (Please Se... = Warming Crib

Q10.25 24. Maximum Temperature: Please provide reasoning if you have chosen 3 or below.

____

____

____

____

____

Display This Question:

If Please select the newborn technology (ies) that you would like to provide feedback on. (Please Se... = Warming Crib

Q10.26 25. Overshoot: Please rate your level of agreement with the statements under optimal and minimal. *Note: The optimal and minimal requirements define a range.*

	1-Disagree (1)	2-Somewhat Disagree (2)	3-Neither Agree nor Disagree (3)	4-Mostly Agree (4)	5-Fully agree (5)	Other - Do not have the expertise to comment (6)
**Optimal:** < 2°C (1)	○	○	○	○	○	○
**Minimal:** Same as Optimal. (2)	○	○	○	○	○	○

Display This Question:

If Please select the newborn technology (ies) that you would like to provide feedback on. (Please Se... = Warming Crib

Q10.27 26. Overshoot: Please provide reasoning if you have chosen 3 or below.

____

____

____

____

____

Display This Question:

If Please select the newborn technology (ies) that you would like to provide feedback on. (Please Se... = Warming Crib

Q10.28 27. Time to Indicate Accurate Temperature: Please rate your level of agreement with the statements under optimal and minimal. *Note: The optimal and minimal requirements define a range.*

	1-Disagree (1)	2-Somewhat Disagree (2)	3-Neither Agree nor Disagree (3)	4-Mostly Agree (4)	5-Fully agree (5)	Other - Do not have the expertise to comment (6)
**Optimal:** < 90 seconds (1)	○	○	○	○	○	○
**Minimal:** < 3 minutes (2)	○	○	○	○	○	○

Display This Question:

If Please select the newborn technology (ies) that you would like to provide feedback on. (Please Se... = Warming Crib

Q10.29 28. Time to Indicate Accurate Temperature: Please provide reasoning if you have chosen 3 or below.

____

____

____

____

____

Display This Question:

If Please select the newborn technology (ies) that you would like to provide feedback on. (Please Se... = Warming Crib

Q10.30 29. Uniformity: Please rate your level of agreement with the statements under optimal and minimal. *Note: The optimal and minimal requirements define a range.*

	1-Disagree (1)	2-Somewhat Disagree (2)	3-Neither Agree nor Disagree (3)	4-Mostly Agree (4)	5-Fully agree (5)	Other - Do not have the expertise to comment (6)
**Optimal:** < 1°C (1)	○	○	○	○	○	○
**Minimal:** Same as Optimal. (2)	○	○	○	○	○	○

Display This Question:

If Please select the newborn technology (ies) that you would like to provide feedback on. (Please Se... = Warming Crib

Q10.31 30. Uniformity: Please provide reasoning if you have chosen 3 or below.

____

____

____

____

____

Display This Question:

If Please select the newborn technology (ies) that you would like to provide feedback on. (Please Se... = Warming Crib

Q10.32 31. Alarm Characteristics Please rate your level of agreement with the statements under optimal and minimal. *Note: The optimal and minimal requirements define a range.*

	1-Disagree (1)	2-Somewhat Disagree (2)	3-Neither Agree nor Disagree (3)	4-Mostly Agree (4)	5-Fully agree (5)	Other - Do not have the expertise to comment (6)
**Optimal:** Visual and Auditory (1)	○	○	○	○	○	○
**Minimal:** Visual (2)	○	○	○	○	○	○

Display This Question:

If Please select the newborn technology (ies) that you would like to provide feedback on. (Please Se... = Warming Crib

Q10.33 32. Alarm Characteristics: Please provide reasoning if you have chosen 3 or below.

____

____

____

____

____

Display This Question:

If Please select the newborn technology (ies) that you would like to provide feedback on. (Please Se... = Warming Crib

Q10.34 33. Alarm Limits: Please rate your level of agreement with the statements under optimal and minimal. *Note: The optimal and minimal requirements define a range.*

	1-Disagree (1)	2-Somewhat Disagree (2)	3-Neither Agree nor Disagree (3)	4-Mostly Agree (4)	5-Fully agree (5)	Other - Do not have the expertise to comment (6)
**Optimal:** Adjustable (1)	○	○	○	○	○	○
**Minimal:** 36.5°C-37.5°C (2)	○	○	○	○	○	○

Display This Question:

If Please select the newborn technology (ies) that you would like to provide feedback on. (Please Se... = Warming Crib

Q10.35 34. Alarm Characteristics: Please provide reasoning if you have chosen 3 or below.

____

____

____

____

____

Display This Question:

If Please select the newborn technology (ies) that you would like to provide feedback on. (Please Se... = Warming Crib

Q10.36 35. Consumables: Please rate your level of agreement with the statements under optimal and minimal. *Note: The optimal and minimal requirements define a range.*

	1-Disagree (1)	2-Somewhat Disagree (2)	3-Neither Agree nor Disagree (3)	4-Mostly Agree (4)	5-Fully agree (5)	Other - Do not have the expertise to comment (6)
**Optimal:** > 12 months before required (1)	○	○	○	○	○	○
**Minimal:** > 6 months before required (2)	○	○	○	○	○	○

Display This Question:

If Please select the newborn technology (ies) that you would like to provide feedback on. (Please Se... = Warming Crib

Q10.37 36. Consumables: Please provide reasoning if you have chosen 3 or below.

____

____

____

____

____

Display This Question:

If Please select the newborn technology (ies) that you would like to provide feedback on. (Please Se... = Warming Crib

Q10.38 37. Decontamination: Please rate your level of agreement with the statements under optimal and minimal. *Note: The optimal and minimal requirements define a range.*

	1-Disagree (1)	2-Somewhat Disagree (2)	3-Neither Agree nor Disagree (3)	4-Mostly Agree (4)	5-Fully agree (5)	Other - Do not have the expertise to comment (6)
**Optimal:** Easy to clean with common disinfecting agents (1)	o	o	o	o	o	o
**Minimal:** Same as Optimal. (2)	o	o	o	o	o	o

Display This Question:

If Please select the newborn technology (ies) that you would like to provide feedback on. (Please Se... = Warming Crib

Q10.39 38. Decontamination: Please provide reasoning if you have chosen 3 or below.

____

____

____

____

____

Display This Question:

If Please select the newborn technology (ies) that you would like to provide feedback on. (Please Se... = Warming Crib

Q10.40 39. Maximum Power Consumption: Please rate your level of agreement with the statements under optimal and minimal. *Note: The optimal and minimal requirements define a range.*

	1-Disagree (1)	2-Somewhat Disagree (2)	3-Neither Agree nor Disagree (3)	4-Mostly Agree (4)	5-Fully agree (5)	Other - Do not have the expertise to comment (6)
**Optimal:** (1)	o	o	o	o	o	o
**Minimal:** (2)	o	o	o	o	o	o

Display This Question:

If Please select the newborn technology (ies) that you would like to provide feedback on. (Please Se... = Warming Crib

Q10.41 40. Maximum Power Consumption: Please provide reasoning if you have chosen 3 or below.

____

____

____

____

____

Display This Question:

If Please select the newborn technology (ies) that you would like to provide feedback on. (Please Se... = Warming Crib

Q10.42 41. Voltage: Please rate your level of agreement with the statements under optimal and minimal. *Note: The optimal and minimal requirements define a range.*

	1-Disagree (1)	2-Somewhat Disagree (2)	3-Neither Agree nor Disagree (3)	4-Mostly Agree (4)	5-Fully agree (5)	Other - Do not have the expertise to comment (6)
**Optimal:** 110-240V 50-60hz (1)	○	○	○	○	○	○
**Minimal:** 220-240V 50-60hz (2)	○	○	○	○	○	○

Display This Question:

If Please select the newborn technology (ies) that you would like to provide feedback on. (Please Se... = Warming Crib

Q10.43 42. Voltage: Please provide reasoning if you have chosen 3 or below.

____

____

____

____

____

Display This Question:

If Please select the newborn technology (ies) that you would like to provide feedback on. (Please Se... = Warming Crib

Q10.44 43. Operating Temperature: Please rate your level of agreement with the statements under optimal and minimal. *Note: The optimal and minimal requirements define a range.*

	1-Disagree (1)	2-Somewhat Disagree (2)	3-Neither Agree nor Disagree (3)	4-Mostly Agree (4)	5-Fully agree (5)	Other - Do not have the expertise to comment (6)
**Optimal:** Harsh ambient condition, temperature 5-45 °C, humidity 15% to 95%, dusty air, elevation >=2000 meters (1)	○	○	○	○	○	○
**Minimal:** Harsh ambient temperature 10-40 °C, humidity 15%-95%, dusty air, elevation up to 2000 meters (2)	○	○	○	○	○	○

Display This Question:

If Please select the newborn technology (ies) that you would like to provide feedback on. (Please Se... = Warming Crib

Q10.45 44. Operating Temperature: Please provide reasoning if you have chosen 3 or below.

____

____

____

Display This Question:

If Please select the newborn technology (ies) that you would like to provide feedback on. (Please Se... = Warming Crib

Q10.46 45. Patient Interface: Please rate your level of agreement with the statements under optimal and minimal. *Note: The optimal and minimal requirements define a range.*

	1-Disagree (1)	2-Somewhat Disagree (2)	3-Neither Agree nor Disagree (3)	4-Mostly Agree (4)	5-Fully agree (5)	Other - Do not have the expertise to comment (6)
**Optimal:** Interface is biocompatible and reusable (1)	○	○	○	○	○	○
**Minimal:** Interface is biocompatible (2)	○	○	○	○	○	○

Display This Question:

If Please select the newborn technology (ies) that you would like to provide feedback on. (Please Se... = Warming Crib

Q10.47 46. Patient Interface: Please provide reasoning if you have chosen 3 or below.

Display This Question:

If Please select the newborn technology (ies) that you would like to provide feedback on. (Please Se... = Warming Crib

Q10.48 47. Patient Accessibility and Visibility: Please rate your level of agreement with the statements under optimal and minimal. *Note: The optimal and minimal requirements define a range.*

	1-Disagree (1)	2-Somewhat Disagree (2)	3-Neither Agree nor Disagree (3)	4-Mostly Agree (4)	5-Fully agree (5)	Other - Do not have the expertise to comment (6)
**Optimal:** Patient is visible and accessible to healthcare worker. (1)	○	○	○	○	○	○
**Minimal:** Same as Optimal. (2)	○	○	○	○	○	○

Display This Question:

If Please select the newborn technology (ies) that you would like to provide feedback on. (Please Se... = Warming Crib

Q10.49 48. Patient Accessibility and Visibility: Please provide reasoning if you have chosen 3 or below.

____

____

____

____

____

Display This Question:

If Please select the newborn technology (ies) that you would like to provide feedback on. (Please Se... = Warming Crib

Q10.50 49. Patient Size: Please rate your level of agreement with the statements under optimal and minimal. *Note: The optimal and minimal requirements define a range.*

	1-Disagree (1)	2-Somewhat Disagree (2)	3-Neither Agree nor Disagree (3)	4-Mostly Agree (4)	5-Fully agree (5)	Other - Do not have the expertise to comment (6)
**Optimal:** Should fit a single infant (1)	○	○	○	○	○	○
**Minimal:** Same as Optimal. (2)	○	○	○	○	○	○

Display This Question:

If Please select the newborn technology (ies) that you would like to provide feedback on. (Please Se... = Warming Crib

Q10.51 50. Patient Size: Please provide reasoning if you have chosen 3 or below.

____

____

____

____

____

Display This Question:

If Please select the newborn technology (ies) that you would like to provide feedback on. (Please Se... = Warming Crib

Q10.52 51. Temperature Control: Please rate your level of agreement with the statements under optimal and minimal. *Note: The optimal and minimal requirements define a range.*

	1-Disagree (1)	2-Somewhat Disagree (2)	3-Neither Agree nor Disagree (3)	4-Mostly Agree (4)	5-Fully agree (5)	Other - Do not have the expertise to comment (6)
**Optimal:** Based on infant's temperature and includes fail-safe mode (1)	o	o	o	o	o	o
**Minimal:** Same as Optimal. (2)	o	o	o	o	o	o

Display This Question:

If Please select the newborn technology (ies) that you would like to provide feedback on. (Please Se... = Warming Crib

Q10.53 52. Temperature Control: Please provide reasoning if you have chosen 3 or below.

____

____

____

____

____

Display This Question:

If Please select the newborn technology (ies) that you would like to provide feedback on. (Please Se... = Warming Crib

Q10.54 53. User Manual: Please rate your level of agreement with the statements under optimal and minimal. *Note: The optimal and minimal requirements define a range.*

	1-Disagree (1)	2-Somewhat Disagree (2)	3-Neither Agree nor Disagree (3)	4-Mostly Agree (4)	5-Fully agree (5)	Other - Do not have the expertise to comment (6)
**Optimal:** User manual and additional training materials (checklists, videos, guides) in English and local language. Attached to device with labels and markings where possible. (1)	○	○	○	○	○	○
**Minimal:** User manual provided. (2)	○	○	○	○	○	○

Display This Question:

If Please select the newborn technology (ies) that you would like to provide feedback on. (Please Se... = Warming Crib

Q10.55 54. User Manual: Please provide reasoning if you have chosen 3 or below.

____

____

____

____

____

Display This Question:

If Please select the newborn technology (ies) that you would like to provide feedback on. (Please Se... = Warming Crib

Q10.56 55. Warranty: Please rate your level of agreement with the statements under optimal and minimal. *Note: The optimal and minimal requirements define a range.*

	1-Disagree (1)	2-Somewhat Disagree (2)	3-Neither Agree nor Disagree (3)	4-Mostly Agree (4)	5-Fully agree (5)	Other - Do not have the expertise to comment (6)
**Optimal:** 5 years (1)	○	○	○	○	○	○
**Minimal:** 1 year (2)	○	○	○	○	○	○

Display This Question:

If Please select the newborn technology (ies) that you would like to provide feedback on. (Please Se... = Warming Crib

Q10.57 56. Warranty: Please provide reasoning if you have chosen 3 or below.

____

____

____

____

____

Display This Question:

If Please select the newborn technology (ies) that you would like to provide feedback on. (Please Se... = Warming Crib

Q10.58 57. Instrument Pricing: Please rate your level of agreement with the statements under optimal and minimal. *Note: The optimal and minimal requirements define a range.*

	1-Disagree (1)	2-Somewhat Disagree (2)	3-Neither Agree nor Disagree (3)	4-Mostly Agree (4)	5-Fully agree (5)	Other - Do not have the expertise to comment (6)
**Optimal:** (1)	○	○	○	○	○	○
**Minimal:** (2)	○	○	○	○	○	○

Display This Question:

If Please select the newborn technology (ies) that you would like to provide feedback on. (Please Se... = Warming Crib

Q10.59 58. Instrument Pricing: Please provide reasoning if you have chosen 3 or below.

____

____

____

____

____

Display This Question:

If Please select the newborn technology (ies) that you would like to provide feedback on. (Please Se... = Warming Crib

Q10.60 59. Consumable Pricing: Please rate your level of agreement with the statements under optimal and minimal. *Note: The optimal and minimal requirements define a range.*

	1-Disagree (1)	2-Somewhat Disagree (2)	3-Neither Agree nor Disagree (3)	4-Mostly Agree (4)	5-Fully agree (5)	Other - Do not have the expertise to comment (6)
**Optimal:** (1)	○	○	○	○	○	○
**Minimal:** (2)	○	○	○	○	○	○

Display This Question:

If Please select the newborn technology (ies) that you would like to provide feedback on. (Please Se... = Warming Crib

Q10.61 60. Consumable Pricing: Please provide reasoning if you have chosen 3 or below.

____

____

____

____

____

End of Block: Warming Crib

Start of Block: Radiant Warmer

Display This Question:

If Please select the newborn technology (ies) that you would like to provide feedback on. (Please Se... = Radiant Warmer

Q11.1 Radiant Warmer

Display This Question:

If Please select the newborn technology (ies) that you would like to provide feedback on. (Please Se... = Radiant Warmer

Q11.2 1. Intended Use: Please rate your level of agreement with the statements under optimal and minimal. *Note: The optimal and minimal requirements define a range.*

	1-Disagree (1)	2-Somewhat Disagree (2)	3-Neither Agree nor Disagree (3)	4-Mostly Agree (4)	5-Fully agree (5)	Other - Do not have the expertise to comment (6)
**Optimal:** Treatment and prevention of hypothermia in neonates requiring intensive thermal care. (1)	○	○	○	○	○	○
**Minimal:** Same as Optimal. (2)	○	○	○	○	○	○

Display This Question:

If Please select the newborn technology (ies) that you would like to provide feedback on. (Please Se... = Radiant Warmer

Q11.3 2. Intended Use: Please provide reasoning if you have chosen 3 or below.

____

____

____

____

____

Display This Question:

If Please select the newborn technology (ies) that you would like to provide feedback on. (Please Se... = Radiant Warmer

Q11.4 3. Target Operator: Please rate your level of agreement with the statements under optimal and minimal. *Note: The optimal and minimal requirements define a range.*

	1-Disagree (1)	2-Somewhat Disagree (2)	3-Neither agree nor disagree (3)	4-Mostly Agre (4)	5-Fully Agree (5)	Other - Do not have the expertise to comment (6)
**Optimal:** For use in low- and middle-income countries by a wide variety of clinicians, including nurses, clinical officers, and pediatricians. (1)	○	○	○	○	○	○
**Minimal:** Same as Optimal (2)	○	○	○	○	○	○

Display This Question:

If Please select the newborn technology (ies) that you would like to provide feedback on. (Please Se... = Radiant Warmer

Q11.5 4. Target Operator: Please provide reasoning if you have chosen 3 or below.

____

Display This Question:

If Please select the newborn technology (ies) that you would like to provide feedback on. (Please Se... = Radiant Warmer

Q11.6 5. Target Population: Please rate your level of agreement with the statements under optimal and minimal. *Note: The optimal and minimal requirements define a range.*

	1-Disagree (1)	2-Somewhat Disagree (2)	3-Neither Agree nor Disagree (3)	4-Mostly Agree (4)	5-Fully agree (5)	Other - Do not have the expertise to comment (6)
**Optimal:** Neonates ((1)	○	○	○	○	○	○
**Minimal:** Same as Optimal. (2)	○	○	○	○	○	○

Display This Question:

If Please select the newborn technology (ies) that you would like to provide feedback on. (Please Se... = Radiant Warmer

Q11.7 6. Target Population: Please provide reasoning if you have chosen 3 or below.

____

Display This Question:

If Please select the newborn technology (ies) that you would like to provide feedback on. (Please Se... = Radiant Warmer

Q11.8 7. Target Setting: Please rate your level of agreement with the statements under optimal and minimal. *Note: The optimal and minimal requirements define a range.*

	1-Disagree (1)	2-Somewhat Disagree (2)	3-Neither Agree nor Disagree (3)	4-Mostly Agree (4)	5-Fully agree (5)	Other - Do not have the expertise to comment (6)
**Optimal:** Hospitals in low-resource settings (1)	○	○	○	○	○	○
**Minimal:** Same as Optimal. (2)	○	○	○	○	○	○

Display This Question:

If Please select the newborn technology (ies) that you would like to provide feedback on. (Please Se... = Radiant Warmer

Q11.9 8. Target Setting: Please provide reasoning if you have chosen 3 or below.

____

____

____

____

____

Display This Question:

If Please select the newborn technology (ies) that you would like to provide feedback on. (Please Se... = Radiant Warmer

Q11.10 9. International Standard: Please rate your level of agreement with the statements under optimal and minimal. *Note: The optimal and minimal requirements define a range.*

	1-Disagree (1)	2-Somewhat Disagree (2)	3-Neither Agree nor Disagree (3)	4-Mostly Agree (4)	5-Fully agree (5)	Other - Do not have the expertise to comment (6)
**Optimal:** ISO 13485:2016 Medical devices – Quality management systems -- Requirements for regulatory purposes. (1)	○	○	○	○	○	○
**Minimal:** Same as Optimal. (2)	○	○	○	○	○	○

Display This Question:

If Please select the newborn technology (ies) that you would like to provide feedback on. (Please Se... = Radiant Warmer

Q11.11 10. International Standard: Please provide reasoning if you have chosen 3 or below.

____

____

____

____

____

Display This Question:

If Please select the newborn technology (ies) that you would like to provide feedback on. (Please Se... = Radiant Warmer

Q11.12 11. Regulation: Please rate your level of agreement with the statements under optimal and minimal. *Note: The optimal and minimal requirements define a range.*

	1-Disagree (1)	2-Somewhat Disagree (2)	3-Neither Agree nor Disagree (3)	4-Mostly Agree (4)	5-Fully agree (5)	Other - Do not have the expertise to comment (6)
**Optimal:** CE marking or US FDA Clearance (1)	○	○	○	○	○	○
**Minimal:** Same as Optimal. (2)	○	○	○	○	○	○

Display This Question:

If Please select the newborn technology (ies) that you would like to provide feedback on. (Please Se... = Radiant Warmer

Q11.13 12. Regulation: Please provide reasoning if you have chosen 3 or below.

____

____

____

____

____

Display This Question:

If Please select the newborn technology (ies) that you would like to provide feedback on. (Please Se... = Radiant Warmer

Q11.14 13. Benchtop Measurement Accuracy: Please rate your level of agreement with the statements under optimal and minimal. *Note: The optimal and minimal requirements define a range.*

	1-Disagree (1)	2-Somewhat Disagree (2)	3-Neither Agree nor Disagree (3)	4-Mostly Agree (4)	5-Fully agree (5)	Other - Do not have the expertise to comment (6)
**Optimal:** ±0.1°C (1)	○	○	○	○	○	○
**Minimal:** Same as Optimal. (2)	○	○	○	○	○	○

Display This Question:

If Please select the newborn technology (ies) that you would like to provide feedback on. (Please Se... = Radiant Warmer

Q11.15 14. Benchtop Measurement Accuracy: Please provide reasoning if you have chosen 3 or below.

____

____

____

____

____

Display This Question:

If Please select the newborn technology (ies) that you would like to provide feedback on. (Please Se... = Radiant Warmer

Q11.16 15. Clinical Measurement Accuracy: Please rate your level of agreement with the statements under optimal and minimal. *Note: The optimal and minimal requirements define a range.*

	1-Disagree (1)	2-Somewhat Disagree (2)	3-Neither Agree nor Disagree (3)	4-Mostly Agree (4)	5-Fully agree (5)	Other - Do not have the expertise to comment (6)
**Optimal:** ±0.2°C (1)	○	○	○	○	○	○
**Minimal:** ±0.5°C (2)	○	○	○	○	○	○

Display This Question:

If Please select the newborn technology (ies) that you would like to provide feedback on. (Please Se... = Radiant Warmer

Q11.17 16. Clinical Measurement Accuracy: Please provide reasoning if you have chosen 3 or below.

____

____

____

____

____

Display This Question:

If Please select the newborn technology (ies) that you would like to provide feedback on. (Please Se... = Radiant Warmer

Q11.18 17. Stability: Please rate your level of agreement with the statements under optimal and minimal. *Note: The optimal and minimal requirements define a range.*

	1-Disagree (1)	2-Somewhat Disagree (2)	3-Neither Agree nor Disagree (3)	4-Mostly Agree (4)	5-Fully agree (5)	Other - Do not have the expertise to comment (6)
**Optimal:** < 0.5°C (1)	o	o	o	o	o	o
**Minimal:** Same as Optimal. (2)	o	o	o	o	o	o

Display This Question:

If Please select the newborn technology (ies) that you would like to provide feedback on. (Please Se... = Radiant Warmer

Q11.19 18. Stability: Please provide reasoning if you have chosen 3 or below.

____

____

____

____

____

Display This Question:

If Please select the newborn technology (ies) that you would like to provide feedback on. (Please Se... = Radiant Warmer

Q11.20 19. Includes APGAR timer: Please rate your level of agreement with the statements under optimal and minimal. *Note: The optimal and minimal requirements define a range.*

	1-Disagree (1)	2-Somewhat Disagree (2)	3-Neither Agree nor Disagree (3)	4-Mostly Agree (4)	5-Fully agree (5)	Other - Do not have the expertise to comment (6)
**Optimal:** Yes (1)	○	○	○	○	○	○
**Minimal:** No (2)	○	○	○	○	○	○

Display This Question:

If Please select the newborn technology (ies) that you would like to provide feedback on. (Please Se... = Radiant Warmer

Q11.21 20. Includes APGAR timer: Please provide reasoning if you have chosen 3 or below.

____

____

____

____

____

Display This Question:

If Please select the newborn technology (ies) that you would like to provide feedback on. (Please Se... = Radiant Warmer

Q11.22 21. Includes Scale: Please rate your level of agreement with the statements under optimal and minimal. *Note: The optimal and minimal requirements define a range.*

	1-Disagree (1)	2-Somewhat Disagree (2)	3-Neither Agree nor Disagree (3)	4-Mostly Agree (4)	5-Fully agree (5)	Other - Do not have the expertise to comment (6)
**Optimal:** Yes (1)	○	○	○	○	○	○
**Minimal:** No (2)	○	○	○	○	○	○

Display This Question:

If Please select the newborn technology (ies) that you would like to provide feedback on. (Please Se... = Radiant Warmer

Q11.23 22. Includes Scale: Please provide reasoning if you have chosen 3 or below.

____

____

____

____

____

Display This Question:

If Please select the newborn technology (ies) that you would like to provide feedback on. (Please Se... = Radiant Warmer

Q11.24 23. Mobility: Please rate your level of agreement with the statements under optimal and minimal. *Note: The optimal and minimal requirements define a range.*

	1-Disagree (1)	2-Somewhat Disagree (2)	3-Neither Agree nor Disagree (3)	4-Mostly Agree (4)	5-Fully agree (5)	Other - Do not have the expertise to comment (6)
**Optimal:** Has wheels; can be moved by one person (1)	o	o	o	o	o	o
**Minimal:** Same as Optimal. (2)	o	o	o	o	o	o

Display This Question:

If Please select the newborn technology (ies) that you would like to provide feedback on. (Please Se... = Radiant Warmer

Q11.25 24. Mobility: Please provide reasoning if you have chosen 3 or below.

____

____

____

____

____

Display This Question:

If Please select the newborn technology (ies) that you would like to provide feedback on. (Please Se... = Radiant Warmer

Q11.26 25. Time to Indicate Accurate Temperature: Please rate your level of agreement with the statements under optimal and minimal. *Note: The optimal and minimal requirements define a range.*

	1-Disagree (1)	2-Somewhat Disagree (2)	3-Neither Agree nor Disagree (3)	4-Mostly Agree (4)	5-Fully agree (5)	Other - Do not have the expertise to comment (6)
**Optimal:** < 90 seconds (1)	o	o	o	o	o	o
**Minimal:** < 3 minutes (2)	o	o	o	o	o	o

Display This Question:

If Please select the newborn technology (ies) that you would like to provide feedback on. (Please Se... = Radiant Warmer

Q11.27 26. Time to Indicate Accurate Temperature: Please provide reasoning if you have chosen 3 or below.

____

____

____

____

____

Display This Question:

If Please select the newborn technology (ies) that you would like to provide feedback on. (Please Se... = Radiant Warmer

Q11.28 27. Uniformity: Please rate your level of agreement with the statements under optimal and minimal. *Note: The optimal and minimal requirements define a range.*

	1-Disagree (1)	2-Somewhat Disagree (2)	3-Neither Agree nor Disagree (3)	4-Mostly Agree (4)	5-Fully agree (5)	Other - Do not have the expertise to comment (6)
**Optimal:** < 1°C (1)	o	o	o	o	o	o
**Minimal:** Same as Optimal. (2)	o	o	o	o	o	o

Display This Question:

If Please select the newborn technology (ies) that you would like to provide feedback on. (Please Se... = Radiant Warmer

Q11.29 28. Uniformity: Please provide reasoning if you have chosen 3 or below.

____

____

____

____

____

Display This Question:

If Please select the newborn technology (ies) that you would like to provide feedback on. (Please Se... = Radiant Warmer

Q11.30 29. Alarm Characteristics: Please rate your level of agreement with the statements under optimal and minimal. *Note: The optimal and minimal requirements define a range.*

	1-Disagree (1)	2-Somewhat Disagree (2)	3-Neither Agree nor Disagree (3)	4-Mostly Agree (4)	5-Fully agree (5)	Other - Do not have the expertise to comment (6)
**Optimal:** Visual and Auditory (1)	○	○	○	○	○	○
**Minimal:** Visual (2)	○	○	○	○	○	○

Display This Question:

If Please select the newborn technology (ies) that you would like to provide feedback on. (Please Se... = Radiant Warmer

Q11.31 30. Alarm Characteristics: Please provide reasoning if you have chosen 3 or below.

____

____

____

____

____

Display This Question:

If Please select the newborn technology (ies) that you would like to provide feedback on. (Please Se... = Radiant Warmer

Q11.32 31. Alarm Limits: Please rate your level of agreement with the statements under optimal and minimal. *Note: The optimal and minimal requirements define a range.*

	1-Disagree (1)	2-Somewhat Disagree (2)	3-Neither Agree nor Disagree (3)	4-Mostly Agree (4)	5-Fully agree (5)	Other - Do not have the expertise to comment (6)
**Optimal:** Adjustable (1)	○	○	○	○	○	○
**Minimal:** 36.5°C-37.5°C (2)	○	○	○	○	○	○

Display This Question:

If Please select the newborn technology (ies) that you would like to provide feedback on. (Please Se... = Radiant Warmer

Q11.33 32. Alarm Limits: Please provide reasoning if you have chosen 3 or below.

____

____

____

____

____

Display This Question:

If Please select the newborn technology (ies) that you would like to provide feedback on. (Please Se... = Radiant Warmer

Q11.34 33. Consumables: Please rate your level of agreement with the statements under optimal and minimal. *Note: The optimal and minimal requirements define a range.*

	1-Disagree (1)	2-Somewhat Disagree (2)	3-Neither Agree nor Disagree (3)	4-Mostly Agree (4)	5-Fully agree (5)	Other - Do not have the expertise to comment (6)
**Optimal:** > 12 months before required (1)	○	○	○	○	○	○
**Minimal:** > 6 months before required (2)	○	○	○	○	○	○

Display This Question:

If Please select the newborn technology (ies) that you would like to provide feedback on. (Please Se... = Radiant Warmer

Q11.35 34. Consumables: Please provide reasoning if you have chosen 3 or below.

____

____

____

____

____

Display This Question:

If Please select the newborn technology (ies) that you would like to provide feedback on. (Please Se... = Radiant Warmer

Q11.36 35. Decontamination: Please rate your level of agreement with the statements under optimal and minimal. *Note: The optimal and minimal requirements define a range.*

	1-Disagree (1)	2-Somewhat Disagree (2)	3-Neither Agree nor Disagree (3)	4-Mostly Agree (4)	5-Fully agree (5)	Other - Do not have the expertise to comment (6)
**Optimal:** Easy to clean with common disinfecting agents (1)	○	○	○	○	○	○
**Minimal:** Same as Optimal. (2)	○	○	○	○	○	○

Display This Question:

If Please select the newborn technology (ies) that you would like to provide feedback on. (Please Se... = Radiant Warmer

Q11.37 36. Decontamination: Please provide reasoning if you have chosen 3 or below.

____

____

____

____

____

Display This Question:

If Please select the newborn technology (ies) that you would like to provide feedback on. (Please Se... = Radiant Warmer

Q11.38 37. Maximum Power Consumption: Please rate your level of agreement with the statements under optimal and minimal. *Note: The optimal and minimal requirements define a range.*

	1-Disagree (1)	2-Somewhat Disagree (2)	3-Neither Agree nor Disagree (3)	4-Mostly Agree (4)	5-Fully agree (5)	Other - Do not have the expertise to comment (6)
**Optimal:** (1)	o	o	o	o	o	o
**Minimal:** (2)	o	o	o	o	o	o

Display This Question:

If Please select the newborn technology (ies) that you would like to provide feedback on. (Please Se... = Radiant Warmer

Q11.39 38. Maximum Power Consumption: Please provide reasoning if you have chosen 3 or below.

____

____

____

____

____

Display This Question:

If Please select the newborn technology (ies) that you would like to provide feedback on. (Please Se... = Radiant Warmer

Q11.40 39. Voltage: Please rate your level of agreement with the statements under optimal and minimal. *Note: The optimal and minimal requirements define a range.*

	1-Disagree (1)	2-Somewhat Disagree (2)	3-Neither Agree nor Disagree (3)	4-Mostly Agree (4)	5-Fully agree (5)	Other - Do not have the expertise to comment (6)
**Optimal:** 110-240V 50-60hz (1)	○	○	○	○	○	○
**Minimal:** 220-240V 50-60hz (2)	○	○	○	○	○	○

Display This Question:

If Please select the newborn technology (ies) that you would like to provide feedback on. (Please Se... = Radiant Warmer

Q11.41 40. Voltage: Please provide reasoning if you have chosen 3 or below.

____

____

____

____

____

Display This Question:

If Please select the newborn technology (ies) that you would like to provide feedback on. (Please Se... = Radiant Warmer

Q11.42 41. Operating Temperature: Please rate your level of agreement with the statements under optimal and minimal. *Note: The optimal and minimal requirements define a range.*

	1-Disagree (1)	2-Somewhat Disagree (2)	3-Neither Agree nor Disagree (3)	4-Mostly Agree (4)	5-Fully agree (5)	Other - Do not have the expertise to comment (6)
**Optimal:** Harsh ambient condition, temperature 5-45 °C, humidity 15% to 95%, dusty air, elevation >=2000 meters (1)	○	○	○	○	○	○
**Minimal:** Harsh ambient temperature 10-40 °C, humidity 15%-95%, dusty air, elevation up to 2000 meters (2)	○	○	○	○	○	○

Display This Question:

If Please select the newborn technology (ies) that you would like to provide feedback on. (Please Se... = Radiant Warmer

Q11.43 42. Operating Temperature: Please provide reasoning if you have chosen 3 or below.

____

____

____

Display This Question:

If Please select the newborn technology (ies) that you would like to provide feedback on. (Please Se... = Radiant Warmer

Q11.44 43. Patient Interface: Please rate your level of agreement with the statements under optimal and minimal. *Note: The optimal and minimal requirements define a range.*

	1-Disagree (1)	2-Somewhat Disagree (2)	3-Neither Agree nor Disagree (3)	4-Mostly Agree (4)	5-Fully agree (5)	Other - Do not have the expertise to comment (6)
**Optimal:** Interface is biocompatible and reusable (1)	○	○	○	○	○	○
**Minimal:** Interface is biocompatible (2)	○	○	○	○	○	○

Display This Question:

If Please select the newborn technology (ies) that you would like to provide feedback on. (Please Se... = Radiant Warmer

Q11.45 44. Patient Interface: Please provide reasoning if you have chosen 3 or below.

____

____

____

____

____

Display This Question:

If Please select the newborn technology (ies) that you would like to provide feedback on. (Please Se... = Radiant Warmer

Q11.46 45. Patient Accessibility and Visibility: Please rate your level of agreement with the statements under optimal and minimal. *Note: The optimal and minimal requirements define a range.*

	1-Disagree (1)	2-Somewhat Disagree (2)	3-Neither Agree nor Disagree (3)	4-Mostly Agree (4)	5-Fully agree (5)	Other - Do not have the expertise to comment (6)
**Optimal:** Patient is visible and accessible to healthcare worker. (1)	○	○	○	○	○	○
**Minimal:** Same as Optimal. (2)	○	○	○	○	○	○

Display This Question:

If Please select the newborn technology (ies) that you would like to provide feedback on. (Please Se... = Radiant Warmer

Q11.47 46. Patient Accessibility and Visibility: Please provide reasoning if you have chosen 3 or below.

____

____

____

____

____

Display This Question:

If Please select the newborn technology (ies) that you would like to provide feedback on. (Please Se... = Radiant Warmer

Q11.48 47. Temperature Control: Please rate your level of agreement with the statements under optimal and minimal. *Note: The optimal and minimal requirements define a range.*

	1-Disagree (1)	2-Somewhat Disagree (2)	3-Neither Agree nor Disagree (3)	4-Mostly Agree (4)	5-Fully agree (5)	Other - Do not have the expertise to comment (6)
**Optimal:** Based on infant's temperature and includes fail-safe mode (1)	o	o	o	o	o	o
**Minimal:** Same as Optimal. (2)	o	o	o	o	o	o

Display This Question:

If Please select the newborn technology (ies) that you would like to provide feedback on. (Please Se... = Radiant Warmer

Q11.49 48. Temperature Control: Please provide reasoning if you have chosen 3 or below.

____

____

____

____

____

Display This Question:

If Please select the newborn technology (ies) that you would like to provide feedback on. (Please Se... = Radiant Warmer

Q11.50 49. User Manual: Please rate your level of agreement with the statements under optimal and minimal. *Note: The optimal and minimal requirements define a range.*

	1-Disagree (1)	2-Somewhat Disagree (2)	3-Neither Agree nor Disagree (3)	4-Mostly Agree (4)	5-Fully agree (5)	Other - Do not have the expertise to comment (6)
**Optimal:** User manual and additional training materials (checklists, videos, guides) in English and local language. Attached to device with labels and markings where possible. (1)	o	o	o	o	o	o
**Minimal:** User manual provided. (2)	o	o	o	o	o	o

Display This Question:

If Please select the newborn technology (ies) that you would like to provide feedback on. (Please Se... = Radiant Warmer

Q11.51 50. User Manual: Please provide reasoning if you have chosen 3 or below.

____

____

____

____

____

Display This Question:

If Please select the newborn technology (ies) that you would like to provide feedback on. (Please Se... = Radiant Warmer

Q11.52 51. Warranty: Please rate your level of agreement with the statements under optimal and minimal. *Note: The optimal and minimal requirements define a range.*

	1-Disagree (1)	2-Somewhat Disagree (2)	3-Neither Agree nor Disagree (3)	4-Mostly Agree (4)	5-Fully agree (5)	Other - Do not have the expertise to comment (6)
**Optimal:** 5 years (1)	○	○	○	○	○	○
**Minimal:** 1 year (2)	○	○	○	○	○	○

Display This Question:

If Please select the newborn technology (ies) that you would like to provide feedback on. (Please Se... = Radiant Warmer

Q11.53 52. Warranty: Please provide reasoning if you have chosen 3 or below.

____

____

____

____

____

Display This Question:

If Please select the newborn technology (ies) that you would like to provide feedback on. (Please Se... = Radiant Warmer

Q11.54 53. Instrument Pricing: Please rate your level of agreement with the statements under optimal and minimal. *Note: The optimal and minimal requirements define a range.*

	1-Disagree (1)	2-Somewhat Disagree (2)	3-Neither Agree nor Disagree (3)	4-Mostly Agree (4)	5-Fully agree (5)	Other - Do not have the expertise to comment (6)
**Optimal:** (1)	o	o	o	o	o	o
**Minimal:** (2)	o	o	o	o	o	o

Display This Question:

If Please select the newborn technology (ies) that you would like to provide feedback on. (Please Se... = Radiant Warmer

Q11.55 54. Instrument Pricing: Please provide reasoning if you have chosen 3 or below.

____

____

____

____

____

Display This Question:

If Please select the newborn technology (ies) that you would like to provide feedback on. (Please Se... = Radiant Warmer

Q11.56 55. Consumable Pricing: Please rate your level of agreement with the statements under optimal and minimal. *Note: The optimal and minimal requirements define a range.*

	1-Disagree (1)	2-Somewhat Disagree (2)	3-Neither Agree nor Disagree (3)	4-Mostly Agree (4)	5-Fully agree (5)	Other - Do not have the expertise to comment (6)
**Optimal:** (1)	○	○	○	○	○	○
**Minimal:** (2)	○	○	○	○	○	○

Display This Question:

If Please select the newborn technology (ies) that you would like to provide feedback on. (Please Se... = Radiant Warmer

Q11.57 56. Consumable Pricing: Please provide reasoning if you have chosen 3 or below.

____

____

____

____

____

End of Block: Radiant Warmer

Start of Block: Temperature Monitor

Display This Question:

If Please select the newborn technology (ies) that you would like to provide feedback on. (Please Se... = Temperature Monitor

Q12.1 Temperature Monitor

Display This Question:

If Please select the newborn technology (ies) that you would like to provide feedback on. (Please Se... = Temperature Monitor

Q12.2 1. Intended Use: Please rate your level of agreement with the statements under optimal and minimal. *Note: The optimal and minimal requirements define a range.*

	1-Disagree (1)	2-Somewhat Disagree (2)	3-Neither Agree nor Disagree (3)	4-Mostly Agree (4)	5-Fully agree (5)	Other - Do not have the expertise to comment (6)
**Optimal:** To provide ongoing diagnoses and monitoring of treatment of hypo- and hyperthermia. (1)	○	○	○	○	○	○
**Minimal:** Same as Optimal. (2)	○	○	○	○	○	○

Display This Question:

If Please select the newborn technology (ies) that you would like to provide feedback on. (Please Se... = Temperature Monitor

Q12.3 2. Intended Use: Please provide reasoning if you have chosen 3 or below.

____

____

____

____

____

Display This Question:

If Please select the newborn technology (ies) that you would like to provide feedback on. (Please Se... = Temperature Monitor

Q12.4 3. Target Operator: Please rate your level of agreement with the statements under optimal and minimal. *Note: The optimal and minimal requirements define a range.*

	1-Disagree (1)	2-Somewhat Disagree (2)	3-Neither agree nor disagree (3)	4-Mostly Agre (4)	5-Fully Agree (5)	Other - Do not have the expertise to comment (6)
**Optimal:** For use in low- and middle-income countries by a wide variety of clinicians, including nurses, clinical officers, and pediatricians. (1)	○	○	○	○	○	○
**Minimal:** Same as Optimal (2)	○	○	○	○	○	○

Display This Question:

If Please select the newborn technology (ies) that you would like to provide feedback on. (Please Se... = Temperature Monitor

Q12.5 4. Target Operator: Please provide reasoning if you have chosen 3 or below.

____

Display This Question:

If Please select the newborn technology (ies) that you would like to provide feedback on. (Please Se... = Temperature Monitor

Q12.6 5. Target Population: Please rate your level of agreement with the statements under optimal and minimal. *Note: The optimal and minimal requirements define a range.*

	1-Disagree (1)	2-Somewhat Disagree (2)	3-Neither Agree nor Disagree (3)	4-Mostly Agree (4)	5-Fully agree (5)	Other - Do not have the expertise to comment (6)
**Optimal:** Neonates ((1)	○	○	○	○	○	○
**Minimal:** Same as Optimal. (2)	○	○	○	○	○	○

Display This Question:

If Please select the newborn technology (ies) that you would like to provide feedback on. (Please Se... = Temperature Monitor

Q12.7 6. Target Population: Please provide reasoning if you have chosen 3 or below.

____

Display This Question:

If Please select the newborn technology (ies) that you would like to provide feedback on. (Please Se... = Temperature Monitor

Q12.8 7. Target Setting: Please rate your level of agreement with the statements under optimal and minimal. *Note: The optimal and minimal requirements define a range.*

	1-Disagree (1)	2-Somewhat Disagree (2)	3-Neither Agree nor Disagree (3)	4-Mostly Agree (4)	5-Fully agree (5)	Other - Do not have the expertise to comment (6)
**Optimal:** Hospitals in low-resource settings (1)	○	○	○	○	○	○
**Minimal:** Same as Optimal. (2)	○	○	○	○	○	○

Display This Question:

If Please select the newborn technology (ies) that you would like to provide feedback on. (Please Se... = Temperature Monitor

Q12.9 8. Target Setting: Please provide reasoning if you have chosen 3 or below.

____

____

____

____

____

Display This Question:

If Please select the newborn technology (ies) that you would like to provide feedback on. (Please Se... = Temperature Monitor

Q12.10 9. International Standard: Please rate your level of agreement with the statements under optimal and minimal. *Note: The optimal and minimal requirements define a range.*

	1-Disagree (1)	2-Somewhat Disagree (2)	3-Neither Agree nor Disagree (3)	4-Mostly Agree (4)	5-Fully agree (5)	Other - Do not have the expertise to comment (6)
**Optimal:** ISO 13485:2016 Medical devices – Quality management systems -- Requirements for regulatory purposes. (1)	o	o	o	o	o	o
**Minimal:** Same as Optimal. (2)	o	o	o	o	o	o

Display This Question:

If Please select the newborn technology (ies) that you would like to provide feedback on. (Please Se... = Temperature Monitor

Q12.11 10. International Standard: Please provide reasoning if you have chosen 3 or below.

____

____

____

____

____

Display This Question:

If Please select the newborn technology (ies) that you would like to provide feedback on. (Please Se... = Temperature Monitor

Q12.12 11. Regulation: Please rate your level of agreement with the statements under optimal and minimal. *Note: The optimal and minimal requirements define a range.*

	1-Disagree (1)	2-Somewhat Disagree (2)	3-Neither Agree nor Disagree (3)	4-Mostly Agree (4)	5-Fully agree (5)	Other - Do not have the expertise to comment (6)
**Optimal:** CE marking or US FDA Clearance (1)	○	○	○	○	○	○
**Minimal:** Same as Optimal. (2)	○	○	○	○	○	○

Display This Question:

If Please select the newborn technology (ies) that you would like to provide feedback on. (Please Se... = Temperature Monitor

Q12.13 12. Regulation: Please provide reasoning if you have chosen 3 or below.

____

____

____

____

____

Display This Question:

If Please select the newborn technology (ies) that you would like to provide feedback on. (Please Se... = Temperature Monitor

Q12.14 13. Benchtop Measurement Accuracy: Please rate your level of agreement with the statements under optimal and minimal. *Note: The optimal and minimal requirements define a range.*

	1-Disagree (1)	2-Somewhat Disagree (2)	3-Neither Agree nor Disagree (3)	4-Mostly Agree (4)	5-Fully agree (5)	Other - Do not have the expertise to comment (6)
**Optimal:** ±0.1°C (1)	○	○	○	○	○	○
**Minimal:** Same as Optimal. (2)	○	○	○	○	○	○

Display This Question:

If Please select the newborn technology (ies) that you would like to provide feedback on. (Please Se... = Temperature Monitor

Q12.15 14. Benchtop Measurement Accuracy: Please provide reasoning if you have chosen 3 or below.

____

____

____

____

____

Display This Question:

If Please select the newborn technology (ies) that you would like to provide feedback on. (Please Se... = Temperature Monitor

Q12.16 15. Clinical Measurement Accuracy: Please rate your level of agreement with the statements under optimal and minimal. *Note: The optimal and minimal requirements define a range.*

	1-Disagree (1)	2-Somewhat Disagree (2)	3-Neither Agree nor Disagree (3)	4-Mostly Agree (4)	5-Fully agree (5)	Other - Do not have the expertise to comment (6)
**Optimal:** ±0.2°C (1)	◯	◯	◯	◯	◯	◯
**Minimal:** ±0.5°C (2)	◯	◯	◯	◯	◯	◯

Display This Question:

If Please select the newborn technology (ies) that you would like to provide feedback on. (Please Se... = Temperature Monitor

Q12.17 16. Clinical Measurement Accuracy: Please provide reasoning if you have chosen 3 or below.

____

____

____

____

____

Display This Question:

If Please select the newborn technology (ies) that you would like to provide feedback on. (Please Se... = Temperature Monitor

Q12.18 17. Time to Indicate Accurate Temperature: Please rate your level of agreement with the statements under optimal and minimal. *Note: The optimal and minimal requirements define a range.*

	1-Disagree (1)	2-Somewhat Disagree (2)	3-Neither Agree nor Disagree (3)	4-Mostly Agree (4)	5-Fully agree (5)	Other - Do not have the expertise to comment (6)
**Optimal:** < 90 seconds (1)	o	o	o	o	o	o
**Minimal:** < 3 minutes (2)	o	o	o	o	o	o

Display This Question:

If Please select the newborn technology (ies) that you would like to provide feedback on. (Please Se... = Temperature Monitor

Q12.19 18. Time to Indicate Accurate Temperature: Please provide reasoning if you have chosen 3 or below.

____

____

____

____

____

Display This Question:

If Please select the newborn technology (ies) that you would like to provide feedback on. (Please Se... = Temperature Monitor

Q12.20 19. Alarm Characteristics: Please rate your level of agreement with the statements under optimal and minimal. *Note: The optimal and minimal requirements define a range.*

	1-Disagree (1)	2-Somewhat Disagree (2)	3-Neither Agree nor Disagree (3)	4-Mostly Agree (4)	5-Fully agree (5)	Other - Do not have the expertise to comment (6)
**Optimal:** Visual and Auditory (1)	o	o	o	o	o	o
**Minimal:** Visual (2)	o	o	o	o	o	o

Display This Question:

If Please select the newborn technology (ies) that you would like to provide feedback on. (Please Se... = Temperature Monitor

Q12.21 20. Alarm Characteristics: Please provide reasoning if you have chosen 3 or below.

____

____

____

____

____

Display This Question:

If Please select the newborn technology (ies) that you would like to provide feedback on. (Please Se... = Temperature Monitor

Q12.22 21. Alarm Limits: Please rate your level of agreement with the statements under optimal and minimal. *Note: The optimal and minimal requirements define a range.*

	1-Disagree (1)	2-Somewhat Disagree (2)	3-Neither Agree nor Disagree (3)	4-Mostly Agree (4)	5-Fully agree (5)	Other - Do not have the expertise to comment (6)
**Optimal:** Adjustable (1)	○	○	○	○	○	○
**Minimal:** 36.5°C-37.5°C (2)	○	○	○	○	○	○

Display This Question:

If Please select the newborn technology (ies) that you would like to provide feedback on. (Please Se... = Temperature Monitor

Q12.23 22. Alarm Limits: Please provide reasoning if you have chosen 3 or below.

____

____

____

____

____

Display This Question:

If Please select the newborn technology (ies) that you would like to provide feedback on. (Please Se... = Temperature Monitor

Q12.24 23. Consumables: Please rate your level of agreement with the statements under optimal and minimal. *Note: The optimal and minimal requirements define a range.*

	1-Disagree (1)	2-Somewhat Disagree (2)	3-Neither Agree nor Disagree (3)	4-Mostly Agree (4)	5-Fully agree (5)	Other - Do not have the expertise to comment (6)
**Optimal:** > 12 months before required (1)	○	○	○	○	○	○
**Minimal:** > 6 months before required (2)	○	○	○	○	○	○

Display This Question:

If Please select the newborn technology (ies) that you would like to provide feedback on. (Please Se... = Temperature Monitor

Q12.25 24. Consumables: Please provide reasoning if you have chosen 3 or below.

____

____

____

____

____

Display This Question:

If Please select the newborn technology (ies) that you would like to provide feedback on. (Please Se... = Temperature Monitor

Q12.26 25. Decontamination: Please rate your level of agreement with the statements under optimal and minimal. *Note: The optimal and minimal requirements define a range.*

	1-Disagree (1)	2-Somewhat Disagree (2)	3-Neither Agree nor Disagree (3)	4-Mostly Agree (4)	5-Fully agree (5)	Other - Do not have the expertise to comment (6)
**Optimal:** Easy to clean with common disinfecting agents (1)	o	o	o	o	o	o
**Minimal:** Same as Optimal. (2)	o	o	o	o	o	o

Display This Question:

If Please select the newborn technology (ies) that you would like to provide feedback on. (Please Se... = Temperature Monitor

Q12.27 26. Decontamination: Please provide reasoning if you have chosen 3 or below.

____

____

____

____

____

Display This Question:

If Please select the newborn technology (ies) that you would like to provide feedback on. (Please Se... = Temperature Monitor

Q12.28 27. Battery Power: Please rate your level of agreement with the statements under optimal and minimal. *Note: The optimal and minimal requirements define a range.*

	1-Disagree (1)	2-Somewhat Disagree (2)	3-Neither Agree nor Disagree (3)	4-Mostly Agree (4)	5-Fully agree (5)	Other - Do not have the expertise to comment (6)
**Optimal:** >4 hour on single charge (1)	○	○	○	○	○	○
**Minimal:** None (2)	○	○	○	○	○	○

Display This Question:

If Please select the newborn technology (ies) that you would like to provide feedback on. (Please Se... = Temperature Monitor

Q12.29 28. Battery Power: Please provide reasoning if you have chosen 3 or below.

____

____

____

____

____

Display This Question:

If Please select the newborn technology (ies) that you would like to provide feedback on. (Please Se... = Temperature Monitor

Q12.30 29. Voltage: Please rate your level of agreement with the statements under optimal and minimal. *Note: The optimal and minimal requirements define a range.*

	1-Disagree (1)	2-Somewhat Disagree (2)	3-Neither Agree nor Disagree (3)	4-Mostly Agree (4)	5-Fully agree (5)	Other - Do not have the expertise to comment (6)
**Optimal:** 110-240V 50-60hz (1)	○	○	○	○	○	○
**Minimal:** 220-240V 50-60hz (2)	○	○	○	○	○	○

Display This Question:

If Please select the newborn technology (ies) that you would like to provide feedback on. (Please Se... = Temperature Monitor

Q12.31 30. Voltage: Please provide reasoning if you have chosen 3 or below.

____

____

____

____

____

Display This Question:

If Please select the newborn technology (ies) that you would like to provide feedback on. (Please Se... = Temperature Monitor

Q12.32 31. Patient Interface: Please rate your level of agreement with the statements under optimal and minimal. *Note: The optimal and minimal requirements define a range.*

	1-Disagree (1)	2-Somewhat Disagree (2)	3-Neither Agree nor Disagree (3)	4-Mostly Agree (4)	5-Fully agree (5)	Other - Do not have the expertise to comment (6)
**Optimal:** Interface is biocompatible and reusable (1)	o	o	o	o	o	o
**Minimal:** Interface is biocompatible (2)	o	o	o	o	o	o

Display This Question:

If Please select the newborn technology (ies) that you would like to provide feedback on. (Please Se... = Temperature Monitor

Q12.33 32. Patient Interface: Please provide reasoning if you have chosen 3 or below.

____

____

____

____

____

Display This Question:

If Please select the newborn technology (ies) that you would like to provide feedback on. (Please Se... = Temperature Monitor

Q12.34 33. Size: Please rate your level of agreement with the statements under optimal and minimal. *Note: The optimal and minimal requirements define a range.*

	1-Disagree (1)	2-Somewhat Disagree (2)	3-Neither Agree nor Disagree (3)	4-Mostly Agree (4)	5-Fully agree (5)	Other - Do not have the expertise to comment (6)
**Optimal:** Small footprint; portable and can be left at bedside (1)	○	○	○	○	○	○
**Minimal:** Same as Optimal. (2)	○	○	○	○	○	○

Display This Question:

If Please select the newborn technology (ies) that you would like to provide feedback on. (Please Se... = Temperature Monitor

Q12.35 34. Size: Please provide reasoning if you have chosen 3 or below.

____

____

____

____

____

Display This Question:

If Please select the newborn technology (ies) that you would like to provide feedback on. (Please Se... = Temperature Monitor

Q12.36 35. Weight: Please rate your level of agreement with the statements under optimal and minimal. *Note: The optimal and minimal requirements define a range.*

	1-Disagree (1)	2-Somewhat Disagree (2)	3-Neither Agree nor Disagree (3)	4-Mostly Agree (4)	5-Fully agree (5)	Other - Do not have the expertise to comment (6)
**Optimal:** (1)	○	○	○	○	○	○
**Minimal:** Same as Optimal. (2)	○	○	○	○	○	○

Display This Question:

If Please select the newborn technology (ies) that you would like to provide feedback on. (Please Se... = Temperature Monitor

Q12.37 36. Weight: Please provide reasoning if you have chosen 3 or below.

____

____

____

____

____

Display This Question:

If Please select the newborn technology (ies) that you would like to provide feedback on. (Please Se... = Temperature Monitor

Q12.38 37. User Manual: Please rate your level of agreement with the statements under optimal and minimal. *Note: The optimal and minimal requirements define a range.*

	1-Disagree (1)	2-Somewhat Disagree (2)	3-Neither Agree nor Disagree (3)	4-Mostly Agree (4)	5-Fully agree (5)	Other - Do not have the expertise to comment (6)
**Optimal:** User manual and additional training materials (checklists, videos, guides) in English and local language. Attached to device with labels and markings where possible. (1)	○	○	○	○	○	○
**Minimal:** User manual provided. (2)	○	○	○	○	○	○

Display This Question:

If Please select the newborn technology (ies) that you would like to provide feedback on. (Please Se... = Temperature Monitor

Q12.39 38. User Manual: Please provide reasoning if you have chosen 3 or below.

____

____

____

____

____

Display This Question:

If Please select the newborn technology (ies) that you would like to provide feedback on. (Please Se... = Temperature Monitor

Q12.40 39. Warranty: Please rate your level of agreement with the statements under optimal and minimal. *Note: The optimal and minimal requirements define a range.*

	1-Disagree (1)	2-Somewhat Disagree (2)	3-Neither Agree nor Disagree (3)	4-Mostly Agree (4)	5-Fully agree (5)	Other - Do not have the expertise to comment (6)
**Optimal:** 5 years (1)	o	o	o	o	o	o
**Minimal:** 1 year (2)	o	o	o	o	o	o

Display This Question:

If Please select the newborn technology (ies) that you would like to provide feedback on. (Please Se... = Temperature Monitor

Q12.41 40. Warranty: Please provide reasoning if you have chosen 3 or below.

____

____

____

____

____

Display This Question:

If Please select the newborn technology (ies) that you would like to provide feedback on. (Please Se... = Temperature Monitor

Q12.42 41. Instrument Pricing: Please rate your level of agreement with the statements under optimal and minimal. *Note: The optimal and minimal requirements define a range.*

	1-Disagree (1)	2-Somewhat Disagree (2)	3-Neither Agree nor Disagree (3)	4-Mostly Agree (4)	5-Fully agree (5)	Other - Do not have the expertise to comment (6)
**Optimal:** (1)	○	○	○	○	○	○
**Minimal:** (2)	○	○	○	○	○	○

Display This Question:

If Please select the newborn technology (ies) that you would like to provide feedback on. (Please Se... = Temperature Monitor

Q12.43 42. Instrument Pricing: Please provide reasoning if you have chosen 3 or below.

____

____

____

____

____

Display This Question:

If Please select the newborn technology (ies) that you would like to provide feedback on. (Please Se... = Temperature Monitor

Q12.44 43. Consumable Pricing: Please rate your level of agreement with the statements under optimal and minimal. *Note: The optimal and minimal requirements define a range.*

	1-Disagree (1)	2-Somewhat Disagree (2)	3-Neither Agree nor Disagree (3)	4-Mostly Agree (4)	5-Fully agree (5)	Other - Do not have the expertise to comment (6)
**Optimal:** (1)	○	○	○	○	○	○
**Minimal:** Same as Optimal. (2)	○	○	○	○	○	○

Display This Question:

If Please select the newborn technology (ies) that you would like to provide feedback on. (Please Se... = Temperature Monitor

Q12.45 44. Consumable Pricing: Please provide reasoning if you have chosen 3 or below.

____

____

____

____

____

End of Block: Temperature Monitor

Start of Block: Syringe Pump

Display This Question:

If Please select the newborn technology (ies) that you would like to provide feedback on. (Please Se... = Syringe Pump

Q13.1 Syringe Pump

Display This Question:

If Please select the newborn technology (ies) that you would like to provide feedback on. (Please Se... = Syringe Pump

Q13.2 1. Intended Use: Please rate your level of agreement with the statements under optimal and minimal. *Note: The optimal and minimal requirements define a range.*

	1-Disagree (1)	2-Somewhat Disagree (2)	3-Neither Agree nor Disagree (3)	4-Mostly Agree (4)	5-Fully agree (5)	Other - Do not have the expertise to comment (6)
**Optimal:** Treatment of conditions requiring precise administration of drugs or fluids; including but not limited to dextrose solution for hypoglycemia and antibiotics for infection. (1)	o	o	o	o	o	o
**Minimal:** Same as Optimal. (2)	o	o	o	o	o	o

Display This Question:

If Please select the newborn technology (ies) that you would like to provide feedback on. (Please Se... = Syringe Pump

Q13.3 2. Intended Use: Please provide reasoning if you have chosen 3 or below.

____

____

____

____

____

Display This Question:

If Please select the newborn technology (ies) that you would like to provide feedback on. (Please Se... = Syringe Pump

Q13.4 3. Target Operator: Please rate your level of agreement with the statements under optimal and minimal. *Note: The optimal and minimal requirements define a range.*

	1-Disagree (1)	2-Somewhat Disagree (2)	3-Neither agree nor disagree (3)	4-Mostly Agre (4)	5-Fully Agree (5)	Other - Do not have the expertise to comment (6)
**Optimal:** For use in low- and middle-income countries by a wide variety of clinicians, including nurses, clinical officers, and pediatricians. (1)	o	o	o	o	o	o
**Minimal:** Same as Optimal (2)	o	o	o	o	o	o

Display This Question:

If Please select the newborn technology (ies) that you would like to provide feedback on. (Please Se... = Syringe Pump

Q13.5 4. Target Operator: Please provide reasoning if you have chosen 3 or below.

____

Display This Question:

If Please select the newborn technology (ies) that you would like to provide feedback on. (Please Se... = Syringe Pump

Q13.6 5. Target Population: Please rate your level of agreement with the statements under optimal and minimal. *Note: The optimal and minimal requirements define a range.*

	1-Disagree (1)	2-Somewhat Disagree (2)	3-Neither Agree nor Disagree (3)	4-Mostly Agree (4)	5-Fully agree (5)	Other - Do not have the expertise to comment (6)
**Optimal:** Neonates ((1)	○	○	○	○	○	○
**Minimal:** Same as Optimal. (2)	○	○	○	○	○	○

Display This Question:

If Please select the newborn technology (ies) that you would like to provide feedback on. (Please Se... = Syringe Pump

Q13.7 6. Target Population: Please provide reasoning if you have chosen 3 or below.

____

Display This Question:

If Please select the newborn technology (ies) that you would like to provide feedback on. (Please Se... = Syringe Pump

Q13.8 7. Target Setting: Please rate your level of agreement with the statements under optimal and minimal. *Note: The optimal and minimal requirements define a range.*

	1-Disagree (1)	2-Somewhat Disagree (2)	3-Neither Agree nor Disagree (3)	4-Mostly Agree (4)	5-Fully agree (5)	Other - Do not have the expertise to comment (6)
**Optimal:** Hospitals in low-resource settings (1)	o	o	o	o	o	o
**Minimal:** Same as Optimal. (2)	o	o	o	o	o	o

Display This Question:

If Please select the newborn technology (ies) that you would like to provide feedback on. (Please Se... = Syringe Pump

Q13.9 8. Target Setting: Please provide reasoning if you have chosen 3 or below.

____

____

____

____

____

Display This Question:

If Please select the newborn technology (ies) that you would like to provide feedback on. (Please Se... = Syringe Pump

Q13.10 9. International Standard: Please rate your level of agreement with the statements under optimal and minimal. *Note: The optimal and minimal requirements define a range.*

	1-Disagree (1)	2-Somewhat Disagree (2)	3-Neither Agree nor Disagree (3)	4-Mostly Agree (4)	5-Fully agree (5)	Other - Do not have the expertise to comment (6)
**Optimal:** ISO 13485:2016 Medical devices – Quality management systems -- Requirements for regulatory purposes. (1)	○	○	○	○	○	○
**Minimal:** Same as Optimal. (2)	○	○	○	○	○	○

Display This Question:

If Please select the newborn technology (ies) that you would like to provide feedback on. (Please Se... = Syringe Pump

Q13.11 10. International Standard: Please provide reasoning if you have chosen 3 or below.

____

____

____

____

____

Display This Question:

If Please select the newborn technology (ies) that you would like to provide feedback on. (Please Se... = Syringe Pump

Q13.12 11. Regulation: Please rate your level of agreement with the statements under optimal and minimal. *Note: The optimal and minimal requirements define a range.*

	1-Disagree (1)	2-Somewhat Disagree (2)	3-Neither Agree nor Disagree (3)	4-Mostly Agree (4)	5-Fully agree (5)	Other - Do not have the expertise to comment (6)
**Optimal:** CE marking or US FDA Clearance (1)	○	○	○	○	○	○
**Minimal:** Same as Optimal. (2)	○	○	○	○	○	○

Display This Question:

If Please select the newborn technology (ies) that you would like to provide feedback on. (Please Se... = Syringe Pump

Q13.13 12. Regulation: Please provide reasoning if you have chosen 3 or below.

____

____

____

____

____

Display This Question:

If Please select the newborn technology (ies) that you would like to provide feedback on. (Please Se... = Syringe Pump

Q13.14 13. Benchtop Measurement Accuracy: Please rate your level of agreement with the statements under optimal and minimal. *Note: The optimal and minimal requirements define a range.*

	1-Disagree (1)	2-Somewhat Disagree (2)	3-Neither Agree nor Disagree (3)	4-Mostly Agree (4)	5-Fully agree (5)	Other - Do not have the expertise to comment (6)
**Optimal:** ±1.0% (1)	○	○	○	○	○	○
**Minimal:** ±3.0% (2)	○	○	○	○	○	○

Display This Question:

If Please select the newborn technology (ies) that you would like to provide feedback on. (Please Se... = Syringe Pump

Q13.15 14. Benchtop Measurement Accuracy: Please provide reasoning if you have chosen 3 or below.

____

____

____

____

____

Display This Question:

If Please select the newborn technology (ies) that you would like to provide feedback on. (Please Se... = Syringe Pump

Q13.16 15. Clinical Measurement Accuracy: Please rate your level of agreement with the statements under optimal and minimal. *Note: The optimal and minimal requirements define a range.*

	1-Disagree (1)	2-Somewhat Disagree (2)	3-Neither Agree nor Disagree (3)	4-Mostly Agree (4)	5-Fully agree (5)	Other - Do not have the expertise to comment (6)
**Optimal:** ±1.0% (1)	○	○	○	○	○	○
**Minimal:** ±3.0% (2)	○	○	○	○	○	○

Display This Question:

If Please select the newborn technology (ies) that you would like to provide feedback on. (Please Se... = Syringe Pump

Q13.17 16. Clinical Measurement Accuracy: Please provide reasoning if you have chosen 3 or below.

____

____

____

____

____

Display This Question:

If Please select the newborn technology (ies) that you would like to provide feedback on. (Please Se... = Syringe Pump

Q13.18 17. Flow Rate Requirements: Please rate your level of agreement with the statements under optimal and minimal. *Note: The optimal and minimal requirements define a range.*

	1-Disagree (1)	2-Somewhat Disagree (2)	3-Neither Agree nor Disagree (3)	4-Mostly Agree (4)	5-Fully agree (5)	Other - Do not have the expertise to comment (6)
**Optimal:** 0.5-60mL/hr (1)	○	○	○	○	○	○
**Minimal:** 3-30mL/hr (2)	○	○	○	○	○	○

Display This Question:

If Please select the newborn technology (ies) that you would like to provide feedback on. (Please Se... = Syringe Pump

Q13.19 18. Flow Rate Requirements: Please provide reasoning if you have chosen 3 or below.

____

____

____

____

____

Display This Question:

If Please select the newborn technology (ies) that you would like to provide feedback on. (Please Se... = Syringe Pump

Q13.20 19. Occlusion Detection: Please rate your level of agreement with the statements under optimal and minimal. *Note: The optimal and minimal requirements define a range.*

	1-Disagree (1)	2-Somewhat Disagree (2)	3-Neither Agree nor Disagree (3)	4-Mostly Agree (4)	5-Fully agree (5)	Other - Do not have the expertise to comment (6)
**Optimal:** Adjustable (1)	○	○	○	○	○	○
**Minimal:** 5, 10, 25 psi (2)	○	○	○	○	○	○

Display This Question:

If Please select the newborn technology (ies) that you would like to provide feedback on. (Please Se... = Syringe Pump

Q13.21 20. Occlusion Detection: Please provide reasoning if you have chosen 3 or below.

____

____

____

____

____

Display This Question:

If Please select the newborn technology (ies) that you would like to provide feedback on. (Please Se... = Syringe Pump

Q13.22 21. Syringe Requirements: Please rate your level of agreement with the statements under optimal and minimal. *Note: The optimal and minimal requirements define a range.*

	1-Disagree (1)	2-Somewhat Disagree (2)	3-Neither Agree nor Disagree (3)	4-Mostly Agree (4)	5-Fully agree (5)	Other - Do not have the expertise to comment (6)
**Optimal:** Syringe 5-60mL, works with multiple syringe types. Failsafe mode to reject syringes that don't match machine settings. (1)	o	o	o	o	o	o
**Minimal:** 5-60mL, proprietary syringes (2)	o	o	o	o	o	o

Display This Question:

If Please select the newborn technology (ies) that you would like to provide feedback on. (Please Se... = Syringe Pump

Q13.23 22. Syringe Requirements: Please provide reasoning if you have chosen 3 or below.

____

____

____

____

____

Display This Question:

If Please select the newborn technology (ies) that you would like to provide feedback on. (Please Se... = Syringe Pump

Q13.24 23. Ability to calculate flow rates based upon patient's size: Please rate your level of agreement with the statements under optimal and minimal. *Note: The optimal and minimal requirements define a range.*

	1-Disagree (1)	2-Somewhat Disagree (2)	3-Neither Agree nor Disagree (3)	4-Mostly Agree (4)	5-Fully agree (5)	Other - Do not have the expertise to comment (6)
**Optimal:** Yes (1)	○	○	○	○	○	○
**Minimal:** No (2)	○	○	○	○	○	○

Display This Question:

If Please select the newborn technology (ies) that you would like to provide feedback on. (Please Se... = Syringe Pump

Q13.25 24. Ability to calculate flow rates based upon patient's size: Please provide reasoning if you have chosen 3 or below.

____

____

____

____

____

Display This Question:

If Please select the newborn technology (ies) that you would like to provide feedback on. (Please Se... = Syringe Pump

Q13.26 25. Drug Library: Please rate your level of agreement with the statements under optimal and minimal. *Note: The optimal and minimal requirements define a range.*

	1-Disagree (1)	2-Somewhat Disagree (2)	3-Neither Agree nor Disagree (3)	4-Mostly Agree (4)	5-Fully agree (5)	Other - Do not have the expertise to comment (6)
**Optimal:** Yes (1)	○	○	○	○	○	○
**Minimal:** No (2)	○	○	○	○	○	○

Display This Question:

If Please select the newborn technology (ies) that you would like to provide feedback on. (Please Se... = Syringe Pump

Q13.27 26. Drug Library: Please provide reasoning if you have chosen 3 or below.

____

____

____

____

____

Display This Question:

If Please select the newborn technology (ies) that you would like to provide feedback on. (Please Se... = Syringe Pump

Q13.28 27. Alarm Characteristics: Please rate your level of agreement with the statements under optimal and minimal. *Note: The optimal and minimal requirements define a range.*

	1-Disagree (1)	2-Somewhat Disagree (2)	3-Neither Agree nor Disagree (3)	4-Mostly Agree (4)	5-Fully agree (5)	Other - Do not have the expertise to comment (6)
**Optimal:** Visual and Auditory (1)	○	○	○	○	○	○
**Minimal:** Visual (2)	○	○	○	○	○	○

Display This Question:

If Please select the newborn technology (ies) that you would like to provide feedback on. (Please Se... = Syringe Pump

Q13.29 28. Alarm Characteristics: Please provide reasoning if you have chosen 3 or below.

____

____

____

____

____

Display This Question:

If Please select the newborn technology (ies) that you would like to provide feedback on. (Please Se... = Syringe Pump

Q13.30 29. Decontamination: Please rate your level of agreement with the statements under optimal and minimal. *Note: The optimal and minimal requirements define a range.*

	1-Disagree (1)	2-Somewhat Disagree (2)	3-Neither Agree nor Disagree (3)	4-Mostly Agree (4)	5-Fully agree (5)	Other - Do not have the expertise to comment (6)
**Optimal:** Easy to clean with common disinfecting agents (1)	○	○	○	○	○	○
**Minimal:** Same as Optimal. (2)	○	○	○	○	○	○

Display This Question:

If Please select the newborn technology (ies) that you would like to provide feedback on. (Please Se... = Syringe Pump

Q13.31 30. Decontamination: Please provide reasoning if you have chosen 3 or below.

____

____

____

____

____

Display This Question:

If Please select the newborn technology (ies) that you would like to provide feedback on. (Please Se... = Syringe Pump

Q13.32 31. Maximum Power Consumption: Please rate your level of agreement with the statements under optimal and minimal. *Note: The optimal and minimal requirements define a range.*

	1-Disagree (1)	2-Somewhat Disagree (2)	3-Neither Agree nor Disagree (3)	4-Mostly Agree (4)	5-Fully agree (5)	Other - Do not have the expertise to comment (6)
**Optimal:** (1)	o	o	o	o	o	o
**Minimal:** (2)	o	o	o	o	o	o

Display This Question:

If Please select the newborn technology (ies) that you would like to provide feedback on. (Please Se... = Syringe Pump

Q13.33 32. Maximum Power Consumption: Please provide reasoning if you have chosen 3 or below.

____

____

____

____

____

Display This Question:

If Please select the newborn technology (ies) that you would like to provide feedback on. (Please Se... = Syringe Pump

Q13.34 33. Voltage: Please rate your level of agreement with the statements under optimal and minimal. *Note: The optimal and minimal requirements define a range.*

	1-Disagree (1)	2-Somewhat Disagree (2)	3-Neither Agree nor Disagree (3)	4-Mostly Agree (4)	5-Fully agree (5)	Other - Do not have the expertise to comment (6)
**Optimal:** 110-240V 50-60hz (1)	o	o	o	o	o	o
**Minimal:** 220-240V 50-60hz (2)	o	o	o	o	o	o

Display This Question:

If Please select the newborn technology (ies) that you would like to provide feedback on. (Please Se... = Syringe Pump

Q13.35 34. Voltage: Please provide reasoning if you have chosen 3 or below.

____

____

____

____

____

Display This Question:

If Please select the newborn technology (ies) that you would like to provide feedback on. (Please Se... = Syringe Pump

Q13.36 35. Battery Power: Please rate your level of agreement with the statements under optimal and minimal. *Note: The optimal and minimal requirements define a range.*

	1-Disagree (1)	2-Somewhat Disagree (2)	3-Neither Agree nor Disagree (3)	4-Mostly Agree (4)	5-Fully agree (5)	Other - Do not have the expertise to comment (6)
**Optimal:** >4hr on single charge (1)	○	○	○	○	○	○
**Minimal:** None. (2)	○	○	○	○	○	○

Display This Question:

If Please select the newborn technology (ies) that you would like to provide feedback on. (Please Se... = Syringe Pump

Q13.37 36. Battery Power: Please provide reasoning if you have chosen 3 or below.

____

____

____

____

____

Display This Question:

If Please select the newborn technology (ies) that you would like to provide feedback on. (Please Se... = Syringe Pump

Q13.38 37. Size: Please rate your level of agreement with the statements under optimal and minimal. *Note: The optimal and minimal requirements define a range.*

	1-Disagree (1)	2-Somewhat Disagree (2)	3-Neither Agree nor Disagree (3)	4-Mostly Agree (4)	5-Fully agree (5)	Other - Do not have the expertise to comment (6)
**Optimal:** Small footprint; portable (1)	○	○	○	○	○	○
**Minimal:** Same as Optimal. (2)	○	○	○	○	○	○

Display This Question:

If Please select the newborn technology (ies) that you would like to provide feedback on. (Please Se... = Syringe Pump

Q13.39 38. Size: Please provide reasoning if you have chosen 3 or below.

____

____

____

____

____

Display This Question:

If Please select the newborn technology (ies) that you would like to provide feedback on. (Please Se... = Syringe Pump

Q13.40 39. Weight: Please rate your level of agreement with the statements under optimal and minimal. *Note: The optimal and minimal requirements define a range.*

	1-Disagree (1)	2-Somewhat Disagree (2)	3-Neither Agree nor Disagree (3)	4-Mostly Agree (4)	5-Fully agree (5)	Other - Do not have the expertise to comment (6)
**Optimal:** (1)	○	○	○	○	○	○
**Minimal:** (2)	○	○	○	○	○	○

Display This Question:

If Please select the newborn technology (ies) that you would like to provide feedback on. (Please Se... = Syringe Pump

Q13.41 40. Weight: Please provide reasoning if you have chosen 3 or below.

____

____

____

____

____

Display This Question:

If Please select the newborn technology (ies) that you would like to provide feedback on. (Please Se... = Syringe Pump

Q13.42 41. User Manual: Please rate your level of agreement with the statements under optimal and minimal. *Note: The optimal and minimal requirements define a range.*

	1-Disagree (1)	2-Somewhat Disagree (2)	3-Neither Agree nor Disagree (3)	4-Mostly Agree (4)	5-Fully agree (5)	Other - Do not have the expertise to comment (6)
**Optimal:** User manual and additional training materials (checklists, videos, guides) in English and local language. Attached to device with labels and markings where possible. (1)	o	o	o	o	o	o
**Minimal:** User manual provided. (2)	o	o	o	o	o	o

Display This Question:

If Please select the newborn technology (ies) that you would like to provide feedback on. (Please Se... = Syringe Pump

Q13.43 42. User Manual: Please provide reasoning if you have chosen 3 or below.

____

____

____

____

____

Display This Question:

If Please select the newborn technology (ies) that you would like to provide feedback on. (Please Se... = Syringe Pump

Q13.44 43. Warranty: Please rate your level of agreement with the statements under optimal and minimal. *Note: The optimal and minimal requirements define a range.*

	1-Disagree (1)	2-Somewhat Disagree (2)	3-Neither Agree nor Disagree (3)	4-Mostly Agree (4)	5-Fully agree (5)	Other - Do not have the expertise to comment (6)
**Optimal:** 5 years (1)	○	○	○	○	○	○
**Minimal:** 1 year (2)	○	○	○	○	○	○

Display This Question:

If Please select the newborn technology (ies) that you would like to provide feedback on. (Please Se... = Syringe Pump

Q13.45 44. Warranty: Please provide reasoning if you have chosen 3 or below.

____

____

____

____

____

Display This Question:

If Please select the newborn technology (ies) that you would like to provide feedback on. (Please Se... = Syringe Pump

Q13.46 45. Instrument Pricing: Please rate your level of agreement with the statements under optimal and minimal. *Note: The optimal and minimal requirements define a range.*

	1-Disagree (1)	2-Somewhat Disagree (2)	3-Neither Agree nor Disagree (3)	4-Mostly Agree (4)	5-Fully agree (5)	Other - Do not have the expertise to comment (6)
**Optimal:** (1)	○	○	○	○	○	○
**Minimal:** (2)	○	○	○	○	○	○

Display This Question:

If Please select the newborn technology (ies) that you would like to provide feedback on. (Please Se... = Syringe Pump

Q13.47 46. Instrument Pricing: Please provide reasoning if you have chosen 3 or below.

____

____

____

____

____

Display This Question:

If Please select the newborn technology (ies) that you would like to provide feedback on. (Please Se... = Syringe Pump

Q13.48 47. Consumable Pricing: Please rate your level of agreement with the statements under optimal and minimal. *Note: The optimal and minimal requirements define a range.*

	1-Disagree (1)	2-Somewhat Disagree (2)	3-Neither Agree nor Disagree (3)	4-Mostly Agree (4)	5-Fully agree (5)	Other - Do not have the expertise to comment (6)
**Optimal:** (1)	○	○	○	○	○	○
**Minimal:** (2)	○	○	○	○	○	○

Display This Question:

If Please select the newborn technology (ies) that you would like to provide feedback on. (Please Se... = Syringe Pump

Q13.49 48. Consumable Pricing: Please provide reasoning if you have chosen 3 or below.

____

____

____

____

____

End of Block: Syringe Pump

Start of Block: Sepsis Test

Display This Question:

If Please select the newborn technology (ies) that you would like to provide feedback on. (Please Se... = Sepsis Test

Q14.1 **Sepsis Test:**

Background: Sepsis (serious infection) in neonates and young infants is devastating for many babies and their families around the world. It is also not easy to diagnose. There are some useful guidelines that help to identify neonates and young infants at risk of sepsis, and guide clinical management. However, even when these guidelines are used, many more babies

receive antibiotics than those who truly have serious bacterial infections which need antibiotics. (1) Researchers around the world are trying to develop a point of care test for sepsis. This is a test that can be done by any healthcare worker with a quick result. However, a point of care test for sepsis could be used in a number of ways, and it is important that researchers know which way (a ‘Use Case’) will be most helpful to healthcare workers. The purpose of this component of the survey is to evaluate which of these ‘Use Cases’ would be of most practical benefit to clinicians who manage neonates with possible serious bacterial infections. The aim is that a test like this would be used in combination with existing guidelines provided by the World Health Organization. (2) Use Cases Descriptions: Please read the following 6 Use Case descriptions and then answer the 3 questions below. **Use Case 1. Start Antibiotics - Community Referral:** A test that can be used when a baby first comes to a health facility from the community for assessment, and has one or more signs of possible serious bacterial infection. Examples of these include respiratory rate >60 breaths per minute, being unable to breastfeed, or deep jaundice. The test is to help the healthcare worker decide if they should start antibiotics. If the test is positive, this means that the baby is likely to have a serious bacterial infection. The baby needs antibiotics and supportive care. If a blood culture can be sent, this should be collected before the antibiotics are started. If the test is negative, this means the baby is highly unlikely to have a serious bacterial infection. Instead they need careful observation, and the healthcare worker should consider other reasons for their illness. **Use Case 2. Start Antibiotics - Well Baby with Risk Factors at Birth:** A test that can be used when an otherwise well baby has been born with risk factors for sepsis. Examples of these risk factors are fever in the mother during labour, prolonged rupture of the membranes (>18 hours), or foul-smelling amniotic fluid. Other non-maternal risk factors might include preterm labour. The test is to help the healthcare worker decide if they should start antibiotics. If the test is positive, this means that the baby is likely to have a serious bacterial infection. The baby needs antibiotics and supportive care. If a blood culture can be sent, this should be collected before the antibiotics are started. If the test is negative, this means the baby is highly unlikely to have a serious bacterial infection. The baby would stay with mother and receive normal newborn care.

Use Case 3. Start Antibiotics - Unwell at Birth: A test that can be used when a baby has been born with signs of sepsis with or without maternal risk factors. Signs of sepsis include tachypnea, temperature instability, or tachycardia. The test is to help the healthcare worker decide if they should start antibiotics.

If the test is positive, this means the baby is likely to have a serious bacterial infection. The baby needs antibiotics and supportive care. If a blood culture can be sent, the sample should be collected before the antibiotics are started.

If the test is negative, this means the baby is highly unlikely to have a serious bacterial infection. If the baby remains unwell, they need careful observation, and the healthcare worker should consider other reasons for their illness. **Use Case 4. Start Antibiotics - Small or Premature Baby who becomes Unwell:** A test that can be used for a baby who is already admitted to a health facility because they are small or premature who becomes unwell and has one or more signs of a possible serious bacterial infection. The test is to help the healthcare worker decide if they should start antibiotics. If the test is positive, this means that the baby is likely to have a serious bacterial infection. The baby needs antibiotics and supportive care. If a blood culture

can be sent, this should be collected before the antibiotics are started.   If the test is negative, this means the baby is highly unlikely to have a serious bacterial infection. Instead they need careful observation, and the healthcare worker should consider other reasons for their illness.   **Use Case 5. Stop Antibiotics:** A test that can be used for a baby who is already admitted to a health facility and who has already received at least one day of antibiotics for a possible serious bacterial infection. The test is to help the healthcare worker decide if the antibiotics can stop.   If the test is positive this means that the baby is likely to have a serious bacterial infection. The baby needs to continue their antibiotics. If there are positive blood or cerebrospinal fluid culture results, the antibiotics may need to change to make sure they are the best antibiotic to treat the infection that has been identified.   If the test is negative, this means that the baby is highly unlikely to have a serious bacterial infection. The antibiotics can stop. If the baby is still unwell, the healthcare worker should consider other reasons for their illness.   **Use Case 6. Resistance:** A test that can be used for a baby who is already admitted to a district health facility, has already commenced antibiotics, and remains unwell. The test is to tell the healthcare worker if the baby has an infection resistant to first line (the usual) antibiotics.   If the test is positive this means the baby is highly likely to have a serious bacterial infection which is resistant to the first line antibiotics which are usually started. The baby needs a different antibiotic. The test may provide some information which guides the choice of this antibiotic.   If the test is negative, this means that either the baby does not have a serious bacterial infection, or that the infection is being appropriately treated by the first line antibiotics which are usually started.   References: (1)   WHO Recommendations on newborn health: guidelines approved by the WHO Guidelines Review Committee. Geneva: World Health Organization; 2017 (WHO/MCA/17.07). Licence: CC BY-NE-SA 3.0 IGO.   (2)   Saha S, Schrag S, Arifeen S, et al. Causes and incidence of community-acquired serious infections among young children in south Asia (ANISA): an observational cohort study. Lancet 2018; 392:145-59.

Display This Question:

If Please select the newborn technology (ies) that you would like to provide feedback on. (Please Se... = Sepsis Test

Q14.2 **Question 1.** Based upon the Use Case descriptions provided above, please prioritize Use Case 1-6. (drag the indicator to the priority score for each of the 6 Use Cases)

Low Priority   Medium Priority   High Priority

0 10 20 30 40 50 60 70 80 90 100

Use Case 1. Start Antibiotics - Community Referral ()	
Use Case 2. Start Antibiotics - Well Baby with Risk Factors at Birth ()	
Use Case 3. Start Antibiotics - Unwell at Birth ()	
Use Case 4. Start Antibiotics - Small or Premature Baby who becomes Unwell ()	
Use Case 5. Stop Antibiotics ()	
Use Case 6: Resistance ()	

Display This Question:

If Please select the newborn technology (ies) that you would like to provide feedback on. (Please Se... = Sepsis Test

Q14.3 **Question 2:** Based upon the Use Case descriptions provided above, Rank Use Case 1-6. (drag the 6 Use Cases below into the appropriate order of rank - 1 being the highest rank, 6 being the lowest rank)

______ Use Case 1. Start Antibiotics - Community Referral (1)
______ Use Case 2. Start Antibiotics - Well Baby with Risk Factors at Birth (2)
______ Use Case 3. Start Antibiotics - Unwell at Birth (3)
______ Use Case 4. Start Antibiotics - Small or Premature Baby who becomes Unwell (4)
______ Use Case 5. Stop Antibiotics (5)
______ Use Case 6. Resistance (6)

Display This Question:

If Please select the newborn technology (ies) that you would like to provide feedback on. (Please Se... = Sepsis Test

Q14.4 **Question 3:** Are there other Use Cases or situations where you think a point of care test could help healthcare workers to manage young infants with possible serious bacterial infections? If so, please describe them here:

____

____

____

End of Block: Sepsis Test

Start of Block: Hemoglobin Test

Display This Question:

If Please select the newborn technology (ies) that you would like to provide feedback on. (Please Se... = Hemoglobin Test

Q15.1 Hemoglobin Test

Display This Question:

If Please select the newborn technology (ies) that you would like to provide feedback on. (Please Se... = Hemoglobin Test

Q15.2 1. Intended Use: Please rate your level of agreement with the statements under optimal and minimal. *Note: The optimal and minimal requirements define a range.*

	1-Disagree (1)	2-Somewhat Disagree (2)	3-Neither Agree nor Disagree (3)	4-Mostly Agree (4)	5-Fully agree (5)	Other - Do not have the expertise to comment (6)
**Optimal:** Quantitative determination of hemoglobin in capillary, venous, or arterial whole blood. (1)	○	○	○	○	○	○
**Minimal:** Same as Optimal. (2)	○	○	○	○	○	○

Display This Question:

If Please select the newborn technology (ies) that you would like to provide feedback on. (Please Se... = Hemoglobin Test

Q15.3 2. Intended Use: Please provide reasoning if you have chosen 3 or below.

____

____

____

____

____

Display This Question:

If Please select the newborn technology (ies) that you would like to provide feedback on. (Please Se... = Hemoglobin Test

Q15.4 3. Target Operator: Please rate your level of agreement with the statements under optimal and minimal. *Note: The optimal and minimal requirements define a range.*

	1-Disagree (1)	2-Somewhat Disagree (2)	3-Neither agree nor disagree (3)	4-Mostly Agre (4)	5-Fully Agree (5)	Other - Do not have the expertise to comment (6)
**Optimal:** For use in low- and middle-income countries by a wide variety of clinicians, including nurses, clinical officers, and pediatricians. (1)	○	○	○	○	○	○
**Minimal:** Same as Optimal (2)	○	○	○	○	○	○

Display This Question:

If Please select the newborn technology (ies) that you would like to provide feedback on. (Please Se... = Hemoglobin Test

Q15.5 4. Target Operator: Please provide reasoning if you have chosen 3 or below.

____

Display This Question:

If Please select the newborn technology (ies) that you would like to provide feedback on. (Please Se... = Hemoglobin Test

Q15.6 5. Target Population: Please rate your level of agreement with the statements under optimal and minimal. *Note: The optimal and minimal requirements define a range.*

	1-Disagree (1)	2-Somewhat Disagree (2)	3-Neither Agree nor Disagree (3)	4-Mostly Agree (4)	5-Fully agree (5)	Other - Do not have the expertise to comment (6)
**Optimal:** Neonates ((1)	○	○	○	○	○	○
**Minimal:** Same as Optimal. (2)	○	○	○	○	○	○

Display This Question:

If Please select the newborn technology (ies) that you would like to provide feedback on. (Please Se... = Hemoglobin Test

Q15.7 6. Target Population: Please provide reasoning if you have chosen 3 or below.

____

Display This Question:

If Please select the newborn technology (ies) that you would like to provide feedback on. (Please Se... = Hemoglobin Test

Q15.8 7. Target Setting: Please rate your level of agreement with the statements under optimal and minimal. *Note: The optimal and minimal requirements define a range.*

	1-Disagree (1)	2-Somewhat Disagree (2)	3-Neither Agree nor Disagree (3)	4-Mostly Agree (4)	5-Fully agree (5)	Other - Do not have the expertise to comment (6)
**Optimal:** Hospitals in low-resource settings (1)	o	o	o	o	o	o
**Minimal:** Same as Optimal. (2)	o	o	o	o	o	o

Display This Question:

If Please select the newborn technology (ies) that you would like to provide feedback on. (Please Se... = Hemoglobin Test

Q15.9 8. Target Setting: Please provide reasoning if you have chosen 3 or below.

____

____

____

____

____

Display This Question:

If Please select the newborn technology (ies) that you would like to provide feedback on. (Please Se... = Hemoglobin Test

Q15.10 9. International Standard: Please rate your level of agreement with the statements under optimal and minimal. *Note: The optimal and minimal requirements define a range.*

	1-Disagree (1)	2-Somewhat Disagree (2)	3-Neither Agree nor Disagree (3)	4-Mostly Agree (4)	5-Fully agree (5)	Other - Do not have the expertise to comment (6)
**Optimal:** ISO 13485:2016 Medical devices – Quality management systems -- Requirements for regulatory purposes. (1)	○	○	○	○	○	○
**Minimal:** Same as Optimal. (2)	○	○	○	○	○	○

Display This Question:

If Please select the newborn technology (ies) that you would like to provide feedback on. (Please Se... = Hemoglobin Test

Q15.11 10. International Standard: Please provide reasoning if you have chosen 3 or below.

____

____

____

____

____

Display This Question:

If Please select the newborn technology (ies) that you would like to provide feedback on. (Please Se... = Hemoglobin Test

Q15.12 11. Regulation: Please rate your level of agreement with the statements under optimal and minimal. *Note: The optimal and minimal requirements define a range.*

	1-Disagree (1)	2-Somewhat Disagree (2)	3-Neither Agree nor Disagree (3)	4-Mostly Agree (4)	5-Fully agree (5)	Other - Do not have the expertise to comment (6)
**Optimal:** CE marking or US FDA Clearance (1)	○	○	○	○	○	○
**Minimal:** Same as Optimal. (2)	○	○	○	○	○	○

Display This Question:

If Please select the newborn technology (ies) that you would like to provide feedback on. (Please Se... = Hemoglobin Test

Q15.13 12. Regulation: Please provide reasoning if you have chosen 3 or below.

____

____

____

____

____

Display This Question:

If Please select the newborn technology (ies) that you would like to provide feedback on. (Please Se... = Hemoglobin Test

Q15.14 13. Linear Range: Please rate your level of agreement with the statements under optimal and minimal. *Note: The optimal and minimal requirements define a range.*

	1-Disagree (1)	2-Somewhat Disagree (2)	3-Neither Agree nor Disagree (3)	4-Mostly Agree (4)	5-Fully agree (5)	Other - Do not have the expertise to comment (6)
**Optimal:** 0-25 g/dL (1)	○	○	○	○	○	○
**Minimal:** Same as Optimal. (2)	○	○	○	○	○	○

Display This Question:

If Please select the newborn technology (ies) that you would like to provide feedback on. (Please Se... = Hemoglobin Test

Q15.15 14. Linear Range: Please provide reasoning if you have chosen 3 or below.

____

____

____

____

____

Display This Question:

If Please select the newborn technology (ies) that you would like to provide feedback on. (Please Se... = Hemoglobin Test

Q15.16 15. Accuracy: Please rate your level of agreement with the statements under optimal and minimal. *Note: The optimal and minimal requirements define a range.*

	1-Disagree (1)	2-Somewhat Disagree (2)	3-Neither Agree nor Disagree (3)	4-Mostly Agree (4)	5-Fully agree (5)	Other - Do not have the expertise to comment (6)
**Optimal:** +-1 g/dL (1)	o	o	o	o	o	o
**Minimal:** +-1.75 g/dL (2)	o	o	o	o	o	o

Display This Question:

If Please select the newborn technology (ies) that you would like to provide feedback on. (Please Se... = Hemoglobin Test

Q15.17 16. Accuracy: Please provide reasoning if you have chosen 3 or below.

____

____

____

____

____

Display This Question:

If Please select the newborn technology (ies) that you would like to provide feedback on. (Please Se... = Hemoglobin Test

Q15.18 17. Result Format: Please rate your level of agreement with the statements under optimal and minimal. *Note: The optimal and minimal requirements define a range.*

	1-Disagree (1)	2-Somewhat Disagree (2)	3-Neither Agree nor Disagree (3)	4-Mostly Agree (4)	5-Fully agree (5)	Other - Do not have the expertise to comment (6)
**Optimal:** Quantitative across whole linear range (1)	○	○	○	○	○	○
**Minimal:** Quantitative; semi quantitative below 5 or above 25 g/dL (2)	○	○	○	○	○	○

Display This Question:

If Please select the newborn technology (ies) that you would like to provide feedback on. (Please Se... = Hemoglobin Test

Q15.19 18. Result Format: Please provide reasoning if you have chosen 3 or below.

____

____

____

____

____

Display This Question:

If Please select the newborn technology (ies) that you would like to provide feedback on. (Please Se... = Hemoglobin Test

Q15.20 19. Result Units: Please rate your level of agreement with the statements under optimal and minimal. *Note: The optimal and minimal requirements define a range.*

	1-Disagree (1)	2-Somewhat Disagree (2)	3-Neither Agree nor Disagree (3)	4-Mostly Agree (4)	5-Fully agree (5)	Other - Do not have the expertise to comment (6)
**Optimal:** g/dL OR g/L (1)	○	○	○	○	○	○
**Minimal:** Same as Optimal. (2)	○	○	○	○	○	○

Display This Question:

If Please select the newborn technology (ies) that you would like to provide feedback on. (Please Se... = Hemoglobin Test

Q15.21 20. Result Units: Please provide reasoning if you have chosen 3 or below.

____

____

____

____

____

Display This Question:

If Please select the newborn technology (ies) that you would like to provide feedback on. (Please Se... = Hemoglobin Test

Q15.22 21. Precision: Please rate your level of agreement with the statements under optimal and minimal. *Note: The optimal and minimal requirements define a range.*

	1-Disagree (1)	2-Somewhat Disagree (2)	3-Neither Agree nor Disagree (3)	4-Mostly Agree (4)	5-Fully agree (5)	Other - Do not have the expertise to comment (6)
**Optimal:** 1.5% CV (1)	o	o	o	o	o	o
**Minimal:** 2% CV (2)	o	o	o	o	o	o

Display This Question:

If Please select the newborn technology (ies) that you would like to provide feedback on. (Please Se... = Hemoglobin Test

Q15.23 22. Precision: Please provide reasoning if you have chosen 3 or below.

____

____

____

____

____

Display This Question:

If Please select the newborn technology (ies) that you would like to provide feedback on. (Please Se... = Hemoglobin Test

Q15.24 23. Sample: Please rate your level of agreement with the statements under optimal and minimal. *Note: The optimal and minimal requirements define a range.*

	1-Disagree (1)	2-Somewhat Disagree (2)	3-Neither Agree nor Disagree (3)	4-Mostly Agree (4)	5-Fully agree (5)	Other - Do not have the expertise to comment (6)
**Optimal:** whole blood heel-stick sample (1)	○	○	○	○	○	○
**Minimal:** whole blood heel-stick sample (2)	○	○	○	○	○	○

Display This Question:

If Please select the newborn technology (ies) that you would like to provide feedback on. (Please Se... = Hemoglobin Test

Q15.25 24. Sample: Please provide reasoning if you have chosen 3 or below.

____

____

____

____

____

Display This Question:

If Please select the newborn technology (ies) that you would like to provide feedback on. (Please Se... = Hemoglobin Test

Q15.26 25. Number of Steps: Please rate your level of agreement with the statements under optimal and minimal. *Note: The optimal and minimal requirements define a range.*

	1-Disagree (1)	2-Somewhat Disagree (2)	3-Neither Agree nor Disagree (3)	4-Mostly Agree (4)	5-Fully agree (5)	Other - Do not have the expertise to comment (6)
**Optimal:** No more than 1-3 steps (requiring operator intervention) (1)	o	o	o	o	o	o
**Minimal:** No more than 4-6 steps (requiring operator intervention) (2)	o	o	o	o	o	o

Display This Question:

If Please select the newborn technology (ies) that you would like to provide feedback on. (Please Se... = Hemoglobin Test

Q15.27 26. Number of Steps: Please provide reasoning if you have chosen 3 or below.

____

____

____

____

____

Display This Question:

If Please select the newborn technology (ies) that you would like to provide feedback on. (Please Se... = Hemoglobin Test

Q15.28 27. Calibration: Please rate your level of agreement with the statements under optimal and minimal. *Note: The optimal and minimal requirements define a range.*

	1-Disagree (1)	2-Somewhat Disagree (2)	3-Neither Agree nor Disagree (3)	4-Mostly Agree (4)	5-Fully agree (5)	Other - Do not have the expertise to comment (6)
**Optimal:** No calibration (1)	○	○	○	○	○	○
**Minimal:** Minimal user calibration required (2)	○	○	○	○	○	○

Display This Question:

If Please select the newborn technology (ies) that you would like to provide feedback on. (Please Se... = Hemoglobin Test

Q15.29 28. Calibration: Please provide reasoning if you have chosen 3 or below.

____

____

____

____

____

Display This Question:

If Please select the newborn technology (ies) that you would like to provide feedback on. (Please Se... = Hemoglobin Test

Q15.30 29. Kit Stability & Storage: Please rate your level of agreement with the statements under optimal and minimal. *Note: The optimal and minimal requirements define a range.*

	1-Disagree (1)	2-Somewhat Disagree (2)	3-Neither Agree nor Disagree (3)	4-Mostly Agree (4)	5-Fully agree (5)	Other - Do not have the expertise to comment (6)
**Optimal:** Stable for >12 months with harsh ambient conditions (temperature 5-45 °C, humidity 15% to 95%, dusty air, elevation >=2000 meters) and transport stress (48h with fluctuations up to 50°C and down to 0°C) (1)	○	○	○	○	○	○
**Minimal:** Stable for 12 months with harsh ambient conditions (temperature 10-40 °C, humidity 15%-95% elevation up to 2000 meters) and transport stress (48h with fluctuations up to 50°C and down to	○	○	○	○	○	○

0°C) (2)

Display This Question:

If Please select the newborn technology (ies) that you would like to provide feedback on. (Please Se... = Hemoglobin Test

Q15.31 30. Kit Stability & Storage: Please provide reasoning if you have chosen 3 or below.

____

____

____

____

____

Display This Question:

If Please select the newborn technology (ies) that you would like to provide feedback on. (Please Se... = Hemoglobin Test

Q15.32 31. Equipment Required: Please rate your level of agreement with the statements under optimal and minimal. *Note: The optimal and minimal requirements define a range.*

	1-Disagree (1)	2-Somewhat Disagree (2)	3-Neither Agree nor Disagree (3)	4-Mostly Agree (4)	5-Fully agree (5)	Other - Do not have the expertise to comment (6)
**Optimal:** Small, portable or hand-held device; device-free/disposable preferred (1)	o	o	o	o	o	o
**Minimal:** Small, table-top device; portable device optional (2)	o	o	o	o	o	o

Display This Question:

If Please select the newborn technology (ies) that you would like to provide feedback on. (Please Se... = Hemoglobin Test

Q15.33 32. Equipment Required: Please provide reasoning if you have chosen 3 or below.

____

____

____

____

____

Display This Question:

If Please select the newborn technology (ies) that you would like to provide feedback on. (Please Se... = Hemoglobin Test

Q15.34 33. Power Requirement: Please rate your level of agreement with the statements under optimal and minimal. *Note: The optimal and minimal requirements define a range.*

	1-Disagree (1)	2- Somewhat Disagree (2)	3-Neither Agree nor Disagree (3)	4-Mostly Agree (4)	5-Fully agree (5)	Other - Do not have the expertise to comment (6)
**Optimal:** None (i.e. a disposable test that requires no electricity) (1)	○	○	○	○	○	○
**Minimal:** 110-220V AC current; DC power with rechargeable battery lasting up to 8 hours of testing (2)	○	○	○	○	○	○

Display This Question:

If Please select the newborn technology (ies) that you would like to provide feedback on. (Please Se... = Hemoglobin Test

Q15.35 34. Power Requirement: Please provide reasoning if you have chosen 3 or below.

____

____

____

____

____

Display This Question:

If Please select the newborn technology (ies) that you would like to provide feedback on. (Please Se... = Hemoglobin Test

Q15.36 35. Instrument Pricing: Please rate your level of agreement with the statements under optimal and minimal. *Note: The optimal and minimal requirements define a range.*

	1-Disagree (1)	2-Somewhat Disagree (2)	3-Neither Agree nor Disagree (3)	4-Mostly Agree (4)	5-Fully agree (5)	Other - Do not have the expertise to comment (6)
**Optimal:** (1)	○	○	○	○	○	○
**Minimal:** (2)	○	○	○	○	○	○

Display This Question:

If Please select the newborn technology (ies) that you would like to provide feedback on. (Please Se... = Hemoglobin Test

Q15.37 36. Instrument Pricing: Please provide reasoning if you have chosen 3 or below.

____

____

____

____

____

Display This Question:

If Please select the newborn technology (ies) that you would like to provide feedback on. (Please Se... = Hemoglobin Test

Q15.38 37. Consumable Pricing: Please rate your level of agreement with the statements under optimal and minimal. *Note: The optimal and minimal requirements define a range.*

	1-Disagree (1)	2-Somewhat Disagree (2)	3-Neither Agree nor Disagree (3)	4-Mostly Agree (4)	5-Fully agree (5)	Other - Do not have the expertise to comment (6)
**Optimal:** $0.05 per test ex-works (1)	○	○	○	○	○	○
**Minimal:** $0.50 per test ex-works (2)	○	○	○	○	○	○

Display This Question:

If Please select the newborn technology (ies) that you would like to provide feedback on. (Please Se... = Hemoglobin Test

Q15.39 38. Consumable Pricing: Please provide reasoning if you have chosen 3 or below.

____

____

____

____

____

End of Block: Hemoglobin Test

Start of Block: Glucose Test

Display This Question:

If Please select the newborn technology (ies) that you would like to provide feedback on. (Please Se... = Glucose Test

Q16.1 Glucose Test

Display This Question:

If Please select the newborn technology (ies) that you would like to provide feedback on. (Please Se... = Glucose Test

Q16.2 1. Intended Use: Please rate your level of agreement with the statements under optimal and minimal. *Note: The optimal and minimal requirements define a range.*

	1-Disagree (1)	2- Somewhat Disagree (2)	3-Neither Agree nor Disagree (3)	4-Mostly Agree (4)	5-Fully agree (5)	Other - Do not have the expertise to comment (6)
**Optimal:** Quantitative measurement of blood glucose for diagnosis and management of neonatal hypoglycemia (1)	○	○	○	○	○	○
**Minimal:** Same as Optimal. (2)	○	○	○	○	○	○

Display This Question:

If Please select the newborn technology (ies) that you would like to provide feedback on. (Please Se... = Glucose Test

Q16.3 2. Intended Use: Please provide reasoning if you have chosen 3 or below.

____

____

____

____

____

Display This Question:

If Please select the newborn technology (ies) that you would like to provide feedback on. (Please Se... = Glucose Test

Q16.4 3. Target Operator: Please rate your level of agreement with the statements under optimal and minimal. *Note: The optimal and minimal requirements define a range.*

	1-Disagree (1)	2-Somewhat Disagree (2)	3-Neither agree nor disagree (3)	4-Mostly Agre (4)	5-Fully Agree (5)	Other - Do not have the expertise to comment (6)
**Optimal:** For use in low- and middle-income countries by a wide variety of clinicians, including nurses, clinical officers, and pediatricians. (1)	○	○	○	○	○	○
**Minimal:** Same as Optimal (2)	○	○	○	○	○	○

Display This Question:

If Please select the newborn technology (ies) that you would like to provide feedback on. (Please Se... = Glucose Test

Q16.5 4. Target Operator: Please provide reasoning if you have chosen 3 or below.

____

Display This Question:

If Please select the newborn technology (ies) that you would like to provide feedback on. (Please Se... = Glucose Test

Q16.6 5. Target Population: Please rate your level of agreement with the statements under optimal and minimal. *Note: The optimal and minimal requirements define a range.*

	1-Disagree (1)	2-Somewhat Disagree (2)	3-Neither Agree nor Disagree (3)	4-Mostly Agree (4)	5-Fully agree (5)	Other - Do not have the expertise to comment (6)
**Optimal:** Neonates ((1)	○	○	○	○	○	○
**Minimal:** Same as Optimal. (2)	○	○	○	○	○	○

Display This Question:

If Please select the newborn technology (ies) that you would like to provide feedback on. (Please Se... = Glucose Test

Q16.7 6. Target Population: Please provide reasoning if you have chosen 3 or below.

____

Display This Question:

If Please select the newborn technology (ies) that you would like to provide feedback on. (Please Se... = Glucose Test

Q16.8 7. Target Setting: Please rate your level of agreement with the statements under optimal and minimal. *Note: The optimal and minimal requirements define a range.*

	1-Disagree (1)	2-Somewhat Disagree (2)	3-Neither Agree nor Disagree (3)	4-Mostly Agree (4)	5-Fully agree (5)	Other - Do not have the expertise to comment (6)
**Optimal:** Hospitals in low-resource settings (1)	○	○	○	○	○	○
**Minimal:** Same as Optimal. (2)	○	○	○	○	○	○

Display This Question:

If Please select the newborn technology (ies) that you would like to provide feedback on. (Please Se... = Glucose Test

Q16.9 8. Target Setting: Please provide reasoning if you have chosen 3 or below.

____

____

____

____

____

Display This Question:

If Please select the newborn technology (ies) that you would like to provide feedback on. (Please Se... = Glucose Test

Q16.10 9. International Standard: Please rate your level of agreement with the statements under optimal and minimal. *Note: The optimal and minimal requirements define a range.*

	1-Disagree (1)	2-Somewhat Disagree (2)	3-Neither Agree nor Disagree (3)	4-Mostly Agree (4)	5-Fully agree (5)	Other - Do not have the expertise to comment (6)
**Optimal:** ISO 13485:2016 Medical devices – Quality management systems -- Requirements for regulatory purposes. (1)	○	○	○	○	○	○
**Minimal:** Same as Optimal. (2)	○	○	○	○	○	○

Display This Question:

If Please select the newborn technology (ies) that you would like to provide feedback on. (Please Se... = Glucose Test

Q16.11 10. International Standard: Please provide reasoning if you have chosen 3 or below.

____

____

____

____

____

Display This Question:

If Please select the newborn technology (ies) that you would like to provide feedback on. (Please Se... = Glucose Test

Q16.12 11. Regulation: Please rate your level of agreement with the statements under optimal and minimal. *Note: The optimal and minimal requirements define a range.*

	1-Disagree (1)	2-Somewhat Disagree (2)	3-Neither Agree nor Disagree (3)	4-Mostly Agree (4)	5-Fully agree (5)	Other - Do not have the expertise to comment (6)
**Optimal:** CE marking or US FDA Clearance (1)	○	○	○	○	○	○
**Minimal:** Same as Optimal. (2)	○	○	○	○	○	○

Display This Question:

If Please select the newborn technology (ies) that you would like to provide feedback on. (Please Se... = Glucose Test

Q16.13 12. Regulation: Please provide reasoning if you have chosen 3 or below.

____

____

____

____

____

Display This Question:

If Please select the newborn technology (ies) that you would like to provide feedback on. (Please Se... = Glucose Test

Q16.14 13. Linear Range: Please rate your level of agreement with the statements under optimal and minimal. *Note: The optimal and minimal requirements define a range.*

	1-Disagree (1)	2-Somewhat Disagree (2)	3-Neither Agree nor Disagree (3)	4-Mostly Agree (4)	5-Fully agree (5)	Other - Do not have the expertise to comment (6)
**Optimal:** 0-50 mmol/L (0-900 mg/dL) (1)	○	○	○	○	○	○
**Minimal:** 0-20 mmol/L (0-360 mg/dL) (2)	○	○	○	○	○	○

Display This Question:

If Please select the newborn technology (ies) that you would like to provide feedback on. (Please Se... = Glucose Test

Q16.15 14. Linear Range: Please provide reasoning if you have chosen 3 or below.

____

____

____

____

____

Display This Question:

If Please select the newborn technology (ies) that you would like to provide feedback on. (Please Se... = Glucose Test

Q16.16 15. Accuracy: Please rate your level of agreement with the statements under optimal and minimal. *Note: The optimal and minimal requirements define a range.*

	1-Disagree (1)	2-Somewhat Disagree (2)	3-Neither Agree nor Disagree (3)	4-Mostly Agree (4)	5-Fully agree (5)	Other - Do not have the expertise to comment (6)
**Optimal:** ± 0.2 mmol/L at 2.5 mmol/L (± 3.6 mg/dL at 45 mg/dL) (1)	o	o	o	o	o	o
**Minimal:** ± 0.2 mmol/L at 3 mmol/L (± 3.6 mg/dL at 54 mg/dL) (2)	o	o	o	o	o	o

Display This Question:

If Please select the newborn technology (ies) that you would like to provide feedback on. (Please Se... = Glucose Test

Q16.17 16. Accuracy: Please provide reasoning if you have chosen 3 or below.

____

____

____

____

____

Display This Question:

If Please select the newborn technology (ies) that you would like to provide feedback on. (Please Se... = Glucose Test

Q16.18 17. Result Format: Please rate your level of agreement with the statements under optimal and minimal. *Note: The optimal and minimal requirements define a range.*

	1-Disagree (1)	2-Somewhat Disagree (2)	3-Neither Agree nor Disagree (3)	4-Mostly Agree (4)	5-Fully agree (5)	Other - Do not have the expertise to comment (6)
**Optimal:** Quantitative across whole linear range (1)	o	o	o	o	o	o
**Minimal:** Quantitative; semi quantitative at (2)	o	o	o	o	o	o

Display This Question:

If Please select the newborn technology (ies) that you would like to provide feedback on. (Please Se... = Glucose Test

Q16.19 18. Result Format: Please provide reasoning if you have chosen 3 or below.

____

____

____

____

____

Display This Question:

If Please select the newborn technology (ies) that you would like to provide feedback on. (Please Se... = Glucose Test

Q16.20 19. Result Units: Please rate your level of agreement with the statements under optimal and minimal. *Note: The optimal and minimal requirements define a range.*

	1-Disagree (1)	2-Somewhat Disagree (2)	3-Neither Agree nor Disagree (3)	4-Mostly Agree (4)	5-Fully agree (5)	Other - Do not have the expertise to comment (6)
**Optimal:** mg/dL OR mmol/L (1)	○	○	○	○	○	○
**Minimal:** Same as Optimal. (2)	○	○	○	○	○	○

Display This Question:

If Please select the newborn technology (ies) that you would like to provide feedback on. (Please Se... = Glucose Test

Q16.21 20. Result Units: Please provide reasoning if you have chosen 3 or below.

____

____

____

____

____

Display This Question:

If Please select the newborn technology (ies) that you would like to provide feedback on. (Please Se... = Glucose Test

Q16.22 21. Precision: Please rate your level of agreement with the statements under optimal and minimal. *Note: The optimal and minimal requirements define a range.*

	1-Disagree (1)	2-Somewhat Disagree (2)	3-Neither Agree nor Disagree (3)	4-Mostly Agree (4)	5-Fully agree (5)	Other - Do not have the expertise to comment (6)
**Optimal:** +-2% or 2.5 mg/dL, whichever is greater (1)	○	○	○	○	○	○
**Minimal:** 5% CV (2)	○	○	○	○	○	○

Display This Question:

If Please select the newborn technology (ies) that you would like to provide feedback on. (Please Se... = Glucose Test

Q16.23 22. Precision: Please provide reasoning if you have chosen 3 or below.

____

____

____

____

____

Display This Question:

If Please select the newborn technology (ies) that you would like to provide feedback on. (Please Se... = Glucose Test

Q16.24 23. Sample: Please rate your level of agreement with the statements under optimal and minimal. *Note: The optimal and minimal requirements define a range.*

	1-Disagree (1)	2-Somewhat Disagree (2)	3-Neither Agree nor Disagree (3)	4-Mostly Agree (4)	5-Fully agree (5)	Other - Do not have the expertise to comment (6)
**Optimal:** whole blood heel-stick sample (1)	o	o	o	o	o	o
**Minimal:** whole blood heel-stick sample (2)	o	o	o	o	o	o

Display This Question:

If Please select the newborn technology (ies) that you would like to provide feedback on. (Please Se... = Glucose Test

Q16.25 24. Sample: Please provide reasoning if you have chosen 3 or below.

____

____

____

____

____

Display This Question:

If Please select the newborn technology (ies) that you would like to provide feedback on. (Please Se... = Glucose Test

Q16.26 25. Calibration: Please rate your level of agreement with the statements under optimal and minimal. *Note: The optimal and minimal requirements define a range.*

	1-Disagree (1)	2-Somewhat Disagree (2)	3-Neither Agree nor Disagree (3)	4-Mostly Agree (4)	5-Fully agree (5)	Other - Do not have the expertise to comment (6)
**Optimal:** No calibration (1)	o	o	o	o	o	o
**Minimal:** Minimal user calibration required (2)	o	o	o	o	o	o

Display This Question:

If Please select the newborn technology (ies) that you would like to provide feedback on. (Please Se... = Glucose Test

Q16.27 26. Calibration: Please provide reasoning if you have chosen 3 or below.

____

____

____

____

____

Display This Question:

If Please select the newborn technology (ies) that you would like to provide feedback on. (Please Se... = Glucose Test

Q16.28 27. Kit Stability & Storage: Please rate your level of agreement with the statements under optimal and minimal. *Note: The optimal and minimal requirements define a range.*

	1-Disagree (1)	2-Somewhat Disagree (2)	3-Neither Agree nor Disagree (3)	4-Mostly Agree (4)	5-Fully agree (5)	Other - Do not have the expertise to comment (6)
**Optimal:** Stable for >12 months with harsh ambient conditions (temperature 5-45 °C, humidity 15% to 95%, dusty air, elevation >=2000 meters) and transport stress (48h with fluctuations up to 50°C and down to 0°C) (1)	o	o	o	o	o	o
**Minimal:** Stable for 12 months with harsh ambient conditions (temperature 10-40 °C, humidity 15%-95% elevation up to 2000 meters) and transport stress (48h with fluctuations up to 50°C and down to	o	o	o	o	o	o

0°C) (2)

Display This Question:

If Please select the newborn technology (ies) that you would like to provide feedback on. (Please Se... = Glucose Test

Q16.29 28. Kit Stability & Storage: Please provide reasoning if you have chosen 3 or below.

____

____

____

____

____

Display This Question:

If Please select the newborn technology (ies) that you would like to provide feedback on. (Please Se... = Glucose Test

Q16.30 29. Equipment Required: Please rate your level of agreement with the statements under optimal and minimal. *Note: The optimal and minimal requirements define a range.*

	1-Disagree (1)	2-Somewhat Disagree (2)	3-Neither Agree nor Disagree (3)	4-Mostly Agree (4)	5-Fully agree (5)	Other - Do not have the expertise to comment (6)
**Optimal:** Small, portable or hand-held device; device-free/disposable preferred (1)	o	o	o	o	o	o
**Minimal:** Small, table-top device; portable device optional (2)	o	o	o	o	o	o

Display This Question:

If Please select the newborn technology (ies) that you would like to provide feedback on. (Please Se... = Glucose Test

Q16.31 30. Equipment Required: Please provide reasoning if you have chosen 3 or below.

____

____

____

____

____

Display This Question:

If Please select the newborn technology (ies) that you would like to provide feedback on. (Please Se... = Glucose Test

Q16.32 31. Voltage: Please rate your level of agreement with the statements under optimal and minimal. *Note: The optimal and minimal requirements define a range.*

	1-Disagree (1)	2-Somewhat Disagree (2)	3-Neither Agree nor Disagree (3)	4-Mostly Agree (4)	5-Fully agree (5)	Other - Do not have the expertise to comment (6)
**Optimal:** 110-240 50-60hz (1)	○	○	○	○	○	○
**Minimal:** 220-240 50-60hz (2)	○	○	○	○	○	○

Display This Question:

If Please select the newborn technology (ies) that you would like to provide feedback on. (Please Se... = Glucose Test

Q16.33 32. Voltage: Please provide reasoning if you have chosen 3 or below.

____

____

____

____

____

Display This Question:

If Please select the newborn technology (ies) that you would like to provide feedback on. (Please Se... = Glucose Test

Q16.34 33. Power Requirement: Please rate your level of agreement with the statements under optimal and minimal. *Note: The optimal and minimal requirements define a range.*

	1-Disagree (1)	2-Somewhat Disagree (2)	3-Neither Agree nor Disagree (3)	4-Mostly Agree (4)	5-Fully agree (5)	Other - Do not have the expertise to comment (6)
**Optimal:** >4hr on single charge (1)	o	o	o	o	o	o
**Minimal:** None (2)	o	o	o	o	o	o

Display This Question:

If Please select the newborn technology (ies) that you would like to provide feedback on. (Please Se... = Glucose Test

Q16.35 34. Power Requirement: Please provide reasoning if you have chosen 3 or below.

____

____

____

____

____

Display This Question:

If Please select the newborn technology (ies) that you would like to provide feedback on. (Please Se... = Glucose Test

Q16.36 35. Instrument Pricing: Please rate your level of agreement with the statements under optimal and minimal. *Note: The optimal and minimal requirements define a range.*

	1-Disagree (1)	2-Somewhat Disagree (2)	3-Neither Agree nor Disagree (3)	4-Mostly Agree (4)	5-Fully agree (5)	Other - Do not have the expertise to comment (6)
**Optimal:** (1)	○	○	○	○	○	○
**Minimal:** (2)	○	○	○	○	○	○

Display This Question:

If Please select the newborn technology (ies) that you would like to provide feedback on. (Please Se... = Glucose Test

Q16.37 36. Instrument Pricing: Please provide reasoning if you have chosen 3 or below.

____

____

____

____

____

Display This Question:

If Please select the newborn technology (ies) that you would like to provide feedback on. (Please Se... = Glucose Test

Q16.38 37. Consumable Pricing: Please rate your level of agreement with the statements under optimal and minimal. *Note: The optimal and minimal requirements define a range.*

	1-Disagree (1)	2-Somewhat Disagree (2)	3-Neither Agree nor Disagree (3)	4-Mostly Agree (4)	5-Fully agree (5)	Other - Do not have the expertise to comment (6)
**Optimal:** $0.05 per test ex-works (1)	○	○	○	○	○	○
**Minimal:** $1.50 per test ex-works (2)	○	○	○	○	○	○

Display This Question:

If Please select the newborn technology (ies) that you would like to provide feedback on. (Please Se... = Glucose Test

Q16.39 38. Consumable Pricing: Please provide reasoning if you have chosen 3 or below.

____

____

____

____

____

End of Block: Glucose Test

Start of Block: pH Test

Display This Question:

If Please select the newborn technology (ies) that you would like to provide feedback on. (Please Se... = pH Test

Q17.1 pH Test

Display This Question:

If Please select the newborn technology (ies) that you would like to provide feedback on. (Please Se... = pH Test

Q17.2 1. Intended Use: Please rate your level of agreement with the statements under optimal and minimal. *Note: The optimal and minimal requirements define a range.*

	1-Disagree (1)	2-Somewhat Disagree (2)	3-Neither Agree nor Disagree (3)	4-Mostly Agree (4)	5-Fully agree (5)	Other - Do not have the expertise to comment (6)
**Optimal:** Quantitative measurement of pH for diagnosis and management of metabolic acidosis and/or respiratory acidosis. (1)	○	○	○	○	○	○
**Minimal:** Same as Optimal. (2)	○	○	○	○	○	○

Display This Question:

If Please select the newborn technology (ies) that you would like to provide feedback on. (Please Se... = pH Test

Q17.3 2. Intended Use: Please provide reasoning if you have chosen 3 or below.

____

____

____

____

____

Display This Question:

If Please select the newborn technology (ies) that you would like to provide feedback on. (Please Se... = pH Test

Q17.4 3. Target Operator: Please rate your level of agreement with the statements under optimal and minimal. *Note: The optimal and minimal requirements define a range.*

	1-Disagree (1)	2-Somewhat Disagree (2)	3-Neither agree nor disagree (3)	4-Mostly Agre (4)	5-Fully Agree (5)	Other - Do not have the expertise to comment (6)
**Optimal:** For use in low- and middle-income countries by a wide variety of clinicians, including nurses, clinical officers, and pediatricians. (1)	○	○	○	○	○	○
**Minimal:** Same as Optimal (2)	○	○	○	○	○	○

Display This Question:

If Please select the newborn technology (ies) that you would like to provide feedback on. (Please Se... = pH Test

Q17.5 4. Target Operator: Please provide reasoning if you have chosen 3 or below.

____

Display This Question:

If Please select the newborn technology (ies) that you would like to provide feedback on. (Please Se... = pH Test

Q17.6 5. Target Population: Please rate your level of agreement with the statements under optimal and minimal. *Note: The optimal and minimal requirements define a range.*

	1-Disagree (1)	2-Somewhat Disagree (2)	3-Neither Agree nor Disagree (3)	4-Mostly Agree (4)	5-Fully agree (5)	Other - Do not have the expertise to comment (6)
**Optimal:** Neonates ((1)	○	○	○	○	○	○
**Minimal:** Same as Optimal. (2)	○	○	○	○	○	○

Display This Question:

If Please select the newborn technology (ies) that you would like to provide feedback on. (Please Se... = pH Test

Q17.7 6. Target Population: Please provide reasoning if you have chosen 3 or below.

____

Display This Question:

If Please select the newborn technology (ies) that you would like to provide feedback on. (Please Se... = pH Test

Q17.8 7. Target Setting: Please rate your level of agreement with the statements under optimal and minimal. *Note: The optimal and minimal requirements define a range.*

	1-Disagree (1)	2-Somewhat Disagree (2)	3-Neither Agree nor Disagree (3)	4-Mostly Agree (4)	5-Fully agree (5)	Other - Do not have the expertise to comment (6)
**Optimal:** Hospitals in low-resource settings (1)	o	o	o	o	o	o
**Minimal:** Same as Optimal. (2)	o	o	o	o	o	o

Display This Question:

If Please select the newborn technology (ies) that you would like to provide feedback on. (Please Se... = pH Test

Q17.9 8. Target Setting: Please provide reasoning if you have chosen 3 or below.

____

____

____

____

____

Display This Question:

If Please select the newborn technology (ies) that you would like to provide feedback on. (Please Se... = pH Test

Q17.10 9. International Standard: Please rate your level of agreement with the statements under optimal and minimal. *Note: The optimal and minimal requirements define a range.*

	1-Disagree (1)	2-Somewhat Disagree (2)	3-Neither Agree nor Disagree (3)	4-Mostly Agree (4)	5-Fully agree (5)	Other - Do not have the expertise to comment (6)
**Optimal:** ISO 13485:2016 Medical devices – Quality management systems -- Requirements for regulatory purposes. (1)	o	o	o	o	o	o
**Minimal:** Same as Optimal. (2)	o	o	o	o	o	o

Display This Question:

If Please select the newborn technology (ies) that you would like to provide feedback on. (Please Se... = pH Test

Q17.11 10. International Standard: Please provide reasoning if you have chosen 3 or below.

____

____

____

____

____

Display This Question:

If Please select the newborn technology (ies) that you would like to provide feedback on. (Please Se... = pH Test

Q17.12 11. Regulation: Please rate your level of agreement with the statements under optimal and minimal. *Note: The optimal and minimal requirements define a range.*

	1-Disagree (1)	2-Somewhat Disagree (2)	3-Neither Agree nor Disagree (3)	4-Mostly Agree (4)	5-Fully agree (5)	Other - Do not have the expertise to comment (6)
**Optimal:** CE marking or US FDA Clearance (1)	○	○	○	○	○	○
**Minimal:** Same as Optimal. (2)	○	○	○	○	○	○

Display This Question:

If Please select the newborn technology (ies) that you would like to provide feedback on. (Please Se... = pH Test

Q17.13 12. Regulation: Please provide reasoning if you have chosen 3 or below.

____

____

____

____

____

Display This Question:

If Please select the newborn technology (ies) that you would like to provide feedback on. (Please Se... = pH Test

Q17.14 13. Linear Range: Please rate your level of agreement with the statements under optimal and minimal. *Note: The optimal and minimal requirements define a range.*

	1-Disagree (1)	2-Somewhat Disagree (2)	3-Neither Agree nor Disagree (3)	4-Mostly Agree (4)	5-Fully agree (5)	Other - Do not have the expertise to comment (6)
**Optimal:** 6.5-8.2 (1)	o	o	o	o	o	o
**Minimal:** 6.9-7.45 (2)	o	o	o	o	o	o

Display This Question:

If Please select the newborn technology (ies) that you would like to provide feedback on. (Please Se... = pH Test

Q17.15 14. Linear Range: Please provide reasoning if you have chosen 3 or below.

____

____

____

____

____

Display This Question:

If Please select the newborn technology (ies) that you would like to provide feedback on. (Please Se... = pH Test

Q17.16 15. Accuracy: Please rate your level of agreement with the statements under optimal and minimal. *Note: The optimal and minimal requirements define a range.*

	1-Disagree (1)	2-Somewhat Disagree (2)	3-Neither Agree nor Disagree (3)	4-Mostly Agree (4)	5-Fully agree (5)	Other - Do not have the expertise to comment (6)
**Optimal:** ± 0.04 (1)	○	○	○	○	○	○
Minimal: Same as Optimal. (3)	○	○	○	○	○	○

Display This Question:

If Please select the newborn technology (ies) that you would like to provide feedback on. (Please Se... = pH Test

Q17.17 16. Accuracy: Please provide reasoning if you have chosen 3 or below.

____

____

____

____

____

Display This Question:

If Please select the newborn technology (ies) that you would like to provide feedback on. (Please Se... = pH Test

Q17.18 17. Precision: Please rate your level of agreement with the statements under optimal and minimal. *Note: The optimal and minimal requirements define a range.*

	1-Disagree (1)	2-Somewhat Disagree (2)	3-Neither Agree nor Disagree (3)	4-Mostly Agree (4)	5-Fully agree (5)	Other - Do not have the expertise to comment (6)
**Optimal:** +-0.01 (1)	o	o	o	o	o	o
**Minimal:** Same as Optimal. (2)	o	o	o	o	o	o

Display This Question:

If Please select the newborn technology (ies) that you would like to provide feedback on. (Please Se... = pH Test

Q17.19 18. Precision: Please provide reasoning if you have chosen 3 or below.

____

____

____

____

____

Display This Question:

If Please select the newborn technology (ies) that you would like to provide feedback on. (Please Se... = pH Test

Q17.20 19. Sample: Please rate your level of agreement with the statements under optimal and minimal. *Note: The optimal and minimal requirements define a range.*

	1-Disagree (1)	2-Somewhat Disagree (2)	3-Neither Agree nor Disagree (3)	4-Mostly Agree (4)	5-Fully agree (5)	Other - Do not have the expertise to comment (6)
**Optimal:** whole blood heel-stick sample (1)	○	○	○	○	○	○
**Minimal:** whole blood heel-stick sample (2)	○	○	○	○	○	○

Display This Question:

If Please select the newborn technology (ies) that you would like to provide feedback on. (Please Se... = pH Test

Q17.21 20. Sample: Please provide reasoning if you have chosen 3 or below.

____

____

____

____

____

Display This Question:

If Please select the newborn technology (ies) that you would like to provide feedback on. (Please Se... = pH Test

Q17.22 21. Results Format: Please rate your level of agreement with the statements under optimal and minimal. *Note: The optimal and minimal requirements define a range.*

	1-Disagree (1)	2-Somewhat Disagree (2)	3-Neither Agree nor Disagree (3)	4-Mostly Agree (4)	5-Fully agree (5)	Other - Do not have the expertise to comment (6)
**Optimal:** Quantitative (1)	○	○	○	○	○	○
**Minimal:** Same as Optimal. (2)	○	○	○	○	○	○

Display This Question:

If Please select the newborn technology (ies) that you would like to provide feedback on. (Please Se... = pH Test

Q17.23 22. Results Format: Please provide reasoning if you have chosen 3 or below.

____

____

____

____

____

Display This Question:

If Please select the newborn technology (ies) that you would like to provide feedback on. (Please Se... = pH Test

Q17.24 23. Calibration: Please rate your level of agreement with the statements under optimal and minimal. *Note: The optimal and minimal requirements define a range.*

	1-Disagree (1)	2-Somewhat Disagree (2)	3-Neither Agree nor Disagree (3)	4-Mostly Agree (4)	5-Fully agree (5)	Other - Do not have the expertise to comment (6)
**Optimal:** No calibration (1)	○	○	○	○	○	○
**Minimal:** Minimal user calibration required (2)	○	○	○	○	○	○

Display This Question:

If Please select the newborn technology (ies) that you would like to provide feedback on. (Please Se... = pH Test

Q17.25 24. Calibration: Please provide reasoning if you have chosen 3 or below.

____

____

____

____

____

Display This Question:

If Please select the newborn technology (ies) that you would like to provide feedback on. (Please Se... = pH Test

Q17.26 25. Kit Stability & Storage: Please rate your level of agreement with the statements under optimal and minimal. *Note: The optimal and minimal requirements define a range.*

	1-Disagree (1)	2-Somewhat Disagree (2)	3-Neither Agree nor Disagree (3)	4-Mostly Agree (4)	5-Fully agree (5)	Other - Do not have the expertise to comment (6)
**Optimal:** Stable for >12 months with harsh ambient conditions (temperature 5-45 °C, humidity 15% to 95%, dusty air, elevation >=2000 meters) and transport stress (48h with fluctuations up to 50°C and down to 0°C) (1)	○	○	○	○	○	○
**Minimal:** Stable for 12 months with harsh ambient conditions (temperature 10-40 °C, humidity 15%-95% elevation up to 2000 meters) and transport stress (48h with fluctuations up to 50°C and down to	○	○	○	○	○	○

0°C) (2)

Display This Question:

If Please select the newborn technology (ies) that you would like to provide feedback on. (Please Se... = pH Test

Q17.27 26. Kit Stability & Storage: Please provide reasoning if you have chosen 3 or below.

____

____

____

____

____

Display This Question:

If Please select the newborn technology (ies) that you would like to provide feedback on. (Please Se... = pH Test

Q17.28 27. Equipment Required: Please rate your level of agreement with the statements under optimal and minimal. *Note: The optimal and minimal requirements define a range.*

	1-Disagree (1)	2-Somewhat Disagree (2)	3-Neither Agree nor Disagree (3)	4-Mostly Agree (4)	5-Fully agree (5)	Other - Do not have the expertise to comment (6)
**Optimal:** Small, portable or hand-held device; device-free/disposable preferred (1)	○	○	○	○	○	○
**Minimal:** Small, table-top device; portable device optional (2)	○	○	○	○	○	○

Display This Question:

If Please select the newborn technology (ies) that you would like to provide feedback on. (Please Se... = pH Test

Q17.29 28. Equipment Required: Please provide reasoning if you have chosen 3 or below.

____

____

____

____

____

Display This Question:

If Please select the newborn technology (ies) that you would like to provide feedback on. (Please Se... = pH Test

Q17.30 29. Voltage: Please rate your level of agreement with the statements under optimal and minimal. *Note: The optimal and minimal requirements define a range.*

	1-Disagree (1)	2-Somewhat Disagree (2)	3-Neither Agree nor Disagree (3)	4-Mostly Agree (4)	5-Fully agree (5)	Other - Do not have the expertise to comment (6)
**Optimal:** 110-240 50-60hz (1)	o	o	o	o	o	o
**Minimal:** 220-240 50-60hz (2)	o	o	o	o	o	o

Display This Question:

If Please select the newborn technology (ies) that you would like to provide feedback on. (Please Se... = pH Test

Q17.31 30. Voltage: Please provide reasoning if you have chosen 3 or below.

____

____

____

____

____

Display This Question:

If Please select the newborn technology (ies) that you would like to provide feedback on. (Please Se... = pH Test

Q17.32 31. Power Requirement: Please rate your level of agreement with the statements under optimal and minimal. *Note: The optimal and minimal requirements define a range.*

	1-Disagree (1)	2-Somewhat Disagree (2)	3-Neither Agree nor Disagree (3)	4-Mostly Agree (4)	5-Fully agree (5)	Other - Do not have the expertise to comment (6)
**Optimal:** >4hr on single charge (1)	○	○	○	○	○	○
**Minimal:** None (2)	○	○	○	○	○	○

Display This Question:

If Please select the newborn technology (ies) that you would like to provide feedback on. (Please Se... = pH Test

Q17.33 32. Power Requirement: Please provide reasoning if you have chosen 3 or below.

____

____

____

____

____

Display This Question:

If Please select the newborn technology (ies) that you would like to provide feedback on. (Please Se... = pH Test

Q17.34 33. Time to Result: Please rate your level of agreement with the statements under optimal and minimal. *Note: The optimal and minimal requirements define a range.*

	1-Disagree (1)	2-Somewhat Disagree (2)	3-Neither Agree nor Disagree (3)	4-Mostly Agree (4)	5-Fully agree (5)	Other - Do not have the expertise to comment (6)
**Optimal:** (1)	○	○	○	○	○	○
**Minimal:** (2)	○	○	○	○	○	○

Display This Question:

If Please select the newborn technology (ies) that you would like to provide feedback on. (Please Se... = pH Test

Q17.35 34. Time to Result: Please provide reasoning if you have chosen 3 or below.

____

____

____

____

____

Display This Question:

If Please select the newborn technology (ies) that you would like to provide feedback on. (Please Se... = pH Test

Q17.36 35. Instrument Pricing: Please rate your level of agreement with the statements under optimal and minimal. *Note: The optimal and minimal requirements define a range.*

	1-Disagree (1)	2-Somewhat Disagree (2)	3-Neither Agree nor Disagree (3)	4-Mostly Agree (4)	5-Fully agree (5)	Other - Do not have the expertise to comment (6)
**Optimal:** (1)	○	○	○	○	○	○
**Minimal:** (2)	○	○	○	○	○	○

Display This Question:

If Please select the newborn technology (ies) that you would like to provide feedback on. (Please Se... = pH Test

Q17.37 36. Instrument Pricing: Please provide reasoning if you have chosen 3 or below.

____

____

____

____

____

Display This Question:

If Please select the newborn technology (ies) that you would like to provide feedback on. (Please Se... = pH Test

Q17.38 37. Consumable Pricing: Please rate your level of agreement with the statements under optimal and minimal. *Note: The optimal and minimal requirements define a range.*

	1-Disagree (1)	2-Somewhat Disagree (2)	3-Neither Agree nor Disagree (3)	4-Mostly Agree (4)	5-Fully agree (5)	Other - Do not have the expertise to comment (6)
**Optimal:** $0.05 per test ex-works (1)	○	○	○	○	○	○
**Minimal:** $1.50 per test ex-works (2)	○	○	○	○	○	○

Display This Question:

If Please select the newborn technology (ies) that you would like to provide feedback on. (Please Se... = pH Test

Q17.39 38. Consumable Pricing: Please provide reasoning if you have chosen 3 or below.

____

____

____

____

____

End of Block: pH Test

Start of Block: Other

Display This Question:

If Please select the newborn technology (ies) that you would like to provide feedback on. (Please Se... = Other

Q18.1 Please list the other technologies, not covered in this survey, that should be included as part of Comprehensive Newborn Care.

____

End of Block: Other

Start of Block: Survey Wrap-Up

Q19.1 Optional: If you are interested in providing more feedback on newborn technologies, please list your contact information below.

○ First Name (1) ____

○ Last Name (2) ____

○ Organization Name (3) ____

○ Title (4) ____

○ Email (5) ____

Q19.2 Do you have recommendations for other people that could potentially fill this survey out? If so, please list their contact information below.

○ Contact #1 (1) ____

○ Contact #2 (2) ____

○ Contact #3 (3) ____

Q19.3 Thank you for completing this survey. Are there any additional comments you think we should consider?

____

____

____

End of Block: Survey Wrap-Up
